# Comprehensive profiling of circular RNA expressions reveals potential diagnostic and prognostic biomarkers in multiple myeloma

**DOI:** 10.1186/s12885-020-6515-2

**Published:** 2020-01-16

**Authors:** Fan Zhou, Dongjiao Wang, Wei Wei, Haimin Chen, Haotian Shi, Nian Zhou, Lixia Wu, Rong Peng

**Affiliations:** 1 Department of Hematology and Oncology, Shanghai Jing’an District Zhabei Central Hospital, Shanghai, China; 2 Myeloma cooperative group of Shanghai district and county blood alliance, Shanghai, China

**Keywords:** Circular RNA, Expression profiles, Bioinformatic analysis, Multiple myeloma, Diagnostic and prognostic value

## Abstract

**Background:**

This study aimed to explore the heterogeneity of circRNA expression pattern via microarray, and further evaluate the potential of 10 specific circRNAs as diagnostic and prognostic biomarkers in multiple myeloma (MM).

**Methods:**

In exploration stage (stage I), circRNA expression profiles were detected by the microarray in bone marrow plasma cells from 4 MM patients and 4 healthy controls (HCs), and bioinformatic analyses were performed. In validation stage (stage II), top 10 upregulated and top 10 downregulated circRNAs identified in stage I were detected in 60 MM patients and 30 HCs for further validation; the diagnostic and prognostic values of these circRNAs in MM patients were analyzed.

**Results:**

In stage I, 122 upregulated and 260 downregulated circRNAs were identified in MM patients compared with HCs. GO, KEGG and pathway enrichment analyses revealed that these circRNAs were implicated in neoplastic pathways such as MAPK and VEGF signaling pathways. In stage II, circ-PTK2, circ-RNF217, circ-RERE, circ-NAGPA and circ-KCNQ5 were validated to be upregulated and circ-AFF2, circ-WWC3, circ-DNAJC5, circ-KLHL2, circ-IQGAP1 and circ-AL137655 were validated to be downregulated in MM compared with controls. Circ-PTK2 and circ-RNF217 were correlated with poor treatment response and survival, while circ-AFF2 predicted good treatment response and survival in MM patients.

**Conclusions:**

This study provides valuable reference for profound understanding about circRNA expression patterns in MM, and validates that circ-PTK2, circ-RNF217 and circ-AFF2 might serve as potential prognostic biomarkers in MM.

## Background

Multiple myeloma (MM) is the second most common hematological malignancy derived from long-lived antibody-producing plasma cells in the bone marrow and is characterized by the presence of monoclonal immunoglobins in the serum and/or urine [1]. Over the past half century, the introduction of novel drugs (such as bortezomib) and application of hematopoietic stem cell transplantation have turned the rapid lethal MM into a chronic and manageable disease with extended survival in most of the patients [2, 3]. However, MM lacks symptoms in early stage, and the identification of the disease onset is difficult to be achieved by current examinations [4]. Moreover, the obstacles in treatment such as relapse and multidrug resistance are still common, contributing to poor prognosis in MM patients [5]. Therefore, it is essential to explore novel biomarkers that would help with diagnosis and improve the survival in MM patients.

Circular RNAs (circRNAs) are a class of non-coding RNAs originated by back-splicing of the precursor messenger RNA and forming a covalent loop with no 5′ to 3′ polarity or polyadenylated tail [6]. CircRNAs are stable, abundant and evolutionarily conserved, and mounting studies have proven that they contain target sites for microRNAs (miRNAs), thereby participate in the pathogenesis of various diseases through disturbing miRNAs signal axis [7]. With the development of circRNA microarrays, our knowledge about circRNA expression patterns has been initially uncovered in various diseases. In cancer research, circRNA expression patterns have been studied in some solid tumors including breast cancer, esophageal squamous cell cancer, epithelial ovarian cancer, etc., and a number of circRNAs are disclosed to involve in the pathophysiological progression of these malignancies [8–11]. As for hematological malignancies, an extensive analysis of circRNA expression profiles reveals a total of 464 dysregulated circRNAs (147 upregulated and 317 downregulated) in acute myeloid leukemia (AML) patients compared with healthy controls, and among these circRNAs, circ_0004277 is validated to be positively associated with prognosis in AML patients [9]. Whereas in MM, the expression profiles of circRNAs are not yet reported. Concidering that circRNAs are differentially expressed and involve in the pathophysiological progression of solid tumors as well as hematological malignancies, we speculated that they might play critical roles in MM as well.

This present study aimed to investigate the heterogeneity of circRNA expression pattern via microarray, and further evaluate the potential of 10 specific circRNAs as diagnostic and prognostic biomarkers in MM.

## Methods

### Study design

**Fig. 1:**
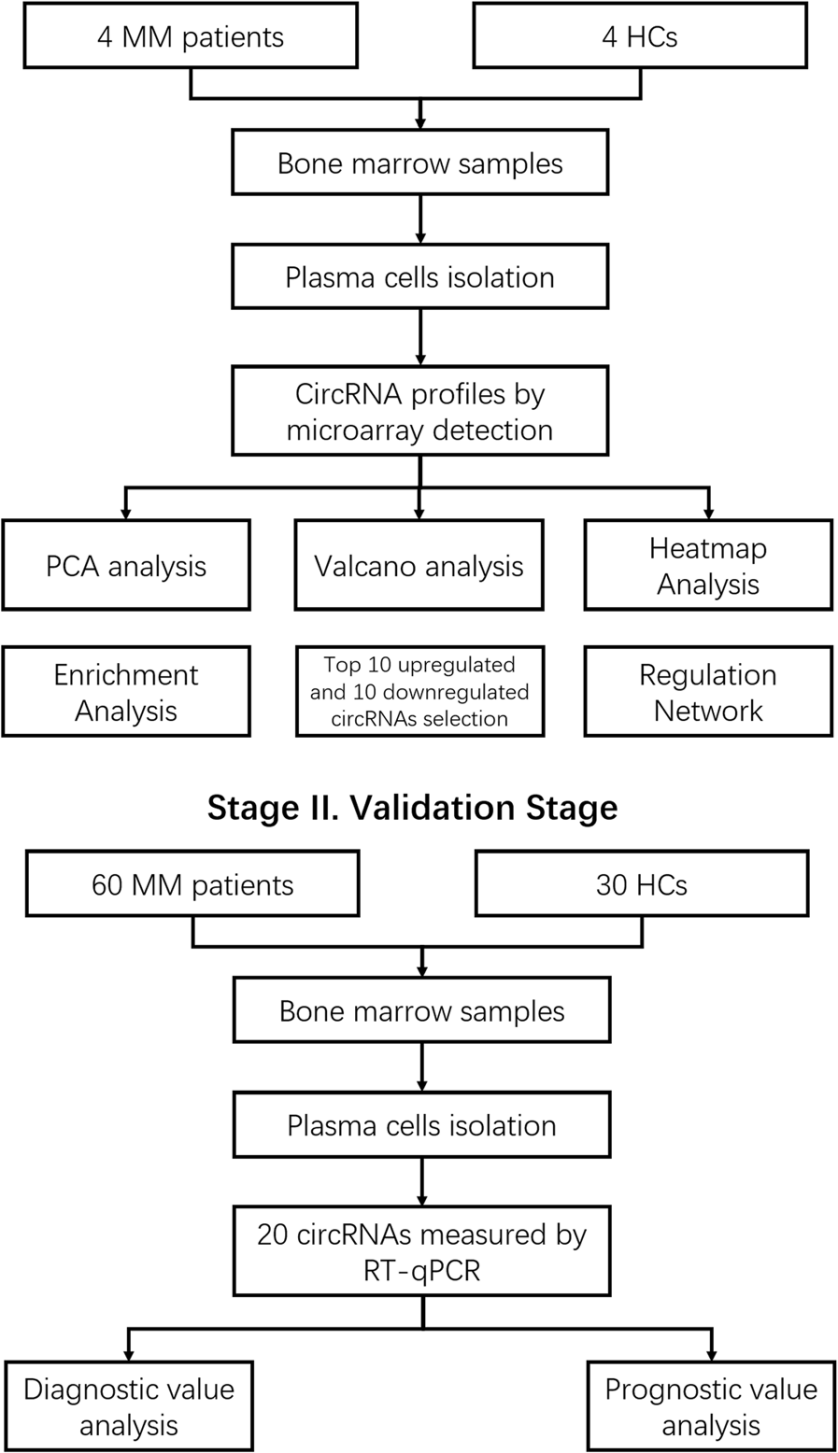
Study Flow. MM, multiple myeloma; HCs, healthy controls; PCA, principal component analysis; RT-qPCR, Real Time quantitative polymerase chain reaction

This study consisted of two stages (Fig. 1). In stage I (Exploration Stage), bone marrow samples were collected from 4 MM patients and 4 healthy controls (HCs), and plasma cells were isolated. Then circRNA expression profiles were detected by the microarray, and the bioinformatic analysis of circRNA microarray was performed. CircRNAs with at least 50% samples flagged as “Detected” were selected for further analysis, among which, circRNAs with ≥2.0 fold-changes (FC) and adjusted *P* values < 0.05 were defined as circRNAs with significant differential expression. In the stage II (Validation Stage), top 10 upregulated and top 10 downregulated circRNAs (based on rank of absolute value for log_2_FC) were selected from dysregulated circRNAs identified in the stage I, then were determined by quantitative polymerase chain reaction (qPCR) in the 60 MM patients (including the 4 MM patients in the stage I) and 30 HCs (including the 4 HCs in the stage I) for validation, and the diagnostic and prognostic value of these circRNAs in MM patients were further analyzed.

### Participants

Between October 2015 and September 2018, 60 de novo MM patients and 30 HCs were consecutively recruited from the Shanghai Jing’an District Zhabei Central Hospital. The inclusion criteria for the MM patients were: (1) newly diagnosed as MM according to International Myeloma Working Group (IMWG) updated criteria for the diagnosis of multiple myeloma (2014); (2) age more than 18 years; (3) life expectancy more than 12 months; (4) able to be regularly followed up. Following MM patients were excluded: (1) relapsed or secondary MM; (2) history of stem cell transplantation (SCT), chemotherapy, radiotherapy or other systematic treatments before enrollment; (3) accompanied with other malignancies; (4) serious infection (e.g. Human Immunodeficiency Virus); (5) pregnant women or lactating women. Besides, all 30 enrolled HCs were healthy bone marrow donors, whose health status was confirmed before donation by appropriate examinations. This study was approved by the Institutional Review Board of Shanghai Jing’an District Zhabei Central Hospital and was conducted according to the Ethical Guidelines for Human Genome/Gene Research issued by the Chinese Government. All participants provided written informed consents before enrollment.

### Collection of baseline data

Baseline data were collected after the patients signed the informed consents, including demographic information, such as age and gender, clinical characteristics and laboratory tests, such as immunoglobulin subtype, bone lesion, hemoglobin (Hb), calcium, serum creatinine (Scr), albumin (ALB), Beta-2-microglobulin (β2-MG), Durie-Salmon Stage, the International Staging System (ISS) Stage, lactate dehydrogenase (LDH) and cytogenetics abnormality. Durie-Salmon Stage and ISS Stage were evaluated in accordance with the Durie-Salmon Criteria and ISS Criteria respectively [12, 13], and cytogenetics abnormalities were determined by fluorescence in situ hybridization.

### Collection and processing of samples

For enrolled MM patients, bone marrow samples were extracted and collected before any treatment; as for the HCs, bone marrow samples were obtained on the enrollment. Immediately after collection of bone marrow samples, separation of mononuclear cells was performed with gradient density centrifugation, then plasma cells were purified using CD138-coated magnetic beads (Miltenyi Biotec, Germany), and all operations were carried out in strict accordance with the manufacturer’s instructions to ensure greater than 90% plasma cell purity.

### RNA extraction and quality control

Total RNAs were extracted from the plasma cells using the Trizol reagent (Invitrogen, USA), according to the manufacturer’s protocol, and RNA integrity was assessed using Agilent 2100 Bioanalyzer (Agilent, USA). Total RNA was quantified using NanoDrop ND-1000 spectrophotometer (Thermo, USA), then linear RNAs were diminished using RNase R (Epicentre, USA).

### Microarray detection of circRNAs

After removing linear RNAs, 4 samples from MM patients and 4 samples from HCs were amplified and transcribed into fluorescent cRNA utilizing a random priming method with a Super RNA Labeling Kit (Arraystar, USA), and the labeled cRNAs were purified using RNeasy Mini Kit (Qiagen, Germany). Then, the samples were hybridized with a CapitalBio Technology Human CircRNA Array v1 (Agilent, USA) and Hybridization Kit (Agilent, USA) following the manufacturer’s standard protocols in an Agilent Hybridization Oven (Agilent, USA). After hybridization, the hybridized arrays were washed, fixed and scanned using an Agilent Microarray Scanner (Agilent, USA). Scanned images were imported into Agilent Feature Extraction software (version 11.0.1.1) to obtain raw data. Quantile normalization and low-intensity filtering were carried out with the use of R software package (R version 3.1.2). The circRNAs with at least 50% of samples flagged as “Detected” were selected for further analysis.

### Bioinformatics analysis

Bioinformatics analysis was performed using R software package (R version 3.1.2). In brief, principal component analysis (PCA) of circRNA expression profiles was completed by Stats package; differentially expressed circRNAs between MM patients and HCs were analyzed with independent samples t-test using limma package, and circRNAs with a FC ≥2.0 and an adjusted *P* value (BH multiple test correction) < 0.05 were identified as differentially expressed circRNAs, which were displayed by volcano plots; heatmap plot of differentially expressed circRNAs were completed by pheatmap package. Gene Ontology (GO) enrichment analyses of dysregulated circRNAs were performed based on their located mRNAs and target miRNAs respectively; Kyoko Encyclopedia of Genes and Genomes (KEGG) enrichment analysis and pathway enrichment analysis of dysregulated circRNAs were performed based on their located mRNAs and predicted target miRNAs respectively. In order to investigate the regulation network between circRNAs and their target miRNAs, top 10 upregulated and top 10 downregulated circRNAs in MM sample were selected (based on rank of absolute value for log_2_FC) to plot the circRNA-miRNA network using miRanda Database.

### Validation of 20 candidate circRNAs by qPCR

A total of 60 MM patients’ samples and 30 HCs’ samples were used for qPCR validation. Top 10 upregulated and top 10 downregulated circRNAs were selected from differentially expressed circRNAs (identified in stage I) by the rank of the absolute value of Log_2_FC and were determined by the qPCR, which was performed briefly as follows: after removing linear RNA using RNase R (Epicentre, USA), RNA was reverse transcribed into cDNA with random primer using PrimeScript™ RT reagent Kit (Perfect Real Time) (Takara, Japan) according to the manufacturer’s instructions. Then qPCR was carried out using TB Green™ Fast qPCR Mix (Takara, Japan). The circRNAs relative expression was calculated using 2^-△△Ct^ method and normalized to GAPDH. All of the quantitative PCR reactions were conducted in triplicate. The primers used in qPCR were listed in Table 1. The expressions of top 10 upregulated and top 10 downregulated circRNAs detected by qPCR between 4 MM patients and 4 HCs from Stage I were shown in Additional file 1: Table S1.

**Table 1:** Characteristics of MM patients in Stage I and Stage II respectively

Characteristics	Stage I (*N* = 4)	Stage II (*N* = 60)
Age, years, mean (SD)	64.5 (4.5)	60.0 (9.4)
Gender (male/female), No.	2/2	37/23
Immunoglobulin subtype, No. (%)		
IgG	2 (50.0)	32 (53.3)
IgA	0 (0.0)	14 (23.4)
IgM	0 (0.0)	1 (1.7)
IgD	1 (25.0)	2 (3.3)
Bence-Jones protein	1 (25.0)	11 (18.3)
Bone lesion, No. (%)	3 (75.0)	42 (70.0)
Laboratory indexes, median (IQR)		
Hb (g/dL)	11.1 (8.7–13.5)	10.3 (9.0–11.8)
Calcium (mg/dL)	11.8 (8.9–12.1)	10.4 (9.1–11.7)
Scr (mg/dL)	1.6 (1.4–1.7)	1.6 (1.3–1.9)
ALB (mg/dL)	4.1 (3.7–4.5)	3.8 (3.2–4.5)
β2-MG (mg/L)	2.8 (1.2–4.2)	4.7 (2.8–9.0)
LDH (U/L)	170.6 (128.6–348.1)	183.1 (151.9–214.1)
Durie-Salmon stage, No. (%)		
I	0 (0.0)	2 (3.3)
II	2 (50.0)	32 (53.4)
III	2 (50.0)	26 (43.3)
ISS stage, No. (%)		
I	1 (25.0)	13 (21.7)
II	2 (50.0)	21 (35.0)
III	1 (25.0)	26 (43.3)
Cytogenetics, No. (%)		
t (4; 14)	0 (0.0)	6 (10.0)
t (14; 16)	0 (0.0)	7 (11.7)
Del (17p)	0 (0.0)	5 (8.3)

*MM* multiple myeloma, *SD* standard deviation, *Ig* immunoglobulin, *IQR* interquartile range, *Hb* hemoglobin, *Scr* serum creatinine, *ALB* albumin, *β2-MG* β2-microglobulin, *LDH* lactate dehydrogenase, *ISS* international staging system

### Treatment and follow up

All MM patients received appropriate treatments based on disease conditions according to IMWG Recommendations for the Treatment of Multiple Myeloma–Related Bone Disease, and the treatment responses were evaluated referring to the IMWG criteria as well. Response was classified as complete response (CR), very good partial response (VGPR), partial response (PR), and the overall response rate (ORR) was calculated as CR + VGPR+PR. All MM patients were routinely followed up to 2018/12/31 with the median follow-up duration of 24.0 months (range: 5.0–36.0 months). Besides, progression free survival (PFS) was calculated from the date of initiation treatment to the date of disease progression or death; Overall survival (OS) was calculated from the date of initiation treatment to the date of death.

### Statistical analysis

Data were displayed as mean and standard deviation (SD), median and interquartile range (IQR) or count (percentage). Comparisons were determined by the independent sample t test, Wilcoxon rank sum test or Chi-square test. Univariate and multivariate logistic regression analyses were performed to screen the circRNAs predicting MM risk. For the independent circRNAs in predicting MM risk, single and combined receiver operating characteristic (ROC) curves were plotted, and the area under the curve (AUC) was calculated to assess the diagnostic value of these circRNAs for MM. Survival profiles were displayed with Kaplan-Meier curves, and the difference in survival was determined by the log-rank test. All statistical analyses were performed using SPSS 24.0 Software (IBM, USA) or R software (Version 3.1.2), and graphs were plotted using GraphPad 7.01 Software (GraphPad, USA). *P* value < 0.05 was considered as significant.

## Results

### Baseline characteristics of MM patients in stage I and stage II respectively

In Stage I, 4 MM patients aged 64.5 ± 4.5 years with 2 male and 2 females were included for microarray assay (Table 2). The number of MM patients with Durie-Salmon stage I, II and III were 0 (0.0%), 2 (50.0%) and 2 (50.0%) respectively; and those in ISS stage I, II and III were 1 (25.0%), 2 (50.0%) and 1 (25.0%) respectively. In Stage II, 60 MM patients aged 60.0 ± 9.4 years with 37 males and 23 females were included for qPCR validation. There were 2 (3.3%), 32 (53.4%) and 26 (43.3%) patients in Durie-Salmon stage I, II and III respectively; and 13 (21.7%), 21 (35.0%) and 26 (43.3%) patients in ISS stage I, II and III respectively. See Table 2 for other detailed baseline information of MM patients in Stage I and Stage II.

**Table 2:** Top 10 upregulated and 10 downregulated circRNAs in MM patients compared to HCs

circRNA	Alias	Probe	Type	Chromosome	Start	End	Log_2_FC	*P* value	Adjusted *P* value	Gene Symbol	Trend
hsa_circRNA_104700	hsa_circ_0005273	ASCRP004952	exonic	chr8	141,710,989	141,716,304	3.409915	4.27E-05	0.002753	PTK2	UP
hsa_circRNA_102913	hsa_circ_0058058	ASCRP003221	exonic	chr2	216,177,220	216,190,861	2.939907	0.000842	0.01638	ATIC	UP
hsa_circRNA_104181	hsa_circ_0077765	ASCRP004454	exonic	chr6	125,366,356	125,398,004	2.927002	0.000841	0.01638	RNF217	UP
hsa_circRNA_100033	hsa_circ_0009581	ASCRP000423	exonic	chr1	8,555,122	8,601,377	2.343938	0.002566	0.031823	RERE	UP
hsa_circRNA_103276	hsa_circ_0064136	ASCRP003574	exonic	chr3	9,482,139	9,506,356	2.255827	2.83E-05	0.002231	SETD5	UP
hsa_circRNA_101695	hsa_circ_0007146	ASCRP002042	exonic	chr16	5,077,135	5,078,186	2.247917	0.000244	0.007711	NAGPA	UP
hsa_circRNA_104134	hsa_circ_0004136	ASCRP004407	exonic	chr6	73,713,630	73,751,785	2.232947	9.65E-05	0.004596	KCNQ5	UP
hsa_circRNA_104640	hsa_circ_0001806	ASCRP004893	exonic	chr8	68,018,139	68,028,357	2.221722	0.00036	0.009799	CSPP1	UP
hsa_circRNA_100542	hsa_circ_0017639	ASCRP000922	exonic	chr10	7,290,509	7,327,916	2.132697	6.32E-05	0.003752	SFMBT2	UP
hsa_circRNA_101287	hsa_circ_0008274	ASCRP001647	exonic	chr13	96,485,180	96,489,456	2.118815	0.004029	0.041781	UGGT2	UP
hsa_circRNA_105034	hsa_circ_0001947	ASCRP005281	exonic	chrX	147,743,428	147,744,289	−4.46399	3.17E-06	0.000578	AFF2	DOWN
hsa_circRNA_104980	hsa_circ_0001910	ASCRP005227	exonic	chrX	10,031,484	10,066,619	−4.12073	4.21E-07	0.000174	WWC3	DOWN
hsa_circRNA_101280	hsa_circ_0000497	ASCRP001641	exonic	chr13	78,293,666	78,327,493	−3.99021	2.15E-07	0.000126	SLAIN1	DOWN
hsa_circRNA_100526	hsa_circ_0004277	ASCRP000907	exonic	chr10	1,125,950	1,126,416	−3.95344	1.73E-07	0.000126	WDR37	DOWN
hsa_circRNA_100527	hsa_circ_0017446	ASCRP000908	exonic	chr10	1,125,950	1,132,297	−3.95022	2.21E-07	0.000126	WDR37	DOWN
hsa_circRNA_103106	hsa_circ_0007609	ASCRP003410	exonic	chr20	62,559,687	62,562,375	−3.8805	2.07E-07	0.000126	DNAJC5	DOWN
hsa_circRNA_103765	hsa_circ_0071375	ASCRP004055	exonic	chr4	166,141,085	166,184,511	−3.31029	5.36E-06	0.000749	KLHL2	DOWN
hsa_circRNA_101648	hsa_circ_0000651	ASCRP001997	exonic	chr15	90,982,563	90,986,710	−3.28088	3.21E-07	0.000163	IQGAP1	DOWN
hsa_circRNA_100731	hsa_circ_0020594	ASCRP001105	exonic	chr11	133,583	134,947	−3.25608	0.000108	0.004888	AL137655	DOWN
hsa_circRNA_104689	hsa_circ_0001824	ASCRP004941	exonic	chr8	131,164,981	131,181,313	−3.24653	2.21E-07	0.000126	ASAP1	DOWN

Top 10 upregulated and 10 downregulated circRNAs in MM patients compared to HCs were selected by the rank of absolute value of Log_2_FC. *MM* multiple myeloma, *HCs* health controls, *circRNA* circular RNA, *FC* fold change

### Identification of differentially expressed circRNAs in MM by microarray

**Fig. 2:**
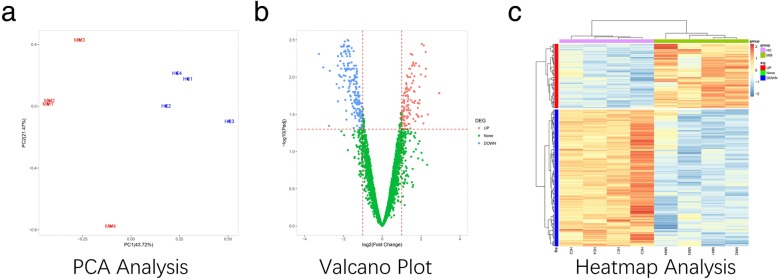
PCA, Valcano and Heatmap analyses of circRNA expression profiles. **a** MM patients and HCs were divided into two different groups by circRNAs. **b** 122 circRNAs were upregulated and 260 circRNAs were downregulated in MM compared with HCs. **c** The differentially expressed circRNAs were able to differentiate MM patients from HCs. MM, multiple myeloma; circRNAs, circular RNAs; HCs, healthy controls

PCA analysis showed clear segregation between 4 MM patients and 4 HCs, which indicated that circRNA expression patterns were able to distinguish MM patients from HCs (Fig. 2a). The valcano analysis was used to determine differentially expressed circRNAs between MM and HCs, which illustrated that 122 circRNAs were upregulated and 260 circRNAs were downregulated in MM compared with HCs (Fig. 2b). The threshold was set to fold change ≥2.0 and adjusted *P* value < 0.05. Following that, 122 upregulated and 260 downregulated circRNAs were included in heatmap analysis, and were shown to differentiate MM patients from HCs clearly (Fig. 2c).

### GO and KEGG enrichment analysis based on located genes and target miRNAs of dysregulated circRNAs in MM

**Fig. 3:**
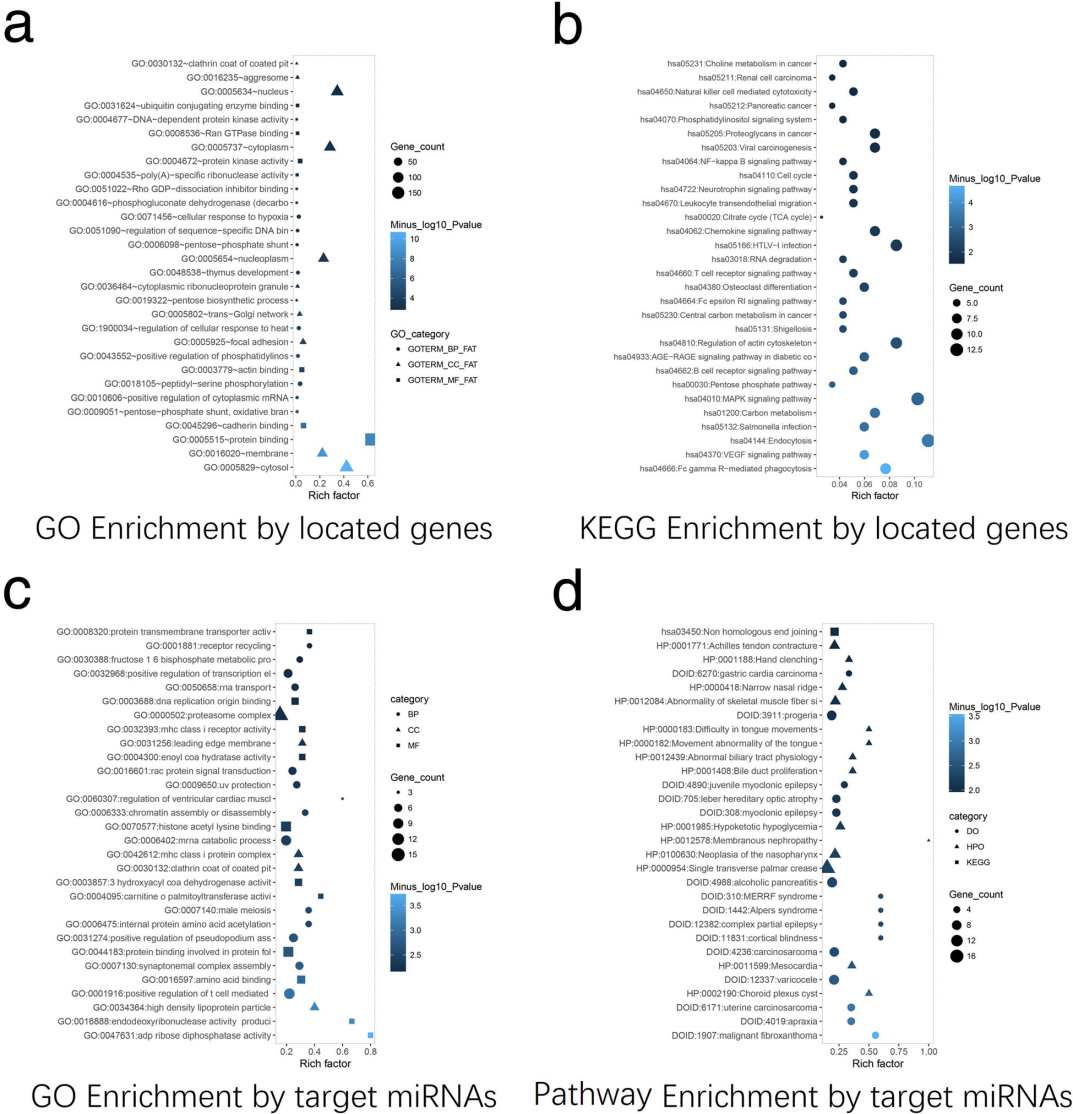
GO, KEGG and pathway enrichment analyses for circRNAs. The biological process, cellular component and molecular function that circRNAs were implicated in was revealed by GO enrichment analysis (**a**, **c**). In GO category, dot, triangle and square was used to symbolize BP, CC and MF respectively; the size of the symbols presented the gene counts; the gradation of color stood for the value of minus log10 adjusted *P* value. The cellular signaling pathways that circRNAs were implicated in were assessed by KEGG enrichment analysis (**b**) and pathway enrichment analysis (**d**). In KEGG by located genes, the size of the dot presented the gene counts; the gradation of color stood for the value of minus log10 P value. In pathway enrichment analysis by target miRNAs, category, dot, triangle and square was used to symbolize DO, HPO and KEGG respectively; the size of the symbols presented the gene counts; the gradation of color stood for the value of minus log10 P value. BP, biological process; CC, cellular component; MF, molecular function; DO, disease ontology; HPO, human phenotype ontology; KEGG, Kyoto Encyclopedia of Genes and Genomes; circRNAs, circular RNAs

GO enrichment analysis by located genes revealed that the located genes of dysregulated circRNAs in MM were enriched in biological processes (e.g. positive regulation of cytoplasmic mRNA and cellular response to hypoxia), cellular components (e.g. cytosol and membrane), molecular functions (e.g. protein binding and protein kinase activity) (Fig. 3a). And from KEGG enrichment analysis, the located genes of dysregulated circRNAs in MM were enriched in pathways such as VEGF signaling pathway and MAPK signaling pathway, which were well-known neoplastic pathways (Fig. 3b). According to GO enrichment analysis by target miRNAs, the target miRNAs of dysregulated circRNAs in MM were enriched in biological processes (e.g. positive regulation of t cell mediated cytotoxicity and synaptonemal complex assembly), cellular components (e.g. high density lipoprotein particle and MHC class I protein complex), and molecular functions (e.g. adp ribose diphosphatase activities and endodeoxynuclease activity produci) (Fig. 3c). Regarding the pathway enrichment analysis by target miRNAs, the target miRNAs of dysregulated circRNAs in MM were disclosed to be enriched in pathways that underline the ontology of various malignancies such as malignant fibroxanthoma and carcinosarcoma (Fig. 3d).

### Top 10 upregulated and top 10 downregulated circRNAs in MM patient compared with HCs by microarray and regulation network of these circRNAs

**Fig. 4:**
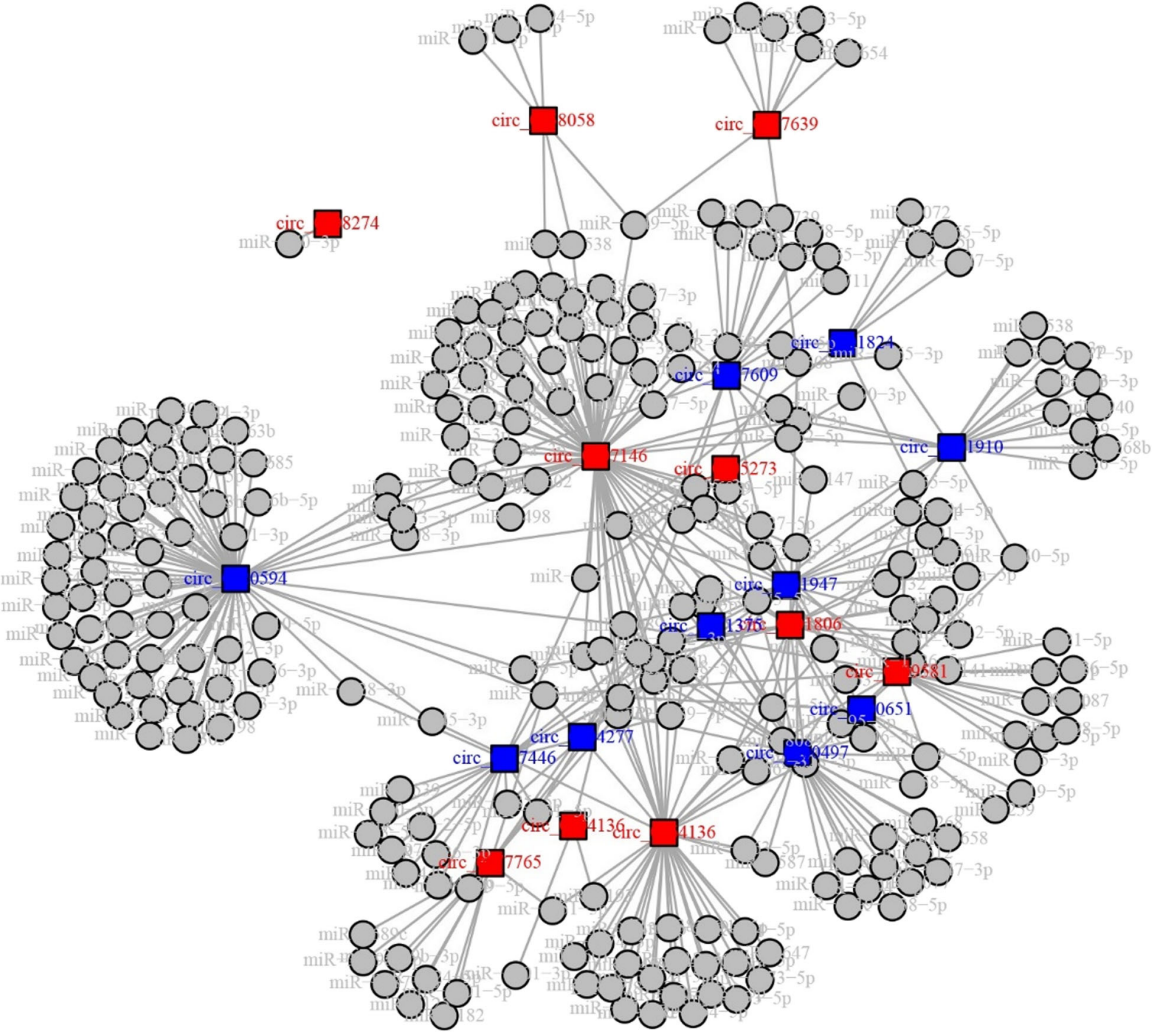
Regulation network of top 10 upregulated and top 10 downregulated circRNAs by microarray. The regulation network of circRNAs was as shown. The red squares represented the top 10 upregulated circRNAs; the blue squares represented the top 10 downregulated circRNAs, the gray dots represented the target miRNAs of circRNAs. The circRNA-miRNA network was plotted using miRanda Database. CircRNAs, circular RNAs; miRNA, micro RNA

Top 10 upregulated and top 10 downregulated circRNAs detected by microarray in MM patients compared to HCs were selected by the rank of the absolute value of Log_2_FC, and the detailed information of these circRNAs was listed in Table 3. Besides, the regulation network of these circRNAs with their target miRNAs was shown in Fig. 4.

**Table 3:** The primers used in qPCR

Genes	Species	Forward (5′- > 3′)	Reverse (5′- > 3′)
Circ-PTK2	Human	GCGTCTAATCCGACAGCAACA	AGAGATGCCTGACCTGGATAGA
Circ-ATIC	Human	GCCAGTTAGCCTTGAAGCCTTA	CAGGAAATCCCGTCAACTCAGA
Circ-RNF217	Human	AGTGCGAGGGTCAGTCTGT	ATGGCTTGGTGCTGGAATCA
Circ-RERE	Human	AACGACTGTGACCTCCTTATGT	TGTTCAGCCTCCTTGTCTCAG
Circ-SETD5	Human	CCACACCTGGCTCATCTCAC	CCCAGCCCTCAGTTGTATTCTC
Circ-NAGPA	Human	TTCACCAGCCAGGACAACAT	CCACAGTCCAGCTCATCACA
Circ-KCNQ5	Human	AGAGGATGGCAAGGAAGACTGA	ACTCCAGGATCAAGAGGCAACT
Circ-CSPP1	Human	CTGTCCCACCCATCCCATCA	CGTCTCTTGTTCCTCTGTTGCT
Circ-SFMBT2	Human	TCTCCTGCGTCGGTGACTAAG	CCACATAGCGAAGGCGTAATCT
Circ-UGGT2	Human	GGTGGAGTATGATGCTGAGATAAGA	AGAGACTTAATGGCGACTTGGTAA
Circ-AFF2	Human	CGGACATCTCACCAACACTGAA	AGCGTGTTCTGGACTCGGT
Circ-WWC3	Human	CTGCTCCGTTACCGACTCTC	TCTCGCCTCCACTGTTCTCT
Circ-SLAIN1	Human	GCTCCGAAGAAGTATGCCTAAC	GTCTCGCTGCTTCCATCTCA
Circ-WDR37–1	Human	AAGCCAGTCACAGCACCAG	TCCATCAATCGCTTGTCCTTCA
Circ-WDR37–2	Human	TTCCACCAGCAAGATTGTCTCC	GCTCCATCAATCGCTTGTCCTT
Circ-DNAJC5	Human	TGCTACTGCTGCTGCTGTC	CATCTGAGGTTGCGTTCTTGTC
Circ-KLHL2	Human	GCTTCACCCTGTCAACTGCTTA	TGCCAAGGATTCACTGTCACTG
Circ-IQGAP1	Human	AATCCGAATGCCATGCTTGTAA	GATGCCATACTTCTCCAACTCAG
Circ-AL137655	Human	AGGCTGGAGTGTAGTAGTGCTA	TCTGTAGAGGCTGACTGGAGAA
Circ-ASAP1	Human	AGTATGGCAGAGGAGGAAGTGT	AAGTCTCGGAGTGCAGTTAGC
GAPDH	Human	GGAGCGAGATCCCTCCAAAAT	GGCTGTTGTCATACTTCTCATGG

*qPCR* quantitative polymerase chain reaction

### Expression of candidate circRNAs between MM patients and HCs

**Fig. 5:**
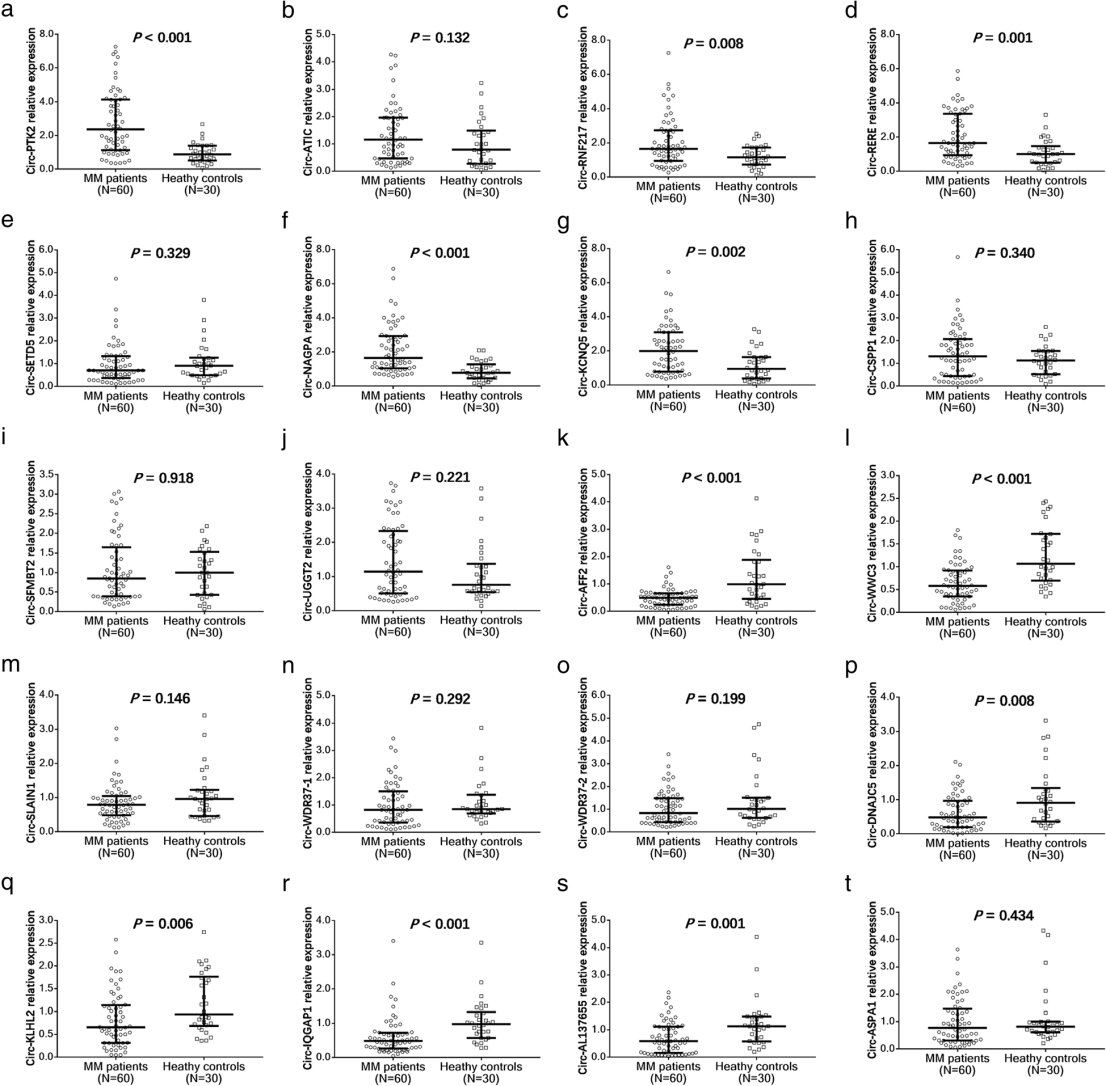
Comparing the levels of candidate circRNAs including Circ-PTK2 (**a**), circ-ATIC (**b**), circ-RNF217 (**c**), circ-RERE (**d**), circ-SETD5 (**e**), circ-NAGPA (**f**), circ-KCNQ5 (**g**), circ-CSPP1 (**h**), circ-SFMPT2 (**i**) and circ-UGGT2 (**j**), circ-AFF2 (**k**), circ-WWC3 (**l**), circ-SLAIN1 (**m**), circ-WDR37-1 (**n**), circ-WDR37-2 (**o**), circ-DNAJC5 (**p**), circ-KLHL2 (**q**), circ-IQGAP1 (**r**), circ-AL137655 (**s**) and circ-ASPA1 (**t**) in MM patients and HCs. Comparison of CircRNAs expressions between MM patients and HCs were determined by Wilcoxon rank sum test. *P* < 0.05 was considered significant. MM, multiple myeloma; circRNAs, circular RNAs

In validation stage, top 10 upregulated and top 10 downregulated circRNAs in MM patients compared to HCs were determined by the qPCR, and compared between MM patients (*N* = 60) and HCs (*N* = 30) for validation. Among the top 10 upregulated circRNAs, circ-PTK2 (*P* < 0.001) (Fig. 5a), circ-RNF217 (*P* = 0.008) (Fig. 5c), circ-RERE (*P* = 0.001) (Fig. 5d), circ-NAGPA (*P* < 0.001) (Fig. 5f) and circ-KCNQ5 (*P* = 0.002) (Fig. 5g) were validated to be upregulated, while circ-ATIC (*P* = 0.132) (Fig. 5b), circ-SETD5 (*P* = 0.329) (Fig. 5e), circ-CSPP1 (*P* = 0.340) (Fig. H), circ-SFMPT2 (*P* = 0.918) (Fig. 5i) and circ-UGGT2 (*P* = 0.221) (Fig. 5j) expression levels were similar in MM patients compared with HCs. As for the validation of the top 10 downregulated circRNAs, circ-AFF2 (*P* < 0.001) (Fig. 5f), circ-WWC3 (*P* < 0.001) (Fig. 5g), circ-DNAJC5 (*P* = 0.008) (Fig. 5p), circ-KLHL2 (*P* = 0.006) (Fig. 5q), circ-IQGAP1 (*P* < 0.001) (Fig. 5r) and circ-AL137655 (*P* = 0.001) (Fig. 5s) expressions were lower in MM patients compared with HCs, while circ-SLAIN1 (*P* = 0.146) (Fig. 5m), circ-WDR37–1 (*P* = 0.292) (Fig. 5n), circ-WDR37–2 (*P* = 0.199) (Fig. 5o) and circ-ASPA1 (*P* = 0.434) (Fig. 5t) levels were similar between MM patients and HCs.

### Correlation of candidate circRNAs with MM risk

**Fig. 6:**
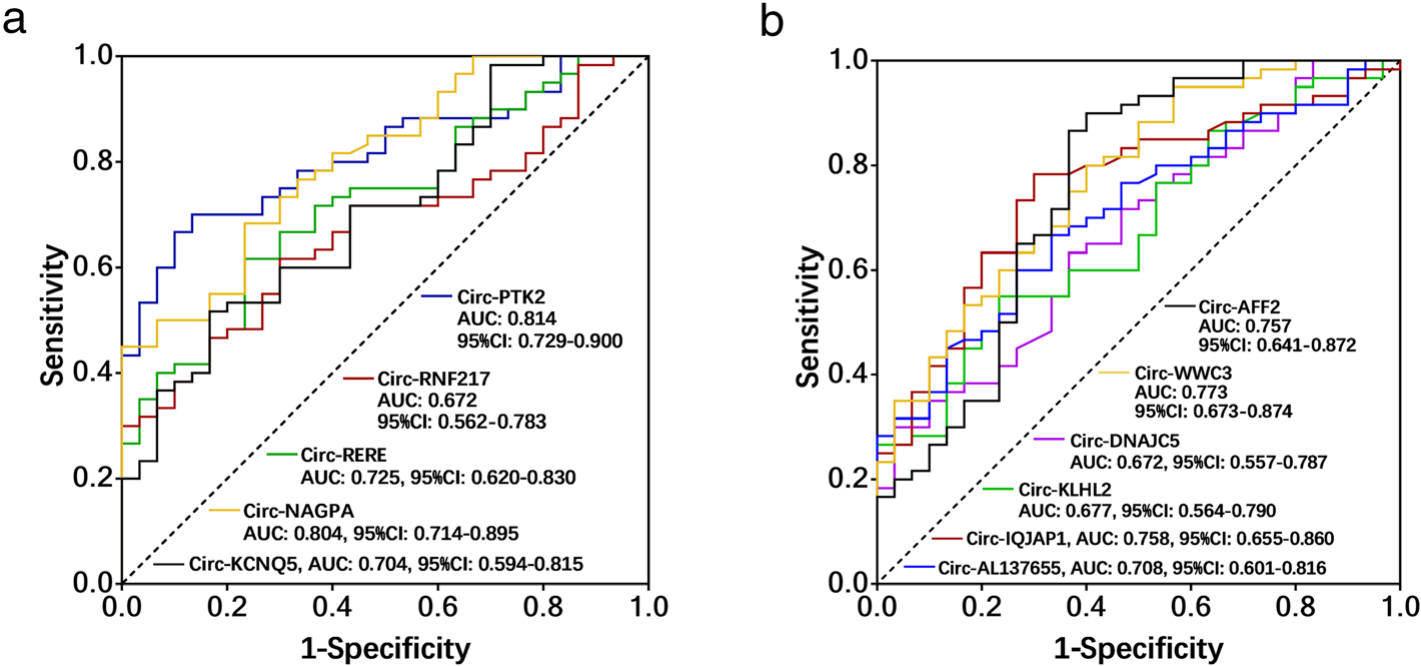
ROC curves of 5 upregulated (**a**) and 6 downregulated (**b**) candidate circRNAs in distinguishing MM patients from HCs. ROC curve, receiver operating characteristics curve; MM, multiple myeloma; circRNAs, circular RNAs; HCs, healthy controls

The 5 upregulated (Fig. 6a) and 6 downregulated (Fig. 6b) circRNAs were then included in the ROC analysis, which illuminated that circ-PTK2 (AUC: 0.814, 95% CI: 0.729–0.900), circ-RNF217 (AUC: 0.672, 95% CI: 0.562–0.783), circ-RERE (AUC: 0.725, 95% CI: 0.620–0.830), circ-NAGPA (AUC: 0.804, 95% CI: 0.714–0.895), circ-KCNQ5 (AUC: 0.704, 95% CI: 0.594–0.815) could predict higher MM risk. And circ-AFF2 (AUC: 0.757, 95% CI: 0.641–0.872), circ-WWC3 (AUC: 0.773, 95% CI: 0.673–0.874), circ-DNAJC5 (AUC: 0.672, 95% CI: 0.557–0.787), circ-KLHL2 (AUC: 0.677, 95% CI: 0.564–0.790), circ-IQJAP1 (AUC: 0.758, 95% CI: 0.655–0.860), circ-AL137655 (AUC: 0.708, 95% CI: 0.601–0.816) could predict lower MM risk.

### Correlation of candidate circRNAs with clinical characteristics in MM patients

Among the top 10 upregulated circRNAs, circ-PTK2 was correlated with higher β2-MG level (*P* = 0.002), advanced ISS stage (*P* = 0.002) and deletion of 17p (*P* = 0.020); circ-RERE was associated with higher calcium concentration (*P* = 0.024) and advanced Durie-Salmon stage (*P* = 0.037); circ-SETD5 was positively correlated with deletion in 17p (*P* = 0.024); circ-KCNQ5 was positively correlated with ALB level (*P* = 0.012); circ-UGGT2 was negatively correlated with Durie-Salmon stage (*P* = 0.037) and positively correlated with deletion in 17p (*P* = 0.020) (Table 4). As for the top 10 downregulated circRNAs, circ-AFF2 correlated with lower β2-MG level (*P* = 0.002) and decreased ISS stage (*P* = 0.002); circ-WWC3 was associated with lower Durie-Salmon stage (*P* = 0.037); circ-WDR37–2 was negatively correlated with deletion in 17p (*P* = 0.016); circ-DNAJC5 was positively correlated with age (*P* = 0.039) and negatively correlated with LDH level (*P* = 0.028); circ-KLHL2 was negatively correlated with ALB level (*P* = 0.012); circ-IQGAP1 was correlated with abundance of IgA (*P* = 0.038) (Table 4). No correlation of candidate circRNAs with other clinical characteristics was observed, and the detailed information was listed in Table 4.

**Table 4:** Correlation of candidate circRNAs relative expression with clinical characteristics

Items	CircRNAs									
	PTK2	ATIC	RNF217	RERE	SETD5	NAGPA	KCNQ5	CSPP1	SFMBT2	UGGT2
	High	High	High	High	High	High	High	High	High	High
Age, No. (%)										
< 60 years	16 (53.3)	15 (50.0)	14 (46.7)	14 (46.7)	14 (46.7)	13 (43.3)	16 (53.3)	16 (53.3)	16 (53.3)	18 (60.0)
≥ 60 years	14 (46.7)	15 (50.0)	16 (53.3)	16 (53.3)	17 (56.7)	17 (56.7)	14 (46.7)	14 (46.7)	14 (46.7)	12 (40.0)
*P* value	0.606	1.000	0.606	0.606	0.438	0.302	0.606	0.606	0.606	0.121
Gender, No. (%)										
Male	17 (45.9)	13 (56.5)	9 (39.1)	11 (47.8)	14 (60.9)	20 (54.1)	19 (51.4)	20 (54.1)	19 (51.4)	18 (48.6)
Female	13 (56.5)	17 (45.9)	21 (56.8)	19 (51.4)	17 (45.9)	10 (43.5)	11 (47.8)	10 (43.5)	11 (47.8)	12 (52.2)
*P* value	0.426	0.426	0.184	0.791	0.261	0.426	0.791	0.426	0.791	0.791
Immunoglobulin subtype, No. (%)										
IgG	13 (40.6)	15 (46.9)	15 (46.9)	16 (50.0)	18 (56.3)	18 (56.3)	17 (53.1)	19 (59.4)	12 (37.5)	13 (40.6)
IgA	7 (50.0)	7 (50.0)	9 (64.3)	4 (28.6)	7 (50.0)	8 (57.1)	4 (28.6)	5 (35.7)	8 (57.1)	9 (64.3)
IgM	1 (100.0)	1 (100.0)	1 (100.0)	1 (100.0)	0 (0.0)	0 (0.0)	1 (100.0)	0 (0.0)	0 (0.0)	1 (100.0)
IgD	2 (100.0)	1 (50.0)	1 (50.0)	2 (100.0)	1 (50.0)	1 (50.0)	1 (50.0)	2 (100.0)	2 (100.0)	0 (0.0)
Bence-Jones protein	7 (63.6)	6 (54.5)	4 (36.4)	7 (63.6)	5 (45.5)	3 (27.3)	7 (63.6)	4 (36.4)	8 (72.7)	7 (63.6)
*P* value	0.293	0.875	0.544	0.172	0.822	0.398	0.341	0.193	0.109	0.193
Bone lesion, No. (%)										
No	9 (50.0)	8 (44.4)	11 (61.1)	7 (38.9)	10 (55.6)	10 (55.6)	10 (55.6)	11 (61.1)	7 (38.9)	7 (38.9)
Yes	21 (50.0)	22 (52.4)	19 (45.2)	23 (54.8)	21 (50.0)	20 (47.6)	20 (47.6)	19 (45.2)	23 (54.8)	23 (54.8)
*P* value	1.000	0.573	0.260	0.260	0.693	0.573	0.573	0.260	0.260	0.260
Hb, No. (%)										
< 10 g/dL	14 (51.9)	12 (44.4)	14 (51.9)	14 (51.9)	16 (59.3)	14 (51.9)	15 (55.6)	17 (63.0)	13 (48.1)	11 (40.7)
≥ 10 g/dL	16 (48.5)	18 (54.5)	16 (48.5)	16 (48.5)	15 (45.5)	16 (48.5)	15 (45.5)	13 (39.4)	17 (51.5)	19 (57.6)
*P* value	0.795	0.436	0.795	0.795	0.287	0.795	0.436	0.069	0.795	0.194
Calcium, No. (%)										
< 11.5 mg/dL	21 (50.0)	22 (52.4)	18 (42.9)	17 (40.5)	21 (50.0)	19 (45.2)	20 (47.6)	20 (47.6)	20 (47.6)	22 (52.4)
≥ 11.5 mg/dL	9 (50.0)	8 (44.4)	12 (66.7)	13 (72.2)	10 (55.6)	11 (61.1)	10 (55.6)	10 (55.6)	10 (55.6)	8 (44.4)
*P* value	1.000	0.573	0.091	**0.024**	0.693	0.260	0.573	0.573	0.573	0.573
Scr, No. (%)										
< 2 mg/dL	25 (52.1)	26 (54.2)	23 (47.9)	22 (45.8)	26 (54.2)	26 (54.2)	24 (50.0)	26 (54.2)	23 (47.9)	25 (52.1)
≥ 2 mg/dL	5 (41.7)	4 (33.3)	7 (58.3)	8 (66.7)	5 (41.7)	4 (33.3)	6 (50.0)	4 (33.3)	7 (58.3)	5 (41.7)
*P* value	0.519	0.197	0.519	0.197	0.438	0.197	1.000	0.197	0.519	0.519
ALB, No. (%)										
< 3.5 mg/dL	10 (52.6)	12 (63.2)	7 (36.8)	10 (52.6)	11 (57.9)	12 (63.2)	5 (26.3)	6 (31.6)	8 (42.1)	8 (42.1)
≥ 3.5 mg/dL	20 (48.8)	18 (43.9)	23 (56.1)	20 (48.8)	20 (48.8)	18 (43.9)	25 (61.0)	24 (58.5)	22 (53.7)	22 (53.7)
*P* value	0.781	0.165	0.165	0.781	0.511	0.165	**0.012**	0.052	0.405	0.405
β2-MG, No. (%)										
< 5.5 mg/L	11 (32.4)	16 (47.1)	16 (47.1)	16 (47.1)	17 (50.0)	20 (58.8)	15 (44.1)	17 (50.0)	15 (44.1)	16 (47.1)
≥ 5.5 mg/L	19 (73.1)	14 (53.8)	14 (53.8)	14 (53.8)	14 (53.8)	10 (38.5)	15 (57.7)	13 (50.0)	15 (57.7)	14 (53.8)
*P* value	**0.002**	0.602	0.602	0.602	0.768	0.118	0.297	1.000	0.297	0.602
LDH, No. (%)										
< 220 U/L	23 (48.9)	21 (44.7)	25 (53.2)	25 (53.2)	24 (51.1)	25 (53.2)	25 (53.2)	25 (53.2)	22 (46.8)	23 (48.9)
≥ 220 U/L	7 (53.8)	9 (69.2)	5 (38.5)	5 (38.5)	7 (53.8)	5 (38.5)	5 (38.5)	5 (38.5)	8 (61.5)	7 (53.8)
*P* value	0.754	0.117	0.347	0.347	0.859	0.347	0.347	0.347	0.347	0.754
Durie-Salmon stage, No. (%)										
I/II	16 (47.1)	17 (50.0)	15 (44.1)	13 (38.2)	15 (44.1)	16 (47.1)	14 (41.2)	16 (47.1)	18 (52.9)	21 (61.8)
III	14 (53.8)	13 (50.0)	15 (57.7)	17 (65.4)	16 (61.5)	14 (53.8)	16 (61.5)	14 (53.8)	12 (46.2)	9 (24.6)
*P* value	0.602	1.000	0.297	**0.037**	0.181	0.602	0.118	0.602	0.602	**0.037**
ISS stage, No. (%)										
I/II	11 (32.4)	16 (47.1)	16 (47.1)	16 (47.1)	17 (50.0)	20 (58.8)	15 (44.1)	17 (50.0)	15 (44.1)	16 (47.1)
III	19 (73.1)	14 (53.8)	14 (53.8)	14 (53.8)	14 (53.8)	10 (38.5)	15 (57.7)	13 (50.0)	15 (57.7)	14 (53.8)
*P* value	**0.002**	0.602	0.602	0.602	0.768	0.118	0.297	1.000	0.297	0.602
t (4; 14), No. (%)										
No	25 (46.3)	27 (50.0)	27 (50.0)	26 (48.1)	28 (51.9)	28 (51.9)	26 (48.1)	28 (51.9)	27 (50.0)	27 (50.0)
Yes	5 (83.3)	3 (50.0)	3 (50.0)	4 (66.7)	3 (50.0)	2 (33.3)	4 (66.7)	2 (33.3)	3 (50.0)	3 (50.0)
*P* value	0.085	1.000	1.000	0.389	0.931	0.389	0.389	0.389	1.000	1.000
t (14; 16), No. (%)										
No	27 (50.9)	26 (49.1)	27 (50.9)	28 (52.8)	27 (50.9)	26 (49.1)	27 (50.9)	27 (50.9)	27 (50.9)	28 (52.8)
Yes	3 (42.9)	4 (57.1)	3 (42.9)	2 (28.6)	4 (57.1)	4 (57.1)	3 (42.9)	3 (42.9)	3 (42.9)	2 (28.6)
*P* value	0.688	0.688	0.688	0.228	0.758	0.688	0.688	0.688	0.688	0.228
Del (17p), No. (%)										
No	25 (45.5)	28 (50.9)	27 (49.1)	28 (50.9)	26 (47.3)	27 (49.1)	26 (47.3)	29 (52.7)	27 (49.1)	30 (54.5)
Yes	5 (100.0)	2 (40.0)	3 (60.0)	2 (40.0)	5 (100.0)	3 (60.0)	4 (80.0)	1 (20.0)	3 (60.0)	0 (0.0)
*P* value	**0.020**	0.640	0.640	0.640	**0.024**	0.640	0.161	0.161	0.640	**0.020**
Items					CircRNAs					
	AFF2	WWC3	SLAIN1	WDR37–1	WDR37–2	DNAJC5	KLHL2	IQGAP1	AL137655	ASAP1
	High	High	High	High	High	High	High	High	High	High
Age, No. (%)										
< 60 years	12 (40.0)	15 (50.0)	13 (43.3)	17 (56.7)	15 (50.0)	11 (36.7)	16 (53.3)	15 (50.0)	12 (40.0)	12 (40.0)
≥ 60 years	18 (60.0)	15 (50.0)	17 (56.7)	13 (43.3)	16 (53.3)	19 (63.3)	14 (46.7)	15 (50.0)	18 (60.0)	18 (60.0)
*P* value	0.121	1.000	0.302	0.302	0.796	**0.039**	0.606	1.000	0.121	0.121
Gender, No. (%)										
Male	11 (47.8)	18 (48.6)	12 (52.2)	13 (56.5)	10 (43.5)	13 (56.5)	19 (51.4)	20 (54.1)	19 (51.4)	21 (56.8)
Female	19 (51.4)	12 (52.2)	18 (48.6)	17 (45.9)	21 (56.8)	17 (45.9)	11 (47.8)	10 (43.5)	11 (47.8)	9 (39.1)
*P* value	0.791	0.791	0.791	0.426	0.317	0.426	0.791	0.426	0.791	0.184
Immunoglobulin subtype, No. (%)										
IgG	17 (53.1)	15 (46.9)	13 (40.6)	12 (37.5)	18 (56.3)	18 (56.3)	15 (46.9)	17 (53.1)	17 (53.1)	16 (50.0)
IgA	8 (57.1)	7 (50.0)	10 (70.4)	9 (64.3)	7 (50.0)	5 (35.7)	9 (64.3)	10 (71.4)	7 (50.0)	6 (42.9)
IgM	1 (100.0)	1 (100.0)	0 (0.0)	1 (100.0)	1 (100.0)	0 (0.0)	0 (0.0)	1 (100.0)	1 (100.0)	1 (100.0)
IgD	2 (100.0)	1 (50.0)	1 (50.0)	1 (50.0)	1 (50.0)	1 (50.0)	1 (50.0)	0 (0.0)	1 (50.0)	2 (100.0)
Bence-Jones protein	11 (100.0)	6 (54.5)	6 (54.5)	7 (63.6)	4 (36.4)	6 (54.5)	5 (45.5)	2 (18.2)	4 (36.4)	5 (45.5)
*P* value	0.451	0.875	0.310	0.291	0.689	0.603	0.670	**0.038**	0.746	0.497
Bone lesion, No. (%)										
No	8 (44.4)	9 (50.0)	9 (50.0)	8 (44.4)	7 (38.9)	10 (55.6)	11 (61.1)	11 (61.1)	8 (44.4)	11 (61.1)
Yes	22 (52.4)	21 (50.0)	21 (50.0)	22 (52.4)	24 (57.1)	20 (47.6)	19 (45.2)	19 (45.2)	22 (52.4)	19 (45.2)
*P* value	0.573	1.000	1.000	0.573	0.195	0.573	0.260	0.260	0.573	0.260
Hb, No. (%)										
< 10 g/dL	12 (44.4)	12 (44.4)	14 (51.9)	14 (51.9)	14(51.9)	11 (40.7)	14 (51.9)	13 (48.1)	11 (40.7)	14 (51.9)
≥ 10 g/dL	18 (54.5)	18 (54.5)	16 (48.5)	16 (48.5)	17 (51.5)	19 (57.6)	16 (48.5)	17 (51.5)	19 (57.6)	16 (48.5)
*P* value	0.436	0.436	0.795	0.795	0.979	0.194	0.795	0.795	0.194	0.795
Calcium, No. (%)										
< 11.5 mg/dL	22 (52.4)	23 (54.8)	21 (50.0)	22 (52.4)	21 (50.0)	19 (45.2)	21 (50.0)	21 (50.0)	19 (45.2)	21 (50.0)
≥ 11.5 mg/dL	8 (44.4)	7 (38.9)	9 (50.0)	8 (44.4)	10 (55.6)	11 (61.1)	9 (50.0)	9 (50.0)	11 (61.1)	9 (50.0)
*P* value	0.573	0.260	1.000	0.573	0.693	0.260	1.000	1.000	0.260	1.000
Scr, No. (%)										
< 2 mg/dL	26 (54.2)	22 (45.8)	25 (52.1)	23 (47.9)	23 (47.9)	26 (54.2)	25 (52.1)	23 (47.9)	26 (54.2)	26 (54.2)
≥ 2 mg/dL	4 (33.3)	8 (66.7)	5 (41.7)	7 (58.3)	8 (66.7)	4 (33.3)	5 (41.7)	7 (58.3)	4 (33.3)	4 (33.3)
*P* value	0.197	0.197	0.519	0.519	0.245	0.197	0.519	0.519	0.197	0.197
ALB, No. (%)										
< 3.5 mg/dL	11 (57.9)	10 (52.6)	9 (47.4)	9 (47.4)	9 (47.4)	8 (42.1)	14 (73.7)	12 (63.2)	7 (36.8)	12 (63.2)
≥ 3.5 mg/dL	19 (46.3)	20 (48.8)	21 (51.2)	21 (51.2)	22 (53.7)	22 (53.7)	16 (39.0)	18 (43.9)	23 (56.1)	18 (43.9)
*P* value	0.405	0.781	0.781	0.781	0.650	0.405	**0.012**	0.165	0.165	0.165
β2-MG, No. (%)										
< 5.5 mg/L	23 (67.6)	16 (47.1)	19 (55.9)	20 (58.8)	20 (58.8)	16 (47.1)	18 (52.9)	16 (47.1)	19 (55.9)	20 (58.8)
≥ 5.5 mg/L	7 (26.9)	14 (53.8)	11 (42.3)	10 (38.5)	11 (42.3)	14 (53.8)	12 (46.2)	14 (53.8)	11 (42.3)	10 (38.5)
*P* value	**0.002**	0.602	0.297	0.118	0.205	0.602	0.602	0.602	0.297	0.118
LDH, No. (%)										
< 220 U/L	24 (51.1)	24 (51.1)	24 (51.1)	23 (48.9)	26 (55.3)	27 (57.4)	21 (44.7)	22 (46.8)	24 (51.1)	25 (53.2)
≥ 220 U/L	6 (46.2)	6 (46.2)	6 (46.2)	7 (53.8)	5 (38.5)	3 (23.1)	9 (69.2)	8 (61.5)	6 (46.2)	5 (38.5)
*P* value	0.754	0.754	0.754	0.754	0.282	**0.028**	0.117	0.347	0.754	0.347
Durie-Salmon stage, No. (%)										
I/II	19 (55.9)	21 (61.8)	18 (52.9)	19 (55.9)	17 (50.0)	14 (41.2)	16 (47.1)	19 (55.9)	17 (50.0)	14 (41.2)
III	11 (42.3)	9 (34.6)	12 (46.2)	11 (42.3)	14 (53.8)	16 (61.5)	14 (53.8)	11 (42.3)	13 (50.0)	16 (61.5)
*P* value	0.297	**0.037**	0.602	0.297	0.768	0.118	0.602	0.297	1.000	0.118
ISS stage, No. (%)										
I/II	23 (67.6)	16 (47.1)	19 (55.9)	20 (58.8)	20 (58.8)	16 (47.1)	18 (52.9)	16 (47.1)	19 (55.9)	20 (58.8)
III	7 (26.9)	14 (53.8)	11 (42.3)	10 (38.5)	11 (42.3)	14 (53.8)	12 (46.2)	14 (53.8)	11 (42.3)	10 (38.5)
*P* value	**0.002**	0.602	0.297	0.118	0.205	0.602	0.602	0.602	0.297	0.118
t (4; 14), No. (%)										
No	29 (53.7)	25 (46.3)	25 (46.3)	28 (51.9)	27 (50.0)	26 (48.1)	27 (50.0)	27 (50.0)	27 (50.0)	26 (48.1)
Yes	1 (16.7)	5 (83.3)	5 (83.3)	2 (33.3)	4 (66.7)	4 (66.7)	3 (50.0)	3 (50.0)	3 (50.0)	4 (66.7)
*P* value	0.085	0.085	0.085	0.389	0.438	0.389	1.000	1.000	1.000	0.389
t (14; 16), No. (%)										
No	28 (52.8)	28 (52.8)	27 (50.9)	28 (52.8)	28 (52.8)	27 (50.9)	25 (47.2)	26 (49.1)	28 (52.8)	26 (49.1)
Yes	2 (28.6)	2 (28.6)	3 (42.9)	2 (28.6)	3 (42.9)	3 (42.9)	5 (71.4)	4 (57.1)	2 (28.6)	4 (57.1)
*P* value	0.228	0.228	0.688	0.228	0.620	0.688	0.228	0.688	0.228	0.688
Del (17p), No. (%)										
No	29 (52.7)	29 (52.7)	28 (50.9)	28 (50.9)	31 (56.4)	27 (49.1)	26 (47.3)	27 (49.1)	27 (49.1)	28 (50.9)
Yes	1 (20.0)	1 (20.0)	2 (40.0)	2 (40.0)	0 (0.0)	3 (60.0)	4 (80.0)	3 (60.0)	3 (60.0)	2 (40.0)
*P* value	0.161	0.161	0.640	0.640	**0.016**	0.640	0.161	0.640	0.640	0.640

Comparisons were determined by Chi-square test. *P* value < 0.05 was considered significant. *circRNAs* circular RNAs, *Ig* immunoglobulin, *Hb* hemoglobin, *Scr* serum creatinine, *ALB* albumin, *β2-MG* β2-microglobulin, *LDH* lactate dehydrogenase, *ISS* international staging system. The number in boldface represented statistically significant *P* values

### Correlation of candidate circRNAs with treatment response in MM patients

The correlation of candidate circRNAs with treatment response in MM patients was assessed and we observed that, in the top 10 upregulated circRNAs, circ-PTK2 (*P* = 0.015) was associated with reduced CR; circ-RNF217 (*P* = 0.020) and circ-SETD5 (*P* = 0.029) were correlated with lower ORR (Table 5). As for the top 10 downregulated circRNAs, circ-AFF2 (*P* = 0.002) was positively correlated with CR. No correlation of other candidate circRNAs with treatment response was observed.

**Table 5:** Correlation of circRNAs relative expression with treatment response

circRNAs	CR	Non-CR	*P* value	ORR	Non-ORR	*P* value
Circ-PTK2, No. (%)			**0.015**			0.559
High	3 (10.0)	27 (90.0)		21 (70.0)	9 (30.0)	
Low	11 (36.7)	19 (63.3)		23 (76.7)	7 (23.3)	
Circ-ATIC, No. (%)			0.222			0.559
High	9 (30.0)	21 (70.0)		21 (70.0)	9 (30.0)	
Low	5 (16.7)	25 (83.3)		23 (76.7)	7 (23.3)	
Circ-RNF217, No. (%)			0.222			**0.020**
High	5 (16.7)	25 (83.3)		18 (60.0)	12 (40.0)	
Low	9 (30.0)	21 (70.0)		26 (86.7)	4 (13.3)	
Circ-RERE, No. (%)			1.000			0.559
High	7 (23.3)	23 (76.7)		21 (70.0)	9 (30.0)	
Low	7 (23.3)	23 (76.7)		23 (76.7)	7 (23.3)	
Circ-SETD5, No. (%)			0.173			**0.029**
High	5 (16.1)	26 (83.9)		19 (61.3)	12 (38.7)	
Low	9 (31.0)	20 (69.0)		25 (86.2)	4 (13.8)	
Circ-NAGPA, No. (%)			1.000			0.559
High	7 (23.3)	23 (76.7)		21 (70.0)	9 (30.0)	
Low	7 (23.3)	23 (76.7)		23 (76.7)	7 (23.3)	
Circ-KCNQ5, No. (%)			0.222			0.243
High	5 (16.7)	25 (83.3)		24 (80.0)	6 (20.0)	
Low	9 (30.0)	21 (70.0)		20 (66.7)	10 (33.3)	
Circ-CSPP1, No. (%)			0.067			0.559
High	10 (33.3)	20 (66.7)		23 (76.7)	7 (23.3)	
Low	4 (13.3)	26 (86.7)		21 (70.0)	9 (30.))	
Circ-SFMBT2, No. (%)			0.067			1.000
High	10 (33.3)	20 (66.7)		22 (73.3)	8 (26.7)	
Low	4 (13.3)	26 (86.7)		22 (73.3)	8 (26.7)	
Circ-UGGT2, No. (%)			0.542			0.243
High	8 (26.7)	22 (73.3)		24 (80.0)	6 (20.0)	
Low	6 (20.0)	24 (80.0)		20 (66.7)	10 (33.3)	
Circ-AFF2, No. (%)			**0.002**			0.559
High	12 (40.0)	18 (60.0)		23 (76.7)	7 (23.3)	
Low	2 (6.7)	28 (93.3)		21 (70.0)	9 (30.0)	
Circ-WWC3, No. (%)			0.222			0.080
High	5 (16.7)	25 (83.3)		19 (63.3)	11 (36.7)	
Low	9 (30.0)	21 (70.0)		25 (83.3)	5 (16.7)	
Circ-SLAIN1, No. (%)			0.222			0.559
High	5 (16.7)	25 (83.3)		21 (70.0)	9 (30.0)	
Low	9 (30.0)	21 (70.0)		23 (76.7)	7 (23.3)	
Circ-WDR37–1, No. (%)			0.542			0.559
High	6 (20.0)	24 (80.0)		23 (76.7)	7 (23.3)	
Low	8 (26.7)	22 (73.3)		21 (70.0)	9 (30.0)	
Circ-WDR37–2, No. (%)			0.640			0.185
High	8 (25.8)	23 (74.2)		25 (80.6)	6 (19.4)	
Low	6 (20.7)	23 (79.3)		19 (65.5)	10 (34.5)	
Circ-DNAJC5, No. (%)			0.222			0.243
High	5 (16.7)	25 (83.3)		20 (66.7)	10 (33.3)	
Low	9 (30.0)	12 (70.0)		24 (80.0)	6 (20.0)	
Circ-KLHL2, No. (%)			0.542			0.559
High	6 (20.0)	24 (80.0)		23 (76.7)	7 (23.3)	
Low	8 (26.7)	22 (73.3)		21 (70.0)	9 (30.0)	
Circ-IQGAP1, No. (%)			0.542			0.243
High	6 (20.0)	24 (80.0)		24 (80.0)	6 (20.0)	
Low	8 (26.7)	22 (73.3)		20 (66.7)	10 (33.3)	
Circ-AL137655, No. (%)			1.000			0.080
High	7 (23.3)	23 (76.7)		25 (83.3)	5 (16.7)	
Low	7 (23.3)	23 (76.7)		19 (63.3)	11 (36.7)	
Circ-ASAP1, No. (%)			0.542			0.559
High	6 (20.0)	24 (80.0)		21 (70.0)	9 (30.0)	
Low	8 (26.7)	22 (73.3)		23 (76.7)	7 (23.3)	

Comparisons were determined by Chi-square test. *P* value < 0.05 was considered significant. *CR* complete response, *ORR* overall response rate. The number in boldface represented statistically significant *P* values

### Correlation of candidate circRNAs with survival profiles in MM patients

**Fig. 7:**
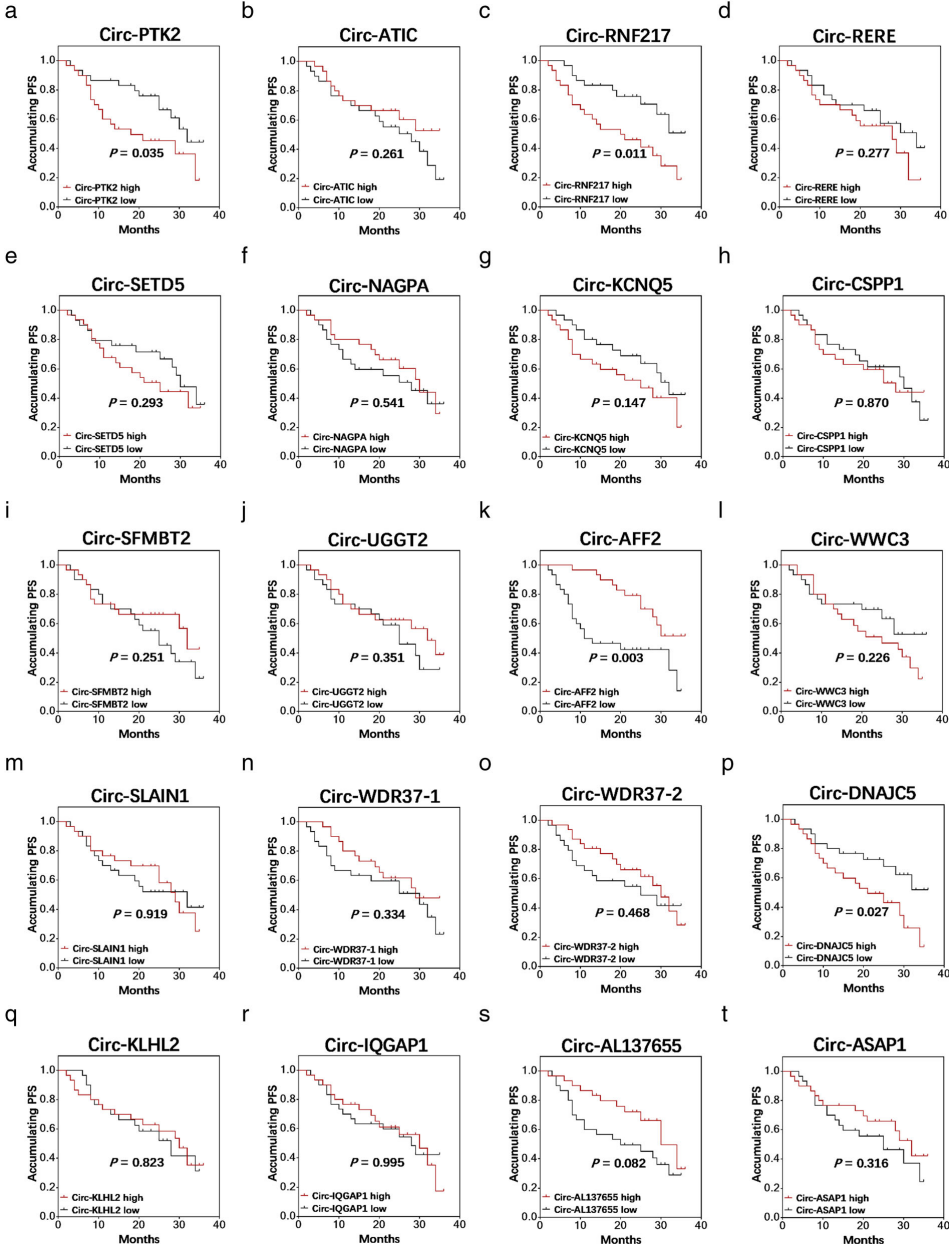
Correlation of candidate circRNAs including Circ-PTK2 (**a**), circ-ATIC (**b**), circ-RNF217 (**c**), circ-RERE (**d**), circ-SETD5 (**e**), circ-NAGPA (**f**), circ-KCNQ5 (**g**), circ-CSPP1 (**h**), circ-SFMPT2 (**i**), circ-UGGT2 (**j**), circ-AFF2 (**k**), circ-WWC3 (**l**), circ-SLAIN1 (**m**), circ-WDR37-1 (**n**), circ-WDR37-2 (**o**), circ-DNAJC5 (**p**), circ-KLHL2 (**q**), circ-IQGAP1 (**r**), circ-AL137655 (**s**) and circ-ASPA1 (**t**) with PFS in MM patients. PFS was displayed with Kaplan-Meier curves, and the difference in survival was determined by the log-rank test. *P* < 0.05 was considered significant. PFS, progression free survival; MM, multiple myeloma; circRNAs, circular RNAs

Circ-PTK2 (*P* = 0.035) (Fig. 7a), circ-RNF217 (*P* = 0.011) (Fig. 7c) and circ-DNAJC5 (*P* = 0.027) (Fig. 7p) were correlated with lower PFS, but circ-AFF2 (*P* = 0.003) (Fig. 7k) predicted longer PFS. Whereas the other candidate circRNAs, including circ-ATIC (*P* = 0.261) (Fig. 7b), circ-RERE (*P* = 0.277) (Fig. 7d), circ-SETD5 (*P* = 0.293) (Fig. 7e), circ-NAGPA (*P* = 0.541) (Fig. 7f), circ-KCNQ5 (*P* = 0.147) (Fig. 7g), circ-CSPP1 (*P* = 0.870) **(**Fig. 7h), circ-SFMBT2 (*P* = 0.251) (Fig. 7i), circ-UGGT2 (*P* = 0.351) (Fig. 7j), circ-WWC3 (*P* = 0.226) (Fig. 7l), circ-SLAIN1 (*P* = 0.919) (Fig. 7m), circ-WDR37–1 (*P* = 0.334) (Fig. 7n), circ-WDR37–2 (*P* = 0.468) (Fig. 7o), circ-KLHL2 (*P* = 0.823) (Fig. 7q), circ-IQGAP1 (*P* = 0.995) (Fig. 7r), circ-AL137655 (*P* = 0.082) (Fig. 7s) and circ-ASAP1 (*P* = 0.316) (Fig. 7t) were not correlated with PFS in MM patients.

**Fig. 8:**
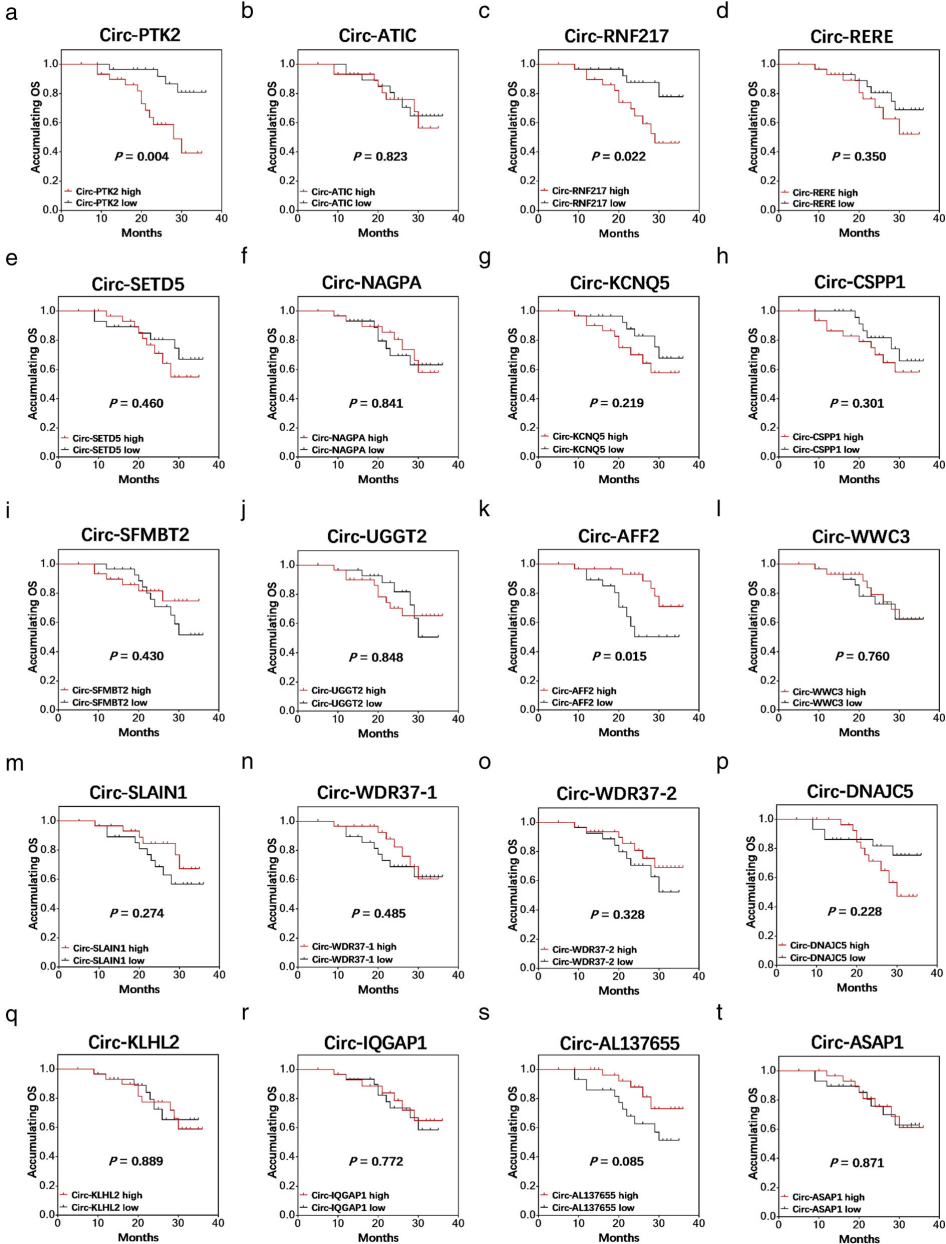
Correlation of candidate circRNAs including Circ-PTK2 (**a**), circ-ATIC (**b**), circ-RNF217 (**c**), circ-RERE (**d**), circ-SETD5 (**e**), circ-NAGPA (**f**), circ-KCNQ5 (**g**), circ-CSPP1 (**h**), circ-SFMPT2 (**i**), circ-UGGT2 (**j**), circ-AFF2 (**k**), circ-WWC3 (**l**), circ-SLAIN1 (**m**), circ-WDR37-1 (**n**), circ-WDR37-2 (**o**), circ-DNAJC5 (**p**), circ-KLHL2 (**q**), circ-IQGAP1 (**r**), circ-AL137655 (**s**) and circ-ASPA1 (**t**) with OS in MM patients. OS was displayed with Kaplan-Meier curves, and the difference in survival was determined by the log-rank test. P < 0.05 was considered significant. OS, overall survival; MM, multiple myeloma; circRNAs, circular RNAs

Regarding OS, circ-PTK2 (*P* = 0.004) (Fig. 8a) and circ-RNF217 (*P* = 0.022) (Fig. 8c) were associated with lower OS, but circ-AFF2 (*P* = 0.015) (Fig. 8k) was associated with longer PFS. Other candidate circRNAs including circ-ATIC (*P* = 0.823) (Fig. 8b), circ-RERE (*P* = 0.350) (Fig. 8d), circ-SETD5 (*P* = 0.460) (Fig. 8e), circ-NAGPA (*P* = 0.841) (Fig. 8f) circ-KCNQ5 (*P* = 0.219) (Fig. 8g), circ-CSPP1 (*P* = 0.301) (Fig. 8h), circ-SFMBT2 (*P* = 0.430) (Fig. 8i), circ-UGGT2 (*P* = 0.848) (Fig. 8j), circ-WWC3 (*P* = 0.760) (Fig. 8l), circ-SLAIN1 (*P* = 0.274) (Fig. 8m), circ-WDR37–1 (*P* = 0.485) (Fig. 8n), circ-WDR37–2 (*P* = 0.328) (Fig. 8o), circ-DNAJC5 (*P* = 0.228) **(**Fig. 8p), circ-KLHL2 (*P* = 0.889) (Fig. 8q), circ-IQGAP1 (*P* = 0.772) (Fig. 8r), circ-AL137655 (*P* = 0.085) (Fig. 8s) and circ-ASAP1 (*P* = 0.871) (Fig. 8t) were not correlated with OS in MM patients.

## Discussion

From this comprehensive analysis of circRNA expression profiles in MM, (1) we found that circRNA expression patterns were able to distinguish MM patients from HCs, and there were 122 upregulated and 260 downregulated circRNAs in MM compared with HCs, which were implicated in neoplastic signaling pathways such as MAPK signaling pathways and VEGF signaling pathway. (2) In validation stage, 5 out of 10 upregulated and 6 out of 10 downregulated circRNAs by microarray were confirmed by qPCR, and these circRNAs could distinguish MM patients from healthy controls. (3) Circ-PTK2 and circ-RNF217 were correlated with poor treatment response and survival, while circ-AFF2 predicted favorable treatment response and survival in MM patients.

With the rapid progression and wide application of high-throughput sequencing and microarray, the expression pattern of circRNAs in various human diseases are increasingly reported, and the dysregulated expressions of circRNAs are shown to contribute to the pathogenesis of various cancers [7, 9–11, 14–19]. For instance, in hepatitis B-related HCC, 189 upregulated circRNAs and 37 downregulated circRNAs were found by circRNA microarray, and circRNA_100,338 is further validated to be associated with metastatic progression by acting as an endogenous sponge for miR-141-3p [7]. Another study identifies 2556 upregulated and 1832 downregulated circRNAs in epithelial ovarian cancer tissues compared with normal ovarian tissues by microarray and bioinformatic analysis [11]. As for hematological malignancies, one previous study reveals the expression patterns of circRNAs in AML and exhibits 147 upregulated and 317 downregulated circRNAs in AML patients compared with healthy controls [10]. These previous studies uncover the expression patterns of circRNAs in several cancers including hematological malignancy, however, there is currently no study on heterogenicity of circRNA expression profiles in MM yet. In our study, we performed circRNA microarray and identified 122 upregulated and 260 downregulated circRNAs in bone marrow plasma cells of MM patients compared with HCs. In addition, these dysregulated circRNAs were shown to be involved in neoplastic signaling pathways including MAPK signaling pathway and VEGF signaling pathway. To our knowledge, this was the first study that investigates the expression patterns of circRNAs in MM, which might serve as valuable reference for further investigation of circRNAs functions in MM.

Benefiting from the stable nature and RNA degradation resistance, circRNAs are considered as prominent and novel biomarkers for many diseases, especially cancer, and there are specific circRNAs whose clinical values have been highlighted in several cancers. For instance, circ-LDLRAD3 is upregulated in pancreatic cancer, and is disclosed to be a potential biomarker in disease diagnosis [20]. In addition, circ_0014130 is positively correlated with TNM stage as well as lymphatic metastasis, and is of good diagnostic potential for NSCLC [21]. As for hematological malignancies, circ_0004277 is downregulated and offers a diagnostic biomarker in AML [10]. These aforementioned studies emphasized the potential of several specific circRNAs as biomarkers in diagnosis of solid tumors and hematological malignancies to a certain extent, however, the diagnostic value of circRNAs in MM is still misty. In addition, circRNA expression profile is a novel concept developed in recent years, and the current comprehensive screening of circRNA expression such as microarray is still limited by the accuracy. In addition, the sample size for microarray was far smaller than that of q-PCR, therefore, it was highly possible that the two analyses yielded deviation in results. CircRNA expression profiles by microarray aimed to give us a macroscopic view about the expression patterns of circRNAs, however, a larger sample size and more accurate tool were needed for a more refined understanding. Therefore, we carried out the Stage II analysis using q-PCR in a larger sample size. We selected the top 10 upregulated and top 10 downregulated circRNAs from the previous bioinformatic analyses, and validated regarding their diagnostic potential in MM with a larger sample size by qPCR. Our analyses revealed that, 5 out of 10 upregulated and 6 out of 10 downregulated circRNAs by microarray were confirmed by qPCR, and these circRNAs could distinguish MM patients from healthy controls. The possible explanations could be: (1) These circRNAs might influence the transcription of their parental genes by acting as restoration pools. For instance, circ-PTK2 serves as restoration pool for its paternal gene PTK2, which was identified as oncogene in MM, and increases the expression of PTK2 gene, thereby increases MM risk [22]. (2) These circRNAs might influence the pathogenesis of MM by sponging their target miRNAs. For example, circ-AFF2 might sponge miR-638 and inhibit the oncogenic function of miR-638 in MM (as shown in circRNA regulation network (Fig. 4)). Additionally, circ-PTK2 might act as sponge for anti-oncogenic miR-1298-5p and promote the neoplastic progression in MM (retrieved from tissue specific circRNA database: http://gb.whu.edu.cn/TSCD/). Although further studies were needed to analyze and demonstrate the detailed mechanisms of these circRNAs in MM, our study still illuminated that circ-PTK2, circ-RERE, circ-AFF2 and circ-WWC3 could serve as novel diagnostic biomarkers in MM.

Although rarely shown, it is still evident from the existing studies that some specific circRNAs are closely correlated with treatment response and may have potential prognostic value in cancer patients. For example, circ_0000285 expression is lower in cisplatin-resistant bladder cancer patients compared to cisplatin-sensitive patients and is independently correlated with poor treatment outcomes in bladder cancer patients [23]. Regarding survival, circ-RAD23B, an oncogene in NSCLC, predicts shorter OS in NSCLC patients [24]. Additionally, circRNA expression profiles display that circ_0001017 and circ_0061276 are correlated with longer OS in gastric cancer patients [25]. As for hematological malignancies, only one study exhibits that circ_100053 contributes to leukemogenesis in chronic myeloid leukemia (CML) and predicts increased resistance to imatinib as well as poor survival in CML patients [26]. Although the correlation of several specific circRNAs with patients’ prognosis in solid tumors as well as hematological malignancy has been reported, the correlation of circRNAs with prognosis in MM is still unknown [27]. In order to get a more profound understanding of the correlation of circRNAs with prognosis in MM, we evaluated the correlation of the top 10 upregulated and top 10 downregulated circRNAs with treatment response as well as survival, and disclosed that circ-PTK2 and circ-RNF217 were correlated with poor treatment response and survival, while circ-AFF2 predicted good treatment response and survival in MM patients. The possible reasons were: (1) According to our analyses, these circRNAs were closely correlated with clinicopathological features in MM patients, therefore, they would affect the prognosis of MM patients via influencing the clinicopathological features such as Durie-Salmon stage and deletion at 17p. (2) These circRNAs might change the cell sensitivity to chemotherapy and develop drug resistance via targeting miRNAs, thereby influence prognosis in cancer patients. For instance, circ-AFF2 might sponge miR-638, which was previously shown to induce drug resistance in human breast cancer, thereby reduced drug resistance and improved prognosis in MM patients [28]. (3) As explained above, these circRNAs might impact the normal function of miRNAs by serving as miRNA sponges in MM (see Fig. 4 for regulation network of candidate circRNAs and the detailed miRNAs), thereby influenced prognosis in MM patients. In addition, the tubular form of potential miRNA targets of the top 10 upregulated and top 10 downregulated circRNAs was shown in Additional file 2: Table S2, and the potential target miRNAs of all the 122 upregulated and 260 downregulated circRNAs were listed in Additional file 3.

This study first revealed the differential expressions of circRNAs and determined circRNAs with diagnostic and prognostic potential in MM, whereas there were still some shortcomings. Firstly, although several circRNAs with potential as diagnostic and prognostic biomarkers for MM were identified, the molecular mechanisms of these circRNAs in MM pathology were not investigated. Secondly, due to the budget, the sample size was relatively small for stage I, and MM patients from different clinical stage were not included for analysis, which could be improved in further studies. Thirdly, we explored the prognostic value of circRNAs but not in a logical approach because it was not the main goal in this study. However, it would be of great clinical significance to further detect the correlation of these circRNAs with prognosis in MM patients in a more logical way in the future. Moreover, the use of circRNAs as biomarkers for cancers is still in the early stage of research, and thorough practical proofs and standards were needed for clinical application. Studies that further validate the feasibility of circRNAs as diagnostic and prognostic biomarkers in cancer are needed to lead the bench side findings to real-life application.

## Conclusion

In conclusion, this study provides valuable reference for profound understanding about expression patterns of circRNAs in MM, and validates that circ-PTK2, circ-RNF217 and circ-AFF2 might serve as potential prognostic biomarkers in MM.

## Supplementary information

**Additional file 1: Table S1.** CircRNAs expression by qPCR in the 4 MM patients and 4 HCs involved in the Stage I

**Additional file 2: Table S2.** miRNA targets of the top 10 upregulated and top 10 downregulated circRNAs.

**Additional file 3.** The potential target miRNAs of all the upregulated and downregulated ircRNAs.

## Data Availability

The datasets used and/or analysed during the current study are available from the corresponding author on reasonable request.

## Abbreviations

ALB: Albumin
AML: Acute myeloid leukemia
AUC: Area under the curve
circRNAs: Circular RNAs
CR: Complete response
FC: Fold-changes
GO: Gene ontology
Hb: Hemoglobin
HCs: Healthy controls
IMWG: International Myeloma Working Group
IQR: Interquartile range
ISS: International staging system
KEGG: Kyoko Encyclopedia of Genes and Genomes
LDH: Lactate dehydrogenase
miRNAs: microRNAs
MM: Multiple myeloma
ORR: Overall response rate
OS: Overall survival
PCA: Principal component analysis
PFS: Progression free survival
PR: Partial response
qPCR: Quantitative polymerase chain reaction
ROC: Receiver operating characteristic
Scr: Serum creatinine
SCT: Stem cell transplantation
SD: Standard deviation
VGPR: Very good partial response
β2-MG: Beta-2-microglobulin
