# Supplementary material for: Comprehensive profiling of circular RNA expressions reveals potential diagnostic and prognostic biomarkers in multiple myeloma
Source: BMC Cancer. 2020 Jan 16;20:40. doi: 10.1186/s12885-020-6515-2 (PMC6966810; doi:10.1186/s12885-020-6515-2)
Supplement: Supplementary file 1 — Additional file 1: Table S1. CircRNAs expression by qPCR in the 4 MM patients and 4 HCs involved in the Stage I [file 12885_2020_6515_MOESM1_ESM.docx]

**Table S1.**

CircRNAs expression by qPCR in the 4 MM patients and 4 HCs involved in the Stage I

| Genes | CircRNAs relative expression, median (IQR) | | *P* value |
| --- | --- | --- | --- |
|  | MM patients (N=4) | HCs (N=4) |  |
| Circ-PTK2 | 1.122 (0.523-2.996) | 0.204 (0.099-0.282) | 0.021 |
| Circ-ATIC | 2.350 (1.254-4.034) | 0.479 (0.091-0.798) | 0.021 |
| Circ-RNF217 | 1.351 (0.502-2.649) | 0.287 (0.134-0.407) | 0.021 |
| Circ-RERE | 0.923 (0.720-3.031) | 0.165 (0.082-0.182) | 0.021 |
| Circ-SETD5 | 0.693 (0.215-1.603) | 0.284 (0.180-0.414) | 0.386 |
| Circ-NAGPA | 1.227 (0.987-3.165) | 0.718 (0.279-0.867) | 0.021 |
| Circ-KCNQ5 | 3.217 (1.318-5.809) | 0.360 (0.273-0.533) | 0.021 |
| Circ-CSPP1 | 1.508 (1.257-2.747) | 0.630 (0.219-1.555) | 0.149 |
| Circ-SFMBT2 | 1.259 (0.966-1.594) | 0.554 (0.435-0.910) | 0.043 |
| Circ-UGGT2 | 1.691 (0.472-2.823) | 0.495 (0.377-0.641) | 0.248 |
| Circ-AFF2 | 0.523 (0.220-0.821) | 2.438 (1.882-2.813) | 0.021 |
| Circ-WWC3 | 0.412 (0.400-0.620) | 1.905 (1.180-2.347) | 0.021 |
| Circ-SLAIN1 | 0.783 (0.612-1.485) | 1.705 (0.944-3.090) | 0.149 |
| Circ-WDR37-1 | 0.888 (0.374-1.503) | 1.603 (1.088-3.317) | 0.083 |
| Circ-WDR37-2 | 1.539 (0.460-2.534) | 3.985 (3.241-4.702) | 0.021 |
| Circ-DNAJC5 | 0.460 (0.238-1.242) | 2.836 (2.556-3.206) | 0.021 |
| Circ-KLHL2 | 1.091 (0.355-1.808) | 1.835 (0.839-2.014) | 0.386 |
| Circ-IQGAP1 | 0.324 (0.207-0.517) | 1.304 (1.028-2.023) | 0.021 |
| Circ-AL137655 | 0.893 (0.651-1.126) | 1.507 (1.217-2.810) | 0.021 |
| Circ-ASAP1 | 0.610 (0.130-2.742) | 2.640 (1.833-3.915) | 0.149 |

Comparison was determined by the Wilcoxon rank sum test. *P* value <0.05 was considered significant. MM: multiple myeloma; HCs: healthy controls; IQR: interquartile range.
