# Supplementary material for: Comprehensive profiling of circular RNA expressions reveals potential diagnostic and prognostic biomarkers in multiple myeloma
Source: BMC Cancer. 2020 Jan 16;20:40. doi: 10.1186/s12885-020-6515-2 (PMC6966810; doi:10.1186/s12885-020-6515-2)
Supplement: Supplementary file 2 — Additional file 2: Table S2. miRNA targets of the top 10 upregulated and top 10 downregulated circRNAs. [file 12885_2020_6515_MOESM2_ESM.docx]

**Table S2.**

miRNA targets of the top 10 upregulated and top 10 downregulated circRNAs.

| CircRNA.ID | MiRNA.ID |
| --- | --- |
| hsa_circ_0000497 | hsa-miR-141-5p |
|  | hsa-miR-296-3p |
|  | hsa-miR-328-5p |
|  | hsa-miR-612 |
|  | hsa-miR-637 |
|  | hsa-miR-762 |
|  | hsa-miR-939-5p |
|  | hsa-miR-1238-5p |
|  | hsa-miR-1207-5p |
|  | hsa-miR-1292-5p |
|  | hsa-miR-4268 |
|  | hsa-miR-3620-5p |
|  | hsa-miR-1587 |
|  | hsa-miR-4658 |
|  | hsa-miR-4674 |
|  | hsa-miR-4745-5p |
|  | hsa-miR-4758-5p |
|  | hsa-miR-4783-3p |
|  | hsa-miR-6089 |
|  | hsa-miR-6719-3p |
|  | hsa-miR-6756-5p |
|  | hsa-miR-6763-5p |
|  | hsa-miR-6775-5p |
|  | hsa-miR-6794-5p |
|  | hsa-miR-6795-5p |
|  | hsa-miR-6803-5p |
|  | hsa-miR-6815-3p |
|  | hsa-miR-6846-5p |
|  | hsa-miR-6848-5p |
|  | hsa-miR-6861-5p |
|  | hsa-miR-7847-3p |
|  | hsa-miR-8077 |
|  | hsa-miR-8089 |
| hsa_circ_0000651 | hsa-miR-1226-5p |
|  | hsa-miR-4259 |
|  | hsa-miR-4758-5p |
|  | hsa-miR-6727-5p |
|  | hsa-miR-6779-5p |
|  | hsa-miR-6819-5p |
| hsa_circ_0001806 | hsa-miR-328-5p |
|  | hsa-miR-608 |
|  | hsa-miR-638 |
|  | hsa-miR-1229-5p |
|  | hsa-miR-3132 |
|  | hsa-miR-3141 |
|  | hsa-miR-3147 |
|  | hsa-miR-3151-5p |
|  | hsa-miR-4763-3p |
|  | hsa-miR-4767 |
|  | hsa-miR-6722-3p |
|  | hsa-miR-6756-5p |
|  | hsa-miR-6775-5p |
|  | hsa-miR-6786-5p |
|  | hsa-miR-6795-5p |
|  | hsa-miR-6812-5p |
|  | hsa-miR-6846-5p |
|  | hsa-miR-6879-5p |
|  | hsa-miR-6887-5p |
|  | hsa-miR-8089 |
| hsa_circ_0001824 | hsa-miR-638 |
|  | hsa-miR-4655-5p |
|  | hsa-miR-4685-5p |
|  | hsa-miR-6747-5p |
|  | hsa-miR-6765-5p |
|  | hsa-miR-6865-5p |
|  | hsa-miR-8072 |
| hsa_circ_0001910 | hsa-miR-210-3p |
|  | hsa-miR-503-3p |
|  | hsa-miR-940 |
|  | hsa-miR-1247-5p |
|  | hsa-miR-1538 |
|  | hsa-miR-4292 |
|  | hsa-miR-1268b |
|  | hsa-miR-4640-5p |
|  | hsa-miR-4665-3p |
|  | hsa-miR-4691-5p |
|  | hsa-miR-4741 |
|  | hsa-miR-4783-3p |
|  | hsa-miR-6726-5p |
|  | hsa-miR-6762-5p |
|  | hsa-miR-6770-3p |
|  | hsa-miR-6775-5p |
|  | hsa-miR-6780a-5p |
|  | hsa-miR-6869-5p |
|  | hsa-miR-6882-3p |
| hsa_circ_0001947 | hsa-miR-34a-5p |
|  | hsa-miR-637 |
|  | hsa-miR-638 |
|  | hsa-miR-661 |
|  | hsa-miR-1226-5p |
|  | hsa-miR-1915-3p |
|  | hsa-miR-3191-3p |
|  | hsa-miR-4298 |
|  | hsa-miR-3620-5p |
|  | hsa-miR-4707-5p |
|  | hsa-miR-4763-3p |
|  | hsa-miR-6089 |
|  | hsa-miR-6727-5p |
|  | hsa-miR-6789-5p |
|  | hsa-miR-6791-5p |
|  | hsa-miR-6803-5p |
|  | hsa-miR-6855-5p |
|  | hsa-miR-6889-5p |
|  | hsa-miR-6894-5p |
| hsa_circ_0004136 | hsa-miR-1193 |
|  | hsa-miR-6089 |
|  | hsa-miR-6501-3p |
| hsa_circ_0004277 | hsa-miR-3620-5p |
|  | hsa-miR-4763-3p |
|  | hsa-miR-6731-5p |
|  | hsa-miR-6751-5p |
|  | hsa-miR-6779-5p |
|  | hsa-miR-6785-5p |
|  | hsa-miR-6803-5p |
| hsa_circ_0005273 | hsa-miR-638 |
|  | hsa-miR-4690-3p |
|  | hsa-miR-6724-5p |
|  | hsa-miR-6749-5p |
|  | hsa-miR-6775-5p |
| hsa_circ_0007146 | hsa-miR-139-3p |
|  | hsa-miR-210-3p |
|  | hsa-miR-296-3p |
|  | hsa-miR-423-3p |
|  | hsa-miR-602 |
|  | hsa-miR-638 |
|  | hsa-miR-762 |
|  | hsa-miR-874-3p |
|  | hsa-miR-541-5p |
|  | hsa-miR-1225-3p |
|  | hsa-miR-1226-3p |
|  | hsa-miR-1237-5p |
|  | hsa-miR-1202 |
|  | hsa-miR-1291 |
|  | hsa-miR-1292-5p |
|  | hsa-miR-1469 |
|  | hsa-miR-1908-3p |
|  | hsa-miR-1914-3p |
|  | hsa-miR-1915-3p |
|  | hsa-miR-2277-5p |
|  | hsa-miR-718 |
|  | hsa-miR-2861 |
|  | hsa-miR-3177-5p |
|  | hsa-miR-3180-3p |
|  | hsa-miR-3187-5p |
|  | hsa-miR-3187-3p |
|  | hsa-miR-3196 |
|  | hsa-miR-3620-5p |
|  | hsa-miR-4498 |
|  | hsa-miR-4538 |
|  | hsa-miR-3972 |
|  | hsa-miR-4632-5p |
|  | hsa-miR-4649-5p |
|  | hsa-miR-4656 |
|  | hsa-miR-4663 |
|  | hsa-miR-4685-5p |
|  | hsa-miR-1343-5p |
|  | hsa-miR-4690-5p |
|  | hsa-miR-4707-5p |
|  | hsa-miR-4715-3p |
|  | hsa-miR-4731-5p |
|  | hsa-miR-4734 |
|  | hsa-miR-4741 |
|  | hsa-miR-4763-3p |
|  | hsa-miR-4783-3p |
|  | hsa-miR-4787-3p |
|  | hsa-miR-5189-5p |
|  | hsa-miR-6089 |
|  | hsa-miR-6722-3p |
|  | hsa-miR-6724-5p |
|  | hsa-miR-6727-5p |
|  | hsa-miR-6762-5p |
|  | hsa-miR-6769a-3p |
|  | hsa-miR-6775-5p |
|  | hsa-miR-6779-5p |
|  | hsa-miR-6784-5p |
|  | hsa-miR-6787-5p |
|  | hsa-miR-6791-5p |
|  | hsa-miR-6803-5p |
|  | hsa-miR-6806-5p |
|  | hsa-miR-6825-3p |
|  | hsa-miR-6848-5p |
|  | hsa-miR-6858-3p |
|  | hsa-miR-6861-5p |
|  | hsa-miR-6872-3p |
|  | hsa-miR-6885-5p |
|  | hsa-miR-6889-5p |
|  | hsa-miR-6893-3p |
|  | hsa-miR-7112-5p |
|  | hsa-miR-7113-3p |
|  | hsa-miR-7706 |
| hsa_circ_0007609 | hsa-miR-214-3p |
|  | hsa-miR-412-5p |
|  | hsa-miR-608 |
|  | hsa-miR-874-3p |
|  | hsa-miR-711 |
|  | hsa-miR-3147 |
|  | hsa-miR-4665-5p |
|  | hsa-miR-4665-3p |
|  | hsa-miR-4706 |
|  | hsa-miR-4734 |
|  | hsa-miR-4739 |
|  | hsa-miR-4741 |
|  | hsa-miR-5008-3p |
|  | hsa-miR-6089 |
|  | hsa-miR-6727-5p |
|  | hsa-miR-6749-5p |
|  | hsa-miR-6778-5p |
|  | hsa-miR-6793-5p |
|  | hsa-miR-6803-5p |
|  | hsa-miR-7113-5p |
| hsa_circ_0008274 | hsa-miR-370-3p |
| hsa_circ_0009581 | hsa-miR-328-5p |
|  | hsa-miR-345-3p |
|  | hsa-miR-939-5p |
|  | hsa-miR-1226-5p |
|  | hsa-miR-4298 |
|  | hsa-miR-4632-5p |
|  | hsa-miR-4640-5p |
|  | hsa-miR-4726-5p |
|  | hsa-miR-6087 |
|  | hsa-miR-6089 |
|  | hsa-miR-6729-5p |
|  | hsa-miR-6751-5p |
|  | hsa-miR-6752-5p |
|  | hsa-miR-6760-5p |
|  | hsa-miR-6765-5p |
|  | hsa-miR-6798-5p |
|  | hsa-miR-6821-5p |
|  | hsa-miR-6880-5p |
|  | hsa-miR-8089 |
| hsa_circ_0017446 | hsa-miR-143-5p |
|  | hsa-miR-128-2-5p |
|  | hsa-miR-770-5p |
|  | hsa-miR-1539 |
|  | hsa-miR-3620-5p |
|  | hsa-miR-3944-3p |
|  | hsa-miR-4449 |
|  | hsa-miR-4726-3p |
|  | hsa-miR-4728-3p |
|  | hsa-miR-4763-3p |
|  | hsa-miR-1199-5p |
|  | hsa-miR-6724-5p |
|  | hsa-miR-6731-5p |
|  | hsa-miR-6751-5p |
|  | hsa-miR-6779-5p |
|  | hsa-miR-6785-5p |
|  | hsa-miR-6789-5p |
|  | hsa-miR-6803-5p |
|  | hsa-miR-8075 |
| hsa_circ_0017639 | hsa-miR-608 |
|  | hsa-miR-1299 |
|  | hsa-miR-4649-5p |
|  | hsa-miR-4654 |
|  | hsa-miR-4763-5p |
|  | hsa-miR-6789-3p |
|  | hsa-miR-6796-5p |
|  | hsa-miR-7843-5p |
| hsa_circ_0020594 | hsa-miR-198 |
|  | hsa-miR-328-3p |
|  | hsa-miR-483-3p |
|  | hsa-miR-484 |
|  | hsa-miR-615-3p |
|  | hsa-miR-638 |
|  | hsa-miR-1224-3p |
|  | hsa-miR-1296-5p |
|  | hsa-miR-877-3p |
|  | hsa-miR-937-5p |
|  | hsa-miR-1229-3p |
|  | hsa-miR-1234-3p |
|  | hsa-miR-1236-3p |
|  | hsa-miR-1237-3p |
|  | hsa-miR-663b |
|  | hsa-miR-1285-5p |
|  | hsa-miR-1303 |
|  | hsa-miR-1249-3p |
|  | hsa-miR-1292-3p |
|  | hsa-miR-1470 |
|  | hsa-miR-1471 |
|  | hsa-miR-1908-3p |
|  | hsa-miR-1910-5p |
|  | hsa-miR-718 |
|  | hsa-miR-3135a |
|  | hsa-miR-1273d |
|  | hsa-miR-3615 |
|  | hsa-miR-3622a-3p |
|  | hsa-miR-4515 |
|  | hsa-miR-3972 |
|  | hsa-miR-4687-5p |
|  | hsa-miR-4687-3p |
|  | hsa-miR-4713-5p |
|  | hsa-miR-4728-3p |
|  | hsa-miR-4745-3p |
|  | hsa-miR-4747-3p |
|  | hsa-miR-4436b-5p |
|  | hsa-miR-5001-3p |
|  | hsa-miR-5096 |
|  | hsa-miR-5196-3p |
|  | hsa-miR-664b-3p |
|  | hsa-miR-5685 |
|  | hsa-miR-6089 |
|  | hsa-miR-6729-3p |
|  | hsa-miR-6746-3p |
|  | hsa-miR-6748-3p |
|  | hsa-miR-6749-3p |
|  | hsa-miR-6751-5p |
|  | hsa-miR-6752-3p |
|  | hsa-miR-6756-3p |
|  | hsa-miR-6763-3p |
|  | hsa-miR-6782-3p |
|  | hsa-miR-6786-5p |
|  | hsa-miR-6787-3p |
|  | hsa-miR-6793-5p |
|  | hsa-miR-6846-3p |
|  | hsa-miR-6847-5p |
|  | hsa-miR-6851-3p |
|  | hsa-miR-6862-3p |
|  | hsa-miR-6875-3p |
|  | hsa-miR-6877-3p |
|  | hsa-miR-6880-3p |
|  | hsa-miR-6894-3p |
|  | hsa-miR-7111-3p |
|  | hsa-miR-7113-3p |
|  | hsa-miR-7161-3p |
|  | hsa-miR-7703 |
|  | hsa-miR-1273h-3p |
| hsa_circ_0058058 | hsa-miR-1301-3p |
|  | hsa-miR-1291 |
|  | hsa-miR-2114-5p |
|  | hsa-miR-3194-5p |
|  | hsa-miR-4538 |
|  | hsa-miR-4649-5p |
| hsa_circ_0064136 | hsa-miR-134-5p |
|  | hsa-miR-328-5p |
|  | hsa-miR-601 |
|  | hsa-miR-762 |
|  | hsa-miR-1229-5p |
|  | hsa-miR-1237-5p |
|  | hsa-miR-3173-5p |
|  | hsa-miR-1193 |
|  | hsa-miR-3614-3p |
|  | hsa-miR-3619-3p |
|  | hsa-miR-3620-5p |
|  | hsa-miR-3622a-5p |
|  | hsa-miR-4459 |
|  | hsa-miR-1587 |
|  | hsa-miR-4539 |
|  | hsa-miR-4647 |
|  | hsa-miR-1343-5p |
|  | hsa-miR-4691-5p |
|  | hsa-miR-4743-3p |
|  | hsa-miR-4745-3p |
|  | hsa-miR-4763-3p |
|  | hsa-miR-4793-5p |
|  | hsa-miR-5787 |
|  | hsa-miR-6089 |
|  | hsa-miR-6511a-5p |
|  | hsa-miR-6722-3p |
|  | hsa-miR-6738-5p |
|  | hsa-miR-6754-5p |
|  | hsa-miR-6756-5p |
|  | hsa-miR-6763-5p |
|  | hsa-miR-6775-5p |
|  | hsa-miR-6786-5p |
|  | hsa-miR-6803-5p |
|  | hsa-miR-6824-5p |
|  | hsa-miR-6842-3p |
|  | hsa-miR-6848-5p |
|  | hsa-miR-6851-5p |
|  | hsa-miR-6769b-5p |
|  | hsa-miR-6871-5p |
|  | hsa-miR-6874-3p |
|  | hsa-miR-7107-5p |
|  | hsa-miR-8089 |
| hsa_circ_0071375 | hsa-miR-638 |
|  | hsa-miR-1207-5p |
|  | hsa-miR-4763-3p |
| hsa_circ_0077765 | hsa-miR-1224-5p |
|  | hsa-miR-762 |
|  | hsa-miR-1182 |
|  | hsa-miR-3689a-3p |
|  | hsa-miR-3689b-3p |
|  | hsa-miR-3689c |
|  | hsa-miR-4787-5p |
|  | hsa-miR-5001-5p |
|  | hsa-miR-6125 |
|  | hsa-miR-6751-5p |
|  | hsa-miR-6779-5p |
|  | hsa-miR-6803-5p |
|  | hsa-miR-6851-5p |
