# Supplementary material for: Comprehensive profiling of circular RNA expressions reveals potential diagnostic and prognostic biomarkers in multiple myeloma
Source: BMC Cancer. 2020 Jan 16;20:40. doi: 10.1186/s12885-020-6515-2 (PMC6966810; doi:10.1186/s12885-020-6515-2)
Supplement: Supplementary file 3 — Additional file 3. The potential target miRNAs of all the upregulated and downregulated ircRNAs. [file 12885_2020_6515_MOESM3_ESM.docx]

**Additional File 3**

The potential target miRNAs of all the upregulated and downregulated ircRNAs

| miRNA_ID | circRNA.ID |
| --- | --- |
| hsa-let-7e-3p | hsa_circ_0048025 |
| hsa-miR-15a-3p | hsa_circ_0007132 |
| hsa-miR-15a-3p | hsa_circ_0076995 |
| hsa-miR-16-1-3p | hsa_circ_0001934 |
| hsa-miR-18a-3p | hsa_circ_0009349 |
| hsa-miR-18a-3p | hsa_circ_0022723 |
| hsa-miR-18a-3p | hsa_circ_0092297 |
| hsa-miR-18a-3p | hsa_circ_0004705 |
| hsa-miR-23a-5p | hsa_circ_0003379 |
| hsa-miR-23a-5p | hsa_circ_0000375 |
| hsa-miR-23a-5p | hsa_circ_0006597 |
| hsa-miR-23a-5p | hsa_circ_0031933 |
| hsa-miR-23a-5p | hsa_circ_0004519 |
| hsa-miR-23a-5p | hsa_circ_0092277 |
| hsa-miR-23a-5p | hsa_circ_0074264 |
| hsa-miR-23a-5p | hsa_circ_0007132 |
| hsa-miR-23a-5p | hsa_circ_0004381 |
| hsa-miR-23a-5p | hsa_circ_0007145 |
| hsa-miR-23a-5p | hsa_circ_0001708 |
| hsa-miR-23a-5p | hsa_circ_0002094 |
| hsa-miR-24-3p | hsa_circ_0004692 |
| hsa-miR-24-3p | hsa_circ_0065147 |
| hsa-miR-24-3p | hsa_circ_0065149 |
| hsa-miR-24-3p | hsa_circ_0002569 |
| hsa-miR-25-3p | hsa_circ_0048025 |
| hsa-miR-26b-3p | hsa_circ_0001658 |
| hsa-miR-192-3p | hsa_circ_0043278 |
| hsa-miR-197-5p | hsa_circ_0007009 |
| hsa-miR-197-5p | hsa_circ_0017248 |
| hsa-miR-197-5p | hsa_circ_0006649 |
| hsa-miR-197-5p | hsa_circ_0022383 |
| hsa-miR-197-5p | hsa_circ_0003557 |
| hsa-miR-197-5p | hsa_circ_0004137 |
| hsa-miR-197-5p | hsa_circ_0004008 |
| hsa-miR-197-5p | hsa_circ_0000650 |
| hsa-miR-197-5p | hsa_circ_0036768 |
| hsa-miR-197-5p | hsa_circ_0045890 |
| hsa-miR-197-5p | hsa_circ_0006877 |
| hsa-miR-197-5p | hsa_circ_0000973 |
| hsa-miR-197-5p | hsa_circ_0003854 |
| hsa-miR-197-5p | hsa_circ_0002805 |
| hsa-miR-197-5p | hsa_circ_0069748 |
| hsa-miR-197-5p | hsa_circ_0071865 |
| hsa-miR-197-5p | hsa_circ_0005630 |
| hsa-miR-197-3p | hsa_circ_0003838 |
| hsa-miR-197-3p | hsa_circ_0008078 |
| hsa-miR-197-3p | hsa_circ_0007132 |
| hsa-miR-197-3p | hsa_circ_0002451 |
| hsa-miR-197-3p | hsa_circ_0005630 |
| hsa-miR-198 | hsa_circ_0020594 |
| hsa-miR-198 | hsa_circ_0002113 |
| hsa-miR-199a-5p | hsa_circ_0087305 |
| hsa-miR-139-5p | hsa_circ_0003379 |
| hsa-miR-139-3p | hsa_circ_0000375 |
| hsa-miR-139-3p | hsa_circ_0007146 |
| hsa-miR-139-3p | hsa_circ_0044195 |
| hsa-miR-139-3p | hsa_circ_0050119 |
| hsa-miR-139-3p | hsa_circ_0000918 |
| hsa-miR-139-3p | hsa_circ_0000936 |
| hsa-miR-139-3p | hsa_circ_0001756 |
| hsa-miR-139-3p | hsa_circ_0005630 |
| hsa-miR-34a-5p | hsa_circ_0002094 |
| hsa-miR-34a-5p | hsa_circ_0001947 |
| hsa-miR-205-5p | hsa_circ_0045890 |
| hsa-miR-210-5p | hsa_circ_0001400 |
| hsa-miR-210-5p | hsa_circ_0070396 |
| hsa-miR-210-5p | hsa_circ_0081343 |
| hsa-miR-210-3p | hsa_circ_0007146 |
| hsa-miR-210-3p | hsa_circ_0003340 |
| hsa-miR-210-3p | hsa_circ_0007685 |
| hsa-miR-210-3p | hsa_circ_0001910 |
| hsa-miR-211-3p | hsa_circ_0048025 |
| hsa-miR-211-3p | hsa_circ_0073379 |
| hsa-miR-211-3p | hsa_circ_0005540 |
| hsa-miR-211-3p | hsa_circ_0007521 |
| hsa-miR-212-5p | hsa_circ_0000395 |
| hsa-miR-212-5p | hsa_circ_0033144 |
| hsa-miR-214-5p | hsa_circ_0002805 |
| hsa-miR-214-3p | hsa_circ_0007009 |
| hsa-miR-214-3p | hsa_circ_0007609 |
| hsa-miR-214-3p | hsa_circ_0001658 |
| hsa-miR-200b-5p | hsa_circ_0006168 |
| hsa-let-7g-3p | hsa_circ_0002805 |
| hsa-let-7g-3p | hsa_circ_0006022 |
| hsa-let-7i-3p | hsa_circ_0006022 |
| hsa-miR-23b-3p | hsa_circ_0008225 |
| hsa-miR-124-5p | hsa_circ_0022383 |
| hsa-miR-125b-1-3p | hsa_circ_0009142 |
| hsa-miR-125b-1-3p | hsa_circ_0000650 |
| hsa-miR-125b-1-3p | hsa_circ_0036768 |
| hsa-miR-128-1-5p | hsa_circ_0000417 |
| hsa-miR-128-1-5p | hsa_circ_0002564 |
| hsa-miR-128-1-5p | hsa_circ_0002289 |
| hsa-miR-128-1-5p | hsa_circ_0052131 |
| hsa-miR-128-1-5p | hsa_circ_0092283 |
| hsa-miR-132-3p | hsa_circ_0006693 |
| hsa-miR-132-3p | hsa_circ_0001417 |
| hsa-miR-133a-3p | hsa_circ_0044195 |
| hsa-miR-133a-3p | hsa_circ_0007132 |
| hsa-miR-135a-3p | hsa_circ_0045890 |
| hsa-miR-141-5p | hsa_circ_0000497 |
| hsa-miR-143-5p | hsa_circ_0017446 |
| hsa-miR-143-5p | hsa_circ_0000206 |
| hsa-miR-143-5p | hsa_circ_0007132 |
| hsa-miR-145-5p | hsa_circ_0007132 |
| hsa-miR-191-3p | hsa_circ_0031933 |
| hsa-miR-125a-5p | hsa_circ_0009732 |
| hsa-miR-125a-3p | hsa_circ_0000592 |
| hsa-miR-125a-3p | hsa_circ_0002805 |
| hsa-miR-125b-2-3p | hsa_circ_0004692 |
| hsa-miR-127-5p | hsa_circ_0048025 |
| hsa-miR-127-5p | hsa_circ_0002113 |
| hsa-miR-127-5p | hsa_circ_0005630 |
| hsa-miR-134-5p | hsa_circ_0046430 |
| hsa-miR-134-5p | hsa_circ_0008261 |
| hsa-miR-134-5p | hsa_circ_0064136 |
| hsa-miR-134-3p | hsa_circ_0007846 |
| hsa-miR-134-3p | hsa_circ_0003854 |
| hsa-miR-134-3p | hsa_circ_0001400 |
| hsa-miR-134-3p | hsa_circ_0001573 |
| hsa-miR-149-5p | hsa_circ_0000395 |
| hsa-miR-149-5p | hsa_circ_0002805 |
| hsa-miR-149-5p | hsa_circ_0092299 |
| hsa-miR-149-3p | hsa_circ_0017248 |
| hsa-miR-149-3p | hsa_circ_0006254 |
| hsa-miR-149-3p | hsa_circ_0003768 |
| hsa-miR-149-3p | hsa_circ_0022383 |
| hsa-miR-149-3p | hsa_circ_0022392 |
| hsa-miR-149-3p | hsa_circ_0007846 |
| hsa-miR-149-3p | hsa_circ_0046430 |
| hsa-miR-149-3p | hsa_circ_0006877 |
| hsa-miR-149-3p | hsa_circ_0052131 |
| hsa-miR-149-3p | hsa_circ_0000973 |
| hsa-miR-149-3p | hsa_circ_0002805 |
| hsa-miR-149-3p | hsa_circ_0002113 |
| hsa-miR-149-3p | hsa_circ_0061936 |
| hsa-miR-149-3p | hsa_circ_0092299 |
| hsa-miR-149-3p | hsa_circ_0092277 |
| hsa-miR-149-3p | hsa_circ_0069748 |
| hsa-miR-149-3p | hsa_circ_0004381 |
| hsa-miR-149-3p | hsa_circ_0002755 |
| hsa-miR-149-3p | hsa_circ_0087855 |
| hsa-miR-149-3p | hsa_circ_0008812 |
| hsa-miR-149-3p | hsa_circ_0006174 |
| hsa-miR-149-3p | hsa_circ_0087861 |
| hsa-miR-149-3p | hsa_circ_0087862 |
| hsa-miR-149-3p | hsa_circ_0092125 |
| hsa-miR-150-3p | hsa_circ_0015004 |
| hsa-miR-150-3p | hsa_circ_0000417 |
| hsa-miR-150-3p | hsa_circ_0044195 |
| hsa-miR-150-3p | hsa_circ_0048025 |
| hsa-miR-150-3p | hsa_circ_0092299 |
| hsa-miR-150-3p | hsa_circ_0070040 |
| hsa-miR-150-3p | hsa_circ_0003340 |
| hsa-miR-184 | hsa_circ_0000660 |
| hsa-miR-185-5p | hsa_circ_0046292 |
| hsa-miR-185-3p | hsa_circ_0018909 |
| hsa-miR-185-3p | hsa_circ_0000417 |
| hsa-miR-185-3p | hsa_circ_0006597 |
| hsa-miR-185-3p | hsa_circ_0005733 |
| hsa-miR-185-3p | hsa_circ_0040039 |
| hsa-miR-185-3p | hsa_circ_0046430 |
| hsa-miR-185-3p | hsa_circ_0048025 |
| hsa-miR-185-3p | hsa_circ_0004003 |
| hsa-miR-185-3p | hsa_circ_0000950 |
| hsa-miR-185-3p | hsa_circ_0007132 |
| hsa-miR-185-3p | hsa_circ_0002094 |
| hsa-miR-188-5p | hsa_circ_0000417 |
| hsa-miR-193a-5p | hsa_circ_0052131 |
| hsa-miR-128-2-5p | hsa_circ_0000130 |
| hsa-miR-128-2-5p | hsa_circ_0000131 |
| hsa-miR-128-2-5p | hsa_circ_0017446 |
| hsa-miR-128-2-5p | hsa_circ_0000206 |
| hsa-miR-194-3p | hsa_circ_0007846 |
| hsa-miR-194-3p | hsa_circ_0061936 |
| hsa-miR-106b-3p | hsa_circ_0036763 |
| hsa-miR-106b-3p | hsa_circ_0000650 |
| hsa-miR-106b-3p | hsa_circ_0007846 |
| hsa-miR-106b-3p | hsa_circ_0040823 |
| hsa-miR-106b-3p | hsa_circ_0049657 |
| hsa-miR-106b-3p | hsa_circ_0002805 |
| hsa-miR-106b-3p | hsa_circ_0061179 |
| hsa-miR-106b-3p | hsa_circ_0001394 |
| hsa-miR-219a-2-3p | hsa_circ_0072857 |
| hsa-miR-34b-5p | hsa_circ_0040921 |
| hsa-miR-99b-5p | hsa_circ_0001658 |
| hsa-miR-99b-3p | hsa_circ_0018909 |
| hsa-miR-99b-3p | hsa_circ_0036763 |
| hsa-miR-99b-3p | hsa_circ_0000650 |
| hsa-miR-99b-3p | hsa_circ_0047700 |
| hsa-miR-296-5p | hsa_circ_0000690 |
| hsa-miR-296-5p | hsa_circ_0000996 |
| hsa-miR-296-5p | hsa_circ_0002805 |
| hsa-miR-296-5p | hsa_circ_0007132 |
| hsa-miR-296-3p | hsa_circ_0002402 |
| hsa-miR-296-3p | hsa_circ_0003557 |
| hsa-miR-296-3p | hsa_circ_0000417 |
| hsa-miR-296-3p | hsa_circ_0028190 |
| hsa-miR-296-3p | hsa_circ_0000497 |
| hsa-miR-296-3p | hsa_circ_0002564 |
| hsa-miR-296-3p | hsa_circ_0002289 |
| hsa-miR-296-3p | hsa_circ_0007146 |
| hsa-miR-296-3p | hsa_circ_0007846 |
| hsa-miR-296-3p | hsa_circ_0002926 |
| hsa-miR-296-3p | hsa_circ_0000973 |
| hsa-miR-296-3p | hsa_circ_0002805 |
| hsa-miR-296-3p | hsa_circ_0061179 |
| hsa-miR-296-3p | hsa_circ_0071311 |
| hsa-miR-296-3p | hsa_circ_0075748 |
| hsa-miR-296-3p | hsa_circ_0001707 |
| hsa-miR-361-3p | hsa_circ_0003379 |
| hsa-miR-365b-5p | hsa_circ_0005571 |
| hsa-miR-365b-5p | hsa_circ_0092299 |
| hsa-miR-365b-5p | hsa_circ_0076793 |
| hsa-miR-370-3p | hsa_circ_0002402 |
| hsa-miR-370-3p | hsa_circ_0006649 |
| hsa-miR-370-3p | hsa_circ_0003557 |
| hsa-miR-370-3p | hsa_circ_0000417 |
| hsa-miR-370-3p | hsa_circ_0008274 |
| hsa-miR-370-3p | hsa_circ_0031933 |
| hsa-miR-370-3p | hsa_circ_0002564 |
| hsa-miR-370-3p | hsa_circ_0002289 |
| hsa-miR-370-3p | hsa_circ_0045890 |
| hsa-miR-370-3p | hsa_circ_0049657 |
| hsa-miR-370-3p | hsa_circ_0054086 |
| hsa-miR-370-3p | hsa_circ_0002805 |
| hsa-miR-370-3p | hsa_circ_0002113 |
| hsa-miR-370-3p | hsa_circ_0004692 |
| hsa-miR-370-3p | hsa_circ_0084429 |
| hsa-miR-370-3p | hsa_circ_0007521 |
| hsa-miR-377-5p | hsa_circ_0040823 |
| hsa-miR-378a-5p | hsa_circ_0040921 |
| hsa-miR-378a-5p | hsa_circ_0000973 |
| hsa-miR-378a-5p | hsa_circ_0092297 |
| hsa-miR-378a-3p | hsa_circ_0092283 |
| hsa-miR-330-5p | hsa_circ_0008521 |
| hsa-miR-330-5p | hsa_circ_0003848 |
| hsa-miR-330-5p | hsa_circ_0002564 |
| hsa-miR-330-5p | hsa_circ_0002289 |
| hsa-miR-330-5p | hsa_circ_0035944 |
| hsa-miR-330-5p | hsa_circ_0046430 |
| hsa-miR-330-5p | hsa_circ_0003602 |
| hsa-miR-330-5p | hsa_circ_0008639 |
| hsa-miR-330-5p | hsa_circ_0001394 |
| hsa-miR-330-5p | hsa_circ_0001400 |
| hsa-miR-330-5p | hsa_circ_0006693 |
| hsa-miR-330-5p | hsa_circ_0001417 |
| hsa-miR-330-5p | hsa_circ_0007132 |
| hsa-miR-330-5p | hsa_circ_0005630 |
| hsa-miR-330-5p | hsa_circ_0084789 |
| hsa-miR-328-5p | hsa_circ_0009581 |
| hsa-miR-328-5p | hsa_circ_0006837 |
| hsa-miR-328-5p | hsa_circ_0002402 |
| hsa-miR-328-5p | hsa_circ_0007009 |
| hsa-miR-328-5p | hsa_circ_0015004 |
| hsa-miR-328-5p | hsa_circ_0017248 |
| hsa-miR-328-5p | hsa_circ_0017289 |
| hsa-miR-328-5p | hsa_circ_0005090 |
| hsa-miR-328-5p | hsa_circ_0000220 |
| hsa-miR-328-5p | hsa_circ_0006665 |
| hsa-miR-328-5p | hsa_circ_0006649 |
| hsa-miR-328-5p | hsa_circ_0008102 |
| hsa-miR-328-5p | hsa_circ_0003168 |
| hsa-miR-328-5p | hsa_circ_0020005 |
| hsa-miR-328-5p | hsa_circ_0003379 |
| hsa-miR-328-5p | hsa_circ_0022383 |
| hsa-miR-328-5p | hsa_circ_0007372 |
| hsa-miR-328-5p | hsa_circ_0000375 |
| hsa-miR-328-5p | hsa_circ_0000417 |
| hsa-miR-328-5p | hsa_circ_0028190 |
| hsa-miR-328-5p | hsa_circ_0000497 |
| hsa-miR-328-5p | hsa_circ_0031933 |
| hsa-miR-328-5p | hsa_circ_0000650 |
| hsa-miR-328-5p | hsa_circ_0036768 |
| hsa-miR-328-5p | hsa_circ_0007846 |
| hsa-miR-328-5p | hsa_circ_0002696 |
| hsa-miR-328-5p | hsa_circ_0040823 |
| hsa-miR-328-5p | hsa_circ_0045890 |
| hsa-miR-328-5p | hsa_circ_0046430 |
| hsa-miR-328-5p | hsa_circ_0006877 |
| hsa-miR-328-5p | hsa_circ_0050119 |
| hsa-miR-328-5p | hsa_circ_0000918 |
| hsa-miR-328-5p | hsa_circ_0008030 |
| hsa-miR-328-5p | hsa_circ_0000936 |
| hsa-miR-328-5p | hsa_circ_0000950 |
| hsa-miR-328-5p | hsa_circ_0000973 |
| hsa-miR-328-5p | hsa_circ_0055904 |
| hsa-miR-328-5p | hsa_circ_0004218 |
| hsa-miR-328-5p | hsa_circ_0002805 |
| hsa-miR-328-5p | hsa_circ_0061179 |
| hsa-miR-328-5p | hsa_circ_0092299 |
| hsa-miR-328-5p | hsa_circ_0004470 |
| hsa-miR-328-5p | hsa_circ_0002077 |
| hsa-miR-328-5p | hsa_circ_0064136 |
| hsa-miR-328-5p | hsa_circ_0004692 |
| hsa-miR-328-5p | hsa_circ_0065147 |
| hsa-miR-328-5p | hsa_circ_0065149 |
| hsa-miR-328-5p | hsa_circ_0002569 |
| hsa-miR-328-5p | hsa_circ_0092277 |
| hsa-miR-328-5p | hsa_circ_0001400 |
| hsa-miR-328-5p | hsa_circ_0071311 |
| hsa-miR-328-5p | hsa_circ_0073379 |
| hsa-miR-328-5p | hsa_circ_0005540 |
| hsa-miR-328-5p | hsa_circ_0074264 |
| hsa-miR-328-5p | hsa_circ_0001549 |
| hsa-miR-328-5p | hsa_circ_0001573 |
| hsa-miR-328-5p | hsa_circ_0007132 |
| hsa-miR-328-5p | hsa_circ_0004381 |
| hsa-miR-328-5p | hsa_circ_0003340 |
| hsa-miR-328-5p | hsa_circ_0009092 |
| hsa-miR-328-5p | hsa_circ_0001708 |
| hsa-miR-328-5p | hsa_circ_0002094 |
| hsa-miR-328-5p | hsa_circ_0005630 |
| hsa-miR-328-5p | hsa_circ_0084429 |
| hsa-miR-328-5p | hsa_circ_0084615 |
| hsa-miR-328-5p | hsa_circ_0001806 |
| hsa-miR-328-5p | hsa_circ_0084789 |
| hsa-miR-328-5p | hsa_circ_0006566 |
| hsa-miR-328-5p | hsa_circ_0087288 |
| hsa-miR-328-5p | hsa_circ_0087305 |
| hsa-miR-328-5p | hsa_circ_0087855 |
| hsa-miR-328-5p | hsa_circ_0008812 |
| hsa-miR-328-5p | hsa_circ_0006174 |
| hsa-miR-328-5p | hsa_circ_0087861 |
| hsa-miR-328-5p | hsa_circ_0087862 |
| hsa-miR-328-5p | hsa_circ_0092125 |
| hsa-miR-328-3p | hsa_circ_0009732 |
| hsa-miR-328-3p | hsa_circ_0020594 |
| hsa-miR-328-3p | hsa_circ_0000690 |
| hsa-miR-328-3p | hsa_circ_0048025 |
| hsa-miR-328-3p | hsa_circ_0092283 |
| hsa-miR-328-3p | hsa_circ_0004692 |
| hsa-miR-328-3p | hsa_circ_0065147 |
| hsa-miR-328-3p | hsa_circ_0065149 |
| hsa-miR-328-3p | hsa_circ_0002569 |
| hsa-miR-328-3p | hsa_circ_0001573 |
| hsa-miR-328-3p | hsa_circ_0007132 |
| hsa-miR-328-3p | hsa_circ_0002755 |
| hsa-miR-323a-5p | hsa_circ_0007437 |
| hsa-miR-323a-5p | hsa_circ_0007693 |
| hsa-miR-323a-5p | hsa_circ_0012151 |
| hsa-miR-323a-5p | hsa_circ_0000682 |
| hsa-miR-323a-5p | hsa_circ_0046430 |
| hsa-miR-323a-5p | hsa_circ_0004218 |
| hsa-miR-323a-5p | hsa_circ_0007132 |
| hsa-miR-326 | hsa_circ_0022383 |
| hsa-miR-326 | hsa_circ_0049998 |
| hsa-miR-326 | hsa_circ_0002805 |
| hsa-miR-326 | hsa_circ_0007137 |
| hsa-miR-326 | hsa_circ_0007132 |
| hsa-miR-151a-5p | hsa_circ_0003854 |
| hsa-miR-331-5p | hsa_circ_0007132 |
| hsa-miR-331-3p | hsa_circ_0046430 |
| hsa-miR-331-3p | hsa_circ_0006693 |
| hsa-miR-331-3p | hsa_circ_0001417 |
| hsa-miR-331-3p | hsa_circ_0007132 |
| hsa-miR-324-5p | hsa_circ_0040921 |
| hsa-miR-324-5p | hsa_circ_0049657 |
| hsa-miR-324-5p | hsa_circ_0007137 |
| hsa-miR-324-5p | hsa_circ_0007132 |
| hsa-miR-324-3p | hsa_circ_0022723 |
| hsa-miR-324-3p | hsa_circ_0007846 |
| hsa-miR-324-3p | hsa_circ_0002926 |
| hsa-miR-339-5p | hsa_circ_0011571 |
| hsa-miR-339-5p | hsa_circ_0011572 |
| hsa-miR-339-5p | hsa_circ_0007009 |
| hsa-miR-339-5p | hsa_circ_0000463 |
| hsa-miR-339-5p | hsa_circ_0045890 |
| hsa-miR-339-5p | hsa_circ_0054086 |
| hsa-miR-339-5p | hsa_circ_0003854 |
| hsa-miR-339-5p | hsa_circ_0092299 |
| hsa-miR-339-5p | hsa_circ_0070396 |
| hsa-miR-339-5p | hsa_circ_0007132 |
| hsa-miR-339-5p | hsa_circ_0007145 |
| hsa-miR-339-5p | hsa_circ_0081423 |
| hsa-miR-339-3p | hsa_circ_0007132 |
| hsa-miR-133b | hsa_circ_0007132 |
| hsa-miR-345-5p | hsa_circ_0011571 |
| hsa-miR-345-5p | hsa_circ_0011572 |
| hsa-miR-345-5p | hsa_circ_0004519 |
| hsa-miR-345-5p | hsa_circ_0001400 |
| hsa-miR-345-5p | hsa_circ_0071311 |
| hsa-miR-345-5p | hsa_circ_0003528 |
| hsa-miR-345-5p | hsa_circ_0007132 |
| hsa-miR-345-3p | hsa_circ_0009581 |
| hsa-miR-345-3p | hsa_circ_0006837 |
| hsa-miR-345-3p | hsa_circ_0033144 |
| hsa-miR-345-3p | hsa_circ_0000682 |
| hsa-miR-345-3p | hsa_circ_0040039 |
| hsa-miR-345-3p | hsa_circ_0004354 |
| hsa-miR-345-3p | hsa_circ_0092316 |
| hsa-miR-345-3p | hsa_circ_0087288 |
| hsa-miR-346 | hsa_circ_0034189 |
| hsa-miR-346 | hsa_circ_0007132 |
| hsa-miR-346 | hsa_circ_0078522 |
| hsa-miR-346 | hsa_circ_0084789 |
| hsa-miR-422a | hsa_circ_0087288 |
| hsa-miR-423-5p | hsa_circ_0029405 |
| hsa-miR-423-5p | hsa_circ_0092277 |
| hsa-miR-423-5p | hsa_circ_0066444 |
| hsa-miR-423-3p | hsa_circ_0025388 |
| hsa-miR-423-3p | hsa_circ_0007146 |
| hsa-miR-423-3p | hsa_circ_0062397 |
| hsa-miR-423-3p | hsa_circ_0092277 |
| hsa-miR-423-3p | hsa_circ_0007132 |
| hsa-miR-423-3p | hsa_circ_0075748 |
| hsa-miR-425-3p | hsa_circ_0003168 |
| hsa-miR-18b-3p | hsa_circ_0022723 |
| hsa-miR-18b-3p | hsa_circ_0070396 |
| hsa-miR-431-3p | hsa_circ_0016863 |
| hsa-miR-431-3p | hsa_circ_0092297 |
| hsa-miR-431-3p | hsa_circ_0070396 |
| hsa-miR-433-3p | hsa_circ_0004381 |
| hsa-miR-412-5p | hsa_circ_0007609 |
| hsa-miR-412-3p | hsa_circ_0045890 |
| hsa-miR-483-5p | hsa_circ_0006357 |
| hsa-miR-483-3p | hsa_circ_0020594 |
| hsa-miR-483-3p | hsa_circ_0004692 |
| hsa-miR-483-3p | hsa_circ_0005630 |
| hsa-miR-484 | hsa_circ_0020594 |
| hsa-miR-484 | hsa_circ_0092299 |
| hsa-miR-484 | hsa_circ_0007132 |
| hsa-miR-484 | hsa_circ_0005630 |
| hsa-miR-485-5p | hsa_circ_0052131 |
| hsa-miR-485-3p | hsa_circ_0049657 |
| hsa-miR-486-5p | hsa_circ_0009732 |
| hsa-miR-486-5p | hsa_circ_0005733 |
| hsa-miR-486-5p | hsa_circ_0040039 |
| hsa-miR-486-5p | hsa_circ_0081873 |
| hsa-miR-486-5p | hsa_circ_0005630 |
| hsa-miR-487a-5p | hsa_circ_0003489 |
| hsa-miR-490-5p | hsa_circ_0002402 |
| hsa-miR-490-5p | hsa_circ_0071311 |
| hsa-miR-490-5p | hsa_circ_0003738 |
| hsa-miR-490-3p | hsa_circ_0050119 |
| hsa-miR-490-3p | hsa_circ_0092299 |
| hsa-miR-491-5p | hsa_circ_0007132 |
| hsa-miR-491-5p | hsa_circ_0005630 |
| hsa-miR-432-5p | hsa_circ_0003557 |
| hsa-miR-432-5p | hsa_circ_0054086 |
| hsa-miR-432-3p | hsa_circ_0003865 |
| hsa-miR-432-3p | hsa_circ_0001258 |
| hsa-miR-432-3p | hsa_circ_0001573 |
| hsa-miR-494-5p | hsa_circ_0044195 |
| hsa-miR-494-5p | hsa_circ_0048025 |
| hsa-miR-181d-3p | hsa_circ_0029405 |
| hsa-miR-512-5p | hsa_circ_0007009 |
| hsa-miR-498 | hsa_circ_0000996 |
| hsa-miR-498 | hsa_circ_0001573 |
| hsa-miR-526b-5p | hsa_circ_0007137 |
| hsa-miR-523-3p | hsa_circ_0005079 |
| hsa-miR-523-3p | hsa_circ_0069399 |
| hsa-miR-518b | hsa_circ_0007132 |
| hsa-miR-518c-5p | hsa_circ_0092299 |
| hsa-miR-524-3p | hsa_circ_0000375 |
| hsa-miR-519d-5p | hsa_circ_0005630 |
| hsa-miR-518d-3p | hsa_circ_0007132 |
| hsa-miR-500a-5p | hsa_circ_0040823 |
| hsa-miR-503-5p | hsa_circ_0043691 |
| hsa-miR-503-5p | hsa_circ_0008078 |
| hsa-miR-503-3p | hsa_circ_0011422 |
| hsa-miR-503-3p | hsa_circ_0031933 |
| hsa-miR-503-3p | hsa_circ_0040039 |
| hsa-miR-503-3p | hsa_circ_0004354 |
| hsa-miR-503-3p | hsa_circ_0060043 |
| hsa-miR-503-3p | hsa_circ_0001756 |
| hsa-miR-503-3p | hsa_circ_0001910 |
| hsa-miR-504-5p | hsa_circ_0000247 |
| hsa-miR-504-3p | hsa_circ_0003854 |
| hsa-miR-509-3p | hsa_circ_0060043 |
| hsa-miR-487b-5p | hsa_circ_0007132 |
| hsa-miR-551a | hsa_circ_0041050 |
| hsa-miR-551a | hsa_circ_0005630 |
| hsa-miR-92b-5p | hsa_circ_0048025 |
| hsa-miR-92b-5p | hsa_circ_0003854 |
| hsa-miR-92b-5p | hsa_circ_0002805 |
| hsa-miR-92b-5p | hsa_circ_0061179 |
| hsa-miR-564 | hsa_circ_0003379 |
| hsa-miR-566 | hsa_circ_0043522 |
| hsa-miR-572 | hsa_circ_0049888 |
| hsa-miR-572 | hsa_circ_0050119 |
| hsa-miR-572 | hsa_circ_0008590 |
| hsa-miR-572 | hsa_circ_0003854 |
| hsa-miR-572 | hsa_circ_0007132 |
| hsa-miR-581 | hsa_circ_0001658 |
| hsa-miR-584-3p | hsa_circ_0006608 |
| hsa-miR-584-3p | hsa_circ_0007372 |
| hsa-miR-584-3p | hsa_circ_0040921 |
| hsa-miR-584-3p | hsa_circ_0006884 |
| hsa-miR-550a-5p | hsa_circ_0005733 |
| hsa-miR-550a-5p | hsa_circ_0040039 |
| hsa-miR-550a-5p | hsa_circ_0072437 |
| hsa-miR-550a-5p | hsa_circ_0084789 |
| hsa-miR-593-5p | hsa_circ_0007001 |
| hsa-miR-593-5p | hsa_circ_0007846 |
| hsa-miR-593-5p | hsa_circ_0003315 |
| hsa-miR-593-5p | hsa_circ_0050119 |
| hsa-miR-593-5p | hsa_circ_0001658 |
| hsa-miR-596 | hsa_circ_0003168 |
| hsa-miR-596 | hsa_circ_0030051 |
| hsa-miR-596 | hsa_circ_0000650 |
| hsa-miR-596 | hsa_circ_0036768 |
| hsa-miR-596 | hsa_circ_0048025 |
| hsa-miR-596 | hsa_circ_0000973 |
| hsa-miR-596 | hsa_circ_0061052 |
| hsa-miR-596 | hsa_circ_0002805 |
| hsa-miR-596 | hsa_circ_0061179 |
| hsa-miR-596 | hsa_circ_0070396 |
| hsa-miR-596 | hsa_circ_0007132 |
| hsa-miR-597-3p | hsa_circ_0028190 |
| hsa-miR-598-5p | hsa_circ_0006608 |
| hsa-miR-598-5p | hsa_circ_0000660 |
| hsa-miR-598-5p | hsa_circ_0060904 |
| hsa-miR-598-5p | hsa_circ_0066444 |
| hsa-miR-601 | hsa_circ_0000417 |
| hsa-miR-601 | hsa_circ_0064136 |
| hsa-miR-602 | hsa_circ_0003168 |
| hsa-miR-602 | hsa_circ_0003557 |
| hsa-miR-602 | hsa_circ_0007146 |
| hsa-miR-602 | hsa_circ_0048025 |
| hsa-miR-602 | hsa_circ_0092283 |
| hsa-miR-602 | hsa_circ_0007132 |
| hsa-miR-608 | hsa_circ_0009349 |
| hsa-miR-608 | hsa_circ_0009357 |
| hsa-miR-608 | hsa_circ_0016863 |
| hsa-miR-608 | hsa_circ_0016867 |
| hsa-miR-608 | hsa_circ_0006608 |
| hsa-miR-608 | hsa_circ_0017639 |
| hsa-miR-608 | hsa_circ_0022505 |
| hsa-miR-608 | hsa_circ_0028190 |
| hsa-miR-608 | hsa_circ_0003489 |
| hsa-miR-608 | hsa_circ_0007846 |
| hsa-miR-608 | hsa_circ_0042799 |
| hsa-miR-608 | hsa_circ_0045890 |
| hsa-miR-608 | hsa_circ_0045905 |
| hsa-miR-608 | hsa_circ_0046430 |
| hsa-miR-608 | hsa_circ_0004003 |
| hsa-miR-608 | hsa_circ_0004891 |
| hsa-miR-608 | hsa_circ_0002926 |
| hsa-miR-608 | hsa_circ_0049657 |
| hsa-miR-608 | hsa_circ_0000918 |
| hsa-miR-608 | hsa_circ_0000950 |
| hsa-miR-608 | hsa_circ_0092297 |
| hsa-miR-608 | hsa_circ_0004218 |
| hsa-miR-608 | hsa_circ_0002805 |
| hsa-miR-608 | hsa_circ_0061179 |
| hsa-miR-608 | hsa_circ_0007609 |
| hsa-miR-608 | hsa_circ_0092299 |
| hsa-miR-608 | hsa_circ_0063331 |
| hsa-miR-608 | hsa_circ_0004692 |
| hsa-miR-608 | hsa_circ_0065147 |
| hsa-miR-608 | hsa_circ_0004276 |
| hsa-miR-608 | hsa_circ_0001400 |
| hsa-miR-608 | hsa_circ_0069748 |
| hsa-miR-608 | hsa_circ_0001439 |
| hsa-miR-608 | hsa_circ_0072857 |
| hsa-miR-608 | hsa_circ_0003340 |
| hsa-miR-608 | hsa_circ_0009092 |
| hsa-miR-608 | hsa_circ_0002755 |
| hsa-miR-608 | hsa_circ_0002094 |
| hsa-miR-608 | hsa_circ_0001806 |
| hsa-miR-608 | hsa_circ_0084789 |
| hsa-miR-608 | hsa_circ_0087305 |
| hsa-miR-611 | hsa_circ_0036763 |
| hsa-miR-611 | hsa_circ_0000650 |
| hsa-miR-611 | hsa_circ_0046430 |
| hsa-miR-611 | hsa_circ_0049998 |
| hsa-miR-611 | hsa_circ_0092297 |
| hsa-miR-611 | hsa_circ_0001394 |
| hsa-miR-611 | hsa_circ_0006693 |
| hsa-miR-611 | hsa_circ_0001417 |
| hsa-miR-611 | hsa_circ_0007132 |
| hsa-miR-611 | hsa_circ_0005630 |
| hsa-miR-611 | hsa_circ_0006566 |
| hsa-miR-612 | hsa_circ_0002402 |
| hsa-miR-612 | hsa_circ_0018909 |
| hsa-miR-612 | hsa_circ_0008102 |
| hsa-miR-612 | hsa_circ_0029976 |
| hsa-miR-612 | hsa_circ_0000497 |
| hsa-miR-612 | hsa_circ_0036763 |
| hsa-miR-612 | hsa_circ_0000650 |
| hsa-miR-612 | hsa_circ_0000690 |
| hsa-miR-612 | hsa_circ_0004519 |
| hsa-miR-612 | hsa_circ_0045890 |
| hsa-miR-612 | hsa_circ_0045905 |
| hsa-miR-612 | hsa_circ_0046430 |
| hsa-miR-612 | hsa_circ_0001092 |
| hsa-miR-612 | hsa_circ_0002805 |
| hsa-miR-612 | hsa_circ_0061179 |
| hsa-miR-612 | hsa_circ_0092299 |
| hsa-miR-612 | hsa_circ_0092283 |
| hsa-miR-612 | hsa_circ_0004692 |
| hsa-miR-612 | hsa_circ_0065147 |
| hsa-miR-612 | hsa_circ_0065149 |
| hsa-miR-612 | hsa_circ_0002569 |
| hsa-miR-612 | hsa_circ_0003602 |
| hsa-miR-612 | hsa_circ_0001573 |
| hsa-miR-612 | hsa_circ_0007132 |
| hsa-miR-612 | hsa_circ_0007145 |
| hsa-miR-612 | hsa_circ_0001748 |
| hsa-miR-612 | hsa_circ_0005630 |
| hsa-miR-614 | hsa_circ_0001394 |
| hsa-miR-614 | hsa_circ_0007132 |
| hsa-miR-614 | hsa_circ_0003340 |
| hsa-miR-615-5p | hsa_circ_0006608 |
| hsa-miR-615-5p | hsa_circ_0018909 |
| hsa-miR-615-5p | hsa_circ_0007001 |
| hsa-miR-615-5p | hsa_circ_0000375 |
| hsa-miR-615-5p | hsa_circ_0000690 |
| hsa-miR-615-5p | hsa_circ_0046430 |
| hsa-miR-615-5p | hsa_circ_0092297 |
| hsa-miR-615-5p | hsa_circ_0003854 |
| hsa-miR-615-5p | hsa_circ_0092283 |
| hsa-miR-615-5p | hsa_circ_0001394 |
| hsa-miR-615-5p | hsa_circ_0070396 |
| hsa-miR-615-5p | hsa_circ_0007132 |
| hsa-miR-615-5p | hsa_circ_0004381 |
| hsa-miR-615-5p | hsa_circ_0081343 |
| hsa-miR-615-5p | hsa_circ_0081423 |
| hsa-miR-615-5p | hsa_circ_0002094 |
| hsa-miR-615-3p | hsa_circ_0011571 |
| hsa-miR-615-3p | hsa_circ_0011572 |
| hsa-miR-615-3p | hsa_circ_0020594 |
| hsa-miR-615-3p | hsa_circ_0022015 |
| hsa-miR-615-3p | hsa_circ_0092299 |
| hsa-miR-615-3p | hsa_circ_0092283 |
| hsa-miR-615-3p | hsa_circ_0005630 |
| hsa-miR-619-5p | hsa_circ_0000909 |
| hsa-miR-619-5p | hsa_circ_0000973 |
| hsa-miR-619-5p | hsa_circ_0007132 |
| hsa-miR-619-3p | hsa_circ_0002805 |
| hsa-miR-619-3p | hsa_circ_0061179 |
| hsa-miR-619-3p | hsa_circ_0005630 |
| hsa-miR-623 | hsa_circ_0007009 |
| hsa-miR-623 | hsa_circ_0022392 |
| hsa-miR-623 | hsa_circ_0040921 |
| hsa-miR-623 | hsa_circ_0049888 |
| hsa-miR-629-3p | hsa_circ_0007132 |
| hsa-miR-33b-3p | hsa_circ_0022723 |
| hsa-miR-33b-3p | hsa_circ_0002805 |
| hsa-miR-33b-3p | hsa_circ_0061179 |
| hsa-miR-33b-3p | hsa_circ_0004692 |
| hsa-miR-33b-3p | hsa_circ_0065147 |
| hsa-miR-33b-3p | hsa_circ_0067913 |
| hsa-miR-33b-3p | hsa_circ_0067919 |
| hsa-miR-33b-3p | hsa_circ_0005362 |
| hsa-miR-33b-3p | hsa_circ_0007132 |
| hsa-miR-33b-3p | hsa_circ_0081343 |
| hsa-miR-636 | hsa_circ_0034189 |
| hsa-miR-636 | hsa_circ_0092297 |
| hsa-miR-636 | hsa_circ_0001394 |
| hsa-miR-636 | hsa_circ_0007132 |
| hsa-miR-636 | hsa_circ_0002094 |
| hsa-miR-636 | hsa_circ_0007521 |
| hsa-miR-636 | hsa_circ_0005982 |
| hsa-miR-636 | hsa_circ_0003221 |
| hsa-miR-636 | hsa_circ_0002483 |
| hsa-miR-636 | hsa_circ_0006646 |
| hsa-miR-637 | hsa_circ_0009349 |
| hsa-miR-637 | hsa_circ_0009357 |
| hsa-miR-637 | hsa_circ_0002402 |
| hsa-miR-637 | hsa_circ_0000130 |
| hsa-miR-637 | hsa_circ_0000131 |
| hsa-miR-637 | hsa_circ_0003557 |
| hsa-miR-637 | hsa_circ_0007372 |
| hsa-miR-637 | hsa_circ_0000497 |
| hsa-miR-637 | hsa_circ_0036763 |
| hsa-miR-637 | hsa_circ_0000650 |
| hsa-miR-637 | hsa_circ_0036768 |
| hsa-miR-637 | hsa_circ_0004519 |
| hsa-miR-637 | hsa_circ_0040823 |
| hsa-miR-637 | hsa_circ_0046430 |
| hsa-miR-637 | hsa_circ_0048025 |
| hsa-miR-637 | hsa_circ_0002926 |
| hsa-miR-637 | hsa_circ_0049657 |
| hsa-miR-637 | hsa_circ_0060043 |
| hsa-miR-637 | hsa_circ_0002805 |
| hsa-miR-637 | hsa_circ_0061179 |
| hsa-miR-637 | hsa_circ_0092299 |
| hsa-miR-637 | hsa_circ_0071311 |
| hsa-miR-637 | hsa_circ_0001573 |
| hsa-miR-637 | hsa_circ_0007132 |
| hsa-miR-637 | hsa_circ_0006022 |
| hsa-miR-637 | hsa_circ_0087305 |
| hsa-miR-637 | hsa_circ_0001947 |
| hsa-miR-638 | hsa_circ_0002402 |
| hsa-miR-638 | hsa_circ_0007437 |
| hsa-miR-638 | hsa_circ_0007693 |
| hsa-miR-638 | hsa_circ_0007009 |
| hsa-miR-638 | hsa_circ_0015004 |
| hsa-miR-638 | hsa_circ_0007015 |
| hsa-miR-638 | hsa_circ_0007778 |
| hsa-miR-638 | hsa_circ_0018909 |
| hsa-miR-638 | hsa_circ_0003168 |
| hsa-miR-638 | hsa_circ_0020594 |
| hsa-miR-638 | hsa_circ_0006254 |
| hsa-miR-638 | hsa_circ_0003768 |
| hsa-miR-638 | hsa_circ_0022723 |
| hsa-miR-638 | hsa_circ_0003557 |
| hsa-miR-638 | hsa_circ_0000417 |
| hsa-miR-638 | hsa_circ_0001955 |
| hsa-miR-638 | hsa_circ_0000650 |
| hsa-miR-638 | hsa_circ_0036768 |
| hsa-miR-638 | hsa_circ_0007146 |
| hsa-miR-638 | hsa_circ_0007846 |
| hsa-miR-638 | hsa_circ_0040823 |
| hsa-miR-638 | hsa_circ_0040921 |
| hsa-miR-638 | hsa_circ_0092337 |
| hsa-miR-638 | hsa_circ_0043691 |
| hsa-miR-638 | hsa_circ_0045890 |
| hsa-miR-638 | hsa_circ_0046430 |
| hsa-miR-638 | hsa_circ_0048025 |
| hsa-miR-638 | hsa_circ_0000909 |
| hsa-miR-638 | hsa_circ_0049998 |
| hsa-miR-638 | hsa_circ_0005571 |
| hsa-miR-638 | hsa_circ_0050119 |
| hsa-miR-638 | hsa_circ_0000973 |
| hsa-miR-638 | hsa_circ_0003854 |
| hsa-miR-638 | hsa_circ_0004218 |
| hsa-miR-638 | hsa_circ_0002805 |
| hsa-miR-638 | hsa_circ_0061179 |
| hsa-miR-638 | hsa_circ_0061936 |
| hsa-miR-638 | hsa_circ_0092299 |
| hsa-miR-638 | hsa_circ_0001394 |
| hsa-miR-638 | hsa_circ_0001400 |
| hsa-miR-638 | hsa_circ_0070396 |
| hsa-miR-638 | hsa_circ_0070467 |
| hsa-miR-638 | hsa_circ_0071375 |
| hsa-miR-638 | hsa_circ_0071869 |
| hsa-miR-638 | hsa_circ_0072437 |
| hsa-miR-638 | hsa_circ_0072857 |
| hsa-miR-638 | hsa_circ_0073379 |
| hsa-miR-638 | hsa_circ_0005540 |
| hsa-miR-638 | hsa_circ_0006528 |
| hsa-miR-638 | hsa_circ_0074816 |
| hsa-miR-638 | hsa_circ_0074817 |
| hsa-miR-638 | hsa_circ_0001577 |
| hsa-miR-638 | hsa_circ_0001578 |
| hsa-miR-638 | hsa_circ_0007132 |
| hsa-miR-638 | hsa_circ_0009092 |
| hsa-miR-638 | hsa_circ_0001708 |
| hsa-miR-638 | hsa_circ_0002755 |
| hsa-miR-638 | hsa_circ_0002094 |
| hsa-miR-638 | hsa_circ_0005630 |
| hsa-miR-638 | hsa_circ_0001806 |
| hsa-miR-638 | hsa_circ_0001824 |
| hsa-miR-638 | hsa_circ_0008934 |
| hsa-miR-638 | hsa_circ_0005273 |
| hsa-miR-638 | hsa_circ_0087288 |
| hsa-miR-638 | hsa_circ_0087305 |
| hsa-miR-638 | hsa_circ_0001947 |
| hsa-miR-638 | hsa_circ_0092125 |
| hsa-miR-639 | hsa_circ_0007846 |
| hsa-miR-647 | hsa_circ_0008078 |
| hsa-miR-650 | hsa_circ_0007846 |
| hsa-miR-650 | hsa_circ_0001394 |
| hsa-miR-652-5p | hsa_circ_0045890 |
| hsa-miR-652-5p | hsa_circ_0004692 |
| hsa-miR-661 | hsa_circ_0009349 |
| hsa-miR-661 | hsa_circ_0009732 |
| hsa-miR-661 | hsa_circ_0017289 |
| hsa-miR-661 | hsa_circ_0007001 |
| hsa-miR-661 | hsa_circ_0003557 |
| hsa-miR-661 | hsa_circ_0000395 |
| hsa-miR-661 | hsa_circ_0002564 |
| hsa-miR-661 | hsa_circ_0002289 |
| hsa-miR-661 | hsa_circ_0033144 |
| hsa-miR-661 | hsa_circ_0007846 |
| hsa-miR-661 | hsa_circ_0000690 |
| hsa-miR-661 | hsa_circ_0046430 |
| hsa-miR-661 | hsa_circ_0048025 |
| hsa-miR-661 | hsa_circ_0049888 |
| hsa-miR-661 | hsa_circ_0000918 |
| hsa-miR-661 | hsa_circ_0061052 |
| hsa-miR-661 | hsa_circ_0002805 |
| hsa-miR-661 | hsa_circ_0092299 |
| hsa-miR-661 | hsa_circ_0092283 |
| hsa-miR-661 | hsa_circ_0004705 |
| hsa-miR-661 | hsa_circ_0003602 |
| hsa-miR-661 | hsa_circ_0092277 |
| hsa-miR-661 | hsa_circ_0006693 |
| hsa-miR-661 | hsa_circ_0001417 |
| hsa-miR-661 | hsa_circ_0007132 |
| hsa-miR-661 | hsa_circ_0005630 |
| hsa-miR-661 | hsa_circ_0001947 |
| hsa-miR-663a | hsa_circ_0007015 |
| hsa-miR-663a | hsa_circ_0018909 |
| hsa-miR-663a | hsa_circ_0003379 |
| hsa-miR-663a | hsa_circ_0022723 |
| hsa-miR-663a | hsa_circ_0007372 |
| hsa-miR-663a | hsa_circ_0000375 |
| hsa-miR-663a | hsa_circ_0000417 |
| hsa-miR-663a | hsa_circ_0029976 |
| hsa-miR-663a | hsa_circ_0040921 |
| hsa-miR-663a | hsa_circ_0092337 |
| hsa-miR-663a | hsa_circ_0045905 |
| hsa-miR-663a | hsa_circ_0046430 |
| hsa-miR-663a | hsa_circ_0048025 |
| hsa-miR-663a | hsa_circ_0003854 |
| hsa-miR-663a | hsa_circ_0002805 |
| hsa-miR-663a | hsa_circ_0061179 |
| hsa-miR-663a | hsa_circ_0001394 |
| hsa-miR-663a | hsa_circ_0001400 |
| hsa-miR-663a | hsa_circ_0071311 |
| hsa-miR-663a | hsa_circ_0001573 |
| hsa-miR-663a | hsa_circ_0007132 |
| hsa-miR-663a | hsa_circ_0001756 |
| hsa-miR-663a | hsa_circ_0001829 |
| hsa-miR-663a | hsa_circ_0087305 |
| hsa-miR-449b-5p | hsa_circ_0046430 |
| hsa-miR-449b-3p | hsa_circ_0003838 |
| hsa-miR-654-5p | hsa_circ_0076742 |
| hsa-miR-657 | hsa_circ_0006877 |
| hsa-miR-657 | hsa_circ_0092283 |
| hsa-miR-657 | hsa_circ_0001708 |
| hsa-miR-658 | hsa_circ_0009732 |
| hsa-miR-658 | hsa_circ_0007437 |
| hsa-miR-658 | hsa_circ_0007693 |
| hsa-miR-658 | hsa_circ_0007009 |
| hsa-miR-658 | hsa_circ_0017289 |
| hsa-miR-658 | hsa_circ_0005090 |
| hsa-miR-658 | hsa_circ_0000375 |
| hsa-miR-658 | hsa_circ_0000565 |
| hsa-miR-658 | hsa_circ_0048025 |
| hsa-miR-658 | hsa_circ_0049998 |
| hsa-miR-658 | hsa_circ_0003854 |
| hsa-miR-658 | hsa_circ_0004218 |
| hsa-miR-658 | hsa_circ_0061052 |
| hsa-miR-658 | hsa_circ_0002805 |
| hsa-miR-658 | hsa_circ_0061179 |
| hsa-miR-658 | hsa_circ_0062397 |
| hsa-miR-658 | hsa_circ_0071311 |
| hsa-miR-658 | hsa_circ_0072857 |
| hsa-miR-658 | hsa_circ_0003528 |
| hsa-miR-658 | hsa_circ_0007132 |
| hsa-miR-658 | hsa_circ_0004381 |
| hsa-miR-658 | hsa_circ_0005630 |
| hsa-miR-658 | hsa_circ_0087305 |
| hsa-miR-659-3p | hsa_circ_0007372 |
| hsa-miR-659-3p | hsa_circ_0000417 |
| hsa-miR-659-3p | hsa_circ_0040039 |
| hsa-miR-659-3p | hsa_circ_0004354 |
| hsa-miR-659-3p | hsa_circ_0092283 |
| hsa-miR-659-3p | hsa_circ_0003340 |
| hsa-miR-659-3p | hsa_circ_0084021 |
| hsa-miR-542-5p | hsa_circ_0043691 |
| hsa-miR-542-5p | hsa_circ_0002805 |
| hsa-miR-542-5p | hsa_circ_0061179 |
| hsa-miR-758-5p | hsa_circ_0001965 |
| hsa-miR-671-5p | hsa_circ_0002402 |
| hsa-miR-671-5p | hsa_circ_0033144 |
| hsa-miR-671-5p | hsa_circ_0007846 |
| hsa-miR-671-5p | hsa_circ_0040921 |
| hsa-miR-671-5p | hsa_circ_0045890 |
| hsa-miR-671-5p | hsa_circ_0046292 |
| hsa-miR-671-5p | hsa_circ_0046430 |
| hsa-miR-671-5p | hsa_circ_0004003 |
| hsa-miR-671-5p | hsa_circ_0002113 |
| hsa-miR-671-5p | hsa_circ_0092299 |
| hsa-miR-671-5p | hsa_circ_0001394 |
| hsa-miR-671-5p | hsa_circ_0001472 |
| hsa-miR-671-5p | hsa_circ_0007132 |
| hsa-miR-671-5p | hsa_circ_0075748 |
| hsa-miR-671-5p | hsa_circ_0087288 |
| hsa-miR-671-5p | hsa_circ_0092125 |
| hsa-miR-671-3p | hsa_circ_0003854 |
| hsa-miR-671-3p | hsa_circ_0007132 |
| hsa-miR-668-5p | hsa_circ_0001829 |
| hsa-miR-668-3p | hsa_circ_0009349 |
| hsa-miR-668-3p | hsa_circ_0045890 |
| hsa-miR-668-3p | hsa_circ_0002954 |
| hsa-miR-767-3p | hsa_circ_0004692 |
| hsa-miR-767-3p | hsa_circ_0065147 |
| hsa-miR-767-3p | hsa_circ_0065149 |
| hsa-miR-767-3p | hsa_circ_0002569 |
| hsa-miR-1224-5p | hsa_circ_0005371 |
| hsa-miR-1224-5p | hsa_circ_0092299 |
| hsa-miR-1224-5p | hsa_circ_0077765 |
| hsa-miR-1224-3p | hsa_circ_0020594 |
| hsa-miR-1224-3p | hsa_circ_0007001 |
| hsa-miR-1224-3p | hsa_circ_0022505 |
| hsa-miR-1224-3p | hsa_circ_0000996 |
| hsa-miR-1224-3p | hsa_circ_0006006 |
| hsa-miR-1224-3p | hsa_circ_0061936 |
| hsa-miR-1224-3p | hsa_circ_0092299 |
| hsa-miR-1224-3p | hsa_circ_0002094 |
| hsa-miR-1224-3p | hsa_circ_0002451 |
| hsa-miR-1224-3p | hsa_circ_0005630 |
| hsa-miR-1296-5p | hsa_circ_0020594 |
| hsa-miR-1296-5p | hsa_circ_0003768 |
| hsa-miR-1296-5p | hsa_circ_0002564 |
| hsa-miR-1296-5p | hsa_circ_0002289 |
| hsa-miR-1296-5p | hsa_circ_0000690 |
| hsa-miR-1296-5p | hsa_circ_0092299 |
| hsa-miR-1296-5p | hsa_circ_0092283 |
| hsa-miR-1296-3p | hsa_circ_0033144 |
| hsa-miR-1296-3p | hsa_circ_0043522 |
| hsa-miR-1296-3p | hsa_circ_0007132 |
| hsa-miR-1271-3p | hsa_circ_0033144 |
| hsa-miR-1301-3p | hsa_circ_0006649 |
| hsa-miR-1301-3p | hsa_circ_0008102 |
| hsa-miR-1301-3p | hsa_circ_0022723 |
| hsa-miR-1301-3p | hsa_circ_0003557 |
| hsa-miR-1301-3p | hsa_circ_0000462 |
| hsa-miR-1301-3p | hsa_circ_0031933 |
| hsa-miR-1301-3p | hsa_circ_0058058 |
| hsa-miR-1301-3p | hsa_circ_0058514 |
| hsa-miR-1301-3p | hsa_circ_0058520 |
| hsa-miR-1301-3p | hsa_circ_0058522 |
| hsa-miR-1301-3p | hsa_circ_0004692 |
| hsa-miR-1301-3p | hsa_circ_0065147 |
| hsa-miR-1301-3p | hsa_circ_0065149 |
| hsa-miR-1301-3p | hsa_circ_0002569 |
| hsa-miR-1301-3p | hsa_circ_0007132 |
| hsa-miR-449c-5p | hsa_circ_0040921 |
| hsa-miR-449c-5p | hsa_circ_0002094 |
| hsa-miR-769-3p | hsa_circ_0022383 |
| hsa-miR-769-3p | hsa_circ_0022723 |
| hsa-miR-769-3p | hsa_circ_0008916 |
| hsa-miR-769-3p | hsa_circ_0001955 |
| hsa-miR-769-3p | hsa_circ_0040921 |
| hsa-miR-769-3p | hsa_circ_0045890 |
| hsa-miR-769-3p | hsa_circ_0048025 |
| hsa-miR-769-3p | hsa_circ_0049657 |
| hsa-miR-769-3p | hsa_circ_0000950 |
| hsa-miR-769-3p | hsa_circ_0006006 |
| hsa-miR-769-3p | hsa_circ_0007132 |
| hsa-miR-769-3p | hsa_circ_0004381 |
| hsa-miR-769-3p | hsa_circ_0002094 |
| hsa-miR-769-3p | hsa_circ_0008113 |
| hsa-miR-769-3p | hsa_circ_0087305 |
| hsa-miR-766-3p | hsa_circ_0003854 |
| hsa-miR-766-3p | hsa_circ_0092283 |
| hsa-miR-766-3p | hsa_circ_0001329 |
| hsa-miR-766-3p | hsa_circ_0006884 |
| hsa-miR-766-3p | hsa_circ_0001756 |
| hsa-miR-762 | hsa_circ_0002402 |
| hsa-miR-762 | hsa_circ_0000247 |
| hsa-miR-762 | hsa_circ_0022383 |
| hsa-miR-762 | hsa_circ_0022392 |
| hsa-miR-762 | hsa_circ_0022723 |
| hsa-miR-762 | hsa_circ_0000375 |
| hsa-miR-762 | hsa_circ_0000417 |
| hsa-miR-762 | hsa_circ_0000497 |
| hsa-miR-762 | hsa_circ_0004137 |
| hsa-miR-762 | hsa_circ_0033144 |
| hsa-miR-762 | hsa_circ_0007146 |
| hsa-miR-762 | hsa_circ_0007846 |
| hsa-miR-762 | hsa_circ_0006735 |
| hsa-miR-762 | hsa_circ_0040921 |
| hsa-miR-762 | hsa_circ_0042799 |
| hsa-miR-762 | hsa_circ_0044195 |
| hsa-miR-762 | hsa_circ_0045890 |
| hsa-miR-762 | hsa_circ_0046430 |
| hsa-miR-762 | hsa_circ_0004003 |
| hsa-miR-762 | hsa_circ_0004891 |
| hsa-miR-762 | hsa_circ_0050119 |
| hsa-miR-762 | hsa_circ_0000918 |
| hsa-miR-762 | hsa_circ_0000950 |
| hsa-miR-762 | hsa_circ_0092297 |
| hsa-miR-762 | hsa_circ_0003854 |
| hsa-miR-762 | hsa_circ_0004218 |
| hsa-miR-762 | hsa_circ_0002805 |
| hsa-miR-762 | hsa_circ_0061179 |
| hsa-miR-762 | hsa_circ_0061936 |
| hsa-miR-762 | hsa_circ_0064136 |
| hsa-miR-762 | hsa_circ_0001394 |
| hsa-miR-762 | hsa_circ_0001400 |
| hsa-miR-762 | hsa_circ_0070467 |
| hsa-miR-762 | hsa_circ_0071311 |
| hsa-miR-762 | hsa_circ_0072797 |
| hsa-miR-762 | hsa_circ_0072857 |
| hsa-miR-762 | hsa_circ_0002490 |
| hsa-miR-762 | hsa_circ_0001549 |
| hsa-miR-762 | hsa_circ_0001573 |
| hsa-miR-762 | hsa_circ_0007132 |
| hsa-miR-762 | hsa_circ_0008536 |
| hsa-miR-762 | hsa_circ_0077765 |
| hsa-miR-762 | hsa_circ_0001658 |
| hsa-miR-762 | hsa_circ_0078522 |
| hsa-miR-762 | hsa_circ_0003340 |
| hsa-miR-762 | hsa_circ_0002755 |
| hsa-miR-762 | hsa_circ_0001756 |
| hsa-miR-762 | hsa_circ_0005630 |
| hsa-miR-762 | hsa_circ_0001829 |
| hsa-miR-762 | hsa_circ_0087288 |
| hsa-miR-762 | hsa_circ_0087305 |
| hsa-miR-762 | hsa_circ_0001866 |
| hsa-miR-762 | hsa_circ_0087855 |
| hsa-miR-762 | hsa_circ_0008812 |
| hsa-miR-762 | hsa_circ_0006174 |
| hsa-miR-762 | hsa_circ_0087861 |
| hsa-miR-762 | hsa_circ_0087862 |
| hsa-miR-1298-5p | hsa_circ_0007846 |
| hsa-miR-765 | hsa_circ_0069748 |
| hsa-miR-765 | hsa_circ_0072857 |
| hsa-miR-770-5p | hsa_circ_0017446 |
| hsa-miR-770-5p | hsa_circ_0000206 |
| hsa-miR-675-5p | hsa_circ_0007015 |
| hsa-miR-675-5p | hsa_circ_0000247 |
| hsa-miR-675-5p | hsa_circ_0022723 |
| hsa-miR-675-5p | hsa_circ_0007372 |
| hsa-miR-675-5p | hsa_circ_0002564 |
| hsa-miR-675-5p | hsa_circ_0002289 |
| hsa-miR-675-5p | hsa_circ_0036763 |
| hsa-miR-675-5p | hsa_circ_0000650 |
| hsa-miR-675-5p | hsa_circ_0000682 |
| hsa-miR-675-5p | hsa_circ_0045905 |
| hsa-miR-675-5p | hsa_circ_0071311 |
| hsa-miR-675-5p | hsa_circ_0005630 |
| hsa-miR-298 | hsa_circ_0045890 |
| hsa-miR-298 | hsa_circ_0002805 |
| hsa-miR-298 | hsa_circ_0061179 |
| hsa-miR-298 | hsa_circ_0004692 |
| hsa-miR-298 | hsa_circ_0084429 |
| hsa-miR-874-5p | hsa_circ_0000660 |
| hsa-miR-874-5p | hsa_circ_0092337 |
| hsa-miR-874-5p | hsa_circ_0003854 |
| hsa-miR-874-5p | hsa_circ_0004692 |
| hsa-miR-874-5p | hsa_circ_0065147 |
| hsa-miR-874-5p | hsa_circ_0065149 |
| hsa-miR-874-5p | hsa_circ_0002569 |
| hsa-miR-874-5p | hsa_circ_0001400 |
| hsa-miR-874-5p | hsa_circ_0081423 |
| hsa-miR-874-3p | hsa_circ_0014132 |
| hsa-miR-874-3p | hsa_circ_0003379 |
| hsa-miR-874-3p | hsa_circ_0025388 |
| hsa-miR-874-3p | hsa_circ_0007146 |
| hsa-miR-874-3p | hsa_circ_0007846 |
| hsa-miR-874-3p | hsa_circ_0092337 |
| hsa-miR-874-3p | hsa_circ_0008604 |
| hsa-miR-874-3p | hsa_circ_0045890 |
| hsa-miR-874-3p | hsa_circ_0050119 |
| hsa-miR-874-3p | hsa_circ_0003854 |
| hsa-miR-874-3p | hsa_circ_0007609 |
| hsa-miR-874-3p | hsa_circ_0092299 |
| hsa-miR-874-3p | hsa_circ_0004705 |
| hsa-miR-874-3p | hsa_circ_0004692 |
| hsa-miR-874-3p | hsa_circ_0065147 |
| hsa-miR-874-3p | hsa_circ_0065149 |
| hsa-miR-874-3p | hsa_circ_0002569 |
| hsa-miR-874-3p | hsa_circ_0072437 |
| hsa-miR-874-3p | hsa_circ_0007132 |
| hsa-miR-874-3p | hsa_circ_0081423 |
| hsa-miR-874-3p | hsa_circ_0092125 |
| hsa-miR-892b | hsa_circ_0029405 |
| hsa-miR-892b | hsa_circ_0031933 |
| hsa-miR-541-5p | hsa_circ_0016979 |
| hsa-miR-541-5p | hsa_circ_0007146 |
| hsa-miR-541-3p | hsa_circ_0000048 |
| hsa-miR-875-3p | hsa_circ_0002954 |
| hsa-miR-744-5p | hsa_circ_0022383 |
| hsa-miR-744-5p | hsa_circ_0002564 |
| hsa-miR-744-5p | hsa_circ_0002289 |
| hsa-miR-744-5p | hsa_circ_0006877 |
| hsa-miR-885-3p | hsa_circ_0042799 |
| hsa-miR-885-3p | hsa_circ_0049998 |
| hsa-miR-885-3p | hsa_circ_0002094 |
| hsa-miR-877-5p | hsa_circ_0002805 |
| hsa-miR-877-5p | hsa_circ_0061179 |
| hsa-miR-877-3p | hsa_circ_0020594 |
| hsa-miR-877-3p | hsa_circ_0002805 |
| hsa-miR-877-3p | hsa_circ_0004692 |
| hsa-miR-877-3p | hsa_circ_0065147 |
| hsa-miR-877-3p | hsa_circ_0065149 |
| hsa-miR-877-3p | hsa_circ_0002569 |
| hsa-miR-877-3p | hsa_circ_0007132 |
| hsa-miR-877-3p | hsa_circ_0007145 |
| hsa-miR-877-3p | hsa_circ_0002094 |
| hsa-miR-877-3p | hsa_circ_0005630 |
| hsa-miR-887-5p | hsa_circ_0001400 |
| hsa-miR-887-3p | hsa_circ_0046292 |
| hsa-miR-665 | hsa_circ_0007437 |
| hsa-miR-665 | hsa_circ_0007693 |
| hsa-miR-665 | hsa_circ_0012151 |
| hsa-miR-665 | hsa_circ_0007015 |
| hsa-miR-665 | hsa_circ_0022383 |
| hsa-miR-665 | hsa_circ_0000417 |
| hsa-miR-665 | hsa_circ_0092299 |
| hsa-miR-665 | hsa_circ_0001439 |
| hsa-miR-665 | hsa_circ_0084789 |
| hsa-miR-873-3p | hsa_circ_0003854 |
| hsa-miR-760 | hsa_circ_0003908 |
| hsa-miR-760 | hsa_circ_0000650 |
| hsa-miR-760 | hsa_circ_0036768 |
| hsa-miR-760 | hsa_circ_0040823 |
| hsa-miR-760 | hsa_circ_0046292 |
| hsa-miR-760 | hsa_circ_0046430 |
| hsa-miR-760 | hsa_circ_0048025 |
| hsa-miR-760 | hsa_circ_0050119 |
| hsa-miR-760 | hsa_circ_0072857 |
| hsa-miR-760 | hsa_circ_0004381 |
| hsa-miR-760 | hsa_circ_0087305 |
| hsa-miR-301b-5p | hsa_circ_0001971 |
| hsa-miR-301b-5p | hsa_circ_0005251 |
| hsa-miR-920 | hsa_circ_0017289 |
| hsa-miR-920 | hsa_circ_0005090 |
| hsa-miR-920 | hsa_circ_0006790 |
| hsa-miR-920 | hsa_circ_0007385 |
| hsa-miR-921 | hsa_circ_0011422 |
| hsa-miR-921 | hsa_circ_0016863 |
| hsa-miR-921 | hsa_circ_0016867 |
| hsa-miR-921 | hsa_circ_0071869 |
| hsa-miR-933 | hsa_circ_0000247 |
| hsa-miR-933 | hsa_circ_0045890 |
| hsa-miR-933 | hsa_circ_0047700 |
| hsa-miR-933 | hsa_circ_0007132 |
| hsa-miR-935 | hsa_circ_0000835 |
| hsa-miR-935 | hsa_circ_0000836 |
| hsa-miR-935 | hsa_circ_0005362 |
| hsa-miR-937-5p | hsa_circ_0020594 |
| hsa-miR-937-5p | hsa_circ_0000417 |
| hsa-miR-937-5p | hsa_circ_0007846 |
| hsa-miR-937-5p | hsa_circ_0092337 |
| hsa-miR-937-5p | hsa_circ_0092299 |
| hsa-miR-937-5p | hsa_circ_0001549 |
| hsa-miR-937-3p | hsa_circ_0061936 |
| hsa-miR-938 | hsa_circ_0025388 |
| hsa-miR-939-5p | hsa_circ_0009349 |
| hsa-miR-939-5p | hsa_circ_0009357 |
| hsa-miR-939-5p | hsa_circ_0009360 |
| hsa-miR-939-5p | hsa_circ_0009581 |
| hsa-miR-939-5p | hsa_circ_0006837 |
| hsa-miR-939-5p | hsa_circ_0002402 |
| hsa-miR-939-5p | hsa_circ_0015004 |
| hsa-miR-939-5p | hsa_circ_0007015 |
| hsa-miR-939-5p | hsa_circ_0016863 |
| hsa-miR-939-5p | hsa_circ_0016867 |
| hsa-miR-939-5p | hsa_circ_0017289 |
| hsa-miR-939-5p | hsa_circ_0005090 |
| hsa-miR-939-5p | hsa_circ_0000206 |
| hsa-miR-939-5p | hsa_circ_0017461 |
| hsa-miR-939-5p | hsa_circ_0006608 |
| hsa-miR-939-5p | hsa_circ_0006649 |
| hsa-miR-939-5p | hsa_circ_0006254 |
| hsa-miR-939-5p | hsa_circ_0003768 |
| hsa-miR-939-5p | hsa_circ_0000375 |
| hsa-miR-939-5p | hsa_circ_0000417 |
| hsa-miR-939-5p | hsa_circ_0029405 |
| hsa-miR-939-5p | hsa_circ_0000497 |
| hsa-miR-939-5p | hsa_circ_0031933 |
| hsa-miR-939-5p | hsa_circ_0002564 |
| hsa-miR-939-5p | hsa_circ_0002289 |
| hsa-miR-939-5p | hsa_circ_0033144 |
| hsa-miR-939-5p | hsa_circ_0034972 |
| hsa-miR-939-5p | hsa_circ_0036763 |
| hsa-miR-939-5p | hsa_circ_0000650 |
| hsa-miR-939-5p | hsa_circ_0007846 |
| hsa-miR-939-5p | hsa_circ_0040823 |
| hsa-miR-939-5p | hsa_circ_0040921 |
| hsa-miR-939-5p | hsa_circ_0043691 |
| hsa-miR-939-5p | hsa_circ_0045890 |
| hsa-miR-939-5p | hsa_circ_0045905 |
| hsa-miR-939-5p | hsa_circ_0046430 |
| hsa-miR-939-5p | hsa_circ_0048025 |
| hsa-miR-939-5p | hsa_circ_0004003 |
| hsa-miR-939-5p | hsa_circ_0000936 |
| hsa-miR-939-5p | hsa_circ_0000973 |
| hsa-miR-939-5p | hsa_circ_0054086 |
| hsa-miR-939-5p | hsa_circ_0055904 |
| hsa-miR-939-5p | hsa_circ_0092297 |
| hsa-miR-939-5p | hsa_circ_0002377 |
| hsa-miR-939-5p | hsa_circ_0004218 |
| hsa-miR-939-5p | hsa_circ_0002113 |
| hsa-miR-939-5p | hsa_circ_0092299 |
| hsa-miR-939-5p | hsa_circ_0092283 |
| hsa-miR-939-5p | hsa_circ_0004692 |
| hsa-miR-939-5p | hsa_circ_0003602 |
| hsa-miR-939-5p | hsa_circ_0092277 |
| hsa-miR-939-5p | hsa_circ_0001472 |
| hsa-miR-939-5p | hsa_circ_0074264 |
| hsa-miR-939-5p | hsa_circ_0001573 |
| hsa-miR-939-5p | hsa_circ_0007132 |
| hsa-miR-939-5p | hsa_circ_0003340 |
| hsa-miR-939-5p | hsa_circ_0002755 |
| hsa-miR-939-5p | hsa_circ_0005630 |
| hsa-miR-939-5p | hsa_circ_0084429 |
| hsa-miR-939-5p | hsa_circ_0084789 |
| hsa-miR-939-5p | hsa_circ_0006566 |
| hsa-miR-939-5p | hsa_circ_0087288 |
| hsa-miR-939-5p | hsa_circ_0087855 |
| hsa-miR-939-5p | hsa_circ_0008812 |
| hsa-miR-939-5p | hsa_circ_0006174 |
| hsa-miR-939-5p | hsa_circ_0087861 |
| hsa-miR-939-5p | hsa_circ_0087862 |
| hsa-miR-939-5p | hsa_circ_0092125 |
| hsa-miR-939-3p | hsa_circ_0009732 |
| hsa-miR-939-3p | hsa_circ_0011571 |
| hsa-miR-939-3p | hsa_circ_0011572 |
| hsa-miR-939-3p | hsa_circ_0000690 |
| hsa-miR-939-3p | hsa_circ_0046430 |
| hsa-miR-939-3p | hsa_circ_0007967 |
| hsa-miR-939-3p | hsa_circ_0002805 |
| hsa-miR-939-3p | hsa_circ_0061179 |
| hsa-miR-939-3p | hsa_circ_0061936 |
| hsa-miR-939-3p | hsa_circ_0092283 |
| hsa-miR-939-3p | hsa_circ_0092277 |
| hsa-miR-939-3p | hsa_circ_0001400 |
| hsa-miR-939-3p | hsa_circ_0007132 |
| hsa-miR-939-3p | hsa_circ_0008113 |
| hsa-miR-940 | hsa_circ_0000690 |
| hsa-miR-940 | hsa_circ_0092283 |
| hsa-miR-940 | hsa_circ_0004705 |
| hsa-miR-940 | hsa_circ_0001910 |
| hsa-miR-941 | hsa_circ_0084984 |
| hsa-miR-1180-5p | hsa_circ_0003379 |
| hsa-miR-1180-5p | hsa_circ_0049998 |
| hsa-miR-1180-5p | hsa_circ_0003854 |
| hsa-miR-1180-5p | hsa_circ_0092299 |
| hsa-miR-1180-3p | hsa_circ_0031933 |
| hsa-miR-1180-3p | hsa_circ_0040921 |
| hsa-miR-1180-3p | hsa_circ_0001394 |
| hsa-miR-1181 | hsa_circ_0033144 |
| hsa-miR-1181 | hsa_circ_0034920 |
| hsa-miR-1181 | hsa_circ_0005362 |
| hsa-miR-1181 | hsa_circ_0006693 |
| hsa-miR-1181 | hsa_circ_0001417 |
| hsa-miR-1181 | hsa_circ_0007132 |
| hsa-miR-1181 | hsa_circ_0002094 |
| hsa-miR-1181 | hsa_circ_0008113 |
| hsa-miR-1182 | hsa_circ_0006254 |
| hsa-miR-1182 | hsa_circ_0003768 |
| hsa-miR-1182 | hsa_circ_0007372 |
| hsa-miR-1182 | hsa_circ_0000417 |
| hsa-miR-1182 | hsa_circ_0000437 |
| hsa-miR-1182 | hsa_circ_0004372 |
| hsa-miR-1182 | hsa_circ_0003838 |
| hsa-miR-1182 | hsa_circ_0002805 |
| hsa-miR-1182 | hsa_circ_0092277 |
| hsa-miR-1182 | hsa_circ_0001400 |
| hsa-miR-1182 | hsa_circ_0070467 |
| hsa-miR-1182 | hsa_circ_0077765 |
| hsa-miR-1182 | hsa_circ_0001756 |
| hsa-miR-1182 | hsa_circ_0002094 |
| hsa-miR-1182 | hsa_circ_0092125 |
| hsa-miR-1183 | hsa_circ_0011422 |
| hsa-miR-1183 | hsa_circ_0005664 |
| hsa-miR-1183 | hsa_circ_0000247 |
| hsa-miR-1183 | hsa_circ_0018909 |
| hsa-miR-1183 | hsa_circ_0006254 |
| hsa-miR-1183 | hsa_circ_0003768 |
| hsa-miR-1183 | hsa_circ_0046430 |
| hsa-miR-1183 | hsa_circ_0002926 |
| hsa-miR-1183 | hsa_circ_0049998 |
| hsa-miR-1183 | hsa_circ_0092297 |
| hsa-miR-1183 | hsa_circ_0003854 |
| hsa-miR-1183 | hsa_circ_0002805 |
| hsa-miR-1183 | hsa_circ_0061179 |
| hsa-miR-1183 | hsa_circ_0070467 |
| hsa-miR-1183 | hsa_circ_0003528 |
| hsa-miR-1183 | hsa_circ_0007132 |
| hsa-miR-1183 | hsa_circ_0003340 |
| hsa-miR-1183 | hsa_circ_0081343 |
| hsa-miR-1183 | hsa_circ_0084429 |
| hsa-miR-1183 | hsa_circ_0084552 |
| hsa-miR-1183 | hsa_circ_0001811 |
| hsa-miR-1183 | hsa_circ_0087288 |
| hsa-miR-1184 | hsa_circ_0007846 |
| hsa-miR-1184 | hsa_circ_0092299 |
| hsa-miR-1184 | hsa_circ_0007132 |
| hsa-miR-1225-5p | hsa_circ_0002402 |
| hsa-miR-1225-5p | hsa_circ_0003379 |
| hsa-miR-1225-5p | hsa_circ_0000437 |
| hsa-miR-1225-5p | hsa_circ_0031933 |
| hsa-miR-1225-5p | hsa_circ_0046430 |
| hsa-miR-1225-5p | hsa_circ_0002805 |
| hsa-miR-1225-5p | hsa_circ_0066444 |
| hsa-miR-1225-5p | hsa_circ_0001577 |
| hsa-miR-1225-5p | hsa_circ_0001578 |
| hsa-miR-1225-5p | hsa_circ_0076742 |
| hsa-miR-1225-5p | hsa_circ_0001658 |
| hsa-miR-1225-5p | hsa_circ_0003340 |
| hsa-miR-1225-3p | hsa_circ_0022723 |
| hsa-miR-1225-3p | hsa_circ_0000395 |
| hsa-miR-1225-3p | hsa_circ_0007146 |
| hsa-miR-1225-3p | hsa_circ_0001400 |
| hsa-miR-1225-3p | hsa_circ_0006693 |
| hsa-miR-1225-3p | hsa_circ_0001417 |
| hsa-miR-1225-3p | hsa_circ_0070396 |
| hsa-miR-1225-3p | hsa_circ_0007132 |
| hsa-miR-1225-3p | hsa_circ_0008113 |
| hsa-miR-1226-5p | hsa_circ_0009349 |
| hsa-miR-1226-5p | hsa_circ_0009581 |
| hsa-miR-1226-5p | hsa_circ_0006837 |
| hsa-miR-1226-5p | hsa_circ_0002402 |
| hsa-miR-1226-5p | hsa_circ_0007015 |
| hsa-miR-1226-5p | hsa_circ_0016979 |
| hsa-miR-1226-5p | hsa_circ_0006649 |
| hsa-miR-1226-5p | hsa_circ_0020005 |
| hsa-miR-1226-5p | hsa_circ_0003379 |
| hsa-miR-1226-5p | hsa_circ_0006254 |
| hsa-miR-1226-5p | hsa_circ_0003768 |
| hsa-miR-1226-5p | hsa_circ_0007001 |
| hsa-miR-1226-5p | hsa_circ_0022383 |
| hsa-miR-1226-5p | hsa_circ_0022392 |
| hsa-miR-1226-5p | hsa_circ_0003557 |
| hsa-miR-1226-5p | hsa_circ_0007372 |
| hsa-miR-1226-5p | hsa_circ_0000375 |
| hsa-miR-1226-5p | hsa_circ_0003855 |
| hsa-miR-1226-5p | hsa_circ_0028190 |
| hsa-miR-1226-5p | hsa_circ_0035381 |
| hsa-miR-1226-5p | hsa_circ_0000651 |
| hsa-miR-1226-5p | hsa_circ_0007846 |
| hsa-miR-1226-5p | hsa_circ_0000682 |
| hsa-miR-1226-5p | hsa_circ_0044195 |
| hsa-miR-1226-5p | hsa_circ_0045890 |
| hsa-miR-1226-5p | hsa_circ_0046430 |
| hsa-miR-1226-5p | hsa_circ_0000836 |
| hsa-miR-1226-5p | hsa_circ_0002926 |
| hsa-miR-1226-5p | hsa_circ_0000936 |
| hsa-miR-1226-5p | hsa_circ_0000973 |
| hsa-miR-1226-5p | hsa_circ_0008261 |
| hsa-miR-1226-5p | hsa_circ_0061052 |
| hsa-miR-1226-5p | hsa_circ_0002805 |
| hsa-miR-1226-5p | hsa_circ_0061179 |
| hsa-miR-1226-5p | hsa_circ_0004705 |
| hsa-miR-1226-5p | hsa_circ_0092277 |
| hsa-miR-1226-5p | hsa_circ_0006884 |
| hsa-miR-1226-5p | hsa_circ_0001394 |
| hsa-miR-1226-5p | hsa_circ_0001400 |
| hsa-miR-1226-5p | hsa_circ_0069748 |
| hsa-miR-1226-5p | hsa_circ_0070467 |
| hsa-miR-1226-5p | hsa_circ_0071311 |
| hsa-miR-1226-5p | hsa_circ_0001472 |
| hsa-miR-1226-5p | hsa_circ_0073379 |
| hsa-miR-1226-5p | hsa_circ_0007132 |
| hsa-miR-1226-5p | hsa_circ_0004712 |
| hsa-miR-1226-5p | hsa_circ_0007145 |
| hsa-miR-1226-5p | hsa_circ_0082179 |
| hsa-miR-1226-5p | hsa_circ_0001756 |
| hsa-miR-1226-5p | hsa_circ_0002094 |
| hsa-miR-1226-5p | hsa_circ_0084429 |
| hsa-miR-1226-5p | hsa_circ_0008934 |
| hsa-miR-1226-5p | hsa_circ_0007521 |
| hsa-miR-1226-5p | hsa_circ_0005982 |
| hsa-miR-1226-5p | hsa_circ_0003221 |
| hsa-miR-1226-5p | hsa_circ_0002483 |
| hsa-miR-1226-5p | hsa_circ_0006646 |
| hsa-miR-1226-5p | hsa_circ_0087305 |
| hsa-miR-1226-5p | hsa_circ_0087855 |
| hsa-miR-1226-5p | hsa_circ_0008812 |
| hsa-miR-1226-5p | hsa_circ_0006174 |
| hsa-miR-1226-5p | hsa_circ_0087861 |
| hsa-miR-1226-5p | hsa_circ_0087862 |
| hsa-miR-1226-5p | hsa_circ_0001947 |
| hsa-miR-1226-5p | hsa_circ_0092125 |
| hsa-miR-1226-3p | hsa_circ_0007146 |
| hsa-miR-1226-3p | hsa_circ_0050119 |
| hsa-miR-1226-3p | hsa_circ_0008583 |
| hsa-miR-1227-3p | hsa_circ_0045890 |
| hsa-miR-1228-5p | hsa_circ_0009349 |
| hsa-miR-1228-5p | hsa_circ_0003557 |
| hsa-miR-1228-5p | hsa_circ_0000375 |
| hsa-miR-1228-5p | hsa_circ_0007846 |
| hsa-miR-1228-5p | hsa_circ_0046430 |
| hsa-miR-1228-5p | hsa_circ_0048025 |
| hsa-miR-1228-5p | hsa_circ_0006877 |
| hsa-miR-1228-5p | hsa_circ_0008030 |
| hsa-miR-1228-5p | hsa_circ_0052131 |
| hsa-miR-1228-5p | hsa_circ_0092297 |
| hsa-miR-1228-5p | hsa_circ_0003854 |
| hsa-miR-1228-5p | hsa_circ_0001134 |
| hsa-miR-1228-5p | hsa_circ_0004218 |
| hsa-miR-1228-5p | hsa_circ_0061936 |
| hsa-miR-1228-5p | hsa_circ_0070467 |
| hsa-miR-1228-5p | hsa_circ_0074816 |
| hsa-miR-1228-5p | hsa_circ_0074817 |
| hsa-miR-1228-5p | hsa_circ_0007132 |
| hsa-miR-1228-5p | hsa_circ_0002094 |
| hsa-miR-1228-5p | hsa_circ_0005630 |
| hsa-miR-1228-5p | hsa_circ_0084789 |
| hsa-miR-1228-5p | hsa_circ_0087305 |
| hsa-miR-1228-3p | hsa_circ_0000130 |
| hsa-miR-1228-3p | hsa_circ_0000131 |
| hsa-miR-1229-5p | hsa_circ_0016979 |
| hsa-miR-1229-5p | hsa_circ_0022383 |
| hsa-miR-1229-5p | hsa_circ_0023918 |
| hsa-miR-1229-5p | hsa_circ_0023920 |
| hsa-miR-1229-5p | hsa_circ_0023923 |
| hsa-miR-1229-5p | hsa_circ_0007372 |
| hsa-miR-1229-5p | hsa_circ_0000375 |
| hsa-miR-1229-5p | hsa_circ_0003855 |
| hsa-miR-1229-5p | hsa_circ_0000417 |
| hsa-miR-1229-5p | hsa_circ_0031933 |
| hsa-miR-1229-5p | hsa_circ_0003838 |
| hsa-miR-1229-5p | hsa_circ_0045890 |
| hsa-miR-1229-5p | hsa_circ_0006877 |
| hsa-miR-1229-5p | hsa_circ_0055904 |
| hsa-miR-1229-5p | hsa_circ_0003854 |
| hsa-miR-1229-5p | hsa_circ_0004218 |
| hsa-miR-1229-5p | hsa_circ_0061052 |
| hsa-miR-1229-5p | hsa_circ_0002805 |
| hsa-miR-1229-5p | hsa_circ_0092299 |
| hsa-miR-1229-5p | hsa_circ_0064136 |
| hsa-miR-1229-5p | hsa_circ_0092277 |
| hsa-miR-1229-5p | hsa_circ_0001394 |
| hsa-miR-1229-5p | hsa_circ_0069748 |
| hsa-miR-1229-5p | hsa_circ_0070467 |
| hsa-miR-1229-5p | hsa_circ_0001549 |
| hsa-miR-1229-5p | hsa_circ_0001573 |
| hsa-miR-1229-5p | hsa_circ_0004349 |
| hsa-miR-1229-5p | hsa_circ_0009092 |
| hsa-miR-1229-5p | hsa_circ_0001708 |
| hsa-miR-1229-5p | hsa_circ_0005630 |
| hsa-miR-1229-5p | hsa_circ_0001806 |
| hsa-miR-1229-3p | hsa_circ_0011571 |
| hsa-miR-1229-3p | hsa_circ_0011572 |
| hsa-miR-1229-3p | hsa_circ_0020594 |
| hsa-miR-1229-3p | hsa_circ_0003557 |
| hsa-miR-1229-3p | hsa_circ_0000973 |
| hsa-miR-1229-3p | hsa_circ_0007132 |
| hsa-miR-1229-3p | hsa_circ_0002094 |
| hsa-miR-1231 | hsa_circ_0002037 |
| hsa-miR-1233-5p | hsa_circ_0007015 |
| hsa-miR-1233-5p | hsa_circ_0003379 |
| hsa-miR-1233-5p | hsa_circ_0007846 |
| hsa-miR-1233-5p | hsa_circ_0040921 |
| hsa-miR-1233-5p | hsa_circ_0048025 |
| hsa-miR-1233-5p | hsa_circ_0000973 |
| hsa-miR-1233-5p | hsa_circ_0003854 |
| hsa-miR-1233-5p | hsa_circ_0004218 |
| hsa-miR-1233-5p | hsa_circ_0004692 |
| hsa-miR-1233-5p | hsa_circ_0065147 |
| hsa-miR-1233-5p | hsa_circ_0065149 |
| hsa-miR-1233-5p | hsa_circ_0002569 |
| hsa-miR-1233-5p | hsa_circ_0001400 |
| hsa-miR-1233-5p | hsa_circ_0007132 |
| hsa-miR-1233-5p | hsa_circ_0002094 |
| hsa-miR-1233-5p | hsa_circ_0001829 |
| hsa-miR-1233-5p | hsa_circ_0087288 |
| hsa-miR-1233-5p | hsa_circ_0087855 |
| hsa-miR-1233-5p | hsa_circ_0008812 |
| hsa-miR-1233-5p | hsa_circ_0006174 |
| hsa-miR-1233-5p | hsa_circ_0087861 |
| hsa-miR-1233-5p | hsa_circ_0087862 |
| hsa-miR-1233-3p | hsa_circ_0000417 |
| hsa-miR-1233-3p | hsa_circ_0050119 |
| hsa-miR-1233-3p | hsa_circ_0001708 |
| hsa-miR-1234-3p | hsa_circ_0016979 |
| hsa-miR-1234-3p | hsa_circ_0020594 |
| hsa-miR-1234-3p | hsa_circ_0003557 |
| hsa-miR-1234-3p | hsa_circ_0031933 |
| hsa-miR-1234-3p | hsa_circ_0033144 |
| hsa-miR-1234-3p | hsa_circ_0092277 |
| hsa-miR-1234-3p | hsa_circ_0007132 |
| hsa-miR-1236-5p | hsa_circ_0004705 |
| hsa-miR-1236-3p | hsa_circ_0020594 |
| hsa-miR-1236-3p | hsa_circ_0001492 |
| hsa-miR-1236-3p | hsa_circ_0007145 |
| hsa-miR-1237-5p | hsa_circ_0009349 |
| hsa-miR-1237-5p | hsa_circ_0009357 |
| hsa-miR-1237-5p | hsa_circ_0009360 |
| hsa-miR-1237-5p | hsa_circ_0006649 |
| hsa-miR-1237-5p | hsa_circ_0006254 |
| hsa-miR-1237-5p | hsa_circ_0003768 |
| hsa-miR-1237-5p | hsa_circ_0000375 |
| hsa-miR-1237-5p | hsa_circ_0000660 |
| hsa-miR-1237-5p | hsa_circ_0007146 |
| hsa-miR-1237-5p | hsa_circ_0045890 |
| hsa-miR-1237-5p | hsa_circ_0002926 |
| hsa-miR-1237-5p | hsa_circ_0050119 |
| hsa-miR-1237-5p | hsa_circ_0052131 |
| hsa-miR-1237-5p | hsa_circ_0003854 |
| hsa-miR-1237-5p | hsa_circ_0004218 |
| hsa-miR-1237-5p | hsa_circ_0092299 |
| hsa-miR-1237-5p | hsa_circ_0064136 |
| hsa-miR-1237-5p | hsa_circ_0004705 |
| hsa-miR-1237-5p | hsa_circ_0007132 |
| hsa-miR-1237-5p | hsa_circ_0002094 |
| hsa-miR-1237-5p | hsa_circ_0005630 |
| hsa-miR-1237-5p | hsa_circ_0001829 |
| hsa-miR-1237-5p | hsa_circ_0087288 |
| hsa-miR-1237-3p | hsa_circ_0020594 |
| hsa-miR-1237-3p | hsa_circ_0004692 |
| hsa-miR-1237-3p | hsa_circ_0065147 |
| hsa-miR-1237-3p | hsa_circ_0065149 |
| hsa-miR-1237-3p | hsa_circ_0002569 |
| hsa-miR-1237-3p | hsa_circ_0001400 |
| hsa-miR-1237-3p | hsa_circ_0002094 |
| hsa-miR-1237-3p | hsa_circ_0005630 |
| hsa-miR-1237-3p | hsa_circ_0008113 |
| hsa-miR-1238-5p | hsa_circ_0018909 |
| hsa-miR-1238-5p | hsa_circ_0000497 |
| hsa-miR-1238-5p | hsa_circ_0045890 |
| hsa-miR-1238-5p | hsa_circ_0001573 |
| hsa-miR-1238-3p | hsa_circ_0002094 |
| hsa-miR-1238-3p | hsa_circ_0008113 |
| hsa-miR-1202 | hsa_circ_0000206 |
| hsa-miR-1202 | hsa_circ_0017461 |
| hsa-miR-1202 | hsa_circ_0000417 |
| hsa-miR-1202 | hsa_circ_0031933 |
| hsa-miR-1202 | hsa_circ_0007146 |
| hsa-miR-1202 | hsa_circ_0049657 |
| hsa-miR-1202 | hsa_circ_0002805 |
| hsa-miR-1202 | hsa_circ_0061179 |
| hsa-miR-1203 | hsa_circ_0003379 |
| hsa-miR-1203 | hsa_circ_0040039 |
| hsa-miR-1203 | hsa_circ_0004354 |
| hsa-miR-1203 | hsa_circ_0070396 |
| hsa-miR-1203 | hsa_circ_0005630 |
| hsa-miR-1203 | hsa_circ_0087305 |
| hsa-miR-663b | hsa_circ_0006649 |
| hsa-miR-663b | hsa_circ_0020594 |
| hsa-miR-663b | hsa_circ_0003557 |
| hsa-miR-663b | hsa_circ_0000375 |
| hsa-miR-663b | hsa_circ_0000417 |
| hsa-miR-663b | hsa_circ_0028190 |
| hsa-miR-663b | hsa_circ_0029405 |
| hsa-miR-663b | hsa_circ_0033144 |
| hsa-miR-663b | hsa_circ_0007846 |
| hsa-miR-663b | hsa_circ_0045890 |
| hsa-miR-663b | hsa_circ_0046430 |
| hsa-miR-663b | hsa_circ_0002926 |
| hsa-miR-663b | hsa_circ_0050119 |
| hsa-miR-663b | hsa_circ_0092297 |
| hsa-miR-663b | hsa_circ_0092283 |
| hsa-miR-663b | hsa_circ_0004470 |
| hsa-miR-663b | hsa_circ_0092277 |
| hsa-miR-663b | hsa_circ_0001400 |
| hsa-miR-663b | hsa_circ_0007132 |
| hsa-miR-663b | hsa_circ_0081343 |
| hsa-miR-663b | hsa_circ_0087288 |
| hsa-miR-1207-5p | hsa_circ_0011422 |
| hsa-miR-1207-5p | hsa_circ_0007437 |
| hsa-miR-1207-5p | hsa_circ_0007693 |
| hsa-miR-1207-5p | hsa_circ_0016863 |
| hsa-miR-1207-5p | hsa_circ_0017248 |
| hsa-miR-1207-5p | hsa_circ_0003379 |
| hsa-miR-1207-5p | hsa_circ_0022383 |
| hsa-miR-1207-5p | hsa_circ_0000437 |
| hsa-miR-1207-5p | hsa_circ_0028190 |
| hsa-miR-1207-5p | hsa_circ_0000497 |
| hsa-miR-1207-5p | hsa_circ_0007846 |
| hsa-miR-1207-5p | hsa_circ_0045890 |
| hsa-miR-1207-5p | hsa_circ_0046430 |
| hsa-miR-1207-5p | hsa_circ_0004218 |
| hsa-miR-1207-5p | hsa_circ_0001394 |
| hsa-miR-1207-5p | hsa_circ_0071311 |
| hsa-miR-1207-5p | hsa_circ_0071375 |
| hsa-miR-1207-5p | hsa_circ_0001549 |
| hsa-miR-1207-5p | hsa_circ_0001577 |
| hsa-miR-1207-5p | hsa_circ_0001578 |
| hsa-miR-1207-5p | hsa_circ_0007132 |
| hsa-miR-1207-5p | hsa_circ_0003738 |
| hsa-miR-1207-5p | hsa_circ_0076742 |
| hsa-miR-1207-5p | hsa_circ_0002094 |
| hsa-miR-1207-5p | hsa_circ_0087288 |
| hsa-miR-1285-5p | hsa_circ_0020594 |
| hsa-miR-1285-5p | hsa_circ_0000973 |
| hsa-miR-1285-3p | hsa_circ_0029405 |
| hsa-miR-1291 | hsa_circ_0000437 |
| hsa-miR-1291 | hsa_circ_0033144 |
| hsa-miR-1291 | hsa_circ_0007146 |
| hsa-miR-1291 | hsa_circ_0008261 |
| hsa-miR-1291 | hsa_circ_0092297 |
| hsa-miR-1291 | hsa_circ_0058058 |
| hsa-miR-1291 | hsa_circ_0002805 |
| hsa-miR-1291 | hsa_circ_0007137 |
| hsa-miR-1293 | hsa_circ_0002926 |
| hsa-miR-1293 | hsa_circ_0049998 |
| hsa-miR-1293 | hsa_circ_0001187 |
| hsa-miR-1293 | hsa_circ_0008078 |
| hsa-miR-1293 | hsa_circ_0070396 |
| hsa-miR-1293 | hsa_circ_0007132 |
| hsa-miR-1293 | hsa_circ_0084615 |
| hsa-miR-1294 | hsa_circ_0060904 |
| hsa-miR-1299 | hsa_circ_0017639 |
| hsa-miR-1303 | hsa_circ_0020594 |
| hsa-miR-1303 | hsa_circ_0000973 |
| hsa-miR-1304-3p | hsa_circ_0000973 |
| hsa-miR-1247-5p | hsa_circ_0022723 |
| hsa-miR-1247-5p | hsa_circ_0041050 |
| hsa-miR-1247-5p | hsa_circ_0045890 |
| hsa-miR-1247-5p | hsa_circ_0049657 |
| hsa-miR-1247-5p | hsa_circ_0000996 |
| hsa-miR-1247-5p | hsa_circ_0092299 |
| hsa-miR-1247-5p | hsa_circ_0070396 |
| hsa-miR-1247-5p | hsa_circ_0074816 |
| hsa-miR-1247-5p | hsa_circ_0074817 |
| hsa-miR-1247-5p | hsa_circ_0007132 |
| hsa-miR-1247-5p | hsa_circ_0005630 |
| hsa-miR-1247-5p | hsa_circ_0007685 |
| hsa-miR-1247-5p | hsa_circ_0001910 |
| hsa-miR-1247-3p | hsa_circ_0022723 |
| hsa-miR-1247-3p | hsa_circ_0049657 |
| hsa-miR-1247-3p | hsa_circ_0049998 |
| hsa-miR-1247-3p | hsa_circ_0000918 |
| hsa-miR-1247-3p | hsa_circ_0060904 |
| hsa-miR-1247-3p | hsa_circ_0001394 |
| hsa-miR-1247-3p | hsa_circ_0003528 |
| hsa-miR-1247-3p | hsa_circ_0004381 |
| hsa-miR-1249-5p | hsa_circ_0017248 |
| hsa-miR-1249-5p | hsa_circ_0000339 |
| hsa-miR-1249-5p | hsa_circ_0003908 |
| hsa-miR-1249-5p | hsa_circ_0004137 |
| hsa-miR-1249-5p | hsa_circ_0007846 |
| hsa-miR-1249-5p | hsa_circ_0044195 |
| hsa-miR-1249-5p | hsa_circ_0045890 |
| hsa-miR-1249-5p | hsa_circ_0049657 |
| hsa-miR-1249-5p | hsa_circ_0000936 |
| hsa-miR-1249-5p | hsa_circ_0002805 |
| hsa-miR-1249-5p | hsa_circ_0061179 |
| hsa-miR-1249-5p | hsa_circ_0092299 |
| hsa-miR-1249-5p | hsa_circ_0063331 |
| hsa-miR-1249-5p | hsa_circ_0001400 |
| hsa-miR-1249-5p | hsa_circ_0007132 |
| hsa-miR-1249-5p | hsa_circ_0004381 |
| hsa-miR-1249-5p | hsa_circ_0001756 |
| hsa-miR-1249-3p | hsa_circ_0020594 |
| hsa-miR-1249-3p | hsa_circ_0092299 |
| hsa-miR-1249-3p | hsa_circ_0007132 |
| hsa-miR-1254 | hsa_circ_0007015 |
| hsa-miR-1254 | hsa_circ_0029405 |
| hsa-miR-1254 | hsa_circ_0045890 |
| hsa-miR-1254 | hsa_circ_0000936 |
| hsa-miR-1254 | hsa_circ_0004400 |
| hsa-miR-1254 | hsa_circ_0007132 |
| hsa-miR-1254 | hsa_circ_0075748 |
| hsa-miR-1254 | hsa_circ_0082179 |
| hsa-miR-1254 | hsa_circ_0005630 |
| hsa-miR-1266-5p | hsa_circ_0022392 |
| hsa-miR-1266-5p | hsa_circ_0007846 |
| hsa-miR-1266-5p | hsa_circ_0041050 |
| hsa-miR-1266-5p | hsa_circ_0092297 |
| hsa-miR-1266-5p | hsa_circ_0005630 |
| hsa-miR-1266-3p | hsa_circ_0007001 |
| hsa-miR-1266-3p | hsa_circ_0007372 |
| hsa-miR-1266-3p | hsa_circ_0034189 |
| hsa-miR-1266-3p | hsa_circ_0050119 |
| hsa-miR-1266-3p | hsa_circ_0092283 |
| hsa-miR-1266-3p | hsa_circ_0006552 |
| hsa-miR-1268a | hsa_circ_0022383 |
| hsa-miR-1268a | hsa_circ_0000417 |
| hsa-miR-1268a | hsa_circ_0029405 |
| hsa-miR-1268a | hsa_circ_0048025 |
| hsa-miR-1268a | hsa_circ_0000973 |
| hsa-miR-1268a | hsa_circ_0002805 |
| hsa-miR-1268a | hsa_circ_0061179 |
| hsa-miR-1268a | hsa_circ_0070467 |
| hsa-miR-1268a | hsa_circ_0007132 |
| hsa-miR-1268a | hsa_circ_0002755 |
| hsa-miR-1268a | hsa_circ_0001748 |
| hsa-miR-1268a | hsa_circ_0002094 |
| hsa-miR-1268a | hsa_circ_0005630 |
| hsa-miR-1268a | hsa_circ_0087305 |
| hsa-miR-1268a | hsa_circ_0087855 |
| hsa-miR-1268a | hsa_circ_0008812 |
| hsa-miR-1268a | hsa_circ_0006174 |
| hsa-miR-1268a | hsa_circ_0087861 |
| hsa-miR-1268a | hsa_circ_0087862 |
| hsa-miR-1269a | hsa_circ_0034189 |
| hsa-miR-1269a | hsa_circ_0042799 |
| hsa-miR-1270 | hsa_circ_0092283 |
| hsa-miR-1272 | hsa_circ_0002564 |
| hsa-miR-1272 | hsa_circ_0002289 |
| hsa-miR-1273a | hsa_circ_0029405 |
| hsa-miR-1281 | hsa_circ_0007001 |
| hsa-miR-1281 | hsa_circ_0040921 |
| hsa-miR-1281 | hsa_circ_0002451 |
| hsa-miR-1281 | hsa_circ_0005630 |
| hsa-miR-1288-3p | hsa_circ_0050119 |
| hsa-miR-1292-5p | hsa_circ_0000497 |
| hsa-miR-1292-5p | hsa_circ_0007146 |
| hsa-miR-1292-5p | hsa_circ_0004003 |
| hsa-miR-1292-5p | hsa_circ_0004891 |
| hsa-miR-1292-5p | hsa_circ_0049657 |
| hsa-miR-1292-5p | hsa_circ_0050119 |
| hsa-miR-1292-5p | hsa_circ_0000973 |
| hsa-miR-1292-5p | hsa_circ_0002113 |
| hsa-miR-1292-5p | hsa_circ_0092299 |
| hsa-miR-1292-5p | hsa_circ_0004705 |
| hsa-miR-1292-5p | hsa_circ_0001394 |
| hsa-miR-1292-5p | hsa_circ_0007132 |
| hsa-miR-1292-5p | hsa_circ_0075748 |
| hsa-miR-1292-3p | hsa_circ_0020594 |
| hsa-miR-1292-3p | hsa_circ_0040921 |
| hsa-miR-1292-3p | hsa_circ_0045890 |
| hsa-miR-1292-3p | hsa_circ_0048025 |
| hsa-miR-1292-3p | hsa_circ_0002926 |
| hsa-miR-1292-3p | hsa_circ_0004699 |
| hsa-miR-1292-3p | hsa_circ_0070396 |
| hsa-miR-1292-3p | hsa_circ_0008113 |
| hsa-miR-1292-3p | hsa_circ_0087305 |
| hsa-miR-1306-5p | hsa_circ_0007372 |
| hsa-miR-1307-5p | hsa_circ_0075748 |
| hsa-miR-1307-3p | hsa_circ_0018909 |
| hsa-miR-1307-3p | hsa_circ_0000417 |
| hsa-miR-1307-3p | hsa_circ_0046430 |
| hsa-miR-1307-3p | hsa_circ_0002805 |
| hsa-miR-1307-3p | hsa_circ_0061179 |
| hsa-miR-1307-3p | hsa_circ_0004692 |
| hsa-miR-1307-3p | hsa_circ_0065147 |
| hsa-miR-1307-3p | hsa_circ_0065149 |
| hsa-miR-1307-3p | hsa_circ_0002569 |
| hsa-miR-1469 | hsa_circ_0006608 |
| hsa-miR-1469 | hsa_circ_0003168 |
| hsa-miR-1469 | hsa_circ_0007146 |
| hsa-miR-1469 | hsa_circ_0040823 |
| hsa-miR-1469 | hsa_circ_0046292 |
| hsa-miR-1469 | hsa_circ_0046430 |
| hsa-miR-1469 | hsa_circ_0048025 |
| hsa-miR-1469 | hsa_circ_0002926 |
| hsa-miR-1469 | hsa_circ_0050119 |
| hsa-miR-1469 | hsa_circ_0003854 |
| hsa-miR-1469 | hsa_circ_0002805 |
| hsa-miR-1469 | hsa_circ_0061179 |
| hsa-miR-1469 | hsa_circ_0002954 |
| hsa-miR-1469 | hsa_circ_0092283 |
| hsa-miR-1469 | hsa_circ_0001394 |
| hsa-miR-1469 | hsa_circ_0007132 |
| hsa-miR-1469 | hsa_circ_0001658 |
| hsa-miR-1469 | hsa_circ_0001708 |
| hsa-miR-1469 | hsa_circ_0081343 |
| hsa-miR-1469 | hsa_circ_0087305 |
| hsa-miR-1470 | hsa_circ_0020594 |
| hsa-miR-1470 | hsa_circ_0031933 |
| hsa-miR-1470 | hsa_circ_0045890 |
| hsa-miR-1470 | hsa_circ_0050119 |
| hsa-miR-1470 | hsa_circ_0002805 |
| hsa-miR-1470 | hsa_circ_0092283 |
| hsa-miR-1470 | hsa_circ_0007132 |
| hsa-miR-1470 | hsa_circ_0001658 |
| hsa-miR-1470 | hsa_circ_0002094 |
| hsa-miR-1470 | hsa_circ_0087305 |
| hsa-miR-1471 | hsa_circ_0007015 |
| hsa-miR-1471 | hsa_circ_0020594 |
| hsa-miR-1471 | hsa_circ_0050119 |
| hsa-miR-1471 | hsa_circ_0092297 |
| hsa-miR-1471 | hsa_circ_0001329 |
| hsa-miR-1471 | hsa_circ_0006884 |
| hsa-miR-1538 | hsa_circ_0008225 |
| hsa-miR-1538 | hsa_circ_0006608 |
| hsa-miR-1538 | hsa_circ_0003489 |
| hsa-miR-1538 | hsa_circ_0000682 |
| hsa-miR-1538 | hsa_circ_0000690 |
| hsa-miR-1538 | hsa_circ_0045890 |
| hsa-miR-1538 | hsa_circ_0000950 |
| hsa-miR-1538 | hsa_circ_0092283 |
| hsa-miR-1538 | hsa_circ_0004470 |
| hsa-miR-1538 | hsa_circ_0002077 |
| hsa-miR-1538 | hsa_circ_0007132 |
| hsa-miR-1538 | hsa_circ_0005630 |
| hsa-miR-1538 | hsa_circ_0087288 |
| hsa-miR-1538 | hsa_circ_0007685 |
| hsa-miR-1538 | hsa_circ_0001910 |
| hsa-miR-1539 | hsa_circ_0009732 |
| hsa-miR-1539 | hsa_circ_0017446 |
| hsa-miR-1539 | hsa_circ_0000206 |
| hsa-miR-1539 | hsa_circ_0003379 |
| hsa-miR-1539 | hsa_circ_0092299 |
| hsa-miR-1539 | hsa_circ_0076793 |
| hsa-miR-1908-5p | hsa_circ_0045890 |
| hsa-miR-1908-5p | hsa_circ_0048025 |
| hsa-miR-1908-5p | hsa_circ_0074816 |
| hsa-miR-1908-5p | hsa_circ_0074817 |
| hsa-miR-1908-5p | hsa_circ_0007132 |
| hsa-miR-1908-5p | hsa_circ_0003340 |
| hsa-miR-1908-3p | hsa_circ_0011571 |
| hsa-miR-1908-3p | hsa_circ_0011572 |
| hsa-miR-1908-3p | hsa_circ_0020594 |
| hsa-miR-1908-3p | hsa_circ_0003379 |
| hsa-miR-1908-3p | hsa_circ_0007001 |
| hsa-miR-1908-3p | hsa_circ_0022723 |
| hsa-miR-1908-3p | hsa_circ_0034920 |
| hsa-miR-1908-3p | hsa_circ_0007146 |
| hsa-miR-1908-3p | hsa_circ_0000682 |
| hsa-miR-1908-3p | hsa_circ_0000690 |
| hsa-miR-1908-3p | hsa_circ_0040823 |
| hsa-miR-1908-3p | hsa_circ_0045890 |
| hsa-miR-1908-3p | hsa_circ_0050119 |
| hsa-miR-1908-3p | hsa_circ_0003854 |
| hsa-miR-1908-3p | hsa_circ_0002805 |
| hsa-miR-1908-3p | hsa_circ_0061179 |
| hsa-miR-1908-3p | hsa_circ_0092283 |
| hsa-miR-1908-3p | hsa_circ_0004692 |
| hsa-miR-1908-3p | hsa_circ_0065147 |
| hsa-miR-1908-3p | hsa_circ_0065149 |
| hsa-miR-1908-3p | hsa_circ_0002569 |
| hsa-miR-1908-3p | hsa_circ_0006884 |
| hsa-miR-1908-3p | hsa_circ_0070396 |
| hsa-miR-1908-3p | hsa_circ_0007132 |
| hsa-miR-1909-5p | hsa_circ_0006254 |
| hsa-miR-1909-5p | hsa_circ_0003768 |
| hsa-miR-1909-5p | hsa_circ_0004692 |
| hsa-miR-1909-5p | hsa_circ_0065147 |
| hsa-miR-1909-5p | hsa_circ_0065149 |
| hsa-miR-1909-5p | hsa_circ_0002569 |
| hsa-miR-1909-5p | hsa_circ_0007132 |
| hsa-miR-1909-5p | hsa_circ_0001658 |
| hsa-miR-1909-5p | hsa_circ_0001748 |
| hsa-miR-1909-3p | hsa_circ_0007846 |
| hsa-miR-1909-3p | hsa_circ_0004519 |
| hsa-miR-1909-3p | hsa_circ_0040823 |
| hsa-miR-1909-3p | hsa_circ_0092337 |
| hsa-miR-1909-3p | hsa_circ_0045890 |
| hsa-miR-1909-3p | hsa_circ_0046430 |
| hsa-miR-1909-3p | hsa_circ_0000996 |
| hsa-miR-1909-3p | hsa_circ_0092299 |
| hsa-miR-1909-3p | hsa_circ_0007132 |
| hsa-miR-1909-3p | hsa_circ_0005630 |
| hsa-miR-1909-3p | hsa_circ_0001829 |
| hsa-miR-1909-3p | hsa_circ_0087305 |
| hsa-miR-1910-5p | hsa_circ_0018909 |
| hsa-miR-1910-5p | hsa_circ_0020594 |
| hsa-miR-1910-5p | hsa_circ_0022723 |
| hsa-miR-1910-5p | hsa_circ_0025388 |
| hsa-miR-1910-5p | hsa_circ_0003315 |
| hsa-miR-1910-5p | hsa_circ_0050119 |
| hsa-miR-1910-5p | hsa_circ_0076995 |
| hsa-miR-1910-5p | hsa_circ_0005630 |
| hsa-miR-1910-5p | hsa_circ_0008113 |
| hsa-miR-1913 | hsa_circ_0033144 |
| hsa-miR-1913 | hsa_circ_0034189 |
| hsa-miR-1913 | hsa_circ_0004692 |
| hsa-miR-1913 | hsa_circ_0065147 |
| hsa-miR-1913 | hsa_circ_0065149 |
| hsa-miR-1913 | hsa_circ_0002569 |
| hsa-miR-1913 | hsa_circ_0006693 |
| hsa-miR-1913 | hsa_circ_0001417 |
| hsa-miR-1913 | hsa_circ_0007132 |
| hsa-miR-1913 | hsa_circ_0001756 |
| hsa-miR-1913 | hsa_circ_0008113 |
| hsa-miR-1914-5p | hsa_circ_0009732 |
| hsa-miR-1914-5p | hsa_circ_0003168 |
| hsa-miR-1914-5p | hsa_circ_0092297 |
| hsa-miR-1914-5p | hsa_circ_0007967 |
| hsa-miR-1914-5p | hsa_circ_0005630 |
| hsa-miR-1914-3p | hsa_circ_0009349 |
| hsa-miR-1914-3p | hsa_circ_0009357 |
| hsa-miR-1914-3p | hsa_circ_0009360 |
| hsa-miR-1914-3p | hsa_circ_0015004 |
| hsa-miR-1914-3p | hsa_circ_0007015 |
| hsa-miR-1914-3p | hsa_circ_0003557 |
| hsa-miR-1914-3p | hsa_circ_0000375 |
| hsa-miR-1914-3p | hsa_circ_0000417 |
| hsa-miR-1914-3p | hsa_circ_0000565 |
| hsa-miR-1914-3p | hsa_circ_0007146 |
| hsa-miR-1914-3p | hsa_circ_0005571 |
| hsa-miR-1914-3p | hsa_circ_0000950 |
| hsa-miR-1914-3p | hsa_circ_0004218 |
| hsa-miR-1914-3p | hsa_circ_0002805 |
| hsa-miR-1914-3p | hsa_circ_0092299 |
| hsa-miR-1914-3p | hsa_circ_0003602 |
| hsa-miR-1914-3p | hsa_circ_0006693 |
| hsa-miR-1914-3p | hsa_circ_0001417 |
| hsa-miR-1914-3p | hsa_circ_0003278 |
| hsa-miR-1914-3p | hsa_circ_0081423 |
| hsa-miR-1914-3p | hsa_circ_0002094 |
| hsa-miR-1914-3p | hsa_circ_0005630 |
| hsa-miR-1914-3p | hsa_circ_0092125 |
| hsa-miR-1915-5p | hsa_circ_0000650 |
| hsa-miR-1915-5p | hsa_circ_0036768 |
| hsa-miR-1915-5p | hsa_circ_0007132 |
| hsa-miR-1915-3p | hsa_circ_0018909 |
| hsa-miR-1915-3p | hsa_circ_0007146 |
| hsa-miR-1915-3p | hsa_circ_0007846 |
| hsa-miR-1915-3p | hsa_circ_0049998 |
| hsa-miR-1915-3p | hsa_circ_0061052 |
| hsa-miR-1915-3p | hsa_circ_0002805 |
| hsa-miR-1915-3p | hsa_circ_0061179 |
| hsa-miR-1915-3p | hsa_circ_0007132 |
| hsa-miR-1915-3p | hsa_circ_0081343 |
| hsa-miR-1915-3p | hsa_circ_0087305 |
| hsa-miR-1915-3p | hsa_circ_0001947 |
| hsa-miR-1972 | hsa_circ_0029405 |
| hsa-miR-1972 | hsa_circ_0000918 |
| hsa-miR-1972 | hsa_circ_0000973 |
| hsa-miR-1972 | hsa_circ_0092299 |
| hsa-miR-1972 | hsa_circ_0092283 |
| hsa-miR-1972 | hsa_circ_0007132 |
| hsa-miR-1972 | hsa_circ_0005630 |
| hsa-miR-1976 | hsa_circ_0040921 |
| hsa-miR-1976 | hsa_circ_0072437 |
| hsa-miR-1976 | hsa_circ_0001658 |
| hsa-miR-1976 | hsa_circ_0002094 |
| hsa-miR-2114-5p | hsa_circ_0058058 |
| hsa-miR-2277-5p | hsa_circ_0008225 |
| hsa-miR-2277-5p | hsa_circ_0000247 |
| hsa-miR-2277-5p | hsa_circ_0007146 |
| hsa-miR-2277-5p | hsa_circ_0004519 |
| hsa-miR-2277-5p | hsa_circ_0040921 |
| hsa-miR-2277-5p | hsa_circ_0045890 |
| hsa-miR-2277-5p | hsa_circ_0045905 |
| hsa-miR-2277-5p | hsa_circ_0046430 |
| hsa-miR-2277-5p | hsa_circ_0048025 |
| hsa-miR-2277-5p | hsa_circ_0049657 |
| hsa-miR-2277-5p | hsa_circ_0002805 |
| hsa-miR-2277-5p | hsa_circ_0061179 |
| hsa-miR-2277-5p | hsa_circ_0092299 |
| hsa-miR-2277-5p | hsa_circ_0092283 |
| hsa-miR-2277-5p | hsa_circ_0004470 |
| hsa-miR-2277-5p | hsa_circ_0006693 |
| hsa-miR-2277-5p | hsa_circ_0001417 |
| hsa-miR-2277-5p | hsa_circ_0070467 |
| hsa-miR-2277-5p | hsa_circ_0007132 |
| hsa-miR-2277-5p | hsa_circ_0001829 |
| hsa-miR-2277-3p | hsa_circ_0003557 |
| hsa-miR-2681-5p | hsa_circ_0002564 |
| hsa-miR-2681-5p | hsa_circ_0002289 |
| hsa-miR-2682-5p | hsa_circ_0040921 |
| hsa-miR-2682-3p | hsa_circ_0007132 |
| hsa-miR-2682-3p | hsa_circ_0008113 |
| hsa-miR-711 | hsa_circ_0030051 |
| hsa-miR-711 | hsa_circ_0006735 |
| hsa-miR-711 | hsa_circ_0007609 |
| hsa-miR-711 | hsa_circ_0001400 |
| hsa-miR-718 | hsa_circ_0000131 |
| hsa-miR-718 | hsa_circ_0020594 |
| hsa-miR-718 | hsa_circ_0002564 |
| hsa-miR-718 | hsa_circ_0002289 |
| hsa-miR-718 | hsa_circ_0007146 |
| hsa-miR-718 | hsa_circ_0004003 |
| hsa-miR-718 | hsa_circ_0004891 |
| hsa-miR-718 | hsa_circ_0070396 |
| hsa-miR-718 | hsa_circ_0007132 |
| hsa-miR-2861 | hsa_circ_0000417 |
| hsa-miR-2861 | hsa_circ_0033144 |
| hsa-miR-2861 | hsa_circ_0034972 |
| hsa-miR-2861 | hsa_circ_0007146 |
| hsa-miR-2861 | hsa_circ_0040823 |
| hsa-miR-2861 | hsa_circ_0046430 |
| hsa-miR-2861 | hsa_circ_0008590 |
| hsa-miR-2861 | hsa_circ_0002377 |
| hsa-miR-2861 | hsa_circ_0092299 |
| hsa-miR-2861 | hsa_circ_0001400 |
| hsa-miR-2861 | hsa_circ_0071311 |
| hsa-miR-2861 | hsa_circ_0007132 |
| hsa-miR-2861 | hsa_circ_0005630 |
| hsa-miR-2861 | hsa_circ_0087305 |
| hsa-miR-2861 | hsa_circ_0087855 |
| hsa-miR-2861 | hsa_circ_0008812 |
| hsa-miR-2861 | hsa_circ_0006174 |
| hsa-miR-2861 | hsa_circ_0087861 |
| hsa-miR-2861 | hsa_circ_0087862 |
| hsa-miR-3125 | hsa_circ_0076742 |
| hsa-miR-3126-3p | hsa_circ_0007132 |
| hsa-miR-3127-3p | hsa_circ_0002402 |
| hsa-miR-3127-3p | hsa_circ_0011571 |
| hsa-miR-3127-3p | hsa_circ_0011572 |
| hsa-miR-3127-3p | hsa_circ_0003168 |
| hsa-miR-3127-3p | hsa_circ_0000417 |
| hsa-miR-3127-3p | hsa_circ_0035649 |
| hsa-miR-3127-3p | hsa_circ_0000613 |
| hsa-miR-3127-3p | hsa_circ_0008153 |
| hsa-miR-3127-3p | hsa_circ_0049888 |
| hsa-miR-3127-3p | hsa_circ_0004872 |
| hsa-miR-3127-3p | hsa_circ_0092299 |
| hsa-miR-3127-3p | hsa_circ_0070040 |
| hsa-miR-3127-3p | hsa_circ_0007145 |
| hsa-miR-3130-5p | hsa_circ_0092297 |
| hsa-miR-3130-5p | hsa_circ_0007132 |
| hsa-miR-3130-3p | hsa_circ_0002094 |
| hsa-miR-3131 | hsa_circ_0006254 |
| hsa-miR-3131 | hsa_circ_0003768 |
| hsa-miR-3131 | hsa_circ_0045890 |
| hsa-miR-3131 | hsa_circ_0045905 |
| hsa-miR-3131 | hsa_circ_0004003 |
| hsa-miR-3131 | hsa_circ_0050119 |
| hsa-miR-3131 | hsa_circ_0008261 |
| hsa-miR-3132 | hsa_circ_0000395 |
| hsa-miR-3132 | hsa_circ_0031933 |
| hsa-miR-3132 | hsa_circ_0000996 |
| hsa-miR-3132 | hsa_circ_0002805 |
| hsa-miR-3132 | hsa_circ_0061179 |
| hsa-miR-3132 | hsa_circ_0006168 |
| hsa-miR-3132 | hsa_circ_0007137 |
| hsa-miR-3132 | hsa_circ_0001806 |
| hsa-miR-3135a | hsa_circ_0020594 |
| hsa-miR-466 | hsa_circ_0000973 |
| hsa-miR-3137 | hsa_circ_0000417 |
| hsa-miR-3137 | hsa_circ_0029405 |
| hsa-miR-3137 | hsa_circ_0006597 |
| hsa-miR-3137 | hsa_circ_0034189 |
| hsa-miR-3137 | hsa_circ_0002926 |
| hsa-miR-3137 | hsa_circ_0002377 |
| hsa-miR-3137 | hsa_circ_0007132 |
| hsa-miR-3137 | hsa_circ_0003340 |
| hsa-miR-3138 | hsa_circ_0015004 |
| hsa-miR-3138 | hsa_circ_0006649 |
| hsa-miR-3138 | hsa_circ_0008102 |
| hsa-miR-3138 | hsa_circ_0022383 |
| hsa-miR-3138 | hsa_circ_0052131 |
| hsa-miR-3138 | hsa_circ_0000973 |
| hsa-miR-3138 | hsa_circ_0004218 |
| hsa-miR-3138 | hsa_circ_0092277 |
| hsa-miR-3138 | hsa_circ_0076742 |
| hsa-miR-3138 | hsa_circ_0087288 |
| hsa-miR-3141 | hsa_circ_0003379 |
| hsa-miR-3141 | hsa_circ_0000417 |
| hsa-miR-3141 | hsa_circ_0028190 |
| hsa-miR-3141 | hsa_circ_0003315 |
| hsa-miR-3141 | hsa_circ_0045890 |
| hsa-miR-3141 | hsa_circ_0046430 |
| hsa-miR-3141 | hsa_circ_0002805 |
| hsa-miR-3141 | hsa_circ_0069748 |
| hsa-miR-3141 | hsa_circ_0001806 |
| hsa-miR-3141 | hsa_circ_0087305 |
| hsa-miR-1273c | hsa_circ_0029405 |
| hsa-miR-3147 | hsa_circ_0017248 |
| hsa-miR-3147 | hsa_circ_0017289 |
| hsa-miR-3147 | hsa_circ_0005090 |
| hsa-miR-3147 | hsa_circ_0020005 |
| hsa-miR-3147 | hsa_circ_0003557 |
| hsa-miR-3147 | hsa_circ_0000375 |
| hsa-miR-3147 | hsa_circ_0000417 |
| hsa-miR-3147 | hsa_circ_0004137 |
| hsa-miR-3147 | hsa_circ_0008378 |
| hsa-miR-3147 | hsa_circ_0036044 |
| hsa-miR-3147 | hsa_circ_0040823 |
| hsa-miR-3147 | hsa_circ_0040921 |
| hsa-miR-3147 | hsa_circ_0049998 |
| hsa-miR-3147 | hsa_circ_0007609 |
| hsa-miR-3147 | hsa_circ_0092299 |
| hsa-miR-3147 | hsa_circ_0006681 |
| hsa-miR-3147 | hsa_circ_0001400 |
| hsa-miR-3147 | hsa_circ_0074816 |
| hsa-miR-3147 | hsa_circ_0074817 |
| hsa-miR-3147 | hsa_circ_0001549 |
| hsa-miR-3147 | hsa_circ_0001573 |
| hsa-miR-3147 | hsa_circ_0003340 |
| hsa-miR-3147 | hsa_circ_0001756 |
| hsa-miR-3147 | hsa_circ_0005630 |
| hsa-miR-3147 | hsa_circ_0001806 |
| hsa-miR-3147 | hsa_circ_0087288 |
| hsa-miR-3150a-5p | hsa_circ_0025388 |
| hsa-miR-3150a-3p | hsa_circ_0008604 |
| hsa-miR-3150a-3p | hsa_circ_0002805 |
| hsa-miR-3150a-3p | hsa_circ_0061179 |
| hsa-miR-3150a-3p | hsa_circ_0001187 |
| hsa-miR-3150a-3p | hsa_circ_0008078 |
| hsa-miR-3150a-3p | hsa_circ_0001472 |
| hsa-miR-3150a-3p | hsa_circ_0007132 |
| hsa-miR-3150a-3p | hsa_circ_0003700 |
| hsa-miR-3151-5p | hsa_circ_0018909 |
| hsa-miR-3151-5p | hsa_circ_0008521 |
| hsa-miR-3151-5p | hsa_circ_0003848 |
| hsa-miR-3151-5p | hsa_circ_0002564 |
| hsa-miR-3151-5p | hsa_circ_0002289 |
| hsa-miR-3151-5p | hsa_circ_0001806 |
| hsa-miR-3074-5p | hsa_circ_0055904 |
| hsa-miR-3154 | hsa_circ_0002570 |
| hsa-miR-3154 | hsa_circ_0017289 |
| hsa-miR-3154 | hsa_circ_0005090 |
| hsa-miR-3154 | hsa_circ_0003958 |
| hsa-miR-3154 | hsa_circ_0081423 |
| hsa-miR-3154 | hsa_circ_0005630 |
| hsa-miR-3154 | hsa_circ_0084429 |
| hsa-miR-3155a | hsa_circ_0003557 |
| hsa-miR-3157-5p | hsa_circ_0007846 |
| hsa-miR-3160-5p | hsa_circ_0067913 |
| hsa-miR-3160-5p | hsa_circ_0067919 |
| hsa-miR-3160-5p | hsa_circ_0005362 |
| hsa-miR-3162-5p | hsa_circ_0015004 |
| hsa-miR-3162-5p | hsa_circ_0040921 |
| hsa-miR-3162-5p | hsa_circ_0045890 |
| hsa-miR-3162-5p | hsa_circ_0000996 |
| hsa-miR-3162-5p | hsa_circ_0004218 |
| hsa-miR-3162-5p | hsa_circ_0092299 |
| hsa-miR-3162-5p | hsa_circ_0004470 |
| hsa-miR-3162-5p | hsa_circ_0076742 |
| hsa-miR-3162-5p | hsa_circ_0087305 |
| hsa-miR-3162-3p | hsa_circ_0022723 |
| hsa-miR-3162-3p | hsa_circ_0000395 |
| hsa-miR-3162-3p | hsa_circ_0000996 |
| hsa-miR-3162-3p | hsa_circ_0092299 |
| hsa-miR-3162-3p | hsa_circ_0007132 |
| hsa-miR-3166 | hsa_circ_0004470 |
| hsa-miR-3170 | hsa_circ_0022383 |
| hsa-miR-3173-5p | hsa_circ_0064136 |
| hsa-miR-1193 | hsa_circ_0006137 |
| hsa-miR-1193 | hsa_circ_0064136 |
| hsa-miR-1193 | hsa_circ_0004136 |
| hsa-miR-323b-5p | hsa_circ_0018909 |
| hsa-miR-323b-5p | hsa_circ_0033144 |
| hsa-miR-323b-5p | hsa_circ_0040921 |
| hsa-miR-323b-5p | hsa_circ_0000854 |
| hsa-miR-3175 | hsa_circ_0001400 |
| hsa-miR-3177-5p | hsa_circ_0007146 |
| hsa-miR-3177-3p | hsa_circ_0045890 |
| hsa-miR-3180-5p | hsa_circ_0030051 |
| hsa-miR-3180-5p | hsa_circ_0092283 |
| hsa-miR-3180-5p | hsa_circ_0004705 |
| hsa-miR-3180-5p | hsa_circ_0007132 |
| hsa-miR-3180-3p | hsa_circ_0006608 |
| hsa-miR-3180-3p | hsa_circ_0008102 |
| hsa-miR-3180-3p | hsa_circ_0000417 |
| hsa-miR-3180-3p | hsa_circ_0007146 |
| hsa-miR-3180-3p | hsa_circ_0040921 |
| hsa-miR-3180-3p | hsa_circ_0045890 |
| hsa-miR-3180-3p | hsa_circ_0046292 |
| hsa-miR-3180-3p | hsa_circ_0046430 |
| hsa-miR-3180-3p | hsa_circ_0048025 |
| hsa-miR-3180-3p | hsa_circ_0004003 |
| hsa-miR-3180-3p | hsa_circ_0004891 |
| hsa-miR-3180-3p | hsa_circ_0005571 |
| hsa-miR-3180-3p | hsa_circ_0050119 |
| hsa-miR-3180-3p | hsa_circ_0092297 |
| hsa-miR-3180-3p | hsa_circ_0003854 |
| hsa-miR-3180-3p | hsa_circ_0060043 |
| hsa-miR-3180-3p | hsa_circ_0002077 |
| hsa-miR-3180-3p | hsa_circ_0069748 |
| hsa-miR-3180-3p | hsa_circ_0002755 |
| hsa-miR-3180-3p | hsa_circ_0002094 |
| hsa-miR-3180-3p | hsa_circ_0005630 |
| hsa-miR-3181 | hsa_circ_0000690 |
| hsa-miR-3181 | hsa_circ_0046292 |
| hsa-miR-3181 | hsa_circ_0048025 |
| hsa-miR-3181 | hsa_circ_0002805 |
| hsa-miR-3181 | hsa_circ_0061179 |
| hsa-miR-3181 | hsa_circ_0092299 |
| hsa-miR-3181 | hsa_circ_0004692 |
| hsa-miR-3181 | hsa_circ_0065147 |
| hsa-miR-3181 | hsa_circ_0065149 |
| hsa-miR-3181 | hsa_circ_0002569 |
| hsa-miR-3181 | hsa_circ_0007132 |
| hsa-miR-3181 | hsa_circ_0001756 |
| hsa-miR-3183 | hsa_circ_0049998 |
| hsa-miR-3183 | hsa_circ_0054086 |
| hsa-miR-3183 | hsa_circ_0001573 |
| hsa-miR-3184-5p | hsa_circ_0000375 |
| hsa-miR-3184-5p | hsa_circ_0043522 |
| hsa-miR-3184-5p | hsa_circ_0046430 |
| hsa-miR-3184-5p | hsa_circ_0003854 |
| hsa-miR-3184-5p | hsa_circ_0075748 |
| hsa-miR-3184-5p | hsa_circ_0001829 |
| hsa-miR-3184-5p | hsa_circ_0001866 |
| hsa-miR-3186-5p | hsa_circ_0007015 |
| hsa-miR-3186-5p | hsa_circ_0003557 |
| hsa-miR-3186-5p | hsa_circ_0007132 |
| hsa-miR-3186-5p | hsa_circ_0006022 |
| hsa-miR-3187-5p | hsa_circ_0000048 |
| hsa-miR-3187-5p | hsa_circ_0007146 |
| hsa-miR-3187-5p | hsa_circ_0040823 |
| hsa-miR-3187-5p | hsa_circ_0048025 |
| hsa-miR-3187-5p | hsa_circ_0004218 |
| hsa-miR-3187-5p | hsa_circ_0002805 |
| hsa-miR-3187-5p | hsa_circ_0061179 |
| hsa-miR-3187-5p | hsa_circ_0072437 |
| hsa-miR-3187-5p | hsa_circ_0007132 |
| hsa-miR-3187-5p | hsa_circ_0004712 |
| hsa-miR-3187-5p | hsa_circ_0081423 |
| hsa-miR-3187-5p | hsa_circ_0005630 |
| hsa-miR-3187-5p | hsa_circ_0084429 |
| hsa-miR-3187-3p | hsa_circ_0007146 |
| hsa-miR-3187-3p | hsa_circ_0045890 |
| hsa-miR-3187-3p | hsa_circ_0048025 |
| hsa-miR-3187-3p | hsa_circ_0000909 |
| hsa-miR-3187-3p | hsa_circ_0006006 |
| hsa-miR-3187-3p | hsa_circ_0007132 |
| hsa-miR-3187-3p | hsa_circ_0009092 |
| hsa-miR-3188 | hsa_circ_0007015 |
| hsa-miR-3188 | hsa_circ_0006006 |
| hsa-miR-3189-5p | hsa_circ_0009349 |
| hsa-miR-3189-5p | hsa_circ_0002402 |
| hsa-miR-3189-5p | hsa_circ_0017289 |
| hsa-miR-3189-5p | hsa_circ_0005090 |
| hsa-miR-3189-5p | hsa_circ_0022723 |
| hsa-miR-3189-5p | hsa_circ_0031933 |
| hsa-miR-3189-5p | hsa_circ_0007846 |
| hsa-miR-3189-5p | hsa_circ_0000690 |
| hsa-miR-3189-5p | hsa_circ_0040823 |
| hsa-miR-3189-5p | hsa_circ_0046430 |
| hsa-miR-3189-5p | hsa_circ_0000836 |
| hsa-miR-3189-5p | hsa_circ_0000936 |
| hsa-miR-3189-5p | hsa_circ_0000996 |
| hsa-miR-3189-5p | hsa_circ_0002805 |
| hsa-miR-3189-5p | hsa_circ_0092299 |
| hsa-miR-3189-5p | hsa_circ_0006693 |
| hsa-miR-3189-5p | hsa_circ_0006552 |
| hsa-miR-3189-5p | hsa_circ_0071865 |
| hsa-miR-3189-5p | hsa_circ_0007132 |
| hsa-miR-3189-5p | hsa_circ_0002755 |
| hsa-miR-3189-5p | hsa_circ_0001756 |
| hsa-miR-3189-5p | hsa_circ_0005630 |
| hsa-miR-3189-5p | hsa_circ_0087305 |
| hsa-miR-3189-3p | hsa_circ_0022383 |
| hsa-miR-3189-3p | hsa_circ_0046430 |
| hsa-miR-3190-5p | hsa_circ_0001756 |
| hsa-miR-3190-5p | hsa_circ_0005630 |
| hsa-miR-3191-5p | hsa_circ_0002805 |
| hsa-miR-3191-5p | hsa_circ_0061179 |
| hsa-miR-3191-5p | hsa_circ_0092283 |
| hsa-miR-3191-5p | hsa_circ_0007132 |
| hsa-miR-3191-3p | hsa_circ_0046430 |
| hsa-miR-3191-3p | hsa_circ_0048025 |
| hsa-miR-3191-3p | hsa_circ_0000973 |
| hsa-miR-3191-3p | hsa_circ_0054086 |
| hsa-miR-3191-3p | hsa_circ_0092299 |
| hsa-miR-3191-3p | hsa_circ_0001947 |
| hsa-miR-3192-5p | hsa_circ_0008102 |
| hsa-miR-3192-5p | hsa_circ_0045890 |
| hsa-miR-3192-5p | hsa_circ_0046430 |
| hsa-miR-3192-5p | hsa_circ_0003602 |
| hsa-miR-3192-5p | hsa_circ_0001602 |
| hsa-miR-3193 | hsa_circ_0003489 |
| hsa-miR-3194-5p | hsa_circ_0005664 |
| hsa-miR-3194-5p | hsa_circ_0046430 |
| hsa-miR-3194-5p | hsa_circ_0002926 |
| hsa-miR-3194-5p | hsa_circ_0058058 |
| hsa-miR-3194-5p | hsa_circ_0092283 |
| hsa-miR-3194-5p | hsa_circ_0006884 |
| hsa-miR-3194-5p | hsa_circ_0001400 |
| hsa-miR-3194-5p | hsa_circ_0006693 |
| hsa-miR-3194-5p | hsa_circ_0001417 |
| hsa-miR-3194-5p | hsa_circ_0007132 |
| hsa-miR-3194-3p | hsa_circ_0008916 |
| hsa-miR-3194-3p | hsa_circ_0035944 |
| hsa-miR-3194-3p | hsa_circ_0061936 |
| hsa-miR-3195 | hsa_circ_0003557 |
| hsa-miR-3196 | hsa_circ_0003379 |
| hsa-miR-3196 | hsa_circ_0000417 |
| hsa-miR-3196 | hsa_circ_0007146 |
| hsa-miR-3196 | hsa_circ_0040921 |
| hsa-miR-3196 | hsa_circ_0048025 |
| hsa-miR-3196 | hsa_circ_0007132 |
| hsa-miR-3197 | hsa_circ_0009349 |
| hsa-miR-3197 | hsa_circ_0009357 |
| hsa-miR-3197 | hsa_circ_0007015 |
| hsa-miR-3197 | hsa_circ_0003168 |
| hsa-miR-3197 | hsa_circ_0006877 |
| hsa-miR-3197 | hsa_circ_0062397 |
| hsa-miR-3197 | hsa_circ_0003602 |
| hsa-miR-3197 | hsa_circ_0005630 |
| hsa-miR-3197 | hsa_circ_0001829 |
| hsa-miR-3198 | hsa_circ_0000417 |
| hsa-miR-3198 | hsa_circ_0045890 |
| hsa-miR-3198 | hsa_circ_0000854 |
| hsa-miR-3198 | hsa_circ_0002926 |
| hsa-miR-3198 | hsa_circ_0000918 |
| hsa-miR-3198 | hsa_circ_0092297 |
| hsa-miR-3198 | hsa_circ_0069748 |
| hsa-miR-3198 | hsa_circ_0001472 |
| hsa-miR-3198 | hsa_circ_0087288 |
| hsa-miR-3200-3p | hsa_circ_0007132 |
| hsa-miR-1273d | hsa_circ_0007015 |
| hsa-miR-1273d | hsa_circ_0020594 |
| hsa-miR-1273d | hsa_circ_0029405 |
| hsa-miR-1273d | hsa_circ_0004705 |
| hsa-miR-1273d | hsa_circ_0003528 |
| hsa-miR-1273d | hsa_circ_0084552 |
| hsa-miR-378c | hsa_circ_0000650 |
| hsa-miR-378c | hsa_circ_0036768 |
| hsa-miR-378c | hsa_circ_0072857 |
| hsa-miR-4298 | hsa_circ_0009581 |
| hsa-miR-4298 | hsa_circ_0006837 |
| hsa-miR-4298 | hsa_circ_0020005 |
| hsa-miR-4298 | hsa_circ_0022383 |
| hsa-miR-4298 | hsa_circ_0000339 |
| hsa-miR-4298 | hsa_circ_0003908 |
| hsa-miR-4298 | hsa_circ_0000417 |
| hsa-miR-4298 | hsa_circ_0029633 |
| hsa-miR-4298 | hsa_circ_0007846 |
| hsa-miR-4298 | hsa_circ_0050119 |
| hsa-miR-4298 | hsa_circ_0052131 |
| hsa-miR-4298 | hsa_circ_0000996 |
| hsa-miR-4298 | hsa_circ_0092297 |
| hsa-miR-4298 | hsa_circ_0061936 |
| hsa-miR-4298 | hsa_circ_0092299 |
| hsa-miR-4298 | hsa_circ_0002077 |
| hsa-miR-4298 | hsa_circ_0069748 |
| hsa-miR-4298 | hsa_circ_0007132 |
| hsa-miR-4298 | hsa_circ_0084429 |
| hsa-miR-4298 | hsa_circ_0001947 |
| hsa-miR-4298 | hsa_circ_0092125 |
| hsa-miR-4313 | hsa_circ_0045890 |
| hsa-miR-4313 | hsa_circ_0004692 |
| hsa-miR-4313 | hsa_circ_0070396 |
| hsa-miR-4322 | hsa_circ_0007846 |
| hsa-miR-4322 | hsa_circ_0092337 |
| hsa-miR-4322 | hsa_circ_0046430 |
| hsa-miR-4322 | hsa_circ_0060904 |
| hsa-miR-4322 | hsa_circ_0002805 |
| hsa-miR-4322 | hsa_circ_0061179 |
| hsa-miR-4322 | hsa_circ_0092299 |
| hsa-miR-4322 | hsa_circ_0070467 |
| hsa-miR-4322 | hsa_circ_0001573 |
| hsa-miR-4322 | hsa_circ_0007132 |
| hsa-miR-4322 | hsa_circ_0081343 |
| hsa-miR-4322 | hsa_circ_0082179 |
| hsa-miR-4322 | hsa_circ_0006357 |
| hsa-miR-4322 | hsa_circ_0001829 |
| hsa-miR-4321 | hsa_circ_0002805 |
| hsa-miR-4321 | hsa_circ_0007132 |
| hsa-miR-4323 | hsa_circ_0001756 |
| hsa-miR-4257 | hsa_circ_0014132 |
| hsa-miR-4257 | hsa_circ_0001439 |
| hsa-miR-4258 | hsa_circ_0045890 |
| hsa-miR-4258 | hsa_circ_0007132 |
| hsa-miR-4259 | hsa_circ_0008102 |
| hsa-miR-4259 | hsa_circ_0003168 |
| hsa-miR-4259 | hsa_circ_0006254 |
| hsa-miR-4259 | hsa_circ_0003768 |
| hsa-miR-4259 | hsa_circ_0003908 |
| hsa-miR-4259 | hsa_circ_0000651 |
| hsa-miR-4259 | hsa_circ_0043278 |
| hsa-miR-4259 | hsa_circ_0092299 |
| hsa-miR-4259 | hsa_circ_0072797 |
| hsa-miR-4260 | hsa_circ_0007372 |
| hsa-miR-4260 | hsa_circ_0006735 |
| hsa-miR-4260 | hsa_circ_0004003 |
| hsa-miR-4260 | hsa_circ_0004891 |
| hsa-miR-4254 | hsa_circ_0003379 |
| hsa-miR-4254 | hsa_circ_0031933 |
| hsa-miR-4254 | hsa_circ_0070396 |
| hsa-miR-4254 | hsa_circ_0003278 |
| hsa-miR-4326 | hsa_circ_0000660 |
| hsa-miR-4326 | hsa_circ_0004692 |
| hsa-miR-4327 | hsa_circ_0007846 |
| hsa-miR-4327 | hsa_circ_0002113 |
| hsa-miR-4265 | hsa_circ_0000918 |
| hsa-miR-2355-3p | hsa_circ_0002094 |
| hsa-miR-4268 | hsa_circ_0000497 |
| hsa-miR-4268 | hsa_circ_0007132 |
| hsa-miR-4268 | hsa_circ_0076995 |
| hsa-miR-4269 | hsa_circ_0002037 |
| hsa-miR-4270 | hsa_circ_0007001 |
| hsa-miR-4270 | hsa_circ_0000437 |
| hsa-miR-4270 | hsa_circ_0047347 |
| hsa-miR-4270 | hsa_circ_0092299 |
| hsa-miR-4270 | hsa_circ_0003340 |
| hsa-miR-4274 | hsa_circ_0006693 |
| hsa-miR-4274 | hsa_circ_0001417 |
| hsa-miR-4281 | hsa_circ_0018909 |
| hsa-miR-4281 | hsa_circ_0022383 |
| hsa-miR-4281 | hsa_circ_0000417 |
| hsa-miR-4281 | hsa_circ_0040039 |
| hsa-miR-4281 | hsa_circ_0004354 |
| hsa-miR-4281 | hsa_circ_0046430 |
| hsa-miR-4281 | hsa_circ_0002926 |
| hsa-miR-4281 | hsa_circ_0002805 |
| hsa-miR-4281 | hsa_circ_0003602 |
| hsa-miR-4281 | hsa_circ_0071311 |
| hsa-miR-4281 | hsa_circ_0007132 |
| hsa-miR-4281 | hsa_circ_0001658 |
| hsa-miR-4281 | hsa_circ_0002094 |
| hsa-miR-4292 | hsa_circ_0000417 |
| hsa-miR-4292 | hsa_circ_0046430 |
| hsa-miR-4292 | hsa_circ_0050119 |
| hsa-miR-4292 | hsa_circ_0007132 |
| hsa-miR-4292 | hsa_circ_0007685 |
| hsa-miR-4292 | hsa_circ_0001910 |
| hsa-miR-4290 | hsa_circ_0004692 |
| hsa-miR-4290 | hsa_circ_0001658 |
| hsa-miR-4330 | hsa_circ_0067913 |
| hsa-miR-4330 | hsa_circ_0067919 |
| hsa-miR-4330 | hsa_circ_0005362 |
| hsa-miR-3605-3p | hsa_circ_0092299 |
| hsa-miR-3605-3p | hsa_circ_0084789 |
| hsa-miR-3605-3p | hsa_circ_0008113 |
| hsa-miR-3614-5p | hsa_circ_0040823 |
| hsa-miR-3614-5p | hsa_circ_0000836 |
| hsa-miR-3614-5p | hsa_circ_0092283 |
| hsa-miR-3614-3p | hsa_circ_0064136 |
| hsa-miR-3615 | hsa_circ_0020594 |
| hsa-miR-3615 | hsa_circ_0000690 |
| hsa-miR-3615 | hsa_circ_0046292 |
| hsa-miR-3615 | hsa_circ_0005630 |
| hsa-miR-3616-3p | hsa_circ_0007846 |
| hsa-miR-3619-5p | hsa_circ_0003557 |
| hsa-miR-3619-5p | hsa_circ_0007846 |
| hsa-miR-3619-5p | hsa_circ_0061936 |
| hsa-miR-3619-5p | hsa_circ_0071311 |
| hsa-miR-3619-5p | hsa_circ_0007145 |
| hsa-miR-3619-5p | hsa_circ_0001756 |
| hsa-miR-3619-3p | hsa_circ_0045890 |
| hsa-miR-3619-3p | hsa_circ_0064136 |
| hsa-miR-3619-3p | hsa_circ_0072437 |
| hsa-miR-3619-3p | hsa_circ_0007132 |
| hsa-miR-3620-5p | hsa_circ_0009349 |
| hsa-miR-3620-5p | hsa_circ_0002402 |
| hsa-miR-3620-5p | hsa_circ_0015004 |
| hsa-miR-3620-5p | hsa_circ_0007015 |
| hsa-miR-3620-5p | hsa_circ_0017438 |
| hsa-miR-3620-5p | hsa_circ_0004277 |
| hsa-miR-3620-5p | hsa_circ_0017446 |
| hsa-miR-3620-5p | hsa_circ_0000247 |
| hsa-miR-3620-5p | hsa_circ_0008102 |
| hsa-miR-3620-5p | hsa_circ_0006254 |
| hsa-miR-3620-5p | hsa_circ_0003768 |
| hsa-miR-3620-5p | hsa_circ_0022383 |
| hsa-miR-3620-5p | hsa_circ_0022392 |
| hsa-miR-3620-5p | hsa_circ_0022723 |
| hsa-miR-3620-5p | hsa_circ_0003557 |
| hsa-miR-3620-5p | hsa_circ_0000417 |
| hsa-miR-3620-5p | hsa_circ_0000497 |
| hsa-miR-3620-5p | hsa_circ_0033144 |
| hsa-miR-3620-5p | hsa_circ_0000650 |
| hsa-miR-3620-5p | hsa_circ_0036768 |
| hsa-miR-3620-5p | hsa_circ_0007146 |
| hsa-miR-3620-5p | hsa_circ_0007846 |
| hsa-miR-3620-5p | hsa_circ_0006735 |
| hsa-miR-3620-5p | hsa_circ_0040823 |
| hsa-miR-3620-5p | hsa_circ_0042799 |
| hsa-miR-3620-5p | hsa_circ_0045890 |
| hsa-miR-3620-5p | hsa_circ_0046430 |
| hsa-miR-3620-5p | hsa_circ_0050119 |
| hsa-miR-3620-5p | hsa_circ_0008030 |
| hsa-miR-3620-5p | hsa_circ_0000950 |
| hsa-miR-3620-5p | hsa_circ_0092297 |
| hsa-miR-3620-5p | hsa_circ_0060904 |
| hsa-miR-3620-5p | hsa_circ_0061052 |
| hsa-miR-3620-5p | hsa_circ_0092283 |
| hsa-miR-3620-5p | hsa_circ_0064136 |
| hsa-miR-3620-5p | hsa_circ_0066444 |
| hsa-miR-3620-5p | hsa_circ_0004276 |
| hsa-miR-3620-5p | hsa_circ_0001394 |
| hsa-miR-3620-5p | hsa_circ_0070467 |
| hsa-miR-3620-5p | hsa_circ_0001472 |
| hsa-miR-3620-5p | hsa_circ_0002490 |
| hsa-miR-3620-5p | hsa_circ_0001573 |
| hsa-miR-3620-5p | hsa_circ_0007132 |
| hsa-miR-3620-5p | hsa_circ_0004381 |
| hsa-miR-3620-5p | hsa_circ_0002755 |
| hsa-miR-3620-5p | hsa_circ_0006357 |
| hsa-miR-3620-5p | hsa_circ_0002094 |
| hsa-miR-3620-5p | hsa_circ_0001947 |
| hsa-miR-3620-3p | hsa_circ_0034189 |
| hsa-miR-3620-3p | hsa_circ_0046430 |
| hsa-miR-3620-3p | hsa_circ_0007132 |
| hsa-miR-3621 | hsa_circ_0006649 |
| hsa-miR-3621 | hsa_circ_0003854 |
| hsa-miR-3621 | hsa_circ_0061052 |
| hsa-miR-3621 | hsa_circ_0001247 |
| hsa-miR-3621 | hsa_circ_0081343 |
| hsa-miR-3621 | hsa_circ_0002094 |
| hsa-miR-3622a-5p | hsa_circ_0007009 |
| hsa-miR-3622a-5p | hsa_circ_0000973 |
| hsa-miR-3622a-5p | hsa_circ_0064136 |
| hsa-miR-3622a-5p | hsa_circ_0003602 |
| hsa-miR-3622a-5p | hsa_circ_0001400 |
| hsa-miR-3622a-3p | hsa_circ_0020594 |
| hsa-miR-3622b-5p | hsa_circ_0046430 |
| hsa-miR-3622b-5p | hsa_circ_0001549 |
| hsa-miR-3648 | hsa_circ_0014132 |
| hsa-miR-3648 | hsa_circ_0045890 |
| hsa-miR-3648 | hsa_circ_0003854 |
| hsa-miR-3648 | hsa_circ_0001400 |
| hsa-miR-3648 | hsa_circ_0007132 |
| hsa-miR-3648 | hsa_circ_0001708 |
| hsa-miR-3648 | hsa_circ_0081423 |
| hsa-miR-3648 | hsa_circ_0084429 |
| hsa-miR-3648 | hsa_circ_0006022 |
| hsa-miR-3651 | hsa_circ_0007846 |
| hsa-miR-3651 | hsa_circ_0061936 |
| hsa-miR-3651 | hsa_circ_0007132 |
| hsa-miR-3652 | hsa_circ_0000417 |
| hsa-miR-3656 | hsa_circ_0022383 |
| hsa-miR-3656 | hsa_circ_0007846 |
| hsa-miR-3656 | hsa_circ_0004712 |
| hsa-miR-3661 | hsa_circ_0003315 |
| hsa-miR-3661 | hsa_circ_0045890 |
| hsa-miR-3661 | hsa_circ_0045905 |
| hsa-miR-3663-5p | hsa_circ_0002402 |
| hsa-miR-3663-5p | hsa_circ_0003379 |
| hsa-miR-3663-5p | hsa_circ_0022723 |
| hsa-miR-3663-5p | hsa_circ_0040823 |
| hsa-miR-3663-5p | hsa_circ_0000950 |
| hsa-miR-3663-5p | hsa_circ_0002805 |
| hsa-miR-3663-5p | hsa_circ_0008583 |
| hsa-miR-3663-5p | hsa_circ_0003340 |
| hsa-miR-3663-3p | hsa_circ_0058522 |
| hsa-miR-3663-3p | hsa_circ_0007132 |
| hsa-miR-3665 | hsa_circ_0007846 |
| hsa-miR-3665 | hsa_circ_0004712 |
| hsa-miR-3677-5p | hsa_circ_0003557 |
| hsa-miR-3677-5p | hsa_circ_0045890 |
| hsa-miR-3677-5p | hsa_circ_0002805 |
| hsa-miR-3677-5p | hsa_circ_0061179 |
| hsa-miR-3677-5p | hsa_circ_0092299 |
| hsa-miR-3677-5p | hsa_circ_0006884 |
| hsa-miR-3677-5p | hsa_circ_0087288 |
| hsa-miR-3677-3p | hsa_circ_0007372 |
| hsa-miR-3677-3p | hsa_circ_0034189 |
| hsa-miR-3677-3p | hsa_circ_0000854 |
| hsa-miR-3677-3p | hsa_circ_0048025 |
| hsa-miR-3677-3p | hsa_circ_0050119 |
| hsa-miR-3677-3p | hsa_circ_0003854 |
| hsa-miR-3677-3p | hsa_circ_0004218 |
| hsa-miR-3677-3p | hsa_circ_0002805 |
| hsa-miR-3677-3p | hsa_circ_0061179 |
| hsa-miR-3677-3p | hsa_circ_0061936 |
| hsa-miR-3677-3p | hsa_circ_0092299 |
| hsa-miR-3677-3p | hsa_circ_0092283 |
| hsa-miR-3677-3p | hsa_circ_0001577 |
| hsa-miR-3677-3p | hsa_circ_0001578 |
| hsa-miR-3677-3p | hsa_circ_0007132 |
| hsa-miR-3677-3p | hsa_circ_0004381 |
| hsa-miR-3677-3p | hsa_circ_0081343 |
| hsa-miR-3677-3p | hsa_circ_0005630 |
| hsa-miR-3678-3p | hsa_circ_0006649 |
| hsa-miR-3678-3p | hsa_circ_0092125 |
| hsa-miR-3679-5p | hsa_circ_0000213 |
| hsa-miR-3679-5p | hsa_circ_0052131 |
| hsa-miR-3679-5p | hsa_circ_0001187 |
| hsa-miR-3679-5p | hsa_circ_0008078 |
| hsa-miR-3679-5p | hsa_circ_0087855 |
| hsa-miR-3679-5p | hsa_circ_0008812 |
| hsa-miR-3679-5p | hsa_circ_0006174 |
| hsa-miR-3679-5p | hsa_circ_0087861 |
| hsa-miR-3679-5p | hsa_circ_0087862 |
| hsa-miR-3681-3p | hsa_circ_0007132 |
| hsa-miR-3687 | hsa_circ_0007015 |
| hsa-miR-3687 | hsa_circ_0003168 |
| hsa-miR-3687 | hsa_circ_0005571 |
| hsa-miR-3689a-5p | hsa_circ_0007137 |
| hsa-miR-3689a-5p | hsa_circ_0001658 |
| hsa-miR-3689a-3p | hsa_circ_0006735 |
| hsa-miR-3689a-3p | hsa_circ_0077765 |
| hsa-miR-3689a-3p | hsa_circ_0087305 |
| hsa-miR-3690 | hsa_circ_0092299 |
| hsa-miR-3690 | hsa_circ_0084552 |
| hsa-miR-3691-5p | hsa_circ_0045890 |
| hsa-miR-3691-3p | hsa_circ_0007132 |
| hsa-miR-3692-5p | hsa_circ_0003908 |
| hsa-miR-3692-5p | hsa_circ_0007372 |
| hsa-miR-3692-5p | hsa_circ_0000417 |
| hsa-miR-3692-5p | hsa_circ_0000462 |
| hsa-miR-3692-5p | hsa_circ_0002289 |
| hsa-miR-3692-5p | hsa_circ_0045890 |
| hsa-miR-3692-5p | hsa_circ_0008590 |
| hsa-miR-3692-5p | hsa_circ_0002805 |
| hsa-miR-3692-5p | hsa_circ_0061179 |
| hsa-miR-3692-5p | hsa_circ_0092277 |
| hsa-miR-3692-5p | hsa_circ_0076742 |
| hsa-miR-3692-5p | hsa_circ_0084789 |
| hsa-miR-3713 | hsa_circ_0001394 |
| hsa-miR-3714 | hsa_circ_0004692 |
| hsa-miR-3714 | hsa_circ_0065147 |
| hsa-miR-3714 | hsa_circ_0065149 |
| hsa-miR-3714 | hsa_circ_0002569 |
| hsa-miR-3180 | hsa_circ_0048025 |
| hsa-miR-3180 | hsa_circ_0004003 |
| hsa-miR-3180 | hsa_circ_0004891 |
| hsa-miR-3180 | hsa_circ_0005571 |
| hsa-miR-3180 | hsa_circ_0092297 |
| hsa-miR-3180 | hsa_circ_0069748 |
| hsa-miR-3907 | hsa_circ_0003379 |
| hsa-miR-3689b-5p | hsa_circ_0007137 |
| hsa-miR-3689b-5p | hsa_circ_0001658 |
| hsa-miR-3689b-3p | hsa_circ_0074816 |
| hsa-miR-3689b-3p | hsa_circ_0074817 |
| hsa-miR-3689b-3p | hsa_circ_0077765 |
| hsa-miR-3909 | hsa_circ_0031933 |
| hsa-miR-3909 | hsa_circ_0092297 |
| hsa-miR-3909 | hsa_circ_0092299 |
| hsa-miR-3909 | hsa_circ_0092283 |
| hsa-miR-3909 | hsa_circ_0001829 |
| hsa-miR-3909 | hsa_circ_0006566 |
| hsa-miR-3911 | hsa_circ_0000417 |
| hsa-miR-3911 | hsa_circ_0040823 |
| hsa-miR-3911 | hsa_circ_0040921 |
| hsa-miR-3911 | hsa_circ_0061936 |
| hsa-miR-3911 | hsa_circ_0005954 |
| hsa-miR-3911 | hsa_circ_0087305 |
| hsa-miR-3916 | hsa_circ_0007437 |
| hsa-miR-3916 | hsa_circ_0007693 |
| hsa-miR-3916 | hsa_circ_0012151 |
| hsa-miR-3916 | hsa_circ_0075748 |
| hsa-miR-3918 | hsa_circ_0070467 |
| hsa-miR-3919 | hsa_circ_0005371 |
| hsa-miR-3150b-5p | hsa_circ_0046292 |
| hsa-miR-3150b-5p | hsa_circ_0007132 |
| hsa-miR-3921 | hsa_circ_0003168 |
| hsa-miR-3922-5p | hsa_circ_0002402 |
| hsa-miR-3922-5p | hsa_circ_0030051 |
| hsa-miR-3922-5p | hsa_circ_0043278 |
| hsa-miR-3922-5p | hsa_circ_0003854 |
| hsa-miR-3922-5p | hsa_circ_0092299 |
| hsa-miR-3928-3p | hsa_circ_0003854 |
| hsa-miR-3929 | hsa_circ_0007015 |
| hsa-miR-3934-5p | hsa_circ_0001092 |
| hsa-miR-3937 | hsa_circ_0031933 |
| hsa-miR-3937 | hsa_circ_0001472 |
| hsa-miR-3937 | hsa_circ_0001756 |
| hsa-miR-3937 | hsa_circ_0087288 |
| hsa-miR-3940-5p | hsa_circ_0000650 |
| hsa-miR-3940-5p | hsa_circ_0036768 |
| hsa-miR-3940-5p | hsa_circ_0007846 |
| hsa-miR-3940-5p | hsa_circ_0050119 |
| hsa-miR-3940-5p | hsa_circ_0008030 |
| hsa-miR-3940-5p | hsa_circ_0092297 |
| hsa-miR-3940-5p | hsa_circ_0071311 |
| hsa-miR-3940-5p | hsa_circ_0084615 |
| hsa-miR-3940-3p | hsa_circ_0016979 |
| hsa-miR-3940-3p | hsa_circ_0003854 |
| hsa-miR-3940-3p | hsa_circ_0092299 |
| hsa-miR-3940-3p | hsa_circ_0092283 |
| hsa-miR-3940-3p | hsa_circ_0006528 |
| hsa-miR-3940-3p | hsa_circ_0007132 |
| hsa-miR-3943 | hsa_circ_0000690 |
| hsa-miR-3943 | hsa_circ_0045905 |
| hsa-miR-3944-5p | hsa_circ_0007132 |
| hsa-miR-3944-3p | hsa_circ_0000048 |
| hsa-miR-3944-3p | hsa_circ_0017446 |
| hsa-miR-3944-3p | hsa_circ_0000206 |
| hsa-miR-3944-3p | hsa_circ_0006608 |
| hsa-miR-3944-3p | hsa_circ_0022383 |
| hsa-miR-3944-3p | hsa_circ_0006597 |
| hsa-miR-3944-3p | hsa_circ_0002564 |
| hsa-miR-3944-3p | hsa_circ_0002289 |
| hsa-miR-3944-3p | hsa_circ_0034189 |
| hsa-miR-3944-3p | hsa_circ_0040823 |
| hsa-miR-3944-3p | hsa_circ_0046292 |
| hsa-miR-3944-3p | hsa_circ_0046430 |
| hsa-miR-3944-3p | hsa_circ_0002926 |
| hsa-miR-3944-3p | hsa_circ_0050119 |
| hsa-miR-3944-3p | hsa_circ_0008030 |
| hsa-miR-3944-3p | hsa_circ_0002805 |
| hsa-miR-3944-3p | hsa_circ_0061179 |
| hsa-miR-3944-3p | hsa_circ_0004872 |
| hsa-miR-3944-3p | hsa_circ_0092283 |
| hsa-miR-3944-3p | hsa_circ_0001394 |
| hsa-miR-3944-3p | hsa_circ_0007132 |
| hsa-miR-3944-3p | hsa_circ_0092125 |
| hsa-miR-1268b | hsa_circ_0007015 |
| hsa-miR-1268b | hsa_circ_0006254 |
| hsa-miR-1268b | hsa_circ_0003768 |
| hsa-miR-1268b | hsa_circ_0022383 |
| hsa-miR-1268b | hsa_circ_0000417 |
| hsa-miR-1268b | hsa_circ_0029405 |
| hsa-miR-1268b | hsa_circ_0033144 |
| hsa-miR-1268b | hsa_circ_0007846 |
| hsa-miR-1268b | hsa_circ_0046430 |
| hsa-miR-1268b | hsa_circ_0048025 |
| hsa-miR-1268b | hsa_circ_0049657 |
| hsa-miR-1268b | hsa_circ_0000973 |
| hsa-miR-1268b | hsa_circ_0002805 |
| hsa-miR-1268b | hsa_circ_0061179 |
| hsa-miR-1268b | hsa_circ_0070467 |
| hsa-miR-1268b | hsa_circ_0007132 |
| hsa-miR-1268b | hsa_circ_0009092 |
| hsa-miR-1268b | hsa_circ_0001708 |
| hsa-miR-1268b | hsa_circ_0002755 |
| hsa-miR-1268b | hsa_circ_0001748 |
| hsa-miR-1268b | hsa_circ_0002094 |
| hsa-miR-1268b | hsa_circ_0005630 |
| hsa-miR-1268b | hsa_circ_0087305 |
| hsa-miR-1268b | hsa_circ_0087855 |
| hsa-miR-1268b | hsa_circ_0008812 |
| hsa-miR-1268b | hsa_circ_0006174 |
| hsa-miR-1268b | hsa_circ_0087861 |
| hsa-miR-1268b | hsa_circ_0087862 |
| hsa-miR-1268b | hsa_circ_0001910 |
| hsa-miR-4417 | hsa_circ_0022383 |
| hsa-miR-4417 | hsa_circ_0031933 |
| hsa-miR-4417 | hsa_circ_0092297 |
| hsa-miR-4417 | hsa_circ_0060043 |
| hsa-miR-4417 | hsa_circ_0007132 |
| hsa-miR-4417 | hsa_circ_0004381 |
| hsa-miR-4417 | hsa_circ_0006022 |
| hsa-miR-4418 | hsa_circ_0092299 |
| hsa-miR-4433a-5p | hsa_circ_0011571 |
| hsa-miR-4433a-5p | hsa_circ_0011572 |
| hsa-miR-4433a-5p | hsa_circ_0092299 |
| hsa-miR-4433a-3p | hsa_circ_0000650 |
| hsa-miR-4433a-3p | hsa_circ_0036768 |
| hsa-miR-4433a-3p | hsa_circ_0048025 |
| hsa-miR-4433a-3p | hsa_circ_0004381 |
| hsa-miR-4435 | hsa_circ_0092299 |
| hsa-miR-4440 | hsa_circ_0014132 |
| hsa-miR-4440 | hsa_circ_0040823 |
| hsa-miR-4440 | hsa_circ_0060043 |
| hsa-miR-4440 | hsa_circ_0087288 |
| hsa-miR-4440 | hsa_circ_0007351 |
| hsa-miR-4446-3p | hsa_circ_0046430 |
| hsa-miR-4446-3p | hsa_circ_0092297 |
| hsa-miR-4446-3p | hsa_circ_0092299 |
| hsa-miR-4446-3p | hsa_circ_0007132 |
| hsa-miR-4449 | hsa_circ_0007015 |
| hsa-miR-4449 | hsa_circ_0017446 |
| hsa-miR-4449 | hsa_circ_0000206 |
| hsa-miR-4449 | hsa_circ_0018909 |
| hsa-miR-4449 | hsa_circ_0003379 |
| hsa-miR-4449 | hsa_circ_0038799 |
| hsa-miR-4449 | hsa_circ_0002926 |
| hsa-miR-4449 | hsa_circ_0050119 |
| hsa-miR-4449 | hsa_circ_0092297 |
| hsa-miR-4449 | hsa_circ_0003854 |
| hsa-miR-4449 | hsa_circ_0002805 |
| hsa-miR-4449 | hsa_circ_0061179 |
| hsa-miR-4449 | hsa_circ_0092299 |
| hsa-miR-4449 | hsa_circ_0007132 |
| hsa-miR-4449 | hsa_circ_0004381 |
| hsa-miR-4450 | hsa_circ_0006552 |
| hsa-miR-4450 | hsa_circ_0005630 |
| hsa-miR-4459 | hsa_circ_0017248 |
| hsa-miR-4459 | hsa_circ_0006649 |
| hsa-miR-4459 | hsa_circ_0020005 |
| hsa-miR-4459 | hsa_circ_0003379 |
| hsa-miR-4459 | hsa_circ_0022383 |
| hsa-miR-4459 | hsa_circ_0000417 |
| hsa-miR-4459 | hsa_circ_0029405 |
| hsa-miR-4459 | hsa_circ_0003713 |
| hsa-miR-4459 | hsa_circ_0007846 |
| hsa-miR-4459 | hsa_circ_0049657 |
| hsa-miR-4459 | hsa_circ_0050119 |
| hsa-miR-4459 | hsa_circ_0000936 |
| hsa-miR-4459 | hsa_circ_0000973 |
| hsa-miR-4459 | hsa_circ_0006790 |
| hsa-miR-4459 | hsa_circ_0007385 |
| hsa-miR-4459 | hsa_circ_0064136 |
| hsa-miR-4459 | hsa_circ_0065284 |
| hsa-miR-4459 | hsa_circ_0008583 |
| hsa-miR-4459 | hsa_circ_0001400 |
| hsa-miR-4459 | hsa_circ_0069748 |
| hsa-miR-4459 | hsa_circ_0073379 |
| hsa-miR-4459 | hsa_circ_0005540 |
| hsa-miR-4459 | hsa_circ_0002037 |
| hsa-miR-4459 | hsa_circ_0004712 |
| hsa-miR-4459 | hsa_circ_0002094 |
| hsa-miR-4459 | hsa_circ_0005630 |
| hsa-miR-4459 | hsa_circ_0001829 |
| hsa-miR-4459 | hsa_circ_0006566 |
| hsa-miR-3135b | hsa_circ_0018909 |
| hsa-miR-3135b | hsa_circ_0006735 |
| hsa-miR-3135b | hsa_circ_0040823 |
| hsa-miR-3135b | hsa_circ_0046430 |
| hsa-miR-3135b | hsa_circ_0008030 |
| hsa-miR-4462 | hsa_circ_0000213 |
| hsa-miR-4462 | hsa_circ_0028190 |
| hsa-miR-4462 | hsa_circ_0045890 |
| hsa-miR-4462 | hsa_circ_0046430 |
| hsa-miR-4462 | hsa_circ_0002805 |
| hsa-miR-4462 | hsa_circ_0061179 |
| hsa-miR-4463 | hsa_circ_0000417 |
| hsa-miR-4465 | hsa_circ_0005630 |
| hsa-miR-4466 | hsa_circ_0006649 |
| hsa-miR-4466 | hsa_circ_0003557 |
| hsa-miR-4466 | hsa_circ_0000650 |
| hsa-miR-4466 | hsa_circ_0036768 |
| hsa-miR-4466 | hsa_circ_0045890 |
| hsa-miR-4466 | hsa_circ_0048025 |
| hsa-miR-4466 | hsa_circ_0004218 |
| hsa-miR-4466 | hsa_circ_0061052 |
| hsa-miR-4466 | hsa_circ_0004470 |
| hsa-miR-4466 | hsa_circ_0007132 |
| hsa-miR-4466 | hsa_circ_0087305 |
| hsa-miR-4467 | hsa_circ_0007132 |
| hsa-miR-4467 | hsa_circ_0007145 |
| hsa-miR-4469 | hsa_circ_0007846 |
| hsa-miR-4469 | hsa_circ_0049657 |
| hsa-miR-4469 | hsa_circ_0049998 |
| hsa-miR-4469 | hsa_circ_0008261 |
| hsa-miR-4469 | hsa_circ_0072437 |
| hsa-miR-4474-3p | hsa_circ_0001658 |
| hsa-miR-4476 | hsa_circ_0029633 |
| hsa-miR-4476 | hsa_circ_0071311 |
| hsa-miR-3689c | hsa_circ_0074816 |
| hsa-miR-3689c | hsa_circ_0074817 |
| hsa-miR-3689c | hsa_circ_0077765 |
| hsa-miR-3689e | hsa_circ_0007137 |
| hsa-miR-3689e | hsa_circ_0001658 |
| hsa-miR-4479 | hsa_circ_0018909 |
| hsa-miR-4479 | hsa_circ_0006649 |
| hsa-miR-4479 | hsa_circ_0003168 |
| hsa-miR-4479 | hsa_circ_0050119 |
| hsa-miR-4479 | hsa_circ_0002805 |
| hsa-miR-4479 | hsa_circ_0001756 |
| hsa-miR-3155b | hsa_circ_0003557 |
| hsa-miR-4483 | hsa_circ_0046430 |
| hsa-miR-4486 | hsa_circ_0007132 |
| hsa-miR-4488 | hsa_circ_0007846 |
| hsa-miR-4488 | hsa_circ_0004003 |
| hsa-miR-4488 | hsa_circ_0004891 |
| hsa-miR-4488 | hsa_circ_0004218 |
| hsa-miR-4488 | hsa_circ_0001394 |
| hsa-miR-4488 | hsa_circ_0007132 |
| hsa-miR-4492 | hsa_circ_0007846 |
| hsa-miR-4492 | hsa_circ_0081343 |
| hsa-miR-4494 | hsa_circ_0003315 |
| hsa-miR-4494 | hsa_circ_0092297 |
| hsa-miR-4498 | hsa_circ_0009349 |
| hsa-miR-4498 | hsa_circ_0008102 |
| hsa-miR-4498 | hsa_circ_0007146 |
| hsa-miR-4498 | hsa_circ_0046430 |
| hsa-miR-4498 | hsa_circ_0008030 |
| hsa-miR-4498 | hsa_circ_0003854 |
| hsa-miR-4498 | hsa_circ_0071311 |
| hsa-miR-4498 | hsa_circ_0007132 |
| hsa-miR-4505 | hsa_circ_0002094 |
| hsa-miR-2392 | hsa_circ_0092297 |
| hsa-miR-2392 | hsa_circ_0005630 |
| hsa-miR-4507 | hsa_circ_0040823 |
| hsa-miR-4507 | hsa_circ_0046430 |
| hsa-miR-4507 | hsa_circ_0061052 |
| hsa-miR-4507 | hsa_circ_0070040 |
| hsa-miR-4507 | hsa_circ_0001573 |
| hsa-miR-4507 | hsa_circ_0007132 |
| hsa-miR-4507 | hsa_circ_0087855 |
| hsa-miR-4507 | hsa_circ_0008812 |
| hsa-miR-4507 | hsa_circ_0006174 |
| hsa-miR-4507 | hsa_circ_0087861 |
| hsa-miR-4507 | hsa_circ_0087862 |
| hsa-miR-4508 | hsa_circ_0007846 |
| hsa-miR-4508 | hsa_circ_0050119 |
| hsa-miR-4508 | hsa_circ_0081343 |
| hsa-miR-4512 | hsa_circ_0048025 |
| hsa-miR-4513 | hsa_circ_0002490 |
| hsa-miR-4515 | hsa_circ_0020594 |
| hsa-miR-4515 | hsa_circ_0033144 |
| hsa-miR-4515 | hsa_circ_0045890 |
| hsa-miR-4515 | hsa_circ_0008030 |
| hsa-miR-4516 | hsa_circ_0001394 |
| hsa-miR-4518 | hsa_circ_0003557 |
| hsa-miR-4518 | hsa_circ_0045890 |
| hsa-miR-4518 | hsa_circ_0004692 |
| hsa-miR-4519 | hsa_circ_0006597 |
| hsa-miR-1269b | hsa_circ_0034189 |
| hsa-miR-1269b | hsa_circ_0042799 |
| hsa-miR-4525 | hsa_circ_0000996 |
| hsa-miR-4526 | hsa_circ_0004218 |
| hsa-miR-4526 | hsa_circ_0002805 |
| hsa-miR-4526 | hsa_circ_0061179 |
| hsa-miR-4530 | hsa_circ_0003168 |
| hsa-miR-4530 | hsa_circ_0092337 |
| hsa-miR-4530 | hsa_circ_0002805 |
| hsa-miR-4530 | hsa_circ_0061179 |
| hsa-miR-4532 | hsa_circ_0092297 |
| hsa-miR-4532 | hsa_circ_0092299 |
| hsa-miR-4532 | hsa_circ_0001400 |
| hsa-miR-4532 | hsa_circ_0003340 |
| hsa-miR-4532 | hsa_circ_0081423 |
| hsa-miR-4532 | hsa_circ_0001756 |
| hsa-miR-4533 | hsa_circ_0004519 |
| hsa-miR-4533 | hsa_circ_0000936 |
| hsa-miR-4533 | hsa_circ_0073379 |
| hsa-miR-4533 | hsa_circ_0005540 |
| hsa-miR-4533 | hsa_circ_0005519 |
| hsa-miR-4535 | hsa_circ_0022383 |
| hsa-miR-4535 | hsa_circ_0001573 |
| hsa-miR-1587 | hsa_circ_0000497 |
| hsa-miR-1587 | hsa_circ_0040823 |
| hsa-miR-1587 | hsa_circ_0060904 |
| hsa-miR-1587 | hsa_circ_0064136 |
| hsa-miR-1587 | hsa_circ_0001573 |
| hsa-miR-4538 | hsa_circ_0007146 |
| hsa-miR-4538 | hsa_circ_0000682 |
| hsa-miR-4538 | hsa_circ_0008030 |
| hsa-miR-4538 | hsa_circ_0058058 |
| hsa-miR-4539 | hsa_circ_0064136 |
| hsa-miR-4539 | hsa_circ_0007132 |
| hsa-miR-3960 | hsa_circ_0009732 |
| hsa-miR-3960 | hsa_circ_0006608 |
| hsa-miR-3960 | hsa_circ_0018909 |
| hsa-miR-3960 | hsa_circ_0003379 |
| hsa-miR-3960 | hsa_circ_0000375 |
| hsa-miR-3960 | hsa_circ_0007846 |
| hsa-miR-3960 | hsa_circ_0040823 |
| hsa-miR-3960 | hsa_circ_0046430 |
| hsa-miR-3960 | hsa_circ_0048025 |
| hsa-miR-3960 | hsa_circ_0006877 |
| hsa-miR-3960 | hsa_circ_0049998 |
| hsa-miR-3960 | hsa_circ_0092277 |
| hsa-miR-3960 | hsa_circ_0001394 |
| hsa-miR-3960 | hsa_circ_0001400 |
| hsa-miR-3960 | hsa_circ_0071311 |
| hsa-miR-3960 | hsa_circ_0007132 |
| hsa-miR-3960 | hsa_circ_0007145 |
| hsa-miR-3960 | hsa_circ_0001756 |
| hsa-miR-3960 | hsa_circ_0002094 |
| hsa-miR-3960 | hsa_circ_0005630 |
| hsa-miR-3960 | hsa_circ_0001829 |
| hsa-miR-3972 | hsa_circ_0005782 |
| hsa-miR-3972 | hsa_circ_0020594 |
| hsa-miR-3972 | hsa_circ_0007146 |
| hsa-miR-3972 | hsa_circ_0046430 |
| hsa-miR-3972 | hsa_circ_0002926 |
| hsa-miR-3972 | hsa_circ_0006877 |
| hsa-miR-3972 | hsa_circ_0050119 |
| hsa-miR-3972 | hsa_circ_0092297 |
| hsa-miR-3972 | hsa_circ_0092283 |
| hsa-miR-3972 | hsa_circ_0007132 |
| hsa-miR-3972 | hsa_circ_0002755 |
| hsa-miR-3972 | hsa_circ_0001756 |
| hsa-miR-3972 | hsa_circ_0087288 |
| hsa-miR-3978 | hsa_circ_0048025 |
| hsa-miR-4632-5p | hsa_circ_0009349 |
| hsa-miR-4632-5p | hsa_circ_0009581 |
| hsa-miR-4632-5p | hsa_circ_0006837 |
| hsa-miR-4632-5p | hsa_circ_0015004 |
| hsa-miR-4632-5p | hsa_circ_0007015 |
| hsa-miR-4632-5p | hsa_circ_0008102 |
| hsa-miR-4632-5p | hsa_circ_0003557 |
| hsa-miR-4632-5p | hsa_circ_0034189 |
| hsa-miR-4632-5p | hsa_circ_0000660 |
| hsa-miR-4632-5p | hsa_circ_0007146 |
| hsa-miR-4632-5p | hsa_circ_0007846 |
| hsa-miR-4632-5p | hsa_circ_0045890 |
| hsa-miR-4632-5p | hsa_circ_0046430 |
| hsa-miR-4632-5p | hsa_circ_0003854 |
| hsa-miR-4632-5p | hsa_circ_0004218 |
| hsa-miR-4632-5p | hsa_circ_0060904 |
| hsa-miR-4632-5p | hsa_circ_0061052 |
| hsa-miR-4632-5p | hsa_circ_0002805 |
| hsa-miR-4632-5p | hsa_circ_0004470 |
| hsa-miR-4632-5p | hsa_circ_0001394 |
| hsa-miR-4632-5p | hsa_circ_0070467 |
| hsa-miR-4632-5p | hsa_circ_0001472 |
| hsa-miR-4632-5p | hsa_circ_0001549 |
| hsa-miR-4632-5p | hsa_circ_0007132 |
| hsa-miR-4632-5p | hsa_circ_0009092 |
| hsa-miR-4632-5p | hsa_circ_0006566 |
| hsa-miR-4632-3p | hsa_circ_0009349 |
| hsa-miR-4632-3p | hsa_circ_0017438 |
| hsa-miR-4632-3p | hsa_circ_0033144 |
| hsa-miR-4632-3p | hsa_circ_0002926 |
| hsa-miR-4632-3p | hsa_circ_0004692 |
| hsa-miR-4632-3p | hsa_circ_0067913 |
| hsa-miR-4632-3p | hsa_circ_0067919 |
| hsa-miR-4632-3p | hsa_circ_0005362 |
| hsa-miR-4632-3p | hsa_circ_0001472 |
| hsa-miR-4632-3p | hsa_circ_0002094 |
| hsa-miR-4632-3p | hsa_circ_0087305 |
| hsa-miR-4634 | hsa_circ_0003278 |
| hsa-miR-4638-5p | hsa_circ_0045905 |
| hsa-miR-4638-5p | hsa_circ_0008261 |
| hsa-miR-4638-3p | hsa_circ_0003379 |
| hsa-miR-4638-3p | hsa_circ_0003855 |
| hsa-miR-4638-3p | hsa_circ_0033144 |
| hsa-miR-4638-3p | hsa_circ_0041050 |
| hsa-miR-4638-3p | hsa_circ_0046430 |
| hsa-miR-4638-3p | hsa_circ_0058522 |
| hsa-miR-4638-3p | hsa_circ_0002805 |
| hsa-miR-4638-3p | hsa_circ_0061179 |
| hsa-miR-4638-3p | hsa_circ_0008583 |
| hsa-miR-4638-3p | hsa_circ_0001400 |
| hsa-miR-4638-3p | hsa_circ_0005630 |
| hsa-miR-4638-3p | hsa_circ_0001829 |
| hsa-miR-4640-5p | hsa_circ_0009581 |
| hsa-miR-4640-5p | hsa_circ_0006837 |
| hsa-miR-4640-5p | hsa_circ_0005782 |
| hsa-miR-4640-5p | hsa_circ_0002402 |
| hsa-miR-4640-5p | hsa_circ_0007015 |
| hsa-miR-4640-5p | hsa_circ_0017289 |
| hsa-miR-4640-5p | hsa_circ_0005090 |
| hsa-miR-4640-5p | hsa_circ_0006254 |
| hsa-miR-4640-5p | hsa_circ_0003768 |
| hsa-miR-4640-5p | hsa_circ_0022723 |
| hsa-miR-4640-5p | hsa_circ_0000417 |
| hsa-miR-4640-5p | hsa_circ_0003489 |
| hsa-miR-4640-5p | hsa_circ_0000565 |
| hsa-miR-4640-5p | hsa_circ_0035381 |
| hsa-miR-4640-5p | hsa_circ_0007846 |
| hsa-miR-4640-5p | hsa_circ_0002696 |
| hsa-miR-4640-5p | hsa_circ_0040039 |
| hsa-miR-4640-5p | hsa_circ_0045890 |
| hsa-miR-4640-5p | hsa_circ_0046430 |
| hsa-miR-4640-5p | hsa_circ_0047347 |
| hsa-miR-4640-5p | hsa_circ_0048025 |
| hsa-miR-4640-5p | hsa_circ_0004003 |
| hsa-miR-4640-5p | hsa_circ_0054144 |
| hsa-miR-4640-5p | hsa_circ_0006006 |
| hsa-miR-4640-5p | hsa_circ_0004218 |
| hsa-miR-4640-5p | hsa_circ_0061052 |
| hsa-miR-4640-5p | hsa_circ_0002805 |
| hsa-miR-4640-5p | hsa_circ_0061179 |
| hsa-miR-4640-5p | hsa_circ_0092299 |
| hsa-miR-4640-5p | hsa_circ_0004470 |
| hsa-miR-4640-5p | hsa_circ_0002077 |
| hsa-miR-4640-5p | hsa_circ_0001400 |
| hsa-miR-4640-5p | hsa_circ_0072857 |
| hsa-miR-4640-5p | hsa_circ_0007132 |
| hsa-miR-4640-5p | hsa_circ_0004381 |
| hsa-miR-4640-5p | hsa_circ_0003340 |
| hsa-miR-4640-5p | hsa_circ_0081343 |
| hsa-miR-4640-5p | hsa_circ_0001756 |
| hsa-miR-4640-5p | hsa_circ_0005630 |
| hsa-miR-4640-5p | hsa_circ_0084429 |
| hsa-miR-4640-5p | hsa_circ_0006566 |
| hsa-miR-4640-5p | hsa_circ_0087305 |
| hsa-miR-4640-5p | hsa_circ_0007685 |
| hsa-miR-4640-5p | hsa_circ_0001910 |
| hsa-miR-4640-3p | hsa_circ_0000417 |
| hsa-miR-4640-3p | hsa_circ_0040921 |
| hsa-miR-4640-3p | hsa_circ_0050119 |
| hsa-miR-4641 | hsa_circ_0009349 |
| hsa-miR-4642 | hsa_circ_0000682 |
| hsa-miR-4642 | hsa_circ_0049888 |
| hsa-miR-4642 | hsa_circ_0007132 |
| hsa-miR-4646-5p | hsa_circ_0033144 |
| hsa-miR-4646-5p | hsa_circ_0045890 |
| hsa-miR-4646-5p | hsa_circ_0049998 |
| hsa-miR-4646-5p | hsa_circ_0006357 |
| hsa-miR-4646-3p | hsa_circ_0003768 |
| hsa-miR-4646-3p | hsa_circ_0040921 |
| hsa-miR-4646-3p | hsa_circ_0004692 |
| hsa-miR-4647 | hsa_circ_0064136 |
| hsa-miR-4649-5p | hsa_circ_0000048 |
| hsa-miR-4649-5p | hsa_circ_0007437 |
| hsa-miR-4649-5p | hsa_circ_0007693 |
| hsa-miR-4649-5p | hsa_circ_0012151 |
| hsa-miR-4649-5p | hsa_circ_0017639 |
| hsa-miR-4649-5p | hsa_circ_0003168 |
| hsa-miR-4649-5p | hsa_circ_0022383 |
| hsa-miR-4649-5p | hsa_circ_0022392 |
| hsa-miR-4649-5p | hsa_circ_0022723 |
| hsa-miR-4649-5p | hsa_circ_0000375 |
| hsa-miR-4649-5p | hsa_circ_0000417 |
| hsa-miR-4649-5p | hsa_circ_0031933 |
| hsa-miR-4649-5p | hsa_circ_0036763 |
| hsa-miR-4649-5p | hsa_circ_0000650 |
| hsa-miR-4649-5p | hsa_circ_0036768 |
| hsa-miR-4649-5p | hsa_circ_0007146 |
| hsa-miR-4649-5p | hsa_circ_0046430 |
| hsa-miR-4649-5p | hsa_circ_0048025 |
| hsa-miR-4649-5p | hsa_circ_0008261 |
| hsa-miR-4649-5p | hsa_circ_0000996 |
| hsa-miR-4649-5p | hsa_circ_0058058 |
| hsa-miR-4649-5p | hsa_circ_0092299 |
| hsa-miR-4649-5p | hsa_circ_0063331 |
| hsa-miR-4649-5p | hsa_circ_0004692 |
| hsa-miR-4649-5p | hsa_circ_0065147 |
| hsa-miR-4649-5p | hsa_circ_0065149 |
| hsa-miR-4649-5p | hsa_circ_0002569 |
| hsa-miR-4649-5p | hsa_circ_0092277 |
| hsa-miR-4649-5p | hsa_circ_0072437 |
| hsa-miR-4649-5p | hsa_circ_0074371 |
| hsa-miR-4649-5p | hsa_circ_0001549 |
| hsa-miR-4649-5p | hsa_circ_0001573 |
| hsa-miR-4649-5p | hsa_circ_0007132 |
| hsa-miR-4649-5p | hsa_circ_0076742 |
| hsa-miR-4649-5p | hsa_circ_0004381 |
| hsa-miR-4649-5p | hsa_circ_0078522 |
| hsa-miR-4649-5p | hsa_circ_0007145 |
| hsa-miR-4649-5p | hsa_circ_0001756 |
| hsa-miR-4649-5p | hsa_circ_0084615 |
| hsa-miR-4649-5p | hsa_circ_0007521 |
| hsa-miR-4649-5p | hsa_circ_0092125 |
| hsa-miR-4649-3p | hsa_circ_0003557 |
| hsa-miR-4649-3p | hsa_circ_0007967 |
| hsa-miR-4649-3p | hsa_circ_0002805 |
| hsa-miR-4649-3p | hsa_circ_0004692 |
| hsa-miR-4649-3p | hsa_circ_0005630 |
| hsa-miR-4651 | hsa_circ_0007009 |
| hsa-miR-4651 | hsa_circ_0022505 |
| hsa-miR-4651 | hsa_circ_0000417 |
| hsa-miR-4651 | hsa_circ_0004137 |
| hsa-miR-4651 | hsa_circ_0045890 |
| hsa-miR-4651 | hsa_circ_0046430 |
| hsa-miR-4651 | hsa_circ_0000854 |
| hsa-miR-4651 | hsa_circ_0048025 |
| hsa-miR-4651 | hsa_circ_0004003 |
| hsa-miR-4651 | hsa_circ_0006877 |
| hsa-miR-4651 | hsa_circ_0052131 |
| hsa-miR-4651 | hsa_circ_0003854 |
| hsa-miR-4651 | hsa_circ_0004218 |
| hsa-miR-4651 | hsa_circ_0004470 |
| hsa-miR-4651 | hsa_circ_0001394 |
| hsa-miR-4651 | hsa_circ_0002094 |
| hsa-miR-4651 | hsa_circ_0087288 |
| hsa-miR-4652-3p | hsa_circ_0005630 |
| hsa-miR-4654 | hsa_circ_0017639 |
| hsa-miR-4654 | hsa_circ_0000417 |
| hsa-miR-4654 | hsa_circ_0000996 |
| hsa-miR-4654 | hsa_circ_0006693 |
| hsa-miR-4654 | hsa_circ_0001417 |
| hsa-miR-4655-5p | hsa_circ_0015004 |
| hsa-miR-4655-5p | hsa_circ_0031933 |
| hsa-miR-4655-5p | hsa_circ_0007846 |
| hsa-miR-4655-5p | hsa_circ_0040823 |
| hsa-miR-4655-5p | hsa_circ_0046430 |
| hsa-miR-4655-5p | hsa_circ_0048025 |
| hsa-miR-4655-5p | hsa_circ_0000973 |
| hsa-miR-4655-5p | hsa_circ_0092297 |
| hsa-miR-4655-5p | hsa_circ_0001400 |
| hsa-miR-4655-5p | hsa_circ_0003340 |
| hsa-miR-4655-5p | hsa_circ_0009092 |
| hsa-miR-4655-5p | hsa_circ_0001708 |
| hsa-miR-4655-5p | hsa_circ_0001824 |
| hsa-miR-4655-5p | hsa_circ_0008934 |
| hsa-miR-4655-3p | hsa_circ_0022723 |
| hsa-miR-4655-3p | hsa_circ_0006597 |
| hsa-miR-4655-3p | hsa_circ_0046430 |
| hsa-miR-4655-3p | hsa_circ_0004003 |
| hsa-miR-4655-3p | hsa_circ_0004891 |
| hsa-miR-4655-3p | hsa_circ_0054086 |
| hsa-miR-4655-3p | hsa_circ_0002805 |
| hsa-miR-4655-3p | hsa_circ_0092283 |
| hsa-miR-4655-3p | hsa_circ_0070396 |
| hsa-miR-4655-3p | hsa_circ_0007132 |
| hsa-miR-4655-3p | hsa_circ_0003738 |
| hsa-miR-4655-3p | hsa_circ_0001708 |
| hsa-miR-4655-3p | hsa_circ_0087305 |
| hsa-miR-4656 | hsa_circ_0002402 |
| hsa-miR-4656 | hsa_circ_0015004 |
| hsa-miR-4656 | hsa_circ_0016863 |
| hsa-miR-4656 | hsa_circ_0016867 |
| hsa-miR-4656 | hsa_circ_0017289 |
| hsa-miR-4656 | hsa_circ_0003168 |
| hsa-miR-4656 | hsa_circ_0022723 |
| hsa-miR-4656 | hsa_circ_0000417 |
| hsa-miR-4656 | hsa_circ_0029405 |
| hsa-miR-4656 | hsa_circ_0006137 |
| hsa-miR-4656 | hsa_circ_0036763 |
| hsa-miR-4656 | hsa_circ_0000650 |
| hsa-miR-4656 | hsa_circ_0036768 |
| hsa-miR-4656 | hsa_circ_0007146 |
| hsa-miR-4656 | hsa_circ_0040921 |
| hsa-miR-4656 | hsa_circ_0046430 |
| hsa-miR-4656 | hsa_circ_0006877 |
| hsa-miR-4656 | hsa_circ_0008030 |
| hsa-miR-4656 | hsa_circ_0000950 |
| hsa-miR-4656 | hsa_circ_0003854 |
| hsa-miR-4656 | hsa_circ_0004218 |
| hsa-miR-4656 | hsa_circ_0061936 |
| hsa-miR-4656 | hsa_circ_0092299 |
| hsa-miR-4656 | hsa_circ_0003602 |
| hsa-miR-4656 | hsa_circ_0071311 |
| hsa-miR-4656 | hsa_circ_0007132 |
| hsa-miR-4656 | hsa_circ_0002755 |
| hsa-miR-4656 | hsa_circ_0082179 |
| hsa-miR-4656 | hsa_circ_0001748 |
| hsa-miR-4658 | hsa_circ_0000497 |
| hsa-miR-4658 | hsa_circ_0007846 |
| hsa-miR-4658 | hsa_circ_0069748 |
| hsa-miR-4660 | hsa_circ_0007132 |
| hsa-miR-4663 | hsa_circ_0002402 |
| hsa-miR-4663 | hsa_circ_0018909 |
| hsa-miR-4663 | hsa_circ_0003379 |
| hsa-miR-4663 | hsa_circ_0007146 |
| hsa-miR-4663 | hsa_circ_0045890 |
| hsa-miR-4663 | hsa_circ_0050119 |
| hsa-miR-4663 | hsa_circ_0001829 |
| hsa-miR-4664-5p | hsa_circ_0043522 |
| hsa-miR-4664-5p | hsa_circ_0070396 |
| hsa-miR-4664-5p | hsa_circ_0007132 |
| hsa-miR-4664-3p | hsa_circ_0045890 |
| hsa-miR-4664-3p | hsa_circ_0007132 |
| hsa-miR-4665-5p | hsa_circ_0009349 |
| hsa-miR-4665-5p | hsa_circ_0003379 |
| hsa-miR-4665-5p | hsa_circ_0022723 |
| hsa-miR-4665-5p | hsa_circ_0006735 |
| hsa-miR-4665-5p | hsa_circ_0046430 |
| hsa-miR-4665-5p | hsa_circ_0048025 |
| hsa-miR-4665-5p | hsa_circ_0052131 |
| hsa-miR-4665-5p | hsa_circ_0007609 |
| hsa-miR-4665-5p | hsa_circ_0061936 |
| hsa-miR-4665-5p | hsa_circ_0001400 |
| hsa-miR-4665-5p | hsa_circ_0073379 |
| hsa-miR-4665-5p | hsa_circ_0005540 |
| hsa-miR-4665-5p | hsa_circ_0004381 |
| hsa-miR-4665-5p | hsa_circ_0078522 |
| hsa-miR-4665-5p | hsa_circ_0001756 |
| hsa-miR-4665-3p | hsa_circ_0002402 |
| hsa-miR-4665-3p | hsa_circ_0007015 |
| hsa-miR-4665-3p | hsa_circ_0003168 |
| hsa-miR-4665-3p | hsa_circ_0003379 |
| hsa-miR-4665-3p | hsa_circ_0003557 |
| hsa-miR-4665-3p | hsa_circ_0029405 |
| hsa-miR-4665-3p | hsa_circ_0031933 |
| hsa-miR-4665-3p | hsa_circ_0007846 |
| hsa-miR-4665-3p | hsa_circ_0000682 |
| hsa-miR-4665-3p | hsa_circ_0000690 |
| hsa-miR-4665-3p | hsa_circ_0040039 |
| hsa-miR-4665-3p | hsa_circ_0004354 |
| hsa-miR-4665-3p | hsa_circ_0040823 |
| hsa-miR-4665-3p | hsa_circ_0040921 |
| hsa-miR-4665-3p | hsa_circ_0045890 |
| hsa-miR-4665-3p | hsa_circ_0048025 |
| hsa-miR-4665-3p | hsa_circ_0002926 |
| hsa-miR-4665-3p | hsa_circ_0050119 |
| hsa-miR-4665-3p | hsa_circ_0000973 |
| hsa-miR-4665-3p | hsa_circ_0092297 |
| hsa-miR-4665-3p | hsa_circ_0003854 |
| hsa-miR-4665-3p | hsa_circ_0061052 |
| hsa-miR-4665-3p | hsa_circ_0002805 |
| hsa-miR-4665-3p | hsa_circ_0061179 |
| hsa-miR-4665-3p | hsa_circ_0007609 |
| hsa-miR-4665-3p | hsa_circ_0092283 |
| hsa-miR-4665-3p | hsa_circ_0006884 |
| hsa-miR-4665-3p | hsa_circ_0070396 |
| hsa-miR-4665-3p | hsa_circ_0074816 |
| hsa-miR-4665-3p | hsa_circ_0074817 |
| hsa-miR-4665-3p | hsa_circ_0007132 |
| hsa-miR-4665-3p | hsa_circ_0076742 |
| hsa-miR-4665-3p | hsa_circ_0002755 |
| hsa-miR-4665-3p | hsa_circ_0005630 |
| hsa-miR-4665-3p | hsa_circ_0087305 |
| hsa-miR-4665-3p | hsa_circ_0001910 |
| hsa-miR-4665-3p | hsa_circ_0001934 |
| hsa-miR-4669 | hsa_circ_0000417 |
| hsa-miR-4669 | hsa_circ_0029405 |
| hsa-miR-4669 | hsa_circ_0030051 |
| hsa-miR-4669 | hsa_circ_0033144 |
| hsa-miR-4669 | hsa_circ_0046430 |
| hsa-miR-4669 | hsa_circ_0002926 |
| hsa-miR-4669 | hsa_circ_0001394 |
| hsa-miR-4669 | hsa_circ_0075748 |
| hsa-miR-4669 | hsa_circ_0003340 |
| hsa-miR-4673 | hsa_circ_0002402 |
| hsa-miR-4673 | hsa_circ_0017248 |
| hsa-miR-4673 | hsa_circ_0004519 |
| hsa-miR-4673 | hsa_circ_0092337 |
| hsa-miR-4673 | hsa_circ_0092299 |
| hsa-miR-4673 | hsa_circ_0071311 |
| hsa-miR-4673 | hsa_circ_0007132 |
| hsa-miR-4673 | hsa_circ_0002094 |
| hsa-miR-4674 | hsa_circ_0018909 |
| hsa-miR-4674 | hsa_circ_0003168 |
| hsa-miR-4674 | hsa_circ_0000497 |
| hsa-miR-4674 | hsa_circ_0048025 |
| hsa-miR-4674 | hsa_circ_0008590 |
| hsa-miR-4674 | hsa_circ_0061052 |
| hsa-miR-4674 | hsa_circ_0072857 |
| hsa-miR-4674 | hsa_circ_0001573 |
| hsa-miR-4674 | hsa_circ_0003700 |
| hsa-miR-4674 | hsa_circ_0004381 |
| hsa-miR-4674 | hsa_circ_0009092 |
| hsa-miR-4674 | hsa_circ_0084789 |
| hsa-miR-4675 | hsa_circ_0045890 |
| hsa-miR-4675 | hsa_circ_0003340 |
| hsa-miR-4682 | hsa_circ_0000395 |
| hsa-miR-4682 | hsa_circ_0031933 |
| hsa-miR-4682 | hsa_circ_0007132 |
| hsa-miR-4682 | hsa_circ_0007145 |
| hsa-miR-4682 | hsa_circ_0087288 |
| hsa-miR-4683 | hsa_circ_0046430 |
| hsa-miR-4683 | hsa_circ_0092297 |
| hsa-miR-4685-5p | hsa_circ_0002402 |
| hsa-miR-4685-5p | hsa_circ_0009142 |
| hsa-miR-4685-5p | hsa_circ_0007015 |
| hsa-miR-4685-5p | hsa_circ_0017289 |
| hsa-miR-4685-5p | hsa_circ_0003168 |
| hsa-miR-4685-5p | hsa_circ_0003379 |
| hsa-miR-4685-5p | hsa_circ_0022383 |
| hsa-miR-4685-5p | hsa_circ_0000375 |
| hsa-miR-4685-5p | hsa_circ_0000417 |
| hsa-miR-4685-5p | hsa_circ_0000462 |
| hsa-miR-4685-5p | hsa_circ_0029405 |
| hsa-miR-4685-5p | hsa_circ_0031933 |
| hsa-miR-4685-5p | hsa_circ_0000565 |
| hsa-miR-4685-5p | hsa_circ_0000650 |
| hsa-miR-4685-5p | hsa_circ_0036768 |
| hsa-miR-4685-5p | hsa_circ_0007146 |
| hsa-miR-4685-5p | hsa_circ_0007846 |
| hsa-miR-4685-5p | hsa_circ_0046430 |
| hsa-miR-4685-5p | hsa_circ_0048025 |
| hsa-miR-4685-5p | hsa_circ_0000918 |
| hsa-miR-4685-5p | hsa_circ_0008030 |
| hsa-miR-4685-5p | hsa_circ_0000936 |
| hsa-miR-4685-5p | hsa_circ_0092297 |
| hsa-miR-4685-5p | hsa_circ_0004218 |
| hsa-miR-4685-5p | hsa_circ_0092299 |
| hsa-miR-4685-5p | hsa_circ_0004705 |
| hsa-miR-4685-5p | hsa_circ_0092277 |
| hsa-miR-4685-5p | hsa_circ_0001965 |
| hsa-miR-4685-5p | hsa_circ_0001394 |
| hsa-miR-4685-5p | hsa_circ_0006693 |
| hsa-miR-4685-5p | hsa_circ_0001417 |
| hsa-miR-4685-5p | hsa_circ_0001472 |
| hsa-miR-4685-5p | hsa_circ_0007132 |
| hsa-miR-4685-5p | hsa_circ_0003340 |
| hsa-miR-4685-5p | hsa_circ_0081343 |
| hsa-miR-4685-5p | hsa_circ_0001748 |
| hsa-miR-4685-5p | hsa_circ_0005630 |
| hsa-miR-4685-5p | hsa_circ_0001824 |
| hsa-miR-4685-5p | hsa_circ_0008934 |
| hsa-miR-4685-5p | hsa_circ_0087288 |
| hsa-miR-4685-5p | hsa_circ_0087305 |
| hsa-miR-4685-3p | hsa_circ_0004519 |
| hsa-miR-4685-3p | hsa_circ_0040921 |
| hsa-miR-4685-3p | hsa_circ_0005571 |
| hsa-miR-4686 | hsa_circ_0049657 |
| hsa-miR-4686 | hsa_circ_0078522 |
| hsa-miR-4687-5p | hsa_circ_0020594 |
| hsa-miR-4687-5p | hsa_circ_0006597 |
| hsa-miR-4687-5p | hsa_circ_0002805 |
| hsa-miR-4687-5p | hsa_circ_0004692 |
| hsa-miR-4687-5p | hsa_circ_0001756 |
| hsa-miR-4687-3p | hsa_circ_0017289 |
| hsa-miR-4687-3p | hsa_circ_0020005 |
| hsa-miR-4687-3p | hsa_circ_0020594 |
| hsa-miR-4687-3p | hsa_circ_0036763 |
| hsa-miR-4687-3p | hsa_circ_0000650 |
| hsa-miR-4687-3p | hsa_circ_0036768 |
| hsa-miR-4687-3p | hsa_circ_0007846 |
| hsa-miR-4687-3p | hsa_circ_0045905 |
| hsa-miR-4687-3p | hsa_circ_0046430 |
| hsa-miR-4687-3p | hsa_circ_0048025 |
| hsa-miR-4687-3p | hsa_circ_0061052 |
| hsa-miR-4687-3p | hsa_circ_0002805 |
| hsa-miR-4687-3p | hsa_circ_0061179 |
| hsa-miR-4687-3p | hsa_circ_0092299 |
| hsa-miR-4687-3p | hsa_circ_0069748 |
| hsa-miR-4687-3p | hsa_circ_0009092 |
| hsa-miR-4687-3p | hsa_circ_0007145 |
| hsa-miR-4687-3p | hsa_circ_0002094 |
| hsa-miR-1343-5p | hsa_circ_0002402 |
| hsa-miR-1343-5p | hsa_circ_0003557 |
| hsa-miR-1343-5p | hsa_circ_0000417 |
| hsa-miR-1343-5p | hsa_circ_0004137 |
| hsa-miR-1343-5p | hsa_circ_0031933 |
| hsa-miR-1343-5p | hsa_circ_0000660 |
| hsa-miR-1343-5p | hsa_circ_0007146 |
| hsa-miR-1343-5p | hsa_circ_0007846 |
| hsa-miR-1343-5p | hsa_circ_0045890 |
| hsa-miR-1343-5p | hsa_circ_0046430 |
| hsa-miR-1343-5p | hsa_circ_0004003 |
| hsa-miR-1343-5p | hsa_circ_0004891 |
| hsa-miR-1343-5p | hsa_circ_0049657 |
| hsa-miR-1343-5p | hsa_circ_0049998 |
| hsa-miR-1343-5p | hsa_circ_0005571 |
| hsa-miR-1343-5p | hsa_circ_0092297 |
| hsa-miR-1343-5p | hsa_circ_0004218 |
| hsa-miR-1343-5p | hsa_circ_0060904 |
| hsa-miR-1343-5p | hsa_circ_0002805 |
| hsa-miR-1343-5p | hsa_circ_0061179 |
| hsa-miR-1343-5p | hsa_circ_0092299 |
| hsa-miR-1343-5p | hsa_circ_0064136 |
| hsa-miR-1343-5p | hsa_circ_0092277 |
| hsa-miR-1343-5p | hsa_circ_0007132 |
| hsa-miR-1343-5p | hsa_circ_0004381 |
| hsa-miR-1343-5p | hsa_circ_0003340 |
| hsa-miR-1343-5p | hsa_circ_0001708 |
| hsa-miR-1343-5p | hsa_circ_0002755 |
| hsa-miR-1343-5p | hsa_circ_0087288 |
| hsa-miR-1343-5p | hsa_circ_0087305 |
| hsa-miR-1343-5p | hsa_circ_0001866 |
| hsa-miR-1343-5p | hsa_circ_0092125 |
| hsa-miR-1343-3p | hsa_circ_0031933 |
| hsa-miR-1343-3p | hsa_circ_0033144 |
| hsa-miR-1343-3p | hsa_circ_0048025 |
| hsa-miR-1343-3p | hsa_circ_0054086 |
| hsa-miR-1343-3p | hsa_circ_0092297 |
| hsa-miR-1343-3p | hsa_circ_0002805 |
| hsa-miR-1343-3p | hsa_circ_0002113 |
| hsa-miR-1343-3p | hsa_circ_0070396 |
| hsa-miR-1343-3p | hsa_circ_0001573 |
| hsa-miR-1343-3p | hsa_circ_0007132 |
| hsa-miR-1343-3p | hsa_circ_0008113 |
| hsa-miR-4688 | hsa_circ_0048025 |
| hsa-miR-4688 | hsa_circ_0092297 |
| hsa-miR-4688 | hsa_circ_0002805 |
| hsa-miR-4688 | hsa_circ_0061179 |
| hsa-miR-4688 | hsa_circ_0003602 |
| hsa-miR-4688 | hsa_circ_0092277 |
| hsa-miR-4689 | hsa_circ_0009349 |
| hsa-miR-4689 | hsa_circ_0009357 |
| hsa-miR-4689 | hsa_circ_0009360 |
| hsa-miR-4689 | hsa_circ_0011422 |
| hsa-miR-4689 | hsa_circ_0049998 |
| hsa-miR-4689 | hsa_circ_0092299 |
| hsa-miR-4689 | hsa_circ_0005630 |
| hsa-miR-4690-5p | hsa_circ_0003557 |
| hsa-miR-4690-5p | hsa_circ_0029633 |
| hsa-miR-4690-5p | hsa_circ_0007146 |
| hsa-miR-4690-5p | hsa_circ_0008030 |
| hsa-miR-4690-5p | hsa_circ_0004470 |
| hsa-miR-4690-5p | hsa_circ_0002755 |
| hsa-miR-4690-5p | hsa_circ_0087305 |
| hsa-miR-4690-3p | hsa_circ_0025388 |
| hsa-miR-4690-3p | hsa_circ_0002805 |
| hsa-miR-4690-3p | hsa_circ_0061179 |
| hsa-miR-4690-3p | hsa_circ_0007132 |
| hsa-miR-4690-3p | hsa_circ_0005273 |
| hsa-miR-4691-5p | hsa_circ_0017438 |
| hsa-miR-4691-5p | hsa_circ_0003557 |
| hsa-miR-4691-5p | hsa_circ_0008521 |
| hsa-miR-4691-5p | hsa_circ_0003848 |
| hsa-miR-4691-5p | hsa_circ_0002564 |
| hsa-miR-4691-5p | hsa_circ_0002289 |
| hsa-miR-4691-5p | hsa_circ_0007846 |
| hsa-miR-4691-5p | hsa_circ_0045890 |
| hsa-miR-4691-5p | hsa_circ_0045905 |
| hsa-miR-4691-5p | hsa_circ_0000835 |
| hsa-miR-4691-5p | hsa_circ_0000836 |
| hsa-miR-4691-5p | hsa_circ_0048025 |
| hsa-miR-4691-5p | hsa_circ_0002805 |
| hsa-miR-4691-5p | hsa_circ_0092299 |
| hsa-miR-4691-5p | hsa_circ_0064136 |
| hsa-miR-4691-5p | hsa_circ_0006884 |
| hsa-miR-4691-5p | hsa_circ_0001394 |
| hsa-miR-4691-5p | hsa_circ_0006681 |
| hsa-miR-4691-5p | hsa_circ_0007145 |
| hsa-miR-4691-5p | hsa_circ_0002755 |
| hsa-miR-4691-5p | hsa_circ_0002094 |
| hsa-miR-4691-5p | hsa_circ_0007685 |
| hsa-miR-4691-5p | hsa_circ_0001910 |
| hsa-miR-4691-3p | hsa_circ_0001394 |
| hsa-miR-4692 | hsa_circ_0029976 |
| hsa-miR-4695-5p | hsa_circ_0015004 |
| hsa-miR-4695-5p | hsa_circ_0003379 |
| hsa-miR-4695-5p | hsa_circ_0022015 |
| hsa-miR-4695-5p | hsa_circ_0003557 |
| hsa-miR-4695-5p | hsa_circ_0033144 |
| hsa-miR-4695-5p | hsa_circ_0003713 |
| hsa-miR-4695-5p | hsa_circ_0007846 |
| hsa-miR-4695-5p | hsa_circ_0048025 |
| hsa-miR-4695-5p | hsa_circ_0000918 |
| hsa-miR-4695-5p | hsa_circ_0003854 |
| hsa-miR-4695-5p | hsa_circ_0004470 |
| hsa-miR-4695-5p | hsa_circ_0001394 |
| hsa-miR-4695-5p | hsa_circ_0070467 |
| hsa-miR-4695-5p | hsa_circ_0007132 |
| hsa-miR-4695-5p | hsa_circ_0001756 |
| hsa-miR-4695-5p | hsa_circ_0002094 |
| hsa-miR-4695-3p | hsa_circ_0029405 |
| hsa-miR-4695-3p | hsa_circ_0006732 |
| hsa-miR-4695-3p | hsa_circ_0000973 |
| hsa-miR-4695-3p | hsa_circ_0002094 |
| hsa-miR-4697-3p | hsa_circ_0092299 |
| hsa-miR-4697-3p | hsa_circ_0004692 |
| hsa-miR-4697-3p | hsa_circ_0065147 |
| hsa-miR-4697-3p | hsa_circ_0065149 |
| hsa-miR-4697-3p | hsa_circ_0002569 |
| hsa-miR-4697-3p | hsa_circ_0006693 |
| hsa-miR-4697-3p | hsa_circ_0001417 |
| hsa-miR-4700-5p | hsa_circ_0002805 |
| hsa-miR-4700-5p | hsa_circ_0061179 |
| hsa-miR-4700-3p | hsa_circ_0002805 |
| hsa-miR-4700-3p | hsa_circ_0092299 |
| hsa-miR-4700-3p | hsa_circ_0001329 |
| hsa-miR-4700-3p | hsa_circ_0006884 |
| hsa-miR-4700-3p | hsa_circ_0007132 |
| hsa-miR-4700-3p | hsa_circ_0084789 |
| hsa-miR-4701-5p | hsa_circ_0045890 |
| hsa-miR-4701-3p | hsa_circ_0009732 |
| hsa-miR-4706 | hsa_circ_0009349 |
| hsa-miR-4706 | hsa_circ_0009357 |
| hsa-miR-4706 | hsa_circ_0009360 |
| hsa-miR-4706 | hsa_circ_0002402 |
| hsa-miR-4706 | hsa_circ_0000130 |
| hsa-miR-4706 | hsa_circ_0000131 |
| hsa-miR-4706 | hsa_circ_0006649 |
| hsa-miR-4706 | hsa_circ_0003557 |
| hsa-miR-4706 | hsa_circ_0000375 |
| hsa-miR-4706 | hsa_circ_0000417 |
| hsa-miR-4706 | hsa_circ_0034189 |
| hsa-miR-4706 | hsa_circ_0003838 |
| hsa-miR-4706 | hsa_circ_0007846 |
| hsa-miR-4706 | hsa_circ_0040921 |
| hsa-miR-4706 | hsa_circ_0045890 |
| hsa-miR-4706 | hsa_circ_0046292 |
| hsa-miR-4706 | hsa_circ_0046430 |
| hsa-miR-4706 | hsa_circ_0048025 |
| hsa-miR-4706 | hsa_circ_0002926 |
| hsa-miR-4706 | hsa_circ_0049657 |
| hsa-miR-4706 | hsa_circ_0050119 |
| hsa-miR-4706 | hsa_circ_0000936 |
| hsa-miR-4706 | hsa_circ_0003854 |
| hsa-miR-4706 | hsa_circ_0007609 |
| hsa-miR-4706 | hsa_circ_0002113 |
| hsa-miR-4706 | hsa_circ_0004470 |
| hsa-miR-4706 | hsa_circ_0063331 |
| hsa-miR-4706 | hsa_circ_0001394 |
| hsa-miR-4706 | hsa_circ_0072437 |
| hsa-miR-4706 | hsa_circ_0004381 |
| hsa-miR-4706 | hsa_circ_0002755 |
| hsa-miR-4706 | hsa_circ_0001756 |
| hsa-miR-4706 | hsa_circ_0002094 |
| hsa-miR-4706 | hsa_circ_0005630 |
| hsa-miR-4706 | hsa_circ_0087288 |
| hsa-miR-4706 | hsa_circ_0087305 |
| hsa-miR-4706 | hsa_circ_0087855 |
| hsa-miR-4706 | hsa_circ_0008812 |
| hsa-miR-4706 | hsa_circ_0006174 |
| hsa-miR-4706 | hsa_circ_0087861 |
| hsa-miR-4706 | hsa_circ_0087862 |
| hsa-miR-4707-5p | hsa_circ_0002402 |
| hsa-miR-4707-5p | hsa_circ_0000131 |
| hsa-miR-4707-5p | hsa_circ_0016979 |
| hsa-miR-4707-5p | hsa_circ_0006608 |
| hsa-miR-4707-5p | hsa_circ_0003168 |
| hsa-miR-4707-5p | hsa_circ_0003379 |
| hsa-miR-4707-5p | hsa_circ_0022723 |
| hsa-miR-4707-5p | hsa_circ_0000417 |
| hsa-miR-4707-5p | hsa_circ_0031933 |
| hsa-miR-4707-5p | hsa_circ_0034189 |
| hsa-miR-4707-5p | hsa_circ_0036763 |
| hsa-miR-4707-5p | hsa_circ_0000650 |
| hsa-miR-4707-5p | hsa_circ_0007146 |
| hsa-miR-4707-5p | hsa_circ_0007846 |
| hsa-miR-4707-5p | hsa_circ_0040039 |
| hsa-miR-4707-5p | hsa_circ_0004354 |
| hsa-miR-4707-5p | hsa_circ_0040823 |
| hsa-miR-4707-5p | hsa_circ_0092337 |
| hsa-miR-4707-5p | hsa_circ_0045890 |
| hsa-miR-4707-5p | hsa_circ_0046430 |
| hsa-miR-4707-5p | hsa_circ_0048025 |
| hsa-miR-4707-5p | hsa_circ_0004003 |
| hsa-miR-4707-5p | hsa_circ_0004891 |
| hsa-miR-4707-5p | hsa_circ_0002926 |
| hsa-miR-4707-5p | hsa_circ_0005571 |
| hsa-miR-4707-5p | hsa_circ_0050119 |
| hsa-miR-4707-5p | hsa_circ_0092297 |
| hsa-miR-4707-5p | hsa_circ_0058514 |
| hsa-miR-4707-5p | hsa_circ_0058520 |
| hsa-miR-4707-5p | hsa_circ_0058522 |
| hsa-miR-4707-5p | hsa_circ_0003854 |
| hsa-miR-4707-5p | hsa_circ_0004218 |
| hsa-miR-4707-5p | hsa_circ_0002805 |
| hsa-miR-4707-5p | hsa_circ_0061179 |
| hsa-miR-4707-5p | hsa_circ_0002954 |
| hsa-miR-4707-5p | hsa_circ_0092299 |
| hsa-miR-4707-5p | hsa_circ_0004692 |
| hsa-miR-4707-5p | hsa_circ_0065147 |
| hsa-miR-4707-5p | hsa_circ_0065149 |
| hsa-miR-4707-5p | hsa_circ_0002569 |
| hsa-miR-4707-5p | hsa_circ_0001400 |
| hsa-miR-4707-5p | hsa_circ_0071311 |
| hsa-miR-4707-5p | hsa_circ_0001573 |
| hsa-miR-4707-5p | hsa_circ_0007132 |
| hsa-miR-4707-5p | hsa_circ_0001658 |
| hsa-miR-4707-5p | hsa_circ_0001707 |
| hsa-miR-4707-5p | hsa_circ_0081343 |
| hsa-miR-4707-5p | hsa_circ_0082131 |
| hsa-miR-4707-5p | hsa_circ_0002094 |
| hsa-miR-4707-5p | hsa_circ_0005630 |
| hsa-miR-4707-5p | hsa_circ_0084789 |
| hsa-miR-4707-5p | hsa_circ_0087305 |
| hsa-miR-4707-5p | hsa_circ_0001947 |
| hsa-miR-4707-3p | hsa_circ_0009142 |
| hsa-miR-4707-3p | hsa_circ_0003379 |
| hsa-miR-4707-3p | hsa_circ_0033144 |
| hsa-miR-4707-3p | hsa_circ_0003854 |
| hsa-miR-4707-3p | hsa_circ_0092299 |
| hsa-miR-4707-3p | hsa_circ_0004470 |
| hsa-miR-4707-3p | hsa_circ_0007132 |
| hsa-miR-4708-3p | hsa_circ_0009349 |
| hsa-miR-4709-3p | hsa_circ_0050119 |
| hsa-miR-4710 | hsa_circ_0046430 |
| hsa-miR-4710 | hsa_circ_0070467 |
| hsa-miR-4713-5p | hsa_circ_0020594 |
| hsa-miR-4713-5p | hsa_circ_0007132 |
| hsa-miR-4713-3p | hsa_circ_0000206 |
| hsa-miR-4713-3p | hsa_circ_0017461 |
| hsa-miR-4713-3p | hsa_circ_0006735 |
| hsa-miR-4715-3p | hsa_circ_0007146 |
| hsa-miR-4716-5p | hsa_circ_0006732 |
| hsa-miR-4717-3p | hsa_circ_0005733 |
| hsa-miR-4717-3p | hsa_circ_0040039 |
| hsa-miR-4717-3p | hsa_circ_0046430 |
| hsa-miR-4717-3p | hsa_circ_0070467 |
| hsa-miR-4721 | hsa_circ_0022392 |
| hsa-miR-4721 | hsa_circ_0007372 |
| hsa-miR-4721 | hsa_circ_0045890 |
| hsa-miR-4721 | hsa_circ_0045905 |
| hsa-miR-4721 | hsa_circ_0005571 |
| hsa-miR-4721 | hsa_circ_0001394 |
| hsa-miR-4721 | hsa_circ_0071869 |
| hsa-miR-4722-5p | hsa_circ_0017289 |
| hsa-miR-4722-5p | hsa_circ_0036763 |
| hsa-miR-4722-5p | hsa_circ_0000650 |
| hsa-miR-4722-5p | hsa_circ_0036768 |
| hsa-miR-4722-5p | hsa_circ_0007846 |
| hsa-miR-4722-5p | hsa_circ_0045890 |
| hsa-miR-4722-5p | hsa_circ_0045905 |
| hsa-miR-4722-5p | hsa_circ_0048025 |
| hsa-miR-4722-5p | hsa_circ_0050119 |
| hsa-miR-4722-5p | hsa_circ_0008261 |
| hsa-miR-4722-5p | hsa_circ_0001187 |
| hsa-miR-4722-5p | hsa_circ_0008078 |
| hsa-miR-4722-5p | hsa_circ_0061936 |
| hsa-miR-4722-5p | hsa_circ_0092299 |
| hsa-miR-4722-5p | hsa_circ_0004470 |
| hsa-miR-4722-5p | hsa_circ_0002077 |
| hsa-miR-4722-5p | hsa_circ_0001394 |
| hsa-miR-4722-5p | hsa_circ_0071311 |
| hsa-miR-4722-5p | hsa_circ_0001549 |
| hsa-miR-4722-5p | hsa_circ_0004381 |
| hsa-miR-4722-5p | hsa_circ_0002094 |
| hsa-miR-4722-5p | hsa_circ_0005630 |
| hsa-miR-4722-5p | hsa_circ_0087288 |
| hsa-miR-4722-3p | hsa_circ_0016979 |
| hsa-miR-4722-3p | hsa_circ_0031933 |
| hsa-miR-4722-3p | hsa_circ_0002113 |
| hsa-miR-4722-3p | hsa_circ_0007132 |
| hsa-miR-4723-5p | hsa_circ_0002755 |
| hsa-miR-4723-3p | hsa_circ_0040921 |
| hsa-miR-4723-3p | hsa_circ_0000996 |
| hsa-miR-4723-3p | hsa_circ_0004692 |
| hsa-miR-4723-3p | hsa_circ_0065147 |
| hsa-miR-4723-3p | hsa_circ_0065149 |
| hsa-miR-4723-3p | hsa_circ_0002569 |
| hsa-miR-4723-3p | hsa_circ_0006693 |
| hsa-miR-4723-3p | hsa_circ_0001417 |
| hsa-miR-4723-3p | hsa_circ_0007132 |
| hsa-miR-4723-3p | hsa_circ_0084789 |
| hsa-miR-4725-5p | hsa_circ_0087305 |
| hsa-miR-4725-3p | hsa_circ_0016979 |
| hsa-miR-4725-3p | hsa_circ_0049657 |
| hsa-miR-4725-3p | hsa_circ_0006006 |
| hsa-miR-4725-3p | hsa_circ_0002805 |
| hsa-miR-4725-3p | hsa_circ_0061179 |
| hsa-miR-4725-3p | hsa_circ_0092299 |
| hsa-miR-4725-3p | hsa_circ_0004381 |
| hsa-miR-4725-3p | hsa_circ_0005630 |
| hsa-miR-4725-3p | hsa_circ_0006566 |
| hsa-miR-4726-5p | hsa_circ_0009581 |
| hsa-miR-4726-5p | hsa_circ_0006837 |
| hsa-miR-4726-5p | hsa_circ_0005576 |
| hsa-miR-4726-5p | hsa_circ_0009142 |
| hsa-miR-4726-5p | hsa_circ_0007015 |
| hsa-miR-4726-5p | hsa_circ_0017289 |
| hsa-miR-4726-5p | hsa_circ_0005090 |
| hsa-miR-4726-5p | hsa_circ_0006254 |
| hsa-miR-4726-5p | hsa_circ_0003768 |
| hsa-miR-4726-5p | hsa_circ_0003557 |
| hsa-miR-4726-5p | hsa_circ_0000417 |
| hsa-miR-4726-5p | hsa_circ_0031933 |
| hsa-miR-4726-5p | hsa_circ_0001955 |
| hsa-miR-4726-5p | hsa_circ_0002696 |
| hsa-miR-4726-5p | hsa_circ_0007079 |
| hsa-miR-4726-5p | hsa_circ_0046430 |
| hsa-miR-4726-5p | hsa_circ_0000918 |
| hsa-miR-4726-5p | hsa_circ_0052131 |
| hsa-miR-4726-5p | hsa_circ_0058514 |
| hsa-miR-4726-5p | hsa_circ_0058520 |
| hsa-miR-4726-5p | hsa_circ_0058522 |
| hsa-miR-4726-5p | hsa_circ_0061052 |
| hsa-miR-4726-5p | hsa_circ_0004872 |
| hsa-miR-4726-5p | hsa_circ_0092299 |
| hsa-miR-4726-5p | hsa_circ_0002077 |
| hsa-miR-4726-5p | hsa_circ_0006693 |
| hsa-miR-4726-5p | hsa_circ_0072437 |
| hsa-miR-4726-5p | hsa_circ_0073379 |
| hsa-miR-4726-5p | hsa_circ_0005540 |
| hsa-miR-4726-5p | hsa_circ_0007132 |
| hsa-miR-4726-5p | hsa_circ_0004381 |
| hsa-miR-4726-5p | hsa_circ_0003340 |
| hsa-miR-4726-5p | hsa_circ_0005630 |
| hsa-miR-4726-5p | hsa_circ_0087288 |
| hsa-miR-4726-3p | hsa_circ_0002402 |
| hsa-miR-4726-3p | hsa_circ_0011571 |
| hsa-miR-4726-3p | hsa_circ_0011572 |
| hsa-miR-4726-3p | hsa_circ_0017446 |
| hsa-miR-4726-3p | hsa_circ_0000206 |
| hsa-miR-4726-3p | hsa_circ_0092297 |
| hsa-miR-4726-3p | hsa_circ_0007967 |
| hsa-miR-4726-3p | hsa_circ_0007132 |
| hsa-miR-4727-5p | hsa_circ_0006552 |
| hsa-miR-4727-5p | hsa_circ_0005630 |
| hsa-miR-4728-5p | hsa_circ_0017248 |
| hsa-miR-4728-5p | hsa_circ_0023918 |
| hsa-miR-4728-5p | hsa_circ_0023920 |
| hsa-miR-4728-5p | hsa_circ_0023923 |
| hsa-miR-4728-5p | hsa_circ_0029405 |
| hsa-miR-4728-5p | hsa_circ_0004692 |
| hsa-miR-4728-5p | hsa_circ_0065147 |
| hsa-miR-4728-5p | hsa_circ_0065149 |
| hsa-miR-4728-5p | hsa_circ_0002569 |
| hsa-miR-4728-5p | hsa_circ_0070467 |
| hsa-miR-4728-5p | hsa_circ_0001472 |
| hsa-miR-4728-5p | hsa_circ_0073379 |
| hsa-miR-4728-5p | hsa_circ_0005540 |
| hsa-miR-4728-5p | hsa_circ_0005630 |
| hsa-miR-4728-3p | hsa_circ_0017446 |
| hsa-miR-4728-3p | hsa_circ_0000206 |
| hsa-miR-4728-3p | hsa_circ_0020594 |
| hsa-miR-4728-3p | hsa_circ_0004519 |
| hsa-miR-4728-3p | hsa_circ_0040823 |
| hsa-miR-4728-3p | hsa_circ_0045890 |
| hsa-miR-4728-3p | hsa_circ_0047347 |
| hsa-miR-4728-3p | hsa_circ_0005571 |
| hsa-miR-4728-3p | hsa_circ_0004692 |
| hsa-miR-4728-3p | hsa_circ_0007132 |
| hsa-miR-4728-3p | hsa_circ_0006022 |
| hsa-miR-4730 | hsa_circ_0003557 |
| hsa-miR-4730 | hsa_circ_0040921 |
| hsa-miR-4730 | hsa_circ_0092297 |
| hsa-miR-4730 | hsa_circ_0092283 |
| hsa-miR-4731-5p | hsa_circ_0007146 |
| hsa-miR-4731-5p | hsa_circ_0046430 |
| hsa-miR-4731-5p | hsa_circ_0004712 |
| hsa-miR-4732-3p | hsa_circ_0033144 |
| hsa-miR-4733-3p | hsa_circ_0000417 |
| hsa-miR-4733-3p | hsa_circ_0092297 |
| hsa-miR-4734 | hsa_circ_0005782 |
| hsa-miR-4734 | hsa_circ_0002402 |
| hsa-miR-4734 | hsa_circ_0003379 |
| hsa-miR-4734 | hsa_circ_0007001 |
| hsa-miR-4734 | hsa_circ_0000417 |
| hsa-miR-4734 | hsa_circ_0029976 |
| hsa-miR-4734 | hsa_circ_0007146 |
| hsa-miR-4734 | hsa_circ_0007846 |
| hsa-miR-4734 | hsa_circ_0040823 |
| hsa-miR-4734 | hsa_circ_0040921 |
| hsa-miR-4734 | hsa_circ_0045890 |
| hsa-miR-4734 | hsa_circ_0046430 |
| hsa-miR-4734 | hsa_circ_0048025 |
| hsa-miR-4734 | hsa_circ_0061052 |
| hsa-miR-4734 | hsa_circ_0002805 |
| hsa-miR-4734 | hsa_circ_0061179 |
| hsa-miR-4734 | hsa_circ_0007609 |
| hsa-miR-4734 | hsa_circ_0092299 |
| hsa-miR-4734 | hsa_circ_0004705 |
| hsa-miR-4734 | hsa_circ_0001394 |
| hsa-miR-4734 | hsa_circ_0004712 |
| hsa-miR-4734 | hsa_circ_0002755 |
| hsa-miR-4734 | hsa_circ_0002094 |
| hsa-miR-4734 | hsa_circ_0087305 |
| hsa-miR-4738-3p | hsa_circ_0008102 |
| hsa-miR-4739 | hsa_circ_0009349 |
| hsa-miR-4739 | hsa_circ_0009357 |
| hsa-miR-4739 | hsa_circ_0009360 |
| hsa-miR-4739 | hsa_circ_0002402 |
| hsa-miR-4739 | hsa_circ_0002570 |
| hsa-miR-4739 | hsa_circ_0017248 |
| hsa-miR-4739 | hsa_circ_0000220 |
| hsa-miR-4739 | hsa_circ_0006665 |
| hsa-miR-4739 | hsa_circ_0006649 |
| hsa-miR-4739 | hsa_circ_0003168 |
| hsa-miR-4739 | hsa_circ_0020005 |
| hsa-miR-4739 | hsa_circ_0022383 |
| hsa-miR-4739 | hsa_circ_0003557 |
| hsa-miR-4739 | hsa_circ_0007372 |
| hsa-miR-4739 | hsa_circ_0000375 |
| hsa-miR-4739 | hsa_circ_0000417 |
| hsa-miR-4739 | hsa_circ_0004137 |
| hsa-miR-4739 | hsa_circ_0033144 |
| hsa-miR-4739 | hsa_circ_0003838 |
| hsa-miR-4739 | hsa_circ_0007846 |
| hsa-miR-4739 | hsa_circ_0040039 |
| hsa-miR-4739 | hsa_circ_0004354 |
| hsa-miR-4739 | hsa_circ_0045890 |
| hsa-miR-4739 | hsa_circ_0046430 |
| hsa-miR-4739 | hsa_circ_0049657 |
| hsa-miR-4739 | hsa_circ_0049998 |
| hsa-miR-4739 | hsa_circ_0050119 |
| hsa-miR-4739 | hsa_circ_0000936 |
| hsa-miR-4739 | hsa_circ_0000973 |
| hsa-miR-4739 | hsa_circ_0000996 |
| hsa-miR-4739 | hsa_circ_0002805 |
| hsa-miR-4739 | hsa_circ_0061179 |
| hsa-miR-4739 | hsa_circ_0007609 |
| hsa-miR-4739 | hsa_circ_0061936 |
| hsa-miR-4739 | hsa_circ_0092299 |
| hsa-miR-4739 | hsa_circ_0004470 |
| hsa-miR-4739 | hsa_circ_0002077 |
| hsa-miR-4739 | hsa_circ_0004692 |
| hsa-miR-4739 | hsa_circ_0065147 |
| hsa-miR-4739 | hsa_circ_0065149 |
| hsa-miR-4739 | hsa_circ_0002569 |
| hsa-miR-4739 | hsa_circ_0001394 |
| hsa-miR-4739 | hsa_circ_0001400 |
| hsa-miR-4739 | hsa_circ_0069748 |
| hsa-miR-4739 | hsa_circ_0070467 |
| hsa-miR-4739 | hsa_circ_0073379 |
| hsa-miR-4739 | hsa_circ_0005540 |
| hsa-miR-4739 | hsa_circ_0001549 |
| hsa-miR-4739 | hsa_circ_0001573 |
| hsa-miR-4739 | hsa_circ_0007132 |
| hsa-miR-4739 | hsa_circ_0076742 |
| hsa-miR-4739 | hsa_circ_0001658 |
| hsa-miR-4739 | hsa_circ_0004381 |
| hsa-miR-4739 | hsa_circ_0002094 |
| hsa-miR-4739 | hsa_circ_0005630 |
| hsa-miR-4739 | hsa_circ_0084789 |
| hsa-miR-4739 | hsa_circ_0001829 |
| hsa-miR-4740-5p | hsa_circ_0092283 |
| hsa-miR-4740-3p | hsa_circ_0060904 |
| hsa-miR-4740-3p | hsa_circ_0002805 |
| hsa-miR-4740-3p | hsa_circ_0092299 |
| hsa-miR-4740-3p | hsa_circ_0007132 |
| hsa-miR-4741 | hsa_circ_0000048 |
| hsa-miR-4741 | hsa_circ_0007009 |
| hsa-miR-4741 | hsa_circ_0007015 |
| hsa-miR-4741 | hsa_circ_0017289 |
| hsa-miR-4741 | hsa_circ_0005090 |
| hsa-miR-4741 | hsa_circ_0018909 |
| hsa-miR-4741 | hsa_circ_0022505 |
| hsa-miR-4741 | hsa_circ_0022723 |
| hsa-miR-4741 | hsa_circ_0003557 |
| hsa-miR-4741 | hsa_circ_0030051 |
| hsa-miR-4741 | hsa_circ_0031933 |
| hsa-miR-4741 | hsa_circ_0000650 |
| hsa-miR-4741 | hsa_circ_0036768 |
| hsa-miR-4741 | hsa_circ_0007146 |
| hsa-miR-4741 | hsa_circ_0007846 |
| hsa-miR-4741 | hsa_circ_0040823 |
| hsa-miR-4741 | hsa_circ_0040921 |
| hsa-miR-4741 | hsa_circ_0045890 |
| hsa-miR-4741 | hsa_circ_0046430 |
| hsa-miR-4741 | hsa_circ_0048025 |
| hsa-miR-4741 | hsa_circ_0004003 |
| hsa-miR-4741 | hsa_circ_0002926 |
| hsa-miR-4741 | hsa_circ_0006877 |
| hsa-miR-4741 | hsa_circ_0049657 |
| hsa-miR-4741 | hsa_circ_0092297 |
| hsa-miR-4741 | hsa_circ_0061052 |
| hsa-miR-4741 | hsa_circ_0002805 |
| hsa-miR-4741 | hsa_circ_0061179 |
| hsa-miR-4741 | hsa_circ_0007609 |
| hsa-miR-4741 | hsa_circ_0061936 |
| hsa-miR-4741 | hsa_circ_0069399 |
| hsa-miR-4741 | hsa_circ_0072857 |
| hsa-miR-4741 | hsa_circ_0007132 |
| hsa-miR-4741 | hsa_circ_0003738 |
| hsa-miR-4741 | hsa_circ_0003340 |
| hsa-miR-4741 | hsa_circ_0002755 |
| hsa-miR-4741 | hsa_circ_0081343 |
| hsa-miR-4741 | hsa_circ_0002094 |
| hsa-miR-4741 | hsa_circ_0084429 |
| hsa-miR-4741 | hsa_circ_0001910 |
| hsa-miR-4743-5p | hsa_circ_0003168 |
| hsa-miR-4743-5p | hsa_circ_0000417 |
| hsa-miR-4743-5p | hsa_circ_0002805 |
| hsa-miR-4743-5p | hsa_circ_0061179 |
| hsa-miR-4743-5p | hsa_circ_0004470 |
| hsa-miR-4743-5p | hsa_circ_0003602 |
| hsa-miR-4743-5p | hsa_circ_0071311 |
| hsa-miR-4743-5p | hsa_circ_0007132 |
| hsa-miR-4743-5p | hsa_circ_0084429 |
| hsa-miR-4743-5p | hsa_circ_0087305 |
| hsa-miR-4743-5p | hsa_circ_0087855 |
| hsa-miR-4743-5p | hsa_circ_0008812 |
| hsa-miR-4743-5p | hsa_circ_0006174 |
| hsa-miR-4743-5p | hsa_circ_0087861 |
| hsa-miR-4743-5p | hsa_circ_0087862 |
| hsa-miR-4743-3p | hsa_circ_0064136 |
| hsa-miR-4745-5p | hsa_circ_0017248 |
| hsa-miR-4745-5p | hsa_circ_0003379 |
| hsa-miR-4745-5p | hsa_circ_0022392 |
| hsa-miR-4745-5p | hsa_circ_0003557 |
| hsa-miR-4745-5p | hsa_circ_0000375 |
| hsa-miR-4745-5p | hsa_circ_0000417 |
| hsa-miR-4745-5p | hsa_circ_0000497 |
| hsa-miR-4745-5p | hsa_circ_0031933 |
| hsa-miR-4745-5p | hsa_circ_0006735 |
| hsa-miR-4745-5p | hsa_circ_0040921 |
| hsa-miR-4745-5p | hsa_circ_0048025 |
| hsa-miR-4745-5p | hsa_circ_0049657 |
| hsa-miR-4745-5p | hsa_circ_0005571 |
| hsa-miR-4745-5p | hsa_circ_0092297 |
| hsa-miR-4745-5p | hsa_circ_0003854 |
| hsa-miR-4745-5p | hsa_circ_0060043 |
| hsa-miR-4745-5p | hsa_circ_0002805 |
| hsa-miR-4745-5p | hsa_circ_0061179 |
| hsa-miR-4745-5p | hsa_circ_0002113 |
| hsa-miR-4745-5p | hsa_circ_0092299 |
| hsa-miR-4745-5p | hsa_circ_0069748 |
| hsa-miR-4745-5p | hsa_circ_0001472 |
| hsa-miR-4745-5p | hsa_circ_0007132 |
| hsa-miR-4745-5p | hsa_circ_0004381 |
| hsa-miR-4745-5p | hsa_circ_0003340 |
| hsa-miR-4745-5p | hsa_circ_0002094 |
| hsa-miR-4745-5p | hsa_circ_0005630 |
| hsa-miR-4745-3p | hsa_circ_0007437 |
| hsa-miR-4745-3p | hsa_circ_0007693 |
| hsa-miR-4745-3p | hsa_circ_0012151 |
| hsa-miR-4745-3p | hsa_circ_0016979 |
| hsa-miR-4745-3p | hsa_circ_0020594 |
| hsa-miR-4745-3p | hsa_circ_0033144 |
| hsa-miR-4745-3p | hsa_circ_0000682 |
| hsa-miR-4745-3p | hsa_circ_0040823 |
| hsa-miR-4745-3p | hsa_circ_0045890 |
| hsa-miR-4745-3p | hsa_circ_0048025 |
| hsa-miR-4745-3p | hsa_circ_0064136 |
| hsa-miR-4745-3p | hsa_circ_0001400 |
| hsa-miR-4745-3p | hsa_circ_0007132 |
| hsa-miR-4745-3p | hsa_circ_0075748 |
| hsa-miR-4745-3p | hsa_circ_0002755 |
| hsa-miR-4745-3p | hsa_circ_0081343 |
| hsa-miR-4745-3p | hsa_circ_0084552 |
| hsa-miR-4746-5p | hsa_circ_0048025 |
| hsa-miR-4746-5p | hsa_circ_0049657 |
| hsa-miR-4746-5p | hsa_circ_0007132 |
| hsa-miR-4746-5p | hsa_circ_0002755 |
| hsa-miR-4746-5p | hsa_circ_0001756 |
| hsa-miR-4746-5p | hsa_circ_0005630 |
| hsa-miR-4746-3p | hsa_circ_0017248 |
| hsa-miR-4746-3p | hsa_circ_0022383 |
| hsa-miR-4746-3p | hsa_circ_0000463 |
| hsa-miR-4746-3p | hsa_circ_0000682 |
| hsa-miR-4746-3p | hsa_circ_0002805 |
| hsa-miR-4746-3p | hsa_circ_0061179 |
| hsa-miR-4746-3p | hsa_circ_0002755 |
| hsa-miR-4746-3p | hsa_circ_0084789 |
| hsa-miR-4746-3p | hsa_circ_0001829 |
| hsa-miR-4747-3p | hsa_circ_0011571 |
| hsa-miR-4747-3p | hsa_circ_0011572 |
| hsa-miR-4747-3p | hsa_circ_0020594 |
| hsa-miR-4747-3p | hsa_circ_0092297 |
| hsa-miR-4747-3p | hsa_circ_0007967 |
| hsa-miR-4747-3p | hsa_circ_0001394 |
| hsa-miR-4747-3p | hsa_circ_0007132 |
| hsa-miR-4749-5p | hsa_circ_0009349 |
| hsa-miR-4749-5p | hsa_circ_0009357 |
| hsa-miR-4749-5p | hsa_circ_0009360 |
| hsa-miR-4749-5p | hsa_circ_0003557 |
| hsa-miR-4749-5p | hsa_circ_0006006 |
| hsa-miR-4749-5p | hsa_circ_0092299 |
| hsa-miR-4749-5p | hsa_circ_0001394 |
| hsa-miR-4749-5p | hsa_circ_0087305 |
| hsa-miR-4749-3p | hsa_circ_0004692 |
| hsa-miR-4749-3p | hsa_circ_0007132 |
| hsa-miR-4750-5p | hsa_circ_0009732 |
| hsa-miR-4750-5p | hsa_circ_0002402 |
| hsa-miR-4750-5p | hsa_circ_0003557 |
| hsa-miR-4750-5p | hsa_circ_0000375 |
| hsa-miR-4750-5p | hsa_circ_0029405 |
| hsa-miR-4750-5p | hsa_circ_0048025 |
| hsa-miR-4750-5p | hsa_circ_0002926 |
| hsa-miR-4750-5p | hsa_circ_0052131 |
| hsa-miR-4750-5p | hsa_circ_0002805 |
| hsa-miR-4750-5p | hsa_circ_0061179 |
| hsa-miR-4750-5p | hsa_circ_0001573 |
| hsa-miR-4750-5p | hsa_circ_0007132 |
| hsa-miR-4750-5p | hsa_circ_0084429 |
| hsa-miR-4750-5p | hsa_circ_0084789 |
| hsa-miR-4750-3p | hsa_circ_0003379 |
| hsa-miR-4750-3p | hsa_circ_0003557 |
| hsa-miR-4750-3p | hsa_circ_0041050 |
| hsa-miR-4750-3p | hsa_circ_0092299 |
| hsa-miR-4750-3p | hsa_circ_0007132 |
| hsa-miR-4750-3p | hsa_circ_0005630 |
| hsa-miR-4751 | hsa_circ_0006693 |
| hsa-miR-4751 | hsa_circ_0001417 |
| hsa-miR-4751 | hsa_circ_0001829 |
| hsa-miR-4753-3p | hsa_circ_0082131 |
| hsa-miR-4754 | hsa_circ_0003379 |
| hsa-miR-4754 | hsa_circ_0040823 |
| hsa-miR-4754 | hsa_circ_0048025 |
| hsa-miR-4754 | hsa_circ_0092297 |
| hsa-miR-4754 | hsa_circ_0002805 |
| hsa-miR-4754 | hsa_circ_0005630 |
| hsa-miR-4754 | hsa_circ_0001829 |
| hsa-miR-4755-5p | hsa_circ_0004705 |
| hsa-miR-4755-3p | hsa_circ_0092297 |
| hsa-miR-4755-3p | hsa_circ_0092299 |
| hsa-miR-4756-5p | hsa_circ_0017289 |
| hsa-miR-4756-5p | hsa_circ_0005090 |
| hsa-miR-4756-5p | hsa_circ_0000463 |
| hsa-miR-4756-5p | hsa_circ_0007846 |
| hsa-miR-4756-5p | hsa_circ_0045890 |
| hsa-miR-4756-5p | hsa_circ_0045905 |
| hsa-miR-4756-5p | hsa_circ_0002926 |
| hsa-miR-4756-5p | hsa_circ_0002805 |
| hsa-miR-4756-5p | hsa_circ_0007132 |
| hsa-miR-4757-5p | hsa_circ_0016863 |
| hsa-miR-4757-5p | hsa_circ_0016867 |
| hsa-miR-4757-5p | hsa_circ_0002805 |
| hsa-miR-4757-5p | hsa_circ_0061179 |
| hsa-miR-4757-5p | hsa_circ_0001329 |
| hsa-miR-4757-5p | hsa_circ_0006884 |
| hsa-miR-4758-5p | hsa_circ_0009349 |
| hsa-miR-4758-5p | hsa_circ_0017289 |
| hsa-miR-4758-5p | hsa_circ_0005090 |
| hsa-miR-4758-5p | hsa_circ_0018909 |
| hsa-miR-4758-5p | hsa_circ_0006649 |
| hsa-miR-4758-5p | hsa_circ_0003379 |
| hsa-miR-4758-5p | hsa_circ_0003557 |
| hsa-miR-4758-5p | hsa_circ_0000375 |
| hsa-miR-4758-5p | hsa_circ_0000417 |
| hsa-miR-4758-5p | hsa_circ_0000497 |
| hsa-miR-4758-5p | hsa_circ_0033144 |
| hsa-miR-4758-5p | hsa_circ_0000651 |
| hsa-miR-4758-5p | hsa_circ_0045890 |
| hsa-miR-4758-5p | hsa_circ_0046430 |
| hsa-miR-4758-5p | hsa_circ_0048025 |
| hsa-miR-4758-5p | hsa_circ_0004003 |
| hsa-miR-4758-5p | hsa_circ_0002926 |
| hsa-miR-4758-5p | hsa_circ_0006877 |
| hsa-miR-4758-5p | hsa_circ_0049998 |
| hsa-miR-4758-5p | hsa_circ_0000918 |
| hsa-miR-4758-5p | hsa_circ_0008030 |
| hsa-miR-4758-5p | hsa_circ_0092299 |
| hsa-miR-4758-5p | hsa_circ_0063331 |
| hsa-miR-4758-5p | hsa_circ_0070467 |
| hsa-miR-4758-5p | hsa_circ_0001472 |
| hsa-miR-4758-5p | hsa_circ_0073379 |
| hsa-miR-4758-5p | hsa_circ_0005540 |
| hsa-miR-4758-5p | hsa_circ_0007132 |
| hsa-miR-4758-5p | hsa_circ_0004381 |
| hsa-miR-4758-5p | hsa_circ_0003340 |
| hsa-miR-4758-5p | hsa_circ_0002755 |
| hsa-miR-4758-5p | hsa_circ_0002094 |
| hsa-miR-4758-3p | hsa_circ_0003838 |
| hsa-miR-4758-3p | hsa_circ_0000996 |
| hsa-miR-4758-3p | hsa_circ_0006693 |
| hsa-miR-4758-3p | hsa_circ_0001417 |
| hsa-miR-4758-3p | hsa_circ_0001756 |
| hsa-miR-4763-5p | hsa_circ_0017639 |
| hsa-miR-4763-5p | hsa_circ_0003379 |
| hsa-miR-4763-5p | hsa_circ_0045890 |
| hsa-miR-4763-5p | hsa_circ_0050119 |
| hsa-miR-4763-5p | hsa_circ_0055904 |
| hsa-miR-4763-5p | hsa_circ_0003854 |
| hsa-miR-4763-5p | hsa_circ_0092299 |
| hsa-miR-4763-5p | hsa_circ_0092283 |
| hsa-miR-4763-5p | hsa_circ_0007132 |
| hsa-miR-4763-5p | hsa_circ_0005630 |
| hsa-miR-4763-3p | hsa_circ_0002402 |
| hsa-miR-4763-3p | hsa_circ_0007015 |
| hsa-miR-4763-3p | hsa_circ_0017438 |
| hsa-miR-4763-3p | hsa_circ_0004277 |
| hsa-miR-4763-3p | hsa_circ_0017446 |
| hsa-miR-4763-3p | hsa_circ_0000206 |
| hsa-miR-4763-3p | hsa_circ_0017461 |
| hsa-miR-4763-3p | hsa_circ_0000213 |
| hsa-miR-4763-3p | hsa_circ_0006649 |
| hsa-miR-4763-3p | hsa_circ_0003379 |
| hsa-miR-4763-3p | hsa_circ_0006254 |
| hsa-miR-4763-3p | hsa_circ_0003768 |
| hsa-miR-4763-3p | hsa_circ_0022383 |
| hsa-miR-4763-3p | hsa_circ_0022392 |
| hsa-miR-4763-3p | hsa_circ_0022723 |
| hsa-miR-4763-3p | hsa_circ_0003557 |
| hsa-miR-4763-3p | hsa_circ_0003855 |
| hsa-miR-4763-3p | hsa_circ_0000417 |
| hsa-miR-4763-3p | hsa_circ_0029633 |
| hsa-miR-4763-3p | hsa_circ_0033144 |
| hsa-miR-4763-3p | hsa_circ_0036763 |
| hsa-miR-4763-3p | hsa_circ_0000650 |
| hsa-miR-4763-3p | hsa_circ_0036768 |
| hsa-miR-4763-3p | hsa_circ_0007146 |
| hsa-miR-4763-3p | hsa_circ_0007846 |
| hsa-miR-4763-3p | hsa_circ_0002696 |
| hsa-miR-4763-3p | hsa_circ_0040823 |
| hsa-miR-4763-3p | hsa_circ_0040921 |
| hsa-miR-4763-3p | hsa_circ_0045890 |
| hsa-miR-4763-3p | hsa_circ_0046292 |
| hsa-miR-4763-3p | hsa_circ_0046430 |
| hsa-miR-4763-3p | hsa_circ_0048025 |
| hsa-miR-4763-3p | hsa_circ_0002926 |
| hsa-miR-4763-3p | hsa_circ_0006877 |
| hsa-miR-4763-3p | hsa_circ_0049657 |
| hsa-miR-4763-3p | hsa_circ_0049998 |
| hsa-miR-4763-3p | hsa_circ_0050119 |
| hsa-miR-4763-3p | hsa_circ_0000918 |
| hsa-miR-4763-3p | hsa_circ_0008030 |
| hsa-miR-4763-3p | hsa_circ_0000950 |
| hsa-miR-4763-3p | hsa_circ_0092297 |
| hsa-miR-4763-3p | hsa_circ_0006006 |
| hsa-miR-4763-3p | hsa_circ_0003854 |
| hsa-miR-4763-3p | hsa_circ_0004218 |
| hsa-miR-4763-3p | hsa_circ_0061052 |
| hsa-miR-4763-3p | hsa_circ_0002805 |
| hsa-miR-4763-3p | hsa_circ_0061179 |
| hsa-miR-4763-3p | hsa_circ_0064136 |
| hsa-miR-4763-3p | hsa_circ_0001394 |
| hsa-miR-4763-3p | hsa_circ_0070467 |
| hsa-miR-4763-3p | hsa_circ_0071311 |
| hsa-miR-4763-3p | hsa_circ_0071375 |
| hsa-miR-4763-3p | hsa_circ_0001472 |
| hsa-miR-4763-3p | hsa_circ_0072437 |
| hsa-miR-4763-3p | hsa_circ_0001492 |
| hsa-miR-4763-3p | hsa_circ_0073379 |
| hsa-miR-4763-3p | hsa_circ_0005540 |
| hsa-miR-4763-3p | hsa_circ_0001549 |
| hsa-miR-4763-3p | hsa_circ_0001573 |
| hsa-miR-4763-3p | hsa_circ_0007132 |
| hsa-miR-4763-3p | hsa_circ_0002037 |
| hsa-miR-4763-3p | hsa_circ_0001658 |
| hsa-miR-4763-3p | hsa_circ_0004381 |
| hsa-miR-4763-3p | hsa_circ_0003340 |
| hsa-miR-4763-3p | hsa_circ_0002755 |
| hsa-miR-4763-3p | hsa_circ_0001748 |
| hsa-miR-4763-3p | hsa_circ_0001756 |
| hsa-miR-4763-3p | hsa_circ_0006357 |
| hsa-miR-4763-3p | hsa_circ_0002094 |
| hsa-miR-4763-3p | hsa_circ_0005630 |
| hsa-miR-4763-3p | hsa_circ_0001806 |
| hsa-miR-4763-3p | hsa_circ_0084789 |
| hsa-miR-4763-3p | hsa_circ_0001829 |
| hsa-miR-4763-3p | hsa_circ_0087288 |
| hsa-miR-4763-3p | hsa_circ_0087305 |
| hsa-miR-4763-3p | hsa_circ_0087855 |
| hsa-miR-4763-3p | hsa_circ_0008812 |
| hsa-miR-4763-3p | hsa_circ_0006174 |
| hsa-miR-4763-3p | hsa_circ_0087861 |
| hsa-miR-4763-3p | hsa_circ_0087862 |
| hsa-miR-4763-3p | hsa_circ_0001947 |
| hsa-miR-4767 | hsa_circ_0009349 |
| hsa-miR-4767 | hsa_circ_0009357 |
| hsa-miR-4767 | hsa_circ_0009360 |
| hsa-miR-4767 | hsa_circ_0007001 |
| hsa-miR-4767 | hsa_circ_0022723 |
| hsa-miR-4767 | hsa_circ_0007846 |
| hsa-miR-4767 | hsa_circ_0000690 |
| hsa-miR-4767 | hsa_circ_0040823 |
| hsa-miR-4767 | hsa_circ_0040921 |
| hsa-miR-4767 | hsa_circ_0043522 |
| hsa-miR-4767 | hsa_circ_0045890 |
| hsa-miR-4767 | hsa_circ_0046430 |
| hsa-miR-4767 | hsa_circ_0006877 |
| hsa-miR-4767 | hsa_circ_0049998 |
| hsa-miR-4767 | hsa_circ_0050119 |
| hsa-miR-4767 | hsa_circ_0002805 |
| hsa-miR-4767 | hsa_circ_0061179 |
| hsa-miR-4767 | hsa_circ_0092299 |
| hsa-miR-4767 | hsa_circ_0001394 |
| hsa-miR-4767 | hsa_circ_0007132 |
| hsa-miR-4767 | hsa_circ_0075748 |
| hsa-miR-4767 | hsa_circ_0081343 |
| hsa-miR-4767 | hsa_circ_0002094 |
| hsa-miR-4767 | hsa_circ_0001806 |
| hsa-miR-4768-5p | hsa_circ_0072797 |
| hsa-miR-4769-5p | hsa_circ_0007372 |
| hsa-miR-4769-5p | hsa_circ_0049998 |
| hsa-miR-4769-5p | hsa_circ_0007132 |
| hsa-miR-4769-3p | hsa_circ_0033144 |
| hsa-miR-4769-3p | hsa_circ_0004692 |
| hsa-miR-4776-5p | hsa_circ_0000417 |
| hsa-miR-4776-5p | hsa_circ_0001394 |
| hsa-miR-4776-3p | hsa_circ_0004089 |
| hsa-miR-4776-3p | hsa_circ_0007132 |
| hsa-miR-4780 | hsa_circ_0092283 |
| hsa-miR-4436b-5p | hsa_circ_0020594 |
| hsa-miR-4436b-5p | hsa_circ_0034920 |
| hsa-miR-4436b-5p | hsa_circ_0050119 |
| hsa-miR-4436b-5p | hsa_circ_0055904 |
| hsa-miR-4436b-5p | hsa_circ_0067913 |
| hsa-miR-4436b-3p | hsa_circ_0000417 |
| hsa-miR-4436b-3p | hsa_circ_0003854 |
| hsa-miR-4436b-3p | hsa_circ_0084789 |
| hsa-miR-4783-5p | hsa_circ_0005664 |
| hsa-miR-4783-5p | hsa_circ_0008102 |
| hsa-miR-4783-5p | hsa_circ_0007372 |
| hsa-miR-4783-5p | hsa_circ_0000690 |
| hsa-miR-4783-5p | hsa_circ_0002926 |
| hsa-miR-4783-5p | hsa_circ_0002805 |
| hsa-miR-4783-5p | hsa_circ_0061179 |
| hsa-miR-4783-5p | hsa_circ_0070396 |
| hsa-miR-4783-5p | hsa_circ_0071311 |
| hsa-miR-4783-5p | hsa_circ_0007132 |
| hsa-miR-4783-5p | hsa_circ_0005630 |
| hsa-miR-4783-3p | hsa_circ_0000131 |
| hsa-miR-4783-3p | hsa_circ_0006608 |
| hsa-miR-4783-3p | hsa_circ_0018909 |
| hsa-miR-4783-3p | hsa_circ_0000497 |
| hsa-miR-4783-3p | hsa_circ_0033144 |
| hsa-miR-4783-3p | hsa_circ_0035381 |
| hsa-miR-4783-3p | hsa_circ_0007146 |
| hsa-miR-4783-3p | hsa_circ_0045890 |
| hsa-miR-4783-3p | hsa_circ_0046430 |
| hsa-miR-4783-3p | hsa_circ_0002926 |
| hsa-miR-4783-3p | hsa_circ_0002805 |
| hsa-miR-4783-3p | hsa_circ_0061179 |
| hsa-miR-4783-3p | hsa_circ_0092299 |
| hsa-miR-4783-3p | hsa_circ_0069399 |
| hsa-miR-4783-3p | hsa_circ_0001400 |
| hsa-miR-4783-3p | hsa_circ_0007132 |
| hsa-miR-4783-3p | hsa_circ_0004381 |
| hsa-miR-4783-3p | hsa_circ_0001910 |
| hsa-miR-4783-3p | hsa_circ_0001934 |
| hsa-miR-4784 | hsa_circ_0002755 |
| hsa-miR-4785 | hsa_circ_0007009 |
| hsa-miR-4785 | hsa_circ_0045890 |
| hsa-miR-4785 | hsa_circ_0004705 |
| hsa-miR-2467-3p | hsa_circ_0002805 |
| hsa-miR-2467-3p | hsa_circ_0061179 |
| hsa-miR-2467-3p | hsa_circ_0004692 |
| hsa-miR-2467-3p | hsa_circ_0065147 |
| hsa-miR-2467-3p | hsa_circ_0065149 |
| hsa-miR-2467-3p | hsa_circ_0002569 |
| hsa-miR-4786-3p | hsa_circ_0000673 |
| hsa-miR-4786-3p | hsa_circ_0002805 |
| hsa-miR-4786-3p | hsa_circ_0092299 |
| hsa-miR-4786-3p | hsa_circ_0072437 |
| hsa-miR-4786-3p | hsa_circ_0007132 |
| hsa-miR-4786-3p | hsa_circ_0005630 |
| hsa-miR-4787-5p | hsa_circ_0009732 |
| hsa-miR-4787-5p | hsa_circ_0002402 |
| hsa-miR-4787-5p | hsa_circ_0007015 |
| hsa-miR-4787-5p | hsa_circ_0000206 |
| hsa-miR-4787-5p | hsa_circ_0017461 |
| hsa-miR-4787-5p | hsa_circ_0022723 |
| hsa-miR-4787-5p | hsa_circ_0000375 |
| hsa-miR-4787-5p | hsa_circ_0000417 |
| hsa-miR-4787-5p | hsa_circ_0007846 |
| hsa-miR-4787-5p | hsa_circ_0040823 |
| hsa-miR-4787-5p | hsa_circ_0045890 |
| hsa-miR-4787-5p | hsa_circ_0045905 |
| hsa-miR-4787-5p | hsa_circ_0046430 |
| hsa-miR-4787-5p | hsa_circ_0048025 |
| hsa-miR-4787-5p | hsa_circ_0050119 |
| hsa-miR-4787-5p | hsa_circ_0000918 |
| hsa-miR-4787-5p | hsa_circ_0055904 |
| hsa-miR-4787-5p | hsa_circ_0002805 |
| hsa-miR-4787-5p | hsa_circ_0061179 |
| hsa-miR-4787-5p | hsa_circ_0002113 |
| hsa-miR-4787-5p | hsa_circ_0001400 |
| hsa-miR-4787-5p | hsa_circ_0070467 |
| hsa-miR-4787-5p | hsa_circ_0001577 |
| hsa-miR-4787-5p | hsa_circ_0001578 |
| hsa-miR-4787-5p | hsa_circ_0007132 |
| hsa-miR-4787-5p | hsa_circ_0077765 |
| hsa-miR-4787-5p | hsa_circ_0004381 |
| hsa-miR-4787-5p | hsa_circ_0002094 |
| hsa-miR-4787-5p | hsa_circ_0006566 |
| hsa-miR-4787-3p | hsa_circ_0009732 |
| hsa-miR-4787-3p | hsa_circ_0007009 |
| hsa-miR-4787-3p | hsa_circ_0007146 |
| hsa-miR-4787-3p | hsa_circ_0040823 |
| hsa-miR-4787-3p | hsa_circ_0092337 |
| hsa-miR-4787-3p | hsa_circ_0045890 |
| hsa-miR-4787-3p | hsa_circ_0050119 |
| hsa-miR-4787-3p | hsa_circ_0004692 |
| hsa-miR-4787-3p | hsa_circ_0065147 |
| hsa-miR-4787-3p | hsa_circ_0065149 |
| hsa-miR-4787-3p | hsa_circ_0002569 |
| hsa-miR-4787-3p | hsa_circ_0006693 |
| hsa-miR-4787-3p | hsa_circ_0007132 |
| hsa-miR-4787-3p | hsa_circ_0001756 |
| hsa-miR-4787-3p | hsa_circ_0005630 |
| hsa-miR-4787-3p | hsa_circ_0008113 |
| hsa-miR-4787-3p | hsa_circ_0087305 |
| hsa-miR-4793-5p | hsa_circ_0035649 |
| hsa-miR-4793-5p | hsa_circ_0000613 |
| hsa-miR-4793-5p | hsa_circ_0008153 |
| hsa-miR-4793-5p | hsa_circ_0000835 |
| hsa-miR-4793-5p | hsa_circ_0000836 |
| hsa-miR-4793-5p | hsa_circ_0061936 |
| hsa-miR-4793-5p | hsa_circ_0092299 |
| hsa-miR-4793-5p | hsa_circ_0064136 |
| hsa-miR-4793-5p | hsa_circ_0005630 |
| hsa-miR-4793-3p | hsa_circ_0040823 |
| hsa-miR-4797-3p | hsa_circ_0001756 |
| hsa-miR-4800-5p | hsa_circ_0017438 |
| hsa-miR-4800-5p | hsa_circ_0030051 |
| hsa-miR-5001-5p | hsa_circ_0000417 |
| hsa-miR-5001-5p | hsa_circ_0000660 |
| hsa-miR-5001-5p | hsa_circ_0002696 |
| hsa-miR-5001-5p | hsa_circ_0005733 |
| hsa-miR-5001-5p | hsa_circ_0040039 |
| hsa-miR-5001-5p | hsa_circ_0046430 |
| hsa-miR-5001-5p | hsa_circ_0000918 |
| hsa-miR-5001-5p | hsa_circ_0092297 |
| hsa-miR-5001-5p | hsa_circ_0003854 |
| hsa-miR-5001-5p | hsa_circ_0002805 |
| hsa-miR-5001-5p | hsa_circ_0061179 |
| hsa-miR-5001-5p | hsa_circ_0092283 |
| hsa-miR-5001-5p | hsa_circ_0066444 |
| hsa-miR-5001-5p | hsa_circ_0001394 |
| hsa-miR-5001-5p | hsa_circ_0007132 |
| hsa-miR-5001-5p | hsa_circ_0008536 |
| hsa-miR-5001-5p | hsa_circ_0077765 |
| hsa-miR-5001-5p | hsa_circ_0002755 |
| hsa-miR-5001-5p | hsa_circ_0005630 |
| hsa-miR-5001-3p | hsa_circ_0020594 |
| hsa-miR-5001-3p | hsa_circ_0000909 |
| hsa-miR-5004-5p | hsa_circ_0030051 |
| hsa-miR-5004-3p | hsa_circ_0003315 |
| hsa-miR-5004-3p | hsa_circ_0046430 |
| hsa-miR-5006-5p | hsa_circ_0009732 |
| hsa-miR-5006-5p | hsa_circ_0007015 |
| hsa-miR-5006-5p | hsa_circ_0004218 |
| hsa-miR-5006-5p | hsa_circ_0002805 |
| hsa-miR-5006-5p | hsa_circ_0061179 |
| hsa-miR-5006-5p | hsa_circ_0001748 |
| hsa-miR-5006-5p | hsa_circ_0087305 |
| hsa-miR-5006-5p | hsa_circ_0092125 |
| hsa-miR-5006-3p | hsa_circ_0003768 |
| hsa-miR-5006-3p | hsa_circ_0040921 |
| hsa-miR-5008-5p | hsa_circ_0003379 |
| hsa-miR-5008-5p | hsa_circ_0000395 |
| hsa-miR-5008-5p | hsa_circ_0048025 |
| hsa-miR-5008-5p | hsa_circ_0049657 |
| hsa-miR-5008-5p | hsa_circ_0004218 |
| hsa-miR-5008-5p | hsa_circ_0092299 |
| hsa-miR-5008-5p | hsa_circ_0001439 |
| hsa-miR-5008-5p | hsa_circ_0007132 |
| hsa-miR-5008-5p | hsa_circ_0006566 |
| hsa-miR-5008-3p | hsa_circ_0009732 |
| hsa-miR-5008-3p | hsa_circ_0000395 |
| hsa-miR-5008-3p | hsa_circ_0046430 |
| hsa-miR-5008-3p | hsa_circ_0058522 |
| hsa-miR-5008-3p | hsa_circ_0007609 |
| hsa-miR-5008-3p | hsa_circ_0001400 |
| hsa-miR-5008-3p | hsa_circ_0006528 |
| hsa-miR-5008-3p | hsa_circ_0001829 |
| hsa-miR-5087 | hsa_circ_0007015 |
| hsa-miR-5087 | hsa_circ_0045890 |
| hsa-miR-5088-5p | hsa_circ_0022392 |
| hsa-miR-5088-5p | hsa_circ_0029633 |
| hsa-miR-5088-5p | hsa_circ_0000650 |
| hsa-miR-5088-5p | hsa_circ_0036768 |
| hsa-miR-5088-5p | hsa_circ_0007846 |
| hsa-miR-5088-5p | hsa_circ_0040921 |
| hsa-miR-5088-5p | hsa_circ_0092337 |
| hsa-miR-5088-5p | hsa_circ_0045890 |
| hsa-miR-5088-5p | hsa_circ_0000909 |
| hsa-miR-5088-5p | hsa_circ_0008030 |
| hsa-miR-5088-5p | hsa_circ_0070467 |
| hsa-miR-5088-5p | hsa_circ_0076793 |
| hsa-miR-5088-5p | hsa_circ_0004381 |
| hsa-miR-5088-5p | hsa_circ_0002755 |
| hsa-miR-5088-5p | hsa_circ_0005630 |
| hsa-miR-5088-3p | hsa_circ_0011571 |
| hsa-miR-5088-3p | hsa_circ_0011572 |
| hsa-miR-5088-3p | hsa_circ_0031933 |
| hsa-miR-5088-3p | hsa_circ_0092283 |
| hsa-miR-5088-3p | hsa_circ_0003602 |
| hsa-miR-5090 | hsa_circ_0022383 |
| hsa-miR-5090 | hsa_circ_0022723 |
| hsa-miR-5090 | hsa_circ_0003557 |
| hsa-miR-5090 | hsa_circ_0025388 |
| hsa-miR-5090 | hsa_circ_0007137 |
| hsa-miR-5090 | hsa_circ_0001549 |
| hsa-miR-5090 | hsa_circ_0007132 |
| hsa-miR-5090 | hsa_circ_0001756 |
| hsa-miR-5090 | hsa_circ_0002094 |
| hsa-miR-5090 | hsa_circ_0005630 |
| hsa-miR-5095 | hsa_circ_0000973 |
| hsa-miR-5095 | hsa_circ_0002094 |
| hsa-miR-1273f | hsa_circ_0029405 |
| hsa-miR-1273f | hsa_circ_0000973 |
| hsa-miR-1273g-5p | hsa_circ_0029405 |
| hsa-miR-1273g-5p | hsa_circ_0082179 |
| hsa-miR-1273g-3p | hsa_circ_0008788 |
| hsa-miR-1273g-3p | hsa_circ_0029405 |
| hsa-miR-1273g-3p | hsa_circ_0000973 |
| hsa-miR-1273g-3p | hsa_circ_0082179 |
| hsa-miR-5096 | hsa_circ_0020594 |
| hsa-miR-5187-5p | hsa_circ_0045890 |
| hsa-miR-5187-5p | hsa_circ_0001756 |
| hsa-miR-5189-5p | hsa_circ_0003379 |
| hsa-miR-5189-5p | hsa_circ_0000417 |
| hsa-miR-5189-5p | hsa_circ_0007146 |
| hsa-miR-5189-5p | hsa_circ_0007846 |
| hsa-miR-5189-5p | hsa_circ_0040823 |
| hsa-miR-5189-5p | hsa_circ_0045890 |
| hsa-miR-5189-5p | hsa_circ_0045905 |
| hsa-miR-5189-5p | hsa_circ_0000835 |
| hsa-miR-5189-5p | hsa_circ_0000836 |
| hsa-miR-5189-5p | hsa_circ_0000909 |
| hsa-miR-5189-5p | hsa_circ_0061052 |
| hsa-miR-5189-5p | hsa_circ_0002805 |
| hsa-miR-5189-5p | hsa_circ_0001394 |
| hsa-miR-5189-5p | hsa_circ_0001400 |
| hsa-miR-5189-5p | hsa_circ_0001439 |
| hsa-miR-5189-5p | hsa_circ_0001573 |
| hsa-miR-5189-5p | hsa_circ_0007132 |
| hsa-miR-5189-5p | hsa_circ_0087305 |
| hsa-miR-5189-5p | hsa_circ_0092125 |
| hsa-miR-5190 | hsa_circ_0004699 |
| hsa-miR-5190 | hsa_circ_0005630 |
| hsa-miR-5192 | hsa_circ_0046430 |
| hsa-miR-5193 | hsa_circ_0002805 |
| hsa-miR-5193 | hsa_circ_0007132 |
| hsa-miR-5193 | hsa_circ_0005630 |
| hsa-miR-5194 | hsa_circ_0007846 |
| hsa-miR-5196-5p | hsa_circ_0033144 |
| hsa-miR-5196-5p | hsa_circ_0002696 |
| hsa-miR-5196-5p | hsa_circ_0040039 |
| hsa-miR-5196-5p | hsa_circ_0004354 |
| hsa-miR-5196-5p | hsa_circ_0046430 |
| hsa-miR-5196-5p | hsa_circ_0092299 |
| hsa-miR-5196-5p | hsa_circ_0002077 |
| hsa-miR-5196-5p | hsa_circ_0075748 |
| hsa-miR-5196-5p | hsa_circ_0004381 |
| hsa-miR-5196-5p | hsa_circ_0003340 |
| hsa-miR-5196-5p | hsa_circ_0087855 |
| hsa-miR-5196-5p | hsa_circ_0008812 |
| hsa-miR-5196-5p | hsa_circ_0006174 |
| hsa-miR-5196-5p | hsa_circ_0087861 |
| hsa-miR-5196-5p | hsa_circ_0087862 |
| hsa-miR-5196-3p | hsa_circ_0020594 |
| hsa-miR-5196-3p | hsa_circ_0007132 |
| hsa-miR-5196-3p | hsa_circ_0002094 |
| hsa-miR-5196-3p | hsa_circ_0005630 |
| hsa-miR-5196-3p | hsa_circ_0084789 |
| hsa-miR-5571-3p | hsa_circ_0022383 |
| hsa-miR-5572 | hsa_circ_0001829 |
| hsa-miR-664b-5p | hsa_circ_0022505 |
| hsa-miR-664b-5p | hsa_circ_0000650 |
| hsa-miR-664b-5p | hsa_circ_0036768 |
| hsa-miR-664b-5p | hsa_circ_0001756 |
| hsa-miR-664b-5p | hsa_circ_0084789 |
| hsa-miR-664b-3p | hsa_circ_0020594 |
| hsa-miR-5584-5p | hsa_circ_0003838 |
| hsa-miR-5587-3p | hsa_circ_0007132 |
| hsa-miR-1295b-3p | hsa_circ_0004705 |
| hsa-miR-5589-5p | hsa_circ_0092277 |
| hsa-miR-5589-5p | hsa_circ_0005630 |
| hsa-miR-5684 | hsa_circ_0029405 |
| hsa-miR-5684 | hsa_circ_0000973 |
| hsa-miR-5685 | hsa_circ_0020594 |
| hsa-miR-5691 | hsa_circ_0033144 |
| hsa-miR-5698 | hsa_circ_0007015 |
| hsa-miR-5698 | hsa_circ_0006649 |
| hsa-miR-5698 | hsa_circ_0062397 |
| hsa-miR-5698 | hsa_circ_0066444 |
| hsa-miR-5699-3p | hsa_circ_0040921 |
| hsa-miR-5699-3p | hsa_circ_0048025 |
| hsa-miR-5699-3p | hsa_circ_0054086 |
| hsa-miR-5705 | hsa_circ_0009349 |
| hsa-miR-5705 | hsa_circ_0007132 |
| hsa-miR-5787 | hsa_circ_0007015 |
| hsa-miR-5787 | hsa_circ_0018909 |
| hsa-miR-5787 | hsa_circ_0003379 |
| hsa-miR-5787 | hsa_circ_0022383 |
| hsa-miR-5787 | hsa_circ_0007372 |
| hsa-miR-5787 | hsa_circ_0000417 |
| hsa-miR-5787 | hsa_circ_0029405 |
| hsa-miR-5787 | hsa_circ_0031933 |
| hsa-miR-5787 | hsa_circ_0007846 |
| hsa-miR-5787 | hsa_circ_0044195 |
| hsa-miR-5787 | hsa_circ_0046430 |
| hsa-miR-5787 | hsa_circ_0049657 |
| hsa-miR-5787 | hsa_circ_0049998 |
| hsa-miR-5787 | hsa_circ_0050119 |
| hsa-miR-5787 | hsa_circ_0052131 |
| hsa-miR-5787 | hsa_circ_0003854 |
| hsa-miR-5787 | hsa_circ_0064136 |
| hsa-miR-5787 | hsa_circ_0001394 |
| hsa-miR-5787 | hsa_circ_0001400 |
| hsa-miR-5787 | hsa_circ_0070467 |
| hsa-miR-5787 | hsa_circ_0071311 |
| hsa-miR-5787 | hsa_circ_0001549 |
| hsa-miR-5787 | hsa_circ_0001573 |
| hsa-miR-5787 | hsa_circ_0007132 |
| hsa-miR-5787 | hsa_circ_0002755 |
| hsa-miR-5787 | hsa_circ_0001748 |
| hsa-miR-5787 | hsa_circ_0002094 |
| hsa-miR-5787 | hsa_circ_0084429 |
| hsa-miR-5787 | hsa_circ_0001829 |
| hsa-miR-5787 | hsa_circ_0087288 |
| hsa-miR-1199-5p | hsa_circ_0017446 |
| hsa-miR-1199-5p | hsa_circ_0000206 |
| hsa-miR-1199-5p | hsa_circ_0034189 |
| hsa-miR-1199-5p | hsa_circ_0092299 |
| hsa-miR-1199-5p | hsa_circ_0004705 |
| hsa-miR-1199-5p | hsa_circ_0006884 |
| hsa-miR-1199-5p | hsa_circ_0091382 |
| hsa-miR-6068 | hsa_circ_0002926 |
| hsa-miR-6068 | hsa_circ_0002094 |
| hsa-miR-6069 | hsa_circ_0006254 |
| hsa-miR-6069 | hsa_circ_0003768 |
| hsa-miR-6069 | hsa_circ_0000690 |
| hsa-miR-6069 | hsa_circ_0002805 |
| hsa-miR-6069 | hsa_circ_0061179 |
| hsa-miR-6069 | hsa_circ_0092299 |
| hsa-miR-6069 | hsa_circ_0092283 |
| hsa-miR-6069 | hsa_circ_0004692 |
| hsa-miR-6070 | hsa_circ_0084789 |
| hsa-miR-6075 | hsa_circ_0007437 |
| hsa-miR-6075 | hsa_circ_0007693 |
| hsa-miR-6075 | hsa_circ_0012151 |
| hsa-miR-6075 | hsa_circ_0004470 |
| hsa-miR-6076 | hsa_circ_0002037 |
| hsa-miR-6081 | hsa_circ_0015004 |
| hsa-miR-6081 | hsa_circ_0007015 |
| hsa-miR-6081 | hsa_circ_0040921 |
| hsa-miR-6081 | hsa_circ_0046430 |
| hsa-miR-6081 | hsa_circ_0048025 |
| hsa-miR-6081 | hsa_circ_0002926 |
| hsa-miR-6081 | hsa_circ_0006877 |
| hsa-miR-6081 | hsa_circ_0002805 |
| hsa-miR-6081 | hsa_circ_0061179 |
| hsa-miR-6081 | hsa_circ_0001329 |
| hsa-miR-6081 | hsa_circ_0006884 |
| hsa-miR-6081 | hsa_circ_0001400 |
| hsa-miR-6081 | hsa_circ_0007132 |
| hsa-miR-6084 | hsa_circ_0011571 |
| hsa-miR-6084 | hsa_circ_0011572 |
| hsa-miR-6084 | hsa_circ_0022505 |
| hsa-miR-6084 | hsa_circ_0000375 |
| hsa-miR-6084 | hsa_circ_0004519 |
| hsa-miR-6084 | hsa_circ_0048025 |
| hsa-miR-6084 | hsa_circ_0006884 |
| hsa-miR-6084 | hsa_circ_0082131 |
| hsa-miR-6086 | hsa_circ_0017248 |
| hsa-miR-6086 | hsa_circ_0007846 |
| hsa-miR-6087 | hsa_circ_0009581 |
| hsa-miR-6087 | hsa_circ_0006837 |
| hsa-miR-6087 | hsa_circ_0003379 |
| hsa-miR-6087 | hsa_circ_0002805 |
| hsa-miR-6087 | hsa_circ_0002094 |
| hsa-miR-6089 | hsa_circ_0009349 |
| hsa-miR-6089 | hsa_circ_0009357 |
| hsa-miR-6089 | hsa_circ_0009360 |
| hsa-miR-6089 | hsa_circ_0009581 |
| hsa-miR-6089 | hsa_circ_0006837 |
| hsa-miR-6089 | hsa_circ_0009732 |
| hsa-miR-6089 | hsa_circ_0002402 |
| hsa-miR-6089 | hsa_circ_0007437 |
| hsa-miR-6089 | hsa_circ_0007693 |
| hsa-miR-6089 | hsa_circ_0012151 |
| hsa-miR-6089 | hsa_circ_0007009 |
| hsa-miR-6089 | hsa_circ_0014132 |
| hsa-miR-6089 | hsa_circ_0000130 |
| hsa-miR-6089 | hsa_circ_0000131 |
| hsa-miR-6089 | hsa_circ_0015004 |
| hsa-miR-6089 | hsa_circ_0015966 |
| hsa-miR-6089 | hsa_circ_0007015 |
| hsa-miR-6089 | hsa_circ_0016863 |
| hsa-miR-6089 | hsa_circ_0016867 |
| hsa-miR-6089 | hsa_circ_0017289 |
| hsa-miR-6089 | hsa_circ_0005090 |
| hsa-miR-6089 | hsa_circ_0000206 |
| hsa-miR-6089 | hsa_circ_0017461 |
| hsa-miR-6089 | hsa_circ_0000247 |
| hsa-miR-6089 | hsa_circ_0018909 |
| hsa-miR-6089 | hsa_circ_0006649 |
| hsa-miR-6089 | hsa_circ_0008102 |
| hsa-miR-6089 | hsa_circ_0003168 |
| hsa-miR-6089 | hsa_circ_0020594 |
| hsa-miR-6089 | hsa_circ_0003379 |
| hsa-miR-6089 | hsa_circ_0006254 |
| hsa-miR-6089 | hsa_circ_0003768 |
| hsa-miR-6089 | hsa_circ_0022383 |
| hsa-miR-6089 | hsa_circ_0022723 |
| hsa-miR-6089 | hsa_circ_0003557 |
| hsa-miR-6089 | hsa_circ_0007372 |
| hsa-miR-6089 | hsa_circ_0000375 |
| hsa-miR-6089 | hsa_circ_0000417 |
| hsa-miR-6089 | hsa_circ_0029405 |
| hsa-miR-6089 | hsa_circ_0029633 |
| hsa-miR-6089 | hsa_circ_0029976 |
| hsa-miR-6089 | hsa_circ_0000497 |
| hsa-miR-6089 | hsa_circ_0004137 |
| hsa-miR-6089 | hsa_circ_0033144 |
| hsa-miR-6089 | hsa_circ_0035649 |
| hsa-miR-6089 | hsa_circ_0000613 |
| hsa-miR-6089 | hsa_circ_0008153 |
| hsa-miR-6089 | hsa_circ_0036763 |
| hsa-miR-6089 | hsa_circ_0000650 |
| hsa-miR-6089 | hsa_circ_0036768 |
| hsa-miR-6089 | hsa_circ_0007146 |
| hsa-miR-6089 | hsa_circ_0007846 |
| hsa-miR-6089 | hsa_circ_0040823 |
| hsa-miR-6089 | hsa_circ_0040921 |
| hsa-miR-6089 | hsa_circ_0045890 |
| hsa-miR-6089 | hsa_circ_0045905 |
| hsa-miR-6089 | hsa_circ_0046430 |
| hsa-miR-6089 | hsa_circ_0048025 |
| hsa-miR-6089 | hsa_circ_0004003 |
| hsa-miR-6089 | hsa_circ_0004891 |
| hsa-miR-6089 | hsa_circ_0002926 |
| hsa-miR-6089 | hsa_circ_0006877 |
| hsa-miR-6089 | hsa_circ_0049657 |
| hsa-miR-6089 | hsa_circ_0000909 |
| hsa-miR-6089 | hsa_circ_0049998 |
| hsa-miR-6089 | hsa_circ_0050119 |
| hsa-miR-6089 | hsa_circ_0000936 |
| hsa-miR-6089 | hsa_circ_0052131 |
| hsa-miR-6089 | hsa_circ_0000973 |
| hsa-miR-6089 | hsa_circ_0000996 |
| hsa-miR-6089 | hsa_circ_0092297 |
| hsa-miR-6089 | hsa_circ_0003854 |
| hsa-miR-6089 | hsa_circ_0060043 |
| hsa-miR-6089 | hsa_circ_0004218 |
| hsa-miR-6089 | hsa_circ_0061052 |
| hsa-miR-6089 | hsa_circ_0002805 |
| hsa-miR-6089 | hsa_circ_0061179 |
| hsa-miR-6089 | hsa_circ_0007609 |
| hsa-miR-6089 | hsa_circ_0002113 |
| hsa-miR-6089 | hsa_circ_0061936 |
| hsa-miR-6089 | hsa_circ_0092299 |
| hsa-miR-6089 | hsa_circ_0004470 |
| hsa-miR-6089 | hsa_circ_0063331 |
| hsa-miR-6089 | hsa_circ_0002077 |
| hsa-miR-6089 | hsa_circ_0064136 |
| hsa-miR-6089 | hsa_circ_0004692 |
| hsa-miR-6089 | hsa_circ_0065147 |
| hsa-miR-6089 | hsa_circ_0065149 |
| hsa-miR-6089 | hsa_circ_0002569 |
| hsa-miR-6089 | hsa_circ_0003602 |
| hsa-miR-6089 | hsa_circ_0092277 |
| hsa-miR-6089 | hsa_circ_0001394 |
| hsa-miR-6089 | hsa_circ_0001400 |
| hsa-miR-6089 | hsa_circ_0069748 |
| hsa-miR-6089 | hsa_circ_0006693 |
| hsa-miR-6089 | hsa_circ_0001417 |
| hsa-miR-6089 | hsa_circ_0070467 |
| hsa-miR-6089 | hsa_circ_0071311 |
| hsa-miR-6089 | hsa_circ_0073379 |
| hsa-miR-6089 | hsa_circ_0005540 |
| hsa-miR-6089 | hsa_circ_0074371 |
| hsa-miR-6089 | hsa_circ_0001549 |
| hsa-miR-6089 | hsa_circ_0001573 |
| hsa-miR-6089 | hsa_circ_0001577 |
| hsa-miR-6089 | hsa_circ_0001578 |
| hsa-miR-6089 | hsa_circ_0007132 |
| hsa-miR-6089 | hsa_circ_0076742 |
| hsa-miR-6089 | hsa_circ_0004136 |
| hsa-miR-6089 | hsa_circ_0004712 |
| hsa-miR-6089 | hsa_circ_0004381 |
| hsa-miR-6089 | hsa_circ_0078522 |
| hsa-miR-6089 | hsa_circ_0009092 |
| hsa-miR-6089 | hsa_circ_0002755 |
| hsa-miR-6089 | hsa_circ_0082179 |
| hsa-miR-6089 | hsa_circ_0001748 |
| hsa-miR-6089 | hsa_circ_0001756 |
| hsa-miR-6089 | hsa_circ_0002094 |
| hsa-miR-6089 | hsa_circ_0005630 |
| hsa-miR-6089 | hsa_circ_0084429 |
| hsa-miR-6089 | hsa_circ_0084615 |
| hsa-miR-6089 | hsa_circ_0084789 |
| hsa-miR-6089 | hsa_circ_0001829 |
| hsa-miR-6089 | hsa_circ_0087305 |
| hsa-miR-6089 | hsa_circ_0087855 |
| hsa-miR-6089 | hsa_circ_0008812 |
| hsa-miR-6089 | hsa_circ_0006174 |
| hsa-miR-6089 | hsa_circ_0087861 |
| hsa-miR-6089 | hsa_circ_0087862 |
| hsa-miR-6089 | hsa_circ_0001947 |
| hsa-miR-6090 | hsa_circ_0000130 |
| hsa-miR-6090 | hsa_circ_0000131 |
| hsa-miR-6090 | hsa_circ_0015004 |
| hsa-miR-6090 | hsa_circ_0000375 |
| hsa-miR-6090 | hsa_circ_0033144 |
| hsa-miR-6090 | hsa_circ_0002696 |
| hsa-miR-6090 | hsa_circ_0040921 |
| hsa-miR-6090 | hsa_circ_0046430 |
| hsa-miR-6090 | hsa_circ_0000973 |
| hsa-miR-6090 | hsa_circ_0002805 |
| hsa-miR-6090 | hsa_circ_0004705 |
| hsa-miR-6090 | hsa_circ_0007132 |
| hsa-miR-6090 | hsa_circ_0002094 |
| hsa-miR-6090 | hsa_circ_0084615 |
| hsa-miR-6125 | hsa_circ_0007015 |
| hsa-miR-6125 | hsa_circ_0077765 |
| hsa-miR-6125 | hsa_circ_0002094 |
| hsa-miR-6127 | hsa_circ_0006254 |
| hsa-miR-6127 | hsa_circ_0003768 |
| hsa-miR-6127 | hsa_circ_0044195 |
| hsa-miR-6127 | hsa_circ_0087305 |
| hsa-miR-6132 | hsa_circ_0044195 |
| hsa-miR-6165 | hsa_circ_0040921 |
| hsa-miR-6165 | hsa_circ_0065284 |
| hsa-miR-6165 | hsa_circ_0070467 |
| hsa-miR-6499-5p | hsa_circ_0006649 |
| hsa-miR-6499-5p | hsa_circ_0076793 |
| hsa-miR-6499-5p | hsa_circ_0005630 |
| hsa-miR-6500-5p | hsa_circ_0005630 |
| hsa-miR-6500-3p | hsa_circ_0006681 |
| hsa-miR-6501-5p | hsa_circ_0007846 |
| hsa-miR-6501-3p | hsa_circ_0004136 |
| hsa-miR-6501-3p | hsa_circ_0005630 |
| hsa-miR-6503-5p | hsa_circ_0007846 |
| hsa-miR-6503-5p | hsa_circ_0003340 |
| hsa-miR-6505-3p | hsa_circ_0002451 |
| hsa-miR-6506-5p | hsa_circ_0000247 |
| hsa-miR-6506-3p | hsa_circ_0069399 |
| hsa-miR-6510-5p | hsa_circ_0002805 |
| hsa-miR-6510-5p | hsa_circ_0061179 |
| hsa-miR-6510-5p | hsa_circ_0092299 |
| hsa-miR-6510-5p | hsa_circ_0065284 |
| hsa-miR-6510-5p | hsa_circ_0006528 |
| hsa-miR-6510-5p | hsa_circ_0004381 |
| hsa-miR-6510-5p | hsa_circ_0005630 |
| hsa-miR-6510-5p | hsa_circ_0084429 |
| hsa-miR-6510-5p | hsa_circ_0001829 |
| hsa-miR-6510-5p | hsa_circ_0006566 |
| hsa-miR-6510-3p | hsa_circ_0000835 |
| hsa-miR-6510-3p | hsa_circ_0000836 |
| hsa-miR-6511a-5p | hsa_circ_0015004 |
| hsa-miR-6511a-5p | hsa_circ_0029633 |
| hsa-miR-6511a-5p | hsa_circ_0040921 |
| hsa-miR-6511a-5p | hsa_circ_0045890 |
| hsa-miR-6511a-5p | hsa_circ_0048025 |
| hsa-miR-6511a-5p | hsa_circ_0002113 |
| hsa-miR-6511a-5p | hsa_circ_0064136 |
| hsa-miR-6511a-5p | hsa_circ_0087305 |
| hsa-miR-6511a-3p | hsa_circ_0003557 |
| hsa-miR-6511a-3p | hsa_circ_0004692 |
| hsa-miR-6511a-3p | hsa_circ_0065147 |
| hsa-miR-6511a-3p | hsa_circ_0065149 |
| hsa-miR-6511a-3p | hsa_circ_0002569 |
| hsa-miR-6511a-3p | hsa_circ_0070396 |
| hsa-miR-6511a-3p | hsa_circ_0002094 |
| hsa-miR-6512-3p | hsa_circ_0031933 |
| hsa-miR-6513-3p | hsa_circ_0092283 |
| hsa-miR-6514-5p | hsa_circ_0028190 |
| hsa-miR-6514-3p | hsa_circ_0003315 |
| hsa-miR-6514-3p | hsa_circ_0007145 |
| hsa-miR-6515-5p | hsa_circ_0022383 |
| hsa-miR-6715a-3p | hsa_circ_0048025 |
| hsa-miR-6715b-3p | hsa_circ_0048025 |
| hsa-miR-6716-5p | hsa_circ_0000417 |
| hsa-miR-6716-5p | hsa_circ_0005630 |
| hsa-miR-6511b-5p | hsa_circ_0011422 |
| hsa-miR-6511b-5p | hsa_circ_0015004 |
| hsa-miR-6511b-5p | hsa_circ_0029633 |
| hsa-miR-6511b-5p | hsa_circ_0048025 |
| hsa-miR-6511b-5p | hsa_circ_0004003 |
| hsa-miR-6511b-5p | hsa_circ_0049998 |
| hsa-miR-6511b-5p | hsa_circ_0006552 |
| hsa-miR-6511b-5p | hsa_circ_0081343 |
| hsa-miR-6511b-5p | hsa_circ_0084429 |
| hsa-miR-6511b-5p | hsa_circ_0087305 |
| hsa-miR-6511b-3p | hsa_circ_0003557 |
| hsa-miR-6511b-3p | hsa_circ_0008078 |
| hsa-miR-6511b-3p | hsa_circ_0092299 |
| hsa-miR-6511b-3p | hsa_circ_0001258 |
| hsa-miR-6511b-3p | hsa_circ_0004692 |
| hsa-miR-6511b-3p | hsa_circ_0065147 |
| hsa-miR-6511b-3p | hsa_circ_0065149 |
| hsa-miR-6511b-3p | hsa_circ_0002569 |
| hsa-miR-6511b-3p | hsa_circ_0067913 |
| hsa-miR-6511b-3p | hsa_circ_0002094 |
| hsa-miR-6719-3p | hsa_circ_0000497 |
| hsa-miR-6720-5p | hsa_circ_0009349 |
| hsa-miR-6720-5p | hsa_circ_0003379 |
| hsa-miR-6720-5p | hsa_circ_0022723 |
| hsa-miR-6720-5p | hsa_circ_0036763 |
| hsa-miR-6720-5p | hsa_circ_0000650 |
| hsa-miR-6720-5p | hsa_circ_0040823 |
| hsa-miR-6720-5p | hsa_circ_0040921 |
| hsa-miR-6720-5p | hsa_circ_0003854 |
| hsa-miR-6720-5p | hsa_circ_0001329 |
| hsa-miR-6720-5p | hsa_circ_0006884 |
| hsa-miR-6720-5p | hsa_circ_0007132 |
| hsa-miR-6720-5p | hsa_circ_0076742 |
| hsa-miR-6720-5p | hsa_circ_0084789 |
| hsa-miR-6720-3p | hsa_circ_0007132 |
| hsa-miR-6721-5p | hsa_circ_0004470 |
| hsa-miR-6721-5p | hsa_circ_0007132 |
| hsa-miR-6721-5p | hsa_circ_0087855 |
| hsa-miR-6721-5p | hsa_circ_0008812 |
| hsa-miR-6721-5p | hsa_circ_0006174 |
| hsa-miR-6721-5p | hsa_circ_0087861 |
| hsa-miR-6721-5p | hsa_circ_0087862 |
| hsa-miR-6722-3p | hsa_circ_0002402 |
| hsa-miR-6722-3p | hsa_circ_0008057 |
| hsa-miR-6722-3p | hsa_circ_0011571 |
| hsa-miR-6722-3p | hsa_circ_0011572 |
| hsa-miR-6722-3p | hsa_circ_0007015 |
| hsa-miR-6722-3p | hsa_circ_0006608 |
| hsa-miR-6722-3p | hsa_circ_0008102 |
| hsa-miR-6722-3p | hsa_circ_0022383 |
| hsa-miR-6722-3p | hsa_circ_0000417 |
| hsa-miR-6722-3p | hsa_circ_0002289 |
| hsa-miR-6722-3p | hsa_circ_0007146 |
| hsa-miR-6722-3p | hsa_circ_0046430 |
| hsa-miR-6722-3p | hsa_circ_0047347 |
| hsa-miR-6722-3p | hsa_circ_0048025 |
| hsa-miR-6722-3p | hsa_circ_0049888 |
| hsa-miR-6722-3p | hsa_circ_0002805 |
| hsa-miR-6722-3p | hsa_circ_0061179 |
| hsa-miR-6722-3p | hsa_circ_0092299 |
| hsa-miR-6722-3p | hsa_circ_0064136 |
| hsa-miR-6722-3p | hsa_circ_0004705 |
| hsa-miR-6722-3p | hsa_circ_0003602 |
| hsa-miR-6722-3p | hsa_circ_0070467 |
| hsa-miR-6722-3p | hsa_circ_0071869 |
| hsa-miR-6722-3p | hsa_circ_0007132 |
| hsa-miR-6722-3p | hsa_circ_0003738 |
| hsa-miR-6722-3p | hsa_circ_0004381 |
| hsa-miR-6722-3p | hsa_circ_0081343 |
| hsa-miR-6722-3p | hsa_circ_0002094 |
| hsa-miR-6722-3p | hsa_circ_0005630 |
| hsa-miR-6722-3p | hsa_circ_0001806 |
| hsa-miR-6722-3p | hsa_circ_0084789 |
| hsa-miR-6722-3p | hsa_circ_0087288 |
| hsa-miR-6722-3p | hsa_circ_0087305 |
| hsa-miR-6722-3p | hsa_circ_0001866 |
| hsa-miR-6722-3p | hsa_circ_0087855 |
| hsa-miR-6722-3p | hsa_circ_0008812 |
| hsa-miR-6722-3p | hsa_circ_0006174 |
| hsa-miR-6722-3p | hsa_circ_0087861 |
| hsa-miR-6722-3p | hsa_circ_0087862 |
| hsa-miR-6723-5p | hsa_circ_0017289 |
| hsa-miR-6723-5p | hsa_circ_0005090 |
| hsa-miR-6723-5p | hsa_circ_0007132 |
| hsa-miR-6724-5p | hsa_circ_0009349 |
| hsa-miR-6724-5p | hsa_circ_0002402 |
| hsa-miR-6724-5p | hsa_circ_0007437 |
| hsa-miR-6724-5p | hsa_circ_0007693 |
| hsa-miR-6724-5p | hsa_circ_0012151 |
| hsa-miR-6724-5p | hsa_circ_0007015 |
| hsa-miR-6724-5p | hsa_circ_0017446 |
| hsa-miR-6724-5p | hsa_circ_0000206 |
| hsa-miR-6724-5p | hsa_circ_0007778 |
| hsa-miR-6724-5p | hsa_circ_0018909 |
| hsa-miR-6724-5p | hsa_circ_0003168 |
| hsa-miR-6724-5p | hsa_circ_0003379 |
| hsa-miR-6724-5p | hsa_circ_0003557 |
| hsa-miR-6724-5p | hsa_circ_0000375 |
| hsa-miR-6724-5p | hsa_circ_0000417 |
| hsa-miR-6724-5p | hsa_circ_0029405 |
| hsa-miR-6724-5p | hsa_circ_0007146 |
| hsa-miR-6724-5p | hsa_circ_0006735 |
| hsa-miR-6724-5p | hsa_circ_0000682 |
| hsa-miR-6724-5p | hsa_circ_0000690 |
| hsa-miR-6724-5p | hsa_circ_0005733 |
| hsa-miR-6724-5p | hsa_circ_0040039 |
| hsa-miR-6724-5p | hsa_circ_0040823 |
| hsa-miR-6724-5p | hsa_circ_0092337 |
| hsa-miR-6724-5p | hsa_circ_0043691 |
| hsa-miR-6724-5p | hsa_circ_0045890 |
| hsa-miR-6724-5p | hsa_circ_0046430 |
| hsa-miR-6724-5p | hsa_circ_0048025 |
| hsa-miR-6724-5p | hsa_circ_0002926 |
| hsa-miR-6724-5p | hsa_circ_0049888 |
| hsa-miR-6724-5p | hsa_circ_0000909 |
| hsa-miR-6724-5p | hsa_circ_0050119 |
| hsa-miR-6724-5p | hsa_circ_0052131 |
| hsa-miR-6724-5p | hsa_circ_0092297 |
| hsa-miR-6724-5p | hsa_circ_0003854 |
| hsa-miR-6724-5p | hsa_circ_0004218 |
| hsa-miR-6724-5p | hsa_circ_0061052 |
| hsa-miR-6724-5p | hsa_circ_0092299 |
| hsa-miR-6724-5p | hsa_circ_0092283 |
| hsa-miR-6724-5p | hsa_circ_0004470 |
| hsa-miR-6724-5p | hsa_circ_0004692 |
| hsa-miR-6724-5p | hsa_circ_0065147 |
| hsa-miR-6724-5p | hsa_circ_0065149 |
| hsa-miR-6724-5p | hsa_circ_0002569 |
| hsa-miR-6724-5p | hsa_circ_0001394 |
| hsa-miR-6724-5p | hsa_circ_0069399 |
| hsa-miR-6724-5p | hsa_circ_0001400 |
| hsa-miR-6724-5p | hsa_circ_0071311 |
| hsa-miR-6724-5p | hsa_circ_0072857 |
| hsa-miR-6724-5p | hsa_circ_0001573 |
| hsa-miR-6724-5p | hsa_circ_0007132 |
| hsa-miR-6724-5p | hsa_circ_0004381 |
| hsa-miR-6724-5p | hsa_circ_0078522 |
| hsa-miR-6724-5p | hsa_circ_0003340 |
| hsa-miR-6724-5p | hsa_circ_0002755 |
| hsa-miR-6724-5p | hsa_circ_0081343 |
| hsa-miR-6724-5p | hsa_circ_0001756 |
| hsa-miR-6724-5p | hsa_circ_0002094 |
| hsa-miR-6724-5p | hsa_circ_0005630 |
| hsa-miR-6724-5p | hsa_circ_0005273 |
| hsa-miR-6724-5p | hsa_circ_0007521 |
| hsa-miR-6724-5p | hsa_circ_0005982 |
| hsa-miR-6724-5p | hsa_circ_0003221 |
| hsa-miR-6724-5p | hsa_circ_0002483 |
| hsa-miR-6724-5p | hsa_circ_0006646 |
| hsa-miR-6724-5p | hsa_circ_0001829 |
| hsa-miR-6724-5p | hsa_circ_0006566 |
| hsa-miR-6724-5p | hsa_circ_0087288 |
| hsa-miR-6724-5p | hsa_circ_0087305 |
| hsa-miR-6724-5p | hsa_circ_0087855 |
| hsa-miR-6724-5p | hsa_circ_0008812 |
| hsa-miR-6724-5p | hsa_circ_0006174 |
| hsa-miR-6724-5p | hsa_circ_0087861 |
| hsa-miR-6724-5p | hsa_circ_0087862 |
| hsa-miR-6724-5p | hsa_circ_0092125 |
| hsa-miR-6726-5p | hsa_circ_0009349 |
| hsa-miR-6726-5p | hsa_circ_0006649 |
| hsa-miR-6726-5p | hsa_circ_0029405 |
| hsa-miR-6726-5p | hsa_circ_0004137 |
| hsa-miR-6726-5p | hsa_circ_0002564 |
| hsa-miR-6726-5p | hsa_circ_0002289 |
| hsa-miR-6726-5p | hsa_circ_0033144 |
| hsa-miR-6726-5p | hsa_circ_0046430 |
| hsa-miR-6726-5p | hsa_circ_0000973 |
| hsa-miR-6726-5p | hsa_circ_0055904 |
| hsa-miR-6726-5p | hsa_circ_0061936 |
| hsa-miR-6726-5p | hsa_circ_0001394 |
| hsa-miR-6726-5p | hsa_circ_0007132 |
| hsa-miR-6726-5p | hsa_circ_0081343 |
| hsa-miR-6726-5p | hsa_circ_0002094 |
| hsa-miR-6726-5p | hsa_circ_0005630 |
| hsa-miR-6726-5p | hsa_circ_0008934 |
| hsa-miR-6726-5p | hsa_circ_0001910 |
| hsa-miR-6727-5p | hsa_circ_0015004 |
| hsa-miR-6727-5p | hsa_circ_0016863 |
| hsa-miR-6727-5p | hsa_circ_0016867 |
| hsa-miR-6727-5p | hsa_circ_0006608 |
| hsa-miR-6727-5p | hsa_circ_0003168 |
| hsa-miR-6727-5p | hsa_circ_0003557 |
| hsa-miR-6727-5p | hsa_circ_0000375 |
| hsa-miR-6727-5p | hsa_circ_0000417 |
| hsa-miR-6727-5p | hsa_circ_0008916 |
| hsa-miR-6727-5p | hsa_circ_0000651 |
| hsa-miR-6727-5p | hsa_circ_0007146 |
| hsa-miR-6727-5p | hsa_circ_0007846 |
| hsa-miR-6727-5p | hsa_circ_0000682 |
| hsa-miR-6727-5p | hsa_circ_0040823 |
| hsa-miR-6727-5p | hsa_circ_0043278 |
| hsa-miR-6727-5p | hsa_circ_0045890 |
| hsa-miR-6727-5p | hsa_circ_0045905 |
| hsa-miR-6727-5p | hsa_circ_0046292 |
| hsa-miR-6727-5p | hsa_circ_0046430 |
| hsa-miR-6727-5p | hsa_circ_0047347 |
| hsa-miR-6727-5p | hsa_circ_0049998 |
| hsa-miR-6727-5p | hsa_circ_0005571 |
| hsa-miR-6727-5p | hsa_circ_0050119 |
| hsa-miR-6727-5p | hsa_circ_0000973 |
| hsa-miR-6727-5p | hsa_circ_0008261 |
| hsa-miR-6727-5p | hsa_circ_0061052 |
| hsa-miR-6727-5p | hsa_circ_0002805 |
| hsa-miR-6727-5p | hsa_circ_0061179 |
| hsa-miR-6727-5p | hsa_circ_0007609 |
| hsa-miR-6727-5p | hsa_circ_0092299 |
| hsa-miR-6727-5p | hsa_circ_0004705 |
| hsa-miR-6727-5p | hsa_circ_0001394 |
| hsa-miR-6727-5p | hsa_circ_0070467 |
| hsa-miR-6727-5p | hsa_circ_0071311 |
| hsa-miR-6727-5p | hsa_circ_0002490 |
| hsa-miR-6727-5p | hsa_circ_0007132 |
| hsa-miR-6727-5p | hsa_circ_0004381 |
| hsa-miR-6727-5p | hsa_circ_0003340 |
| hsa-miR-6727-5p | hsa_circ_0009092 |
| hsa-miR-6727-5p | hsa_circ_0001708 |
| hsa-miR-6727-5p | hsa_circ_0002755 |
| hsa-miR-6727-5p | hsa_circ_0081343 |
| hsa-miR-6727-5p | hsa_circ_0002094 |
| hsa-miR-6727-5p | hsa_circ_0084429 |
| hsa-miR-6727-5p | hsa_circ_0084789 |
| hsa-miR-6727-5p | hsa_circ_0087305 |
| hsa-miR-6727-5p | hsa_circ_0087855 |
| hsa-miR-6727-5p | hsa_circ_0008812 |
| hsa-miR-6727-5p | hsa_circ_0006174 |
| hsa-miR-6727-5p | hsa_circ_0087861 |
| hsa-miR-6727-5p | hsa_circ_0087862 |
| hsa-miR-6727-5p | hsa_circ_0001947 |
| hsa-miR-6727-3p | hsa_circ_0033144 |
| hsa-miR-6727-3p | hsa_circ_0049998 |
| hsa-miR-6727-3p | hsa_circ_0007132 |
| hsa-miR-6728-5p | hsa_circ_0000417 |
| hsa-miR-6728-5p | hsa_circ_0031933 |
| hsa-miR-6728-5p | hsa_circ_0007846 |
| hsa-miR-6728-5p | hsa_circ_0007137 |
| hsa-miR-6728-3p | hsa_circ_0036763 |
| hsa-miR-6728-3p | hsa_circ_0000650 |
| hsa-miR-6728-3p | hsa_circ_0048025 |
| hsa-miR-6728-3p | hsa_circ_0000996 |
| hsa-miR-6728-3p | hsa_circ_0061936 |
| hsa-miR-6728-3p | hsa_circ_0092299 |
| hsa-miR-6728-3p | hsa_circ_0002094 |
| hsa-miR-6729-5p | hsa_circ_0009581 |
| hsa-miR-6729-5p | hsa_circ_0006837 |
| hsa-miR-6729-5p | hsa_circ_0007015 |
| hsa-miR-6729-5p | hsa_circ_0017248 |
| hsa-miR-6729-5p | hsa_circ_0006649 |
| hsa-miR-6729-5p | hsa_circ_0022723 |
| hsa-miR-6729-5p | hsa_circ_0003557 |
| hsa-miR-6729-5p | hsa_circ_0000417 |
| hsa-miR-6729-5p | hsa_circ_0028190 |
| hsa-miR-6729-5p | hsa_circ_0033144 |
| hsa-miR-6729-5p | hsa_circ_0036763 |
| hsa-miR-6729-5p | hsa_circ_0000650 |
| hsa-miR-6729-5p | hsa_circ_0036768 |
| hsa-miR-6729-5p | hsa_circ_0041050 |
| hsa-miR-6729-5p | hsa_circ_0046430 |
| hsa-miR-6729-5p | hsa_circ_0048025 |
| hsa-miR-6729-5p | hsa_circ_0061052 |
| hsa-miR-6729-5p | hsa_circ_0004692 |
| hsa-miR-6729-5p | hsa_circ_0065147 |
| hsa-miR-6729-5p | hsa_circ_0065149 |
| hsa-miR-6729-5p | hsa_circ_0002569 |
| hsa-miR-6729-5p | hsa_circ_0070467 |
| hsa-miR-6729-5p | hsa_circ_0072437 |
| hsa-miR-6729-5p | hsa_circ_0003528 |
| hsa-miR-6729-5p | hsa_circ_0001573 |
| hsa-miR-6729-5p | hsa_circ_0001577 |
| hsa-miR-6729-5p | hsa_circ_0007132 |
| hsa-miR-6729-5p | hsa_circ_0004381 |
| hsa-miR-6729-5p | hsa_circ_0001748 |
| hsa-miR-6729-5p | hsa_circ_0001756 |
| hsa-miR-6729-5p | hsa_circ_0084429 |
| hsa-miR-6729-3p | hsa_circ_0020594 |
| hsa-miR-6729-3p | hsa_circ_0004692 |
| hsa-miR-6729-3p | hsa_circ_0007132 |
| hsa-miR-6730-5p | hsa_circ_0074264 |
| hsa-miR-6730-5p | hsa_circ_0005251 |
| hsa-miR-6731-5p | hsa_circ_0017438 |
| hsa-miR-6731-5p | hsa_circ_0004277 |
| hsa-miR-6731-5p | hsa_circ_0017446 |
| hsa-miR-6731-5p | hsa_circ_0004470 |
| hsa-miR-6731-5p | hsa_circ_0001472 |
| hsa-miR-6731-3p | hsa_circ_0000395 |
| hsa-miR-6731-3p | hsa_circ_0061936 |
| hsa-miR-6732-5p | hsa_circ_0015004 |
| hsa-miR-6732-5p | hsa_circ_0000417 |
| hsa-miR-6732-5p | hsa_circ_0048025 |
| hsa-miR-6732-5p | hsa_circ_0007132 |
| hsa-miR-6732-3p | hsa_circ_0017438 |
| hsa-miR-6732-3p | hsa_circ_0054086 |
| hsa-miR-6732-3p | hsa_circ_0007132 |
| hsa-miR-6732-3p | hsa_circ_0007145 |
| hsa-miR-6732-3p | hsa_circ_0005630 |
| hsa-miR-6734-5p | hsa_circ_0000417 |
| hsa-miR-6734-5p | hsa_circ_0031933 |
| hsa-miR-6734-5p | hsa_circ_0069748 |
| hsa-miR-6734-5p | hsa_circ_0001472 |
| hsa-miR-6734-3p | hsa_circ_0046430 |
| hsa-miR-6734-3p | hsa_circ_0054086 |
| hsa-miR-6734-3p | hsa_circ_0002805 |
| hsa-miR-6734-3p | hsa_circ_0061936 |
| hsa-miR-6734-3p | hsa_circ_0004692 |
| hsa-miR-6734-3p | hsa_circ_0005630 |
| hsa-miR-6735-5p | hsa_circ_0022392 |
| hsa-miR-6735-5p | hsa_circ_0029633 |
| hsa-miR-6735-5p | hsa_circ_0045905 |
| hsa-miR-6735-5p | hsa_circ_0046430 |
| hsa-miR-6735-5p | hsa_circ_0004003 |
| hsa-miR-6735-5p | hsa_circ_0004891 |
| hsa-miR-6735-5p | hsa_circ_0006877 |
| hsa-miR-6735-5p | hsa_circ_0002113 |
| hsa-miR-6735-5p | hsa_circ_0004692 |
| hsa-miR-6735-5p | hsa_circ_0001472 |
| hsa-miR-6735-5p | hsa_circ_0084615 |
| hsa-miR-6735-3p | hsa_circ_0011571 |
| hsa-miR-6735-3p | hsa_circ_0011572 |
| hsa-miR-6735-3p | hsa_circ_0000690 |
| hsa-miR-6735-3p | hsa_circ_0054086 |
| hsa-miR-6735-3p | hsa_circ_0006693 |
| hsa-miR-6735-3p | hsa_circ_0001417 |
| hsa-miR-6735-3p | hsa_circ_0007132 |
| hsa-miR-6735-3p | hsa_circ_0001748 |
| hsa-miR-6735-3p | hsa_circ_0005630 |
| hsa-miR-6736-5p | hsa_circ_0002402 |
| hsa-miR-6736-5p | hsa_circ_0073379 |
| hsa-miR-6736-5p | hsa_circ_0005540 |
| hsa-miR-6736-5p | hsa_circ_0007132 |
| hsa-miR-6736-5p | hsa_circ_0005630 |
| hsa-miR-6736-5p | hsa_circ_0084615 |
| hsa-miR-6737-5p | hsa_circ_0003557 |
| hsa-miR-6737-5p | hsa_circ_0000417 |
| hsa-miR-6737-5p | hsa_circ_0033144 |
| hsa-miR-6737-5p | hsa_circ_0003838 |
| hsa-miR-6737-5p | hsa_circ_0001955 |
| hsa-miR-6737-5p | hsa_circ_0045890 |
| hsa-miR-6737-5p | hsa_circ_0046430 |
| hsa-miR-6737-5p | hsa_circ_0050119 |
| hsa-miR-6737-5p | hsa_circ_0000918 |
| hsa-miR-6737-5p | hsa_circ_0002805 |
| hsa-miR-6737-5p | hsa_circ_0061179 |
| hsa-miR-6737-5p | hsa_circ_0061936 |
| hsa-miR-6737-5p | hsa_circ_0007132 |
| hsa-miR-6737-5p | hsa_circ_0081423 |
| hsa-miR-6738-5p | hsa_circ_0008102 |
| hsa-miR-6738-5p | hsa_circ_0047347 |
| hsa-miR-6738-5p | hsa_circ_0004470 |
| hsa-miR-6738-5p | hsa_circ_0064136 |
| hsa-miR-6738-5p | hsa_circ_0079449 |
| hsa-miR-6738-5p | hsa_circ_0001756 |
| hsa-miR-6738-3p | hsa_circ_0007001 |
| hsa-miR-6740-5p | hsa_circ_0000375 |
| hsa-miR-6740-5p | hsa_circ_0084429 |
| hsa-miR-6740-3p | hsa_circ_0004692 |
| hsa-miR-6741-5p | hsa_circ_0006608 |
| hsa-miR-6741-5p | hsa_circ_0018909 |
| hsa-miR-6741-5p | hsa_circ_0006649 |
| hsa-miR-6741-5p | hsa_circ_0022505 |
| hsa-miR-6741-5p | hsa_circ_0035381 |
| hsa-miR-6741-5p | hsa_circ_0040921 |
| hsa-miR-6741-5p | hsa_circ_0046430 |
| hsa-miR-6741-5p | hsa_circ_0048025 |
| hsa-miR-6741-5p | hsa_circ_0004003 |
| hsa-miR-6741-5p | hsa_circ_0049657 |
| hsa-miR-6741-5p | hsa_circ_0003854 |
| hsa-miR-6741-5p | hsa_circ_0061052 |
| hsa-miR-6741-5p | hsa_circ_0092283 |
| hsa-miR-6741-5p | hsa_circ_0001400 |
| hsa-miR-6741-5p | hsa_circ_0078522 |
| hsa-miR-6741-5p | hsa_circ_0002094 |
| hsa-miR-6741-5p | hsa_circ_0007521 |
| hsa-miR-6741-5p | hsa_circ_0005982 |
| hsa-miR-6741-5p | hsa_circ_0003221 |
| hsa-miR-6741-5p | hsa_circ_0002483 |
| hsa-miR-6741-5p | hsa_circ_0006646 |
| hsa-miR-6741-5p | hsa_circ_0087305 |
| hsa-miR-6741-3p | hsa_circ_0000996 |
| hsa-miR-6741-3p | hsa_circ_0002805 |
| hsa-miR-6742-5p | hsa_circ_0008102 |
| hsa-miR-6742-5p | hsa_circ_0006735 |
| hsa-miR-6742-5p | hsa_circ_0004003 |
| hsa-miR-6742-5p | hsa_circ_0004891 |
| hsa-miR-6742-5p | hsa_circ_0004705 |
| hsa-miR-6742-3p | hsa_circ_0004519 |
| hsa-miR-6742-3p | hsa_circ_0007132 |
| hsa-miR-6743-5p | hsa_circ_0002402 |
| hsa-miR-6743-5p | hsa_circ_0002570 |
| hsa-miR-6743-5p | hsa_circ_0014132 |
| hsa-miR-6743-5p | hsa_circ_0003379 |
| hsa-miR-6743-5p | hsa_circ_0007372 |
| hsa-miR-6743-5p | hsa_circ_0000417 |
| hsa-miR-6743-5p | hsa_circ_0028190 |
| hsa-miR-6743-5p | hsa_circ_0033144 |
| hsa-miR-6743-5p | hsa_circ_0050119 |
| hsa-miR-6743-5p | hsa_circ_0061052 |
| hsa-miR-6743-5p | hsa_circ_0002805 |
| hsa-miR-6743-5p | hsa_circ_0061179 |
| hsa-miR-6743-5p | hsa_circ_0001394 |
| hsa-miR-6743-5p | hsa_circ_0003278 |
| hsa-miR-6743-5p | hsa_circ_0007132 |
| hsa-miR-6743-5p | hsa_circ_0002094 |
| hsa-miR-6743-5p | hsa_circ_0005630 |
| hsa-miR-6743-5p | hsa_circ_0087305 |
| hsa-miR-6743-5p | hsa_circ_0087855 |
| hsa-miR-6743-5p | hsa_circ_0008812 |
| hsa-miR-6743-5p | hsa_circ_0006174 |
| hsa-miR-6743-5p | hsa_circ_0087861 |
| hsa-miR-6743-5p | hsa_circ_0087862 |
| hsa-miR-6743-3p | hsa_circ_0022723 |
| hsa-miR-6743-3p | hsa_circ_0092299 |
| hsa-miR-6743-3p | hsa_circ_0004692 |
| hsa-miR-6743-3p | hsa_circ_0084789 |
| hsa-miR-6743-3p | hsa_circ_0092125 |
| hsa-miR-6744-3p | hsa_circ_0003168 |
| hsa-miR-6744-3p | hsa_circ_0000835 |
| hsa-miR-6744-3p | hsa_circ_0000836 |
| hsa-miR-6744-3p | hsa_circ_0002805 |
| hsa-miR-6744-3p | hsa_circ_0007132 |
| hsa-miR-6744-3p | hsa_circ_0076793 |
| hsa-miR-6744-3p | hsa_circ_0002094 |
| hsa-miR-6744-3p | hsa_circ_0084552 |
| hsa-miR-6746-5p | hsa_circ_0006608 |
| hsa-miR-6746-5p | hsa_circ_0003168 |
| hsa-miR-6746-5p | hsa_circ_0000417 |
| hsa-miR-6746-5p | hsa_circ_0031933 |
| hsa-miR-6746-5p | hsa_circ_0007846 |
| hsa-miR-6746-5p | hsa_circ_0046430 |
| hsa-miR-6746-5p | hsa_circ_0049657 |
| hsa-miR-6746-5p | hsa_circ_0050119 |
| hsa-miR-6746-5p | hsa_circ_0000936 |
| hsa-miR-6746-5p | hsa_circ_0052131 |
| hsa-miR-6746-5p | hsa_circ_0000973 |
| hsa-miR-6746-5p | hsa_circ_0004218 |
| hsa-miR-6746-5p | hsa_circ_0002805 |
| hsa-miR-6746-5p | hsa_circ_0092299 |
| hsa-miR-6746-5p | hsa_circ_0003602 |
| hsa-miR-6746-5p | hsa_circ_0007132 |
| hsa-miR-6746-5p | hsa_circ_0004381 |
| hsa-miR-6746-5p | hsa_circ_0005519 |
| hsa-miR-6746-3p | hsa_circ_0020594 |
| hsa-miR-6746-3p | hsa_circ_0033144 |
| hsa-miR-6746-3p | hsa_circ_0049998 |
| hsa-miR-6746-3p | hsa_circ_0004699 |
| hsa-miR-6746-3p | hsa_circ_0003602 |
| hsa-miR-6746-3p | hsa_circ_0007132 |
| hsa-miR-6746-3p | hsa_circ_0001756 |
| hsa-miR-6746-3p | hsa_circ_0008113 |
| hsa-miR-6747-5p | hsa_circ_0045890 |
| hsa-miR-6747-5p | hsa_circ_0001472 |
| hsa-miR-6747-5p | hsa_circ_0005630 |
| hsa-miR-6747-5p | hsa_circ_0001824 |
| hsa-miR-6747-5p | hsa_circ_0008934 |
| hsa-miR-6747-5p | hsa_circ_0087288 |
| hsa-miR-6748-5p | hsa_circ_0022392 |
| hsa-miR-6748-5p | hsa_circ_0002077 |
| hsa-miR-6748-3p | hsa_circ_0009732 |
| hsa-miR-6748-3p | hsa_circ_0020594 |
| hsa-miR-6748-3p | hsa_circ_0000395 |
| hsa-miR-6749-5p | hsa_circ_0002402 |
| hsa-miR-6749-5p | hsa_circ_0018909 |
| hsa-miR-6749-5p | hsa_circ_0022383 |
| hsa-miR-6749-5p | hsa_circ_0022723 |
| hsa-miR-6749-5p | hsa_circ_0000417 |
| hsa-miR-6749-5p | hsa_circ_0004008 |
| hsa-miR-6749-5p | hsa_circ_0033144 |
| hsa-miR-6749-5p | hsa_circ_0034189 |
| hsa-miR-6749-5p | hsa_circ_0007846 |
| hsa-miR-6749-5p | hsa_circ_0040823 |
| hsa-miR-6749-5p | hsa_circ_0045890 |
| hsa-miR-6749-5p | hsa_circ_0000854 |
| hsa-miR-6749-5p | hsa_circ_0048025 |
| hsa-miR-6749-5p | hsa_circ_0004003 |
| hsa-miR-6749-5p | hsa_circ_0000909 |
| hsa-miR-6749-5p | hsa_circ_0050119 |
| hsa-miR-6749-5p | hsa_circ_0092297 |
| hsa-miR-6749-5p | hsa_circ_0002805 |
| hsa-miR-6749-5p | hsa_circ_0061179 |
| hsa-miR-6749-5p | hsa_circ_0007609 |
| hsa-miR-6749-5p | hsa_circ_0092299 |
| hsa-miR-6749-5p | hsa_circ_0003602 |
| hsa-miR-6749-5p | hsa_circ_0092277 |
| hsa-miR-6749-5p | hsa_circ_0001394 |
| hsa-miR-6749-5p | hsa_circ_0006693 |
| hsa-miR-6749-5p | hsa_circ_0001417 |
| hsa-miR-6749-5p | hsa_circ_0071311 |
| hsa-miR-6749-5p | hsa_circ_0072437 |
| hsa-miR-6749-5p | hsa_circ_0072857 |
| hsa-miR-6749-5p | hsa_circ_0007132 |
| hsa-miR-6749-5p | hsa_circ_0001748 |
| hsa-miR-6749-5p | hsa_circ_0002094 |
| hsa-miR-6749-5p | hsa_circ_0005630 |
| hsa-miR-6749-5p | hsa_circ_0084429 |
| hsa-miR-6749-5p | hsa_circ_0008934 |
| hsa-miR-6749-5p | hsa_circ_0005273 |
| hsa-miR-6749-5p | hsa_circ_0001829 |
| hsa-miR-6749-5p | hsa_circ_0087288 |
| hsa-miR-6749-5p | hsa_circ_0087305 |
| hsa-miR-6749-3p | hsa_circ_0020594 |
| hsa-miR-6749-3p | hsa_circ_0000417 |
| hsa-miR-6749-3p | hsa_circ_0040921 |
| hsa-miR-6749-3p | hsa_circ_0092337 |
| hsa-miR-6749-3p | hsa_circ_0007132 |
| hsa-miR-6749-3p | hsa_circ_0007145 |
| hsa-miR-6749-3p | hsa_circ_0002094 |
| hsa-miR-6749-3p | hsa_circ_0005630 |
| hsa-miR-6749-3p | hsa_circ_0084429 |
| hsa-miR-6749-3p | hsa_circ_0001829 |
| hsa-miR-6750-5p | hsa_circ_0036763 |
| hsa-miR-6750-5p | hsa_circ_0000650 |
| hsa-miR-6750-5p | hsa_circ_0036768 |
| hsa-miR-6750-5p | hsa_circ_0007846 |
| hsa-miR-6750-5p | hsa_circ_0045890 |
| hsa-miR-6750-5p | hsa_circ_0048025 |
| hsa-miR-6750-5p | hsa_circ_0002805 |
| hsa-miR-6750-5p | hsa_circ_0061179 |
| hsa-miR-6750-5p | hsa_circ_0072857 |
| hsa-miR-6750-5p | hsa_circ_0002037 |
| hsa-miR-6751-5p | hsa_circ_0009581 |
| hsa-miR-6751-5p | hsa_circ_0006837 |
| hsa-miR-6751-5p | hsa_circ_0011422 |
| hsa-miR-6751-5p | hsa_circ_0014132 |
| hsa-miR-6751-5p | hsa_circ_0017289 |
| hsa-miR-6751-5p | hsa_circ_0005090 |
| hsa-miR-6751-5p | hsa_circ_0017438 |
| hsa-miR-6751-5p | hsa_circ_0004277 |
| hsa-miR-6751-5p | hsa_circ_0017446 |
| hsa-miR-6751-5p | hsa_circ_0000247 |
| hsa-miR-6751-5p | hsa_circ_0020594 |
| hsa-miR-6751-5p | hsa_circ_0006254 |
| hsa-miR-6751-5p | hsa_circ_0003768 |
| hsa-miR-6751-5p | hsa_circ_0003557 |
| hsa-miR-6751-5p | hsa_circ_0000375 |
| hsa-miR-6751-5p | hsa_circ_0000417 |
| hsa-miR-6751-5p | hsa_circ_0031933 |
| hsa-miR-6751-5p | hsa_circ_0046430 |
| hsa-miR-6751-5p | hsa_circ_0002926 |
| hsa-miR-6751-5p | hsa_circ_0000936 |
| hsa-miR-6751-5p | hsa_circ_0055904 |
| hsa-miR-6751-5p | hsa_circ_0092297 |
| hsa-miR-6751-5p | hsa_circ_0004218 |
| hsa-miR-6751-5p | hsa_circ_0002805 |
| hsa-miR-6751-5p | hsa_circ_0061179 |
| hsa-miR-6751-5p | hsa_circ_0004705 |
| hsa-miR-6751-5p | hsa_circ_0001394 |
| hsa-miR-6751-5p | hsa_circ_0006681 |
| hsa-miR-6751-5p | hsa_circ_0073379 |
| hsa-miR-6751-5p | hsa_circ_0005540 |
| hsa-miR-6751-5p | hsa_circ_0007132 |
| hsa-miR-6751-5p | hsa_circ_0008536 |
| hsa-miR-6751-5p | hsa_circ_0077765 |
| hsa-miR-6751-5p | hsa_circ_0005630 |
| hsa-miR-6751-5p | hsa_circ_0087305 |
| hsa-miR-6751-3p | hsa_circ_0004692 |
| hsa-miR-6752-5p | hsa_circ_0009581 |
| hsa-miR-6752-5p | hsa_circ_0006837 |
| hsa-miR-6752-5p | hsa_circ_0007015 |
| hsa-miR-6752-5p | hsa_circ_0008102 |
| hsa-miR-6752-5p | hsa_circ_0003379 |
| hsa-miR-6752-5p | hsa_circ_0022383 |
| hsa-miR-6752-5p | hsa_circ_0000339 |
| hsa-miR-6752-5p | hsa_circ_0003908 |
| hsa-miR-6752-5p | hsa_circ_0002884 |
| hsa-miR-6752-5p | hsa_circ_0000375 |
| hsa-miR-6752-5p | hsa_circ_0003855 |
| hsa-miR-6752-5p | hsa_circ_0000417 |
| hsa-miR-6752-5p | hsa_circ_0029633 |
| hsa-miR-6752-5p | hsa_circ_0002289 |
| hsa-miR-6752-5p | hsa_circ_0033144 |
| hsa-miR-6752-5p | hsa_circ_0001955 |
| hsa-miR-6752-5p | hsa_circ_0007846 |
| hsa-miR-6752-5p | hsa_circ_0045890 |
| hsa-miR-6752-5p | hsa_circ_0046430 |
| hsa-miR-6752-5p | hsa_circ_0047347 |
| hsa-miR-6752-5p | hsa_circ_0048025 |
| hsa-miR-6752-5p | hsa_circ_0006877 |
| hsa-miR-6752-5p | hsa_circ_0049998 |
| hsa-miR-6752-5p | hsa_circ_0000918 |
| hsa-miR-6752-5p | hsa_circ_0000936 |
| hsa-miR-6752-5p | hsa_circ_0000973 |
| hsa-miR-6752-5p | hsa_circ_0058522 |
| hsa-miR-6752-5p | hsa_circ_0003854 |
| hsa-miR-6752-5p | hsa_circ_0004218 |
| hsa-miR-6752-5p | hsa_circ_0002805 |
| hsa-miR-6752-5p | hsa_circ_0061936 |
| hsa-miR-6752-5p | hsa_circ_0092299 |
| hsa-miR-6752-5p | hsa_circ_0092277 |
| hsa-miR-6752-5p | hsa_circ_0070467 |
| hsa-miR-6752-5p | hsa_circ_0071311 |
| hsa-miR-6752-5p | hsa_circ_0001549 |
| hsa-miR-6752-5p | hsa_circ_0001573 |
| hsa-miR-6752-5p | hsa_circ_0007132 |
| hsa-miR-6752-5p | hsa_circ_0004381 |
| hsa-miR-6752-5p | hsa_circ_0003340 |
| hsa-miR-6752-5p | hsa_circ_0002094 |
| hsa-miR-6752-5p | hsa_circ_0005630 |
| hsa-miR-6752-5p | hsa_circ_0001829 |
| hsa-miR-6752-5p | hsa_circ_0006566 |
| hsa-miR-6752-5p | hsa_circ_0087288 |
| hsa-miR-6752-5p | hsa_circ_0087305 |
| hsa-miR-6752-5p | hsa_circ_0001866 |
| hsa-miR-6752-5p | hsa_circ_0087855 |
| hsa-miR-6752-5p | hsa_circ_0008812 |
| hsa-miR-6752-5p | hsa_circ_0006174 |
| hsa-miR-6752-5p | hsa_circ_0087861 |
| hsa-miR-6752-5p | hsa_circ_0087862 |
| hsa-miR-6752-3p | hsa_circ_0020594 |
| hsa-miR-6752-3p | hsa_circ_0007132 |
| hsa-miR-6753-5p | hsa_circ_0015004 |
| hsa-miR-6753-5p | hsa_circ_0003557 |
| hsa-miR-6753-5p | hsa_circ_0000650 |
| hsa-miR-6753-5p | hsa_circ_0036768 |
| hsa-miR-6753-5p | hsa_circ_0048025 |
| hsa-miR-6753-5p | hsa_circ_0092297 |
| hsa-miR-6753-5p | hsa_circ_0092283 |
| hsa-miR-6753-5p | hsa_circ_0004470 |
| hsa-miR-6753-5p | hsa_circ_0004692 |
| hsa-miR-6753-5p | hsa_circ_0065147 |
| hsa-miR-6753-5p | hsa_circ_0006884 |
| hsa-miR-6753-5p | hsa_circ_0007137 |
| hsa-miR-6753-5p | hsa_circ_0002490 |
| hsa-miR-6753-5p | hsa_circ_0005630 |
| hsa-miR-6753-3p | hsa_circ_0000395 |
| hsa-miR-6753-3p | hsa_circ_0061936 |
| hsa-miR-6753-3p | hsa_circ_0072437 |
| hsa-miR-6754-5p | hsa_circ_0007015 |
| hsa-miR-6754-5p | hsa_circ_0000417 |
| hsa-miR-6754-5p | hsa_circ_0028190 |
| hsa-miR-6754-5p | hsa_circ_0002926 |
| hsa-miR-6754-5p | hsa_circ_0001178 |
| hsa-miR-6754-5p | hsa_circ_0064136 |
| hsa-miR-6754-5p | hsa_circ_0004705 |
| hsa-miR-6754-5p | hsa_circ_0001400 |
| hsa-miR-6754-5p | hsa_circ_0071311 |
| hsa-miR-6754-5p | hsa_circ_0001472 |
| hsa-miR-6754-5p | hsa_circ_0001549 |
| hsa-miR-6754-5p | hsa_circ_0003340 |
| hsa-miR-6754-5p | hsa_circ_0087288 |
| hsa-miR-6754-3p | hsa_circ_0003557 |
| hsa-miR-6754-3p | hsa_circ_0007372 |
| hsa-miR-6754-3p | hsa_circ_0047700 |
| hsa-miR-6754-3p | hsa_circ_0092283 |
| hsa-miR-6754-3p | hsa_circ_0006022 |
| hsa-miR-6756-5p | hsa_circ_0005782 |
| hsa-miR-6756-5p | hsa_circ_0002402 |
| hsa-miR-6756-5p | hsa_circ_0014132 |
| hsa-miR-6756-5p | hsa_circ_0007015 |
| hsa-miR-6756-5p | hsa_circ_0017248 |
| hsa-miR-6756-5p | hsa_circ_0000213 |
| hsa-miR-6756-5p | hsa_circ_0018909 |
| hsa-miR-6756-5p | hsa_circ_0008102 |
| hsa-miR-6756-5p | hsa_circ_0003168 |
| hsa-miR-6756-5p | hsa_circ_0006254 |
| hsa-miR-6756-5p | hsa_circ_0003768 |
| hsa-miR-6756-5p | hsa_circ_0022383 |
| hsa-miR-6756-5p | hsa_circ_0007372 |
| hsa-miR-6756-5p | hsa_circ_0000375 |
| hsa-miR-6756-5p | hsa_circ_0003855 |
| hsa-miR-6756-5p | hsa_circ_0000395 |
| hsa-miR-6756-5p | hsa_circ_0000417 |
| hsa-miR-6756-5p | hsa_circ_0029405 |
| hsa-miR-6756-5p | hsa_circ_0000497 |
| hsa-miR-6756-5p | hsa_circ_0031933 |
| hsa-miR-6756-5p | hsa_circ_0033144 |
| hsa-miR-6756-5p | hsa_circ_0036763 |
| hsa-miR-6756-5p | hsa_circ_0000650 |
| hsa-miR-6756-5p | hsa_circ_0036768 |
| hsa-miR-6756-5p | hsa_circ_0007846 |
| hsa-miR-6756-5p | hsa_circ_0006735 |
| hsa-miR-6756-5p | hsa_circ_0000682 |
| hsa-miR-6756-5p | hsa_circ_0044195 |
| hsa-miR-6756-5p | hsa_circ_0045890 |
| hsa-miR-6756-5p | hsa_circ_0000854 |
| hsa-miR-6756-5p | hsa_circ_0048025 |
| hsa-miR-6756-5p | hsa_circ_0004003 |
| hsa-miR-6756-5p | hsa_circ_0006877 |
| hsa-miR-6756-5p | hsa_circ_0000918 |
| hsa-miR-6756-5p | hsa_circ_0052131 |
| hsa-miR-6756-5p | hsa_circ_0000973 |
| hsa-miR-6756-5p | hsa_circ_0000996 |
| hsa-miR-6756-5p | hsa_circ_0058522 |
| hsa-miR-6756-5p | hsa_circ_0003854 |
| hsa-miR-6756-5p | hsa_circ_0004218 |
| hsa-miR-6756-5p | hsa_circ_0061936 |
| hsa-miR-6756-5p | hsa_circ_0092299 |
| hsa-miR-6756-5p | hsa_circ_0004470 |
| hsa-miR-6756-5p | hsa_circ_0001258 |
| hsa-miR-6756-5p | hsa_circ_0064136 |
| hsa-miR-6756-5p | hsa_circ_0004705 |
| hsa-miR-6756-5p | hsa_circ_0001394 |
| hsa-miR-6756-5p | hsa_circ_0006681 |
| hsa-miR-6756-5p | hsa_circ_0069748 |
| hsa-miR-6756-5p | hsa_circ_0070467 |
| hsa-miR-6756-5p | hsa_circ_0071311 |
| hsa-miR-6756-5p | hsa_circ_0001472 |
| hsa-miR-6756-5p | hsa_circ_0074264 |
| hsa-miR-6756-5p | hsa_circ_0074816 |
| hsa-miR-6756-5p | hsa_circ_0074817 |
| hsa-miR-6756-5p | hsa_circ_0001549 |
| hsa-miR-6756-5p | hsa_circ_0007132 |
| hsa-miR-6756-5p | hsa_circ_0075748 |
| hsa-miR-6756-5p | hsa_circ_0001658 |
| hsa-miR-6756-5p | hsa_circ_0004381 |
| hsa-miR-6756-5p | hsa_circ_0009092 |
| hsa-miR-6756-5p | hsa_circ_0002755 |
| hsa-miR-6756-5p | hsa_circ_0001748 |
| hsa-miR-6756-5p | hsa_circ_0001756 |
| hsa-miR-6756-5p | hsa_circ_0002094 |
| hsa-miR-6756-5p | hsa_circ_0005630 |
| hsa-miR-6756-5p | hsa_circ_0001806 |
| hsa-miR-6756-5p | hsa_circ_0087288 |
| hsa-miR-6756-5p | hsa_circ_0087305 |
| hsa-miR-6756-3p | hsa_circ_0020594 |
| hsa-miR-6756-3p | hsa_circ_0000650 |
| hsa-miR-6756-3p | hsa_circ_0036768 |
| hsa-miR-6756-3p | hsa_circ_0040921 |
| hsa-miR-6756-3p | hsa_circ_0092299 |
| hsa-miR-6756-3p | hsa_circ_0004692 |
| hsa-miR-6756-3p | hsa_circ_0007132 |
| hsa-miR-6756-3p | hsa_circ_0007145 |
| hsa-miR-6756-3p | hsa_circ_0005630 |
| hsa-miR-6757-5p | hsa_circ_0022392 |
| hsa-miR-6757-5p | hsa_circ_0007846 |
| hsa-miR-6757-5p | hsa_circ_0002696 |
| hsa-miR-6757-5p | hsa_circ_0000854 |
| hsa-miR-6757-5p | hsa_circ_0048025 |
| hsa-miR-6757-5p | hsa_circ_0004705 |
| hsa-miR-6757-5p | hsa_circ_0001400 |
| hsa-miR-6758-5p | hsa_circ_0070467 |
| hsa-miR-6758-3p | hsa_circ_0047347 |
| hsa-miR-6759-5p | hsa_circ_0046430 |
| hsa-miR-6760-5p | hsa_circ_0009581 |
| hsa-miR-6760-5p | hsa_circ_0006837 |
| hsa-miR-6760-5p | hsa_circ_0015966 |
| hsa-miR-6760-5p | hsa_circ_0022383 |
| hsa-miR-6760-3p | hsa_circ_0000395 |
| hsa-miR-6760-3p | hsa_circ_0092299 |
| hsa-miR-6761-3p | hsa_circ_0022723 |
| hsa-miR-6761-3p | hsa_circ_0043522 |
| hsa-miR-6761-3p | hsa_circ_0007132 |
| hsa-miR-6762-5p | hsa_circ_0033144 |
| hsa-miR-6762-5p | hsa_circ_0007146 |
| hsa-miR-6762-5p | hsa_circ_0040039 |
| hsa-miR-6762-5p | hsa_circ_0045890 |
| hsa-miR-6762-5p | hsa_circ_0004003 |
| hsa-miR-6762-5p | hsa_circ_0008261 |
| hsa-miR-6762-5p | hsa_circ_0061052 |
| hsa-miR-6762-5p | hsa_circ_0002805 |
| hsa-miR-6762-5p | hsa_circ_0006884 |
| hsa-miR-6762-5p | hsa_circ_0003278 |
| hsa-miR-6762-5p | hsa_circ_0007132 |
| hsa-miR-6762-5p | hsa_circ_0006566 |
| hsa-miR-6762-5p | hsa_circ_0087305 |
| hsa-miR-6762-5p | hsa_circ_0001910 |
| hsa-miR-6762-3p | hsa_circ_0045890 |
| hsa-miR-6762-3p | hsa_circ_0092299 |
| hsa-miR-6762-3p | hsa_circ_0004692 |
| hsa-miR-6762-3p | hsa_circ_0002755 |
| hsa-miR-6762-3p | hsa_circ_0007521 |
| hsa-miR-6762-3p | hsa_circ_0005982 |
| hsa-miR-6762-3p | hsa_circ_0003221 |
| hsa-miR-6762-3p | hsa_circ_0002483 |
| hsa-miR-6762-3p | hsa_circ_0006646 |
| hsa-miR-6763-5p | hsa_circ_0008102 |
| hsa-miR-6763-5p | hsa_circ_0006254 |
| hsa-miR-6763-5p | hsa_circ_0003768 |
| hsa-miR-6763-5p | hsa_circ_0000497 |
| hsa-miR-6763-5p | hsa_circ_0045890 |
| hsa-miR-6763-5p | hsa_circ_0046430 |
| hsa-miR-6763-5p | hsa_circ_0049998 |
| hsa-miR-6763-5p | hsa_circ_0055904 |
| hsa-miR-6763-5p | hsa_circ_0058514 |
| hsa-miR-6763-5p | hsa_circ_0058520 |
| hsa-miR-6763-5p | hsa_circ_0058522 |
| hsa-miR-6763-5p | hsa_circ_0003854 |
| hsa-miR-6763-5p | hsa_circ_0064136 |
| hsa-miR-6763-5p | hsa_circ_0003738 |
| hsa-miR-6763-5p | hsa_circ_0002037 |
| hsa-miR-6763-5p | hsa_circ_0001756 |
| hsa-miR-6763-5p | hsa_circ_0002094 |
| hsa-miR-6763-3p | hsa_circ_0011571 |
| hsa-miR-6763-3p | hsa_circ_0011572 |
| hsa-miR-6763-3p | hsa_circ_0020594 |
| hsa-miR-6763-3p | hsa_circ_0000417 |
| hsa-miR-6763-3p | hsa_circ_0045890 |
| hsa-miR-6763-3p | hsa_circ_0046430 |
| hsa-miR-6763-3p | hsa_circ_0048025 |
| hsa-miR-6763-3p | hsa_circ_0000996 |
| hsa-miR-6763-3p | hsa_circ_0092283 |
| hsa-miR-6763-3p | hsa_circ_0070396 |
| hsa-miR-6763-3p | hsa_circ_0007132 |
| hsa-miR-6763-3p | hsa_circ_0007145 |
| hsa-miR-6764-5p | hsa_circ_0040823 |
| hsa-miR-6764-5p | hsa_circ_0046292 |
| hsa-miR-6764-5p | hsa_circ_0087305 |
| hsa-miR-6765-5p | hsa_circ_0009581 |
| hsa-miR-6765-5p | hsa_circ_0006837 |
| hsa-miR-6765-5p | hsa_circ_0005782 |
| hsa-miR-6765-5p | hsa_circ_0007015 |
| hsa-miR-6765-5p | hsa_circ_0008788 |
| hsa-miR-6765-5p | hsa_circ_0017248 |
| hsa-miR-6765-5p | hsa_circ_0018909 |
| hsa-miR-6765-5p | hsa_circ_0006649 |
| hsa-miR-6765-5p | hsa_circ_0020005 |
| hsa-miR-6765-5p | hsa_circ_0003379 |
| hsa-miR-6765-5p | hsa_circ_0006254 |
| hsa-miR-6765-5p | hsa_circ_0003768 |
| hsa-miR-6765-5p | hsa_circ_0022383 |
| hsa-miR-6765-5p | hsa_circ_0000417 |
| hsa-miR-6765-5p | hsa_circ_0004008 |
| hsa-miR-6765-5p | hsa_circ_0035381 |
| hsa-miR-6765-5p | hsa_circ_0000660 |
| hsa-miR-6765-5p | hsa_circ_0007846 |
| hsa-miR-6765-5p | hsa_circ_0092337 |
| hsa-miR-6765-5p | hsa_circ_0045890 |
| hsa-miR-6765-5p | hsa_circ_0045905 |
| hsa-miR-6765-5p | hsa_circ_0046430 |
| hsa-miR-6765-5p | hsa_circ_0048025 |
| hsa-miR-6765-5p | hsa_circ_0004003 |
| hsa-miR-6765-5p | hsa_circ_0006877 |
| hsa-miR-6765-5p | hsa_circ_0000909 |
| hsa-miR-6765-5p | hsa_circ_0050119 |
| hsa-miR-6765-5p | hsa_circ_0092297 |
| hsa-miR-6765-5p | hsa_circ_0003854 |
| hsa-miR-6765-5p | hsa_circ_0001134 |
| hsa-miR-6765-5p | hsa_circ_0004218 |
| hsa-miR-6765-5p | hsa_circ_0061936 |
| hsa-miR-6765-5p | hsa_circ_0092299 |
| hsa-miR-6765-5p | hsa_circ_0002077 |
| hsa-miR-6765-5p | hsa_circ_0004705 |
| hsa-miR-6765-5p | hsa_circ_0066444 |
| hsa-miR-6765-5p | hsa_circ_0006681 |
| hsa-miR-6765-5p | hsa_circ_0001400 |
| hsa-miR-6765-5p | hsa_circ_0070467 |
| hsa-miR-6765-5p | hsa_circ_0071311 |
| hsa-miR-6765-5p | hsa_circ_0001472 |
| hsa-miR-6765-5p | hsa_circ_0074816 |
| hsa-miR-6765-5p | hsa_circ_0074817 |
| hsa-miR-6765-5p | hsa_circ_0007132 |
| hsa-miR-6765-5p | hsa_circ_0001756 |
| hsa-miR-6765-5p | hsa_circ_0002094 |
| hsa-miR-6765-5p | hsa_circ_0005630 |
| hsa-miR-6765-5p | hsa_circ_0084552 |
| hsa-miR-6765-5p | hsa_circ_0084789 |
| hsa-miR-6765-5p | hsa_circ_0001824 |
| hsa-miR-6765-5p | hsa_circ_0008934 |
| hsa-miR-6765-5p | hsa_circ_0006566 |
| hsa-miR-6765-5p | hsa_circ_0087305 |
| hsa-miR-6765-3p | hsa_circ_0007846 |
| hsa-miR-6765-3p | hsa_circ_0000690 |
| hsa-miR-6765-3p | hsa_circ_0046430 |
| hsa-miR-6765-3p | hsa_circ_0092283 |
| hsa-miR-6765-3p | hsa_circ_0092277 |
| hsa-miR-6765-3p | hsa_circ_0001400 |
| hsa-miR-6765-3p | hsa_circ_0007132 |
| hsa-miR-6765-3p | hsa_circ_0087288 |
| hsa-miR-6766-5p | hsa_circ_0092337 |
| hsa-miR-6766-5p | hsa_circ_0054144 |
| hsa-miR-6767-5p | hsa_circ_0008788 |
| hsa-miR-6767-5p | hsa_circ_0007132 |
| hsa-miR-6767-3p | hsa_circ_0022723 |
| hsa-miR-6768-5p | hsa_circ_0007372 |
| hsa-miR-6768-5p | hsa_circ_0092299 |
| hsa-miR-6768-5p | hsa_circ_0007132 |
| hsa-miR-6768-3p | hsa_circ_0009732 |
| hsa-miR-6769a-5p | hsa_circ_0000417 |
| hsa-miR-6769a-5p | hsa_circ_0007846 |
| hsa-miR-6769a-5p | hsa_circ_0040823 |
| hsa-miR-6769a-5p | hsa_circ_0000918 |
| hsa-miR-6769a-5p | hsa_circ_0069748 |
| hsa-miR-6769a-5p | hsa_circ_0003340 |
| hsa-miR-6769a-3p | hsa_circ_0007146 |
| hsa-miR-6769a-3p | hsa_circ_0048025 |
| hsa-miR-6769a-3p | hsa_circ_0092283 |
| hsa-miR-6769a-3p | hsa_circ_0004692 |
| hsa-miR-6769a-3p | hsa_circ_0065147 |
| hsa-miR-6769a-3p | hsa_circ_0065149 |
| hsa-miR-6769a-3p | hsa_circ_0002569 |
| hsa-miR-6769a-3p | hsa_circ_0002094 |
| hsa-miR-6770-5p | hsa_circ_0004003 |
| hsa-miR-6770-5p | hsa_circ_0004891 |
| hsa-miR-6770-3p | hsa_circ_0000462 |
| hsa-miR-6770-3p | hsa_circ_0002113 |
| hsa-miR-6770-3p | hsa_circ_0007685 |
| hsa-miR-6770-3p | hsa_circ_0001910 |
| hsa-miR-6771-5p | hsa_circ_0014132 |
| hsa-miR-6771-5p | hsa_circ_0015004 |
| hsa-miR-6771-5p | hsa_circ_0015453 |
| hsa-miR-6771-5p | hsa_circ_0007015 |
| hsa-miR-6771-5p | hsa_circ_0006608 |
| hsa-miR-6771-5p | hsa_circ_0000213 |
| hsa-miR-6771-5p | hsa_circ_0006649 |
| hsa-miR-6771-5p | hsa_circ_0007372 |
| hsa-miR-6771-5p | hsa_circ_0000417 |
| hsa-miR-6771-5p | hsa_circ_0031933 |
| hsa-miR-6771-5p | hsa_circ_0033144 |
| hsa-miR-6771-5p | hsa_circ_0034972 |
| hsa-miR-6771-5p | hsa_circ_0002696 |
| hsa-miR-6771-5p | hsa_circ_0049657 |
| hsa-miR-6771-5p | hsa_circ_0062397 |
| hsa-miR-6771-5p | hsa_circ_0092299 |
| hsa-miR-6771-5p | hsa_circ_0092283 |
| hsa-miR-6771-5p | hsa_circ_0004705 |
| hsa-miR-6771-5p | hsa_circ_0070467 |
| hsa-miR-6771-5p | hsa_circ_0071311 |
| hsa-miR-6771-5p | hsa_circ_0007132 |
| hsa-miR-6771-5p | hsa_circ_0003738 |
| hsa-miR-6771-5p | hsa_circ_0004381 |
| hsa-miR-6771-5p | hsa_circ_0003340 |
| hsa-miR-6771-5p | hsa_circ_0001708 |
| hsa-miR-6771-5p | hsa_circ_0005630 |
| hsa-miR-6771-3p | hsa_circ_0011120 |
| hsa-miR-6771-3p | hsa_circ_0008057 |
| hsa-miR-6771-3p | hsa_circ_0001829 |
| hsa-miR-6772-5p | hsa_circ_0003557 |
| hsa-miR-6772-5p | hsa_circ_0000417 |
| hsa-miR-6772-5p | hsa_circ_0007846 |
| hsa-miR-6772-5p | hsa_circ_0044195 |
| hsa-miR-6772-5p | hsa_circ_0008261 |
| hsa-miR-6772-5p | hsa_circ_0004470 |
| hsa-miR-6772-5p | hsa_circ_0092277 |
| hsa-miR-6772-5p | hsa_circ_0087305 |
| hsa-miR-6772-3p | hsa_circ_0000682 |
| hsa-miR-6772-3p | hsa_circ_0092297 |
| hsa-miR-6772-3p | hsa_circ_0092299 |
| hsa-miR-6773-5p | hsa_circ_0005782 |
| hsa-miR-6773-5p | hsa_circ_0072797 |
| hsa-miR-6774-5p | hsa_circ_0000375 |
| hsa-miR-6774-5p | hsa_circ_0000417 |
| hsa-miR-6774-5p | hsa_circ_0002696 |
| hsa-miR-6774-5p | hsa_circ_0040823 |
| hsa-miR-6774-5p | hsa_circ_0045890 |
| hsa-miR-6774-5p | hsa_circ_0045905 |
| hsa-miR-6774-5p | hsa_circ_0046430 |
| hsa-miR-6774-5p | hsa_circ_0004003 |
| hsa-miR-6774-5p | hsa_circ_0004891 |
| hsa-miR-6774-5p | hsa_circ_0050119 |
| hsa-miR-6774-5p | hsa_circ_0006693 |
| hsa-miR-6774-5p | hsa_circ_0001417 |
| hsa-miR-6774-5p | hsa_circ_0002490 |
| hsa-miR-6774-5p | hsa_circ_0074264 |
| hsa-miR-6774-5p | hsa_circ_0001658 |
| hsa-miR-6774-5p | hsa_circ_0082179 |
| hsa-miR-6774-5p | hsa_circ_0002094 |
| hsa-miR-6775-5p | hsa_circ_0002402 |
| hsa-miR-6775-5p | hsa_circ_0007437 |
| hsa-miR-6775-5p | hsa_circ_0007693 |
| hsa-miR-6775-5p | hsa_circ_0007015 |
| hsa-miR-6775-5p | hsa_circ_0016979 |
| hsa-miR-6775-5p | hsa_circ_0017248 |
| hsa-miR-6775-5p | hsa_circ_0017289 |
| hsa-miR-6775-5p | hsa_circ_0005090 |
| hsa-miR-6775-5p | hsa_circ_0018909 |
| hsa-miR-6775-5p | hsa_circ_0006649 |
| hsa-miR-6775-5p | hsa_circ_0008102 |
| hsa-miR-6775-5p | hsa_circ_0020005 |
| hsa-miR-6775-5p | hsa_circ_0003379 |
| hsa-miR-6775-5p | hsa_circ_0006254 |
| hsa-miR-6775-5p | hsa_circ_0003768 |
| hsa-miR-6775-5p | hsa_circ_0007001 |
| hsa-miR-6775-5p | hsa_circ_0022383 |
| hsa-miR-6775-5p | hsa_circ_0003557 |
| hsa-miR-6775-5p | hsa_circ_0000339 |
| hsa-miR-6775-5p | hsa_circ_0003908 |
| hsa-miR-6775-5p | hsa_circ_0000375 |
| hsa-miR-6775-5p | hsa_circ_0025388 |
| hsa-miR-6775-5p | hsa_circ_0003855 |
| hsa-miR-6775-5p | hsa_circ_0000417 |
| hsa-miR-6775-5p | hsa_circ_0028190 |
| hsa-miR-6775-5p | hsa_circ_0029633 |
| hsa-miR-6775-5p | hsa_circ_0000497 |
| hsa-miR-6775-5p | hsa_circ_0031933 |
| hsa-miR-6775-5p | hsa_circ_0033144 |
| hsa-miR-6775-5p | hsa_circ_0000592 |
| hsa-miR-6775-5p | hsa_circ_0003838 |
| hsa-miR-6775-5p | hsa_circ_0036763 |
| hsa-miR-6775-5p | hsa_circ_0000650 |
| hsa-miR-6775-5p | hsa_circ_0036768 |
| hsa-miR-6775-5p | hsa_circ_0007146 |
| hsa-miR-6775-5p | hsa_circ_0007846 |
| hsa-miR-6775-5p | hsa_circ_0040823 |
| hsa-miR-6775-5p | hsa_circ_0040921 |
| hsa-miR-6775-5p | hsa_circ_0045890 |
| hsa-miR-6775-5p | hsa_circ_0046430 |
| hsa-miR-6775-5p | hsa_circ_0047347 |
| hsa-miR-6775-5p | hsa_circ_0048025 |
| hsa-miR-6775-5p | hsa_circ_0004003 |
| hsa-miR-6775-5p | hsa_circ_0006877 |
| hsa-miR-6775-5p | hsa_circ_0049998 |
| hsa-miR-6775-5p | hsa_circ_0050119 |
| hsa-miR-6775-5p | hsa_circ_0008030 |
| hsa-miR-6775-5p | hsa_circ_0000936 |
| hsa-miR-6775-5p | hsa_circ_0052131 |
| hsa-miR-6775-5p | hsa_circ_0000973 |
| hsa-miR-6775-5p | hsa_circ_0008261 |
| hsa-miR-6775-5p | hsa_circ_0092297 |
| hsa-miR-6775-5p | hsa_circ_0002377 |
| hsa-miR-6775-5p | hsa_circ_0058522 |
| hsa-miR-6775-5p | hsa_circ_0004218 |
| hsa-miR-6775-5p | hsa_circ_0061052 |
| hsa-miR-6775-5p | hsa_circ_0002113 |
| hsa-miR-6775-5p | hsa_circ_0061936 |
| hsa-miR-6775-5p | hsa_circ_0092299 |
| hsa-miR-6775-5p | hsa_circ_0063331 |
| hsa-miR-6775-5p | hsa_circ_0002077 |
| hsa-miR-6775-5p | hsa_circ_0064136 |
| hsa-miR-6775-5p | hsa_circ_0004705 |
| hsa-miR-6775-5p | hsa_circ_0003602 |
| hsa-miR-6775-5p | hsa_circ_0065284 |
| hsa-miR-6775-5p | hsa_circ_0092277 |
| hsa-miR-6775-5p | hsa_circ_0001394 |
| hsa-miR-6775-5p | hsa_circ_0006693 |
| hsa-miR-6775-5p | hsa_circ_0001417 |
| hsa-miR-6775-5p | hsa_circ_0070467 |
| hsa-miR-6775-5p | hsa_circ_0001439 |
| hsa-miR-6775-5p | hsa_circ_0007137 |
| hsa-miR-6775-5p | hsa_circ_0073379 |
| hsa-miR-6775-5p | hsa_circ_0005540 |
| hsa-miR-6775-5p | hsa_circ_0001549 |
| hsa-miR-6775-5p | hsa_circ_0007132 |
| hsa-miR-6775-5p | hsa_circ_0004381 |
| hsa-miR-6775-5p | hsa_circ_0003340 |
| hsa-miR-6775-5p | hsa_circ_0007145 |
| hsa-miR-6775-5p | hsa_circ_0002755 |
| hsa-miR-6775-5p | hsa_circ_0081343 |
| hsa-miR-6775-5p | hsa_circ_0002094 |
| hsa-miR-6775-5p | hsa_circ_0005630 |
| hsa-miR-6775-5p | hsa_circ_0084429 |
| hsa-miR-6775-5p | hsa_circ_0084615 |
| hsa-miR-6775-5p | hsa_circ_0001806 |
| hsa-miR-6775-5p | hsa_circ_0084789 |
| hsa-miR-6775-5p | hsa_circ_0008934 |
| hsa-miR-6775-5p | hsa_circ_0005273 |
| hsa-miR-6775-5p | hsa_circ_0001829 |
| hsa-miR-6775-5p | hsa_circ_0006566 |
| hsa-miR-6775-5p | hsa_circ_0087288 |
| hsa-miR-6775-5p | hsa_circ_0087305 |
| hsa-miR-6775-5p | hsa_circ_0087855 |
| hsa-miR-6775-5p | hsa_circ_0008812 |
| hsa-miR-6775-5p | hsa_circ_0006174 |
| hsa-miR-6775-5p | hsa_circ_0087861 |
| hsa-miR-6775-5p | hsa_circ_0087862 |
| hsa-miR-6775-5p | hsa_circ_0001910 |
| hsa-miR-6775-3p | hsa_circ_0009732 |
| hsa-miR-6775-3p | hsa_circ_0007372 |
| hsa-miR-6775-3p | hsa_circ_0000417 |
| hsa-miR-6775-3p | hsa_circ_0045890 |
| hsa-miR-6775-3p | hsa_circ_0007967 |
| hsa-miR-6775-3p | hsa_circ_0002805 |
| hsa-miR-6775-3p | hsa_circ_0092299 |
| hsa-miR-6775-3p | hsa_circ_0007132 |
| hsa-miR-6775-3p | hsa_circ_0001756 |
| hsa-miR-6776-5p | hsa_circ_0006877 |
| hsa-miR-6776-3p | hsa_circ_0000395 |
| hsa-miR-6776-3p | hsa_circ_0030051 |
| hsa-miR-6776-3p | hsa_circ_0003838 |
| hsa-miR-6776-3p | hsa_circ_0071869 |
| hsa-miR-6777-5p | hsa_circ_0005576 |
| hsa-miR-6777-5p | hsa_circ_0007437 |
| hsa-miR-6777-5p | hsa_circ_0007693 |
| hsa-miR-6777-5p | hsa_circ_0016863 |
| hsa-miR-6777-5p | hsa_circ_0016867 |
| hsa-miR-6777-5p | hsa_circ_0003168 |
| hsa-miR-6777-5p | hsa_circ_0006735 |
| hsa-miR-6777-5p | hsa_circ_0002696 |
| hsa-miR-6777-5p | hsa_circ_0045890 |
| hsa-miR-6777-5p | hsa_circ_0000909 |
| hsa-miR-6777-5p | hsa_circ_0050119 |
| hsa-miR-6777-5p | hsa_circ_0092297 |
| hsa-miR-6777-5p | hsa_circ_0001134 |
| hsa-miR-6777-5p | hsa_circ_0070467 |
| hsa-miR-6777-5p | hsa_circ_0007132 |
| hsa-miR-6777-5p | hsa_circ_0005630 |
| hsa-miR-6777-3p | hsa_circ_0045890 |
| hsa-miR-6777-3p | hsa_circ_0084429 |
| hsa-miR-6778-5p | hsa_circ_0006649 |
| hsa-miR-6778-5p | hsa_circ_0003379 |
| hsa-miR-6778-5p | hsa_circ_0003557 |
| hsa-miR-6778-5p | hsa_circ_0029405 |
| hsa-miR-6778-5p | hsa_circ_0007846 |
| hsa-miR-6778-5p | hsa_circ_0002696 |
| hsa-miR-6778-5p | hsa_circ_0007609 |
| hsa-miR-6778-5p | hsa_circ_0007132 |
| hsa-miR-6778-5p | hsa_circ_0005630 |
| hsa-miR-6779-5p | hsa_circ_0017438 |
| hsa-miR-6779-5p | hsa_circ_0004277 |
| hsa-miR-6779-5p | hsa_circ_0017446 |
| hsa-miR-6779-5p | hsa_circ_0020005 |
| hsa-miR-6779-5p | hsa_circ_0006254 |
| hsa-miR-6779-5p | hsa_circ_0003768 |
| hsa-miR-6779-5p | hsa_circ_0007372 |
| hsa-miR-6779-5p | hsa_circ_0000417 |
| hsa-miR-6779-5p | hsa_circ_0031933 |
| hsa-miR-6779-5p | hsa_circ_0003838 |
| hsa-miR-6779-5p | hsa_circ_0000651 |
| hsa-miR-6779-5p | hsa_circ_0007146 |
| hsa-miR-6779-5p | hsa_circ_0045890 |
| hsa-miR-6779-5p | hsa_circ_0046430 |
| hsa-miR-6779-5p | hsa_circ_0049998 |
| hsa-miR-6779-5p | hsa_circ_0000918 |
| hsa-miR-6779-5p | hsa_circ_0003854 |
| hsa-miR-6779-5p | hsa_circ_0092299 |
| hsa-miR-6779-5p | hsa_circ_0006681 |
| hsa-miR-6779-5p | hsa_circ_0070040 |
| hsa-miR-6779-5p | hsa_circ_0001549 |
| hsa-miR-6779-5p | hsa_circ_0001573 |
| hsa-miR-6779-5p | hsa_circ_0008536 |
| hsa-miR-6779-5p | hsa_circ_0077765 |
| hsa-miR-6779-5p | hsa_circ_0001658 |
| hsa-miR-6779-5p | hsa_circ_0009092 |
| hsa-miR-6779-5p | hsa_circ_0001756 |
| hsa-miR-6779-5p | hsa_circ_0005630 |
| hsa-miR-6780a-5p | hsa_circ_0015004 |
| hsa-miR-6780a-5p | hsa_circ_0000220 |
| hsa-miR-6780a-5p | hsa_circ_0006665 |
| hsa-miR-6780a-5p | hsa_circ_0000417 |
| hsa-miR-6780a-5p | hsa_circ_0003489 |
| hsa-miR-6780a-5p | hsa_circ_0040039 |
| hsa-miR-6780a-5p | hsa_circ_0004354 |
| hsa-miR-6780a-5p | hsa_circ_0055904 |
| hsa-miR-6780a-5p | hsa_circ_0002805 |
| hsa-miR-6780a-5p | hsa_circ_0001910 |
| hsa-miR-6781-5p | hsa_circ_0015004 |
| hsa-miR-6781-5p | hsa_circ_0003168 |
| hsa-miR-6781-5p | hsa_circ_0022383 |
| hsa-miR-6781-5p | hsa_circ_0000339 |
| hsa-miR-6781-5p | hsa_circ_0007372 |
| hsa-miR-6781-5p | hsa_circ_0000417 |
| hsa-miR-6781-5p | hsa_circ_0000650 |
| hsa-miR-6781-5p | hsa_circ_0036768 |
| hsa-miR-6781-5p | hsa_circ_0045890 |
| hsa-miR-6781-5p | hsa_circ_0002805 |
| hsa-miR-6781-5p | hsa_circ_0061179 |
| hsa-miR-6781-5p | hsa_circ_0092299 |
| hsa-miR-6781-5p | hsa_circ_0002077 |
| hsa-miR-6781-5p | hsa_circ_0007132 |
| hsa-miR-6781-3p | hsa_circ_0006597 |
| hsa-miR-6781-3p | hsa_circ_0007132 |
| hsa-miR-6782-5p | hsa_circ_0014132 |
| hsa-miR-6782-5p | hsa_circ_0000131 |
| hsa-miR-6782-5p | hsa_circ_0016863 |
| hsa-miR-6782-5p | hsa_circ_0016867 |
| hsa-miR-6782-5p | hsa_circ_0000220 |
| hsa-miR-6782-5p | hsa_circ_0006665 |
| hsa-miR-6782-5p | hsa_circ_0008102 |
| hsa-miR-6782-5p | hsa_circ_0006254 |
| hsa-miR-6782-5p | hsa_circ_0003768 |
| hsa-miR-6782-5p | hsa_circ_0000375 |
| hsa-miR-6782-5p | hsa_circ_0000417 |
| hsa-miR-6782-5p | hsa_circ_0029405 |
| hsa-miR-6782-5p | hsa_circ_0002696 |
| hsa-miR-6782-5p | hsa_circ_0040039 |
| hsa-miR-6782-5p | hsa_circ_0004354 |
| hsa-miR-6782-5p | hsa_circ_0046430 |
| hsa-miR-6782-5p | hsa_circ_0048025 |
| hsa-miR-6782-5p | hsa_circ_0005571 |
| hsa-miR-6782-5p | hsa_circ_0061052 |
| hsa-miR-6782-5p | hsa_circ_0092299 |
| hsa-miR-6782-5p | hsa_circ_0004705 |
| hsa-miR-6782-5p | hsa_circ_0092277 |
| hsa-miR-6782-5p | hsa_circ_0001394 |
| hsa-miR-6782-5p | hsa_circ_0069748 |
| hsa-miR-6782-5p | hsa_circ_0070467 |
| hsa-miR-6782-5p | hsa_circ_0071311 |
| hsa-miR-6782-5p | hsa_circ_0073379 |
| hsa-miR-6782-5p | hsa_circ_0005540 |
| hsa-miR-6782-5p | hsa_circ_0007132 |
| hsa-miR-6782-5p | hsa_circ_0009092 |
| hsa-miR-6782-5p | hsa_circ_0001708 |
| hsa-miR-6782-5p | hsa_circ_0005630 |
| hsa-miR-6782-5p | hsa_circ_0006566 |
| hsa-miR-6782-5p | hsa_circ_0087288 |
| hsa-miR-6782-5p | hsa_circ_0001866 |
| hsa-miR-6782-5p | hsa_circ_0087855 |
| hsa-miR-6782-5p | hsa_circ_0008812 |
| hsa-miR-6782-5p | hsa_circ_0006174 |
| hsa-miR-6782-5p | hsa_circ_0087861 |
| hsa-miR-6782-5p | hsa_circ_0087862 |
| hsa-miR-6782-3p | hsa_circ_0020594 |
| hsa-miR-6782-3p | hsa_circ_0000395 |
| hsa-miR-6782-3p | hsa_circ_0040039 |
| hsa-miR-6782-3p | hsa_circ_0004354 |
| hsa-miR-6782-3p | hsa_circ_0006693 |
| hsa-miR-6782-3p | hsa_circ_0001417 |
| hsa-miR-6782-3p | hsa_circ_0071865 |
| hsa-miR-6783-5p | hsa_circ_0092299 |
| hsa-miR-6783-3p | hsa_circ_0046430 |
| hsa-miR-6783-3p | hsa_circ_0003602 |
| hsa-miR-6783-3p | hsa_circ_0008639 |
| hsa-miR-6783-3p | hsa_circ_0069399 |
| hsa-miR-6783-3p | hsa_circ_0007132 |
| hsa-miR-6783-3p | hsa_circ_0005630 |
| hsa-miR-6784-5p | hsa_circ_0018909 |
| hsa-miR-6784-5p | hsa_circ_0003168 |
| hsa-miR-6784-5p | hsa_circ_0025388 |
| hsa-miR-6784-5p | hsa_circ_0007146 |
| hsa-miR-6784-5p | hsa_circ_0049998 |
| hsa-miR-6784-5p | hsa_circ_0004705 |
| hsa-miR-6784-5p | hsa_circ_0007132 |
| hsa-miR-6784-5p | hsa_circ_0078522 |
| hsa-miR-6784-3p | hsa_circ_0092337 |
| hsa-miR-6785-5p | hsa_circ_0011422 |
| hsa-miR-6785-5p | hsa_circ_0007437 |
| hsa-miR-6785-5p | hsa_circ_0007693 |
| hsa-miR-6785-5p | hsa_circ_0017438 |
| hsa-miR-6785-5p | hsa_circ_0004277 |
| hsa-miR-6785-5p | hsa_circ_0017446 |
| hsa-miR-6785-5p | hsa_circ_0006649 |
| hsa-miR-6785-5p | hsa_circ_0003557 |
| hsa-miR-6785-5p | hsa_circ_0003854 |
| hsa-miR-6785-5p | hsa_circ_0004218 |
| hsa-miR-6785-5p | hsa_circ_0004692 |
| hsa-miR-6785-5p | hsa_circ_0070467 |
| hsa-miR-6785-3p | hsa_circ_0092299 |
| hsa-miR-6785-3p | hsa_circ_0092283 |
| hsa-miR-6785-3p | hsa_circ_0070396 |
| hsa-miR-6785-3p | hsa_circ_0007132 |
| hsa-miR-6786-5p | hsa_circ_0007015 |
| hsa-miR-6786-5p | hsa_circ_0017248 |
| hsa-miR-6786-5p | hsa_circ_0020594 |
| hsa-miR-6786-5p | hsa_circ_0022383 |
| hsa-miR-6786-5p | hsa_circ_0000375 |
| hsa-miR-6786-5p | hsa_circ_0000417 |
| hsa-miR-6786-5p | hsa_circ_0031933 |
| hsa-miR-6786-5p | hsa_circ_0040921 |
| hsa-miR-6786-5p | hsa_circ_0045890 |
| hsa-miR-6786-5p | hsa_circ_0046430 |
| hsa-miR-6786-5p | hsa_circ_0003854 |
| hsa-miR-6786-5p | hsa_circ_0004218 |
| hsa-miR-6786-5p | hsa_circ_0092299 |
| hsa-miR-6786-5p | hsa_circ_0064136 |
| hsa-miR-6786-5p | hsa_circ_0001394 |
| hsa-miR-6786-5p | hsa_circ_0001400 |
| hsa-miR-6786-5p | hsa_circ_0001549 |
| hsa-miR-6786-5p | hsa_circ_0007132 |
| hsa-miR-6786-5p | hsa_circ_0003340 |
| hsa-miR-6786-5p | hsa_circ_0001756 |
| hsa-miR-6786-5p | hsa_circ_0001806 |
| hsa-miR-6786-5p | hsa_circ_0001829 |
| hsa-miR-6787-5p | hsa_circ_0006137 |
| hsa-miR-6787-5p | hsa_circ_0007146 |
| hsa-miR-6787-5p | hsa_circ_0092337 |
| hsa-miR-6787-5p | hsa_circ_0046430 |
| hsa-miR-6787-5p | hsa_circ_0048025 |
| hsa-miR-6787-5p | hsa_circ_0006877 |
| hsa-miR-6787-5p | hsa_circ_0003854 |
| hsa-miR-6787-5p | hsa_circ_0002805 |
| hsa-miR-6787-5p | hsa_circ_0061179 |
| hsa-miR-6787-5p | hsa_circ_0001472 |
| hsa-miR-6787-5p | hsa_circ_0001573 |
| hsa-miR-6787-5p | hsa_circ_0007132 |
| hsa-miR-6787-5p | hsa_circ_0004381 |
| hsa-miR-6787-5p | hsa_circ_0002755 |
| hsa-miR-6787-5p | hsa_circ_0005630 |
| hsa-miR-6787-3p | hsa_circ_0020594 |
| hsa-miR-6787-3p | hsa_circ_0049998 |
| hsa-miR-6787-3p | hsa_circ_0004692 |
| hsa-miR-6787-3p | hsa_circ_0001707 |
| hsa-miR-6787-3p | hsa_circ_0087305 |
| hsa-miR-6788-3p | hsa_circ_0040921 |
| hsa-miR-6788-3p | hsa_circ_0067913 |
| hsa-miR-6788-3p | hsa_circ_0007132 |
| hsa-miR-6789-5p | hsa_circ_0002402 |
| hsa-miR-6789-5p | hsa_circ_0002570 |
| hsa-miR-6789-5p | hsa_circ_0007015 |
| hsa-miR-6789-5p | hsa_circ_0008788 |
| hsa-miR-6789-5p | hsa_circ_0017446 |
| hsa-miR-6789-5p | hsa_circ_0000206 |
| hsa-miR-6789-5p | hsa_circ_0006649 |
| hsa-miR-6789-5p | hsa_circ_0003379 |
| hsa-miR-6789-5p | hsa_circ_0006254 |
| hsa-miR-6789-5p | hsa_circ_0003768 |
| hsa-miR-6789-5p | hsa_circ_0022392 |
| hsa-miR-6789-5p | hsa_circ_0003557 |
| hsa-miR-6789-5p | hsa_circ_0000375 |
| hsa-miR-6789-5p | hsa_circ_0000417 |
| hsa-miR-6789-5p | hsa_circ_0028190 |
| hsa-miR-6789-5p | hsa_circ_0000660 |
| hsa-miR-6789-5p | hsa_circ_0006735 |
| hsa-miR-6789-5p | hsa_circ_0000682 |
| hsa-miR-6789-5p | hsa_circ_0000690 |
| hsa-miR-6789-5p | hsa_circ_0004519 |
| hsa-miR-6789-5p | hsa_circ_0040921 |
| hsa-miR-6789-5p | hsa_circ_0043522 |
| hsa-miR-6789-5p | hsa_circ_0045890 |
| hsa-miR-6789-5p | hsa_circ_0046430 |
| hsa-miR-6789-5p | hsa_circ_0048025 |
| hsa-miR-6789-5p | hsa_circ_0002926 |
| hsa-miR-6789-5p | hsa_circ_0049998 |
| hsa-miR-6789-5p | hsa_circ_0005571 |
| hsa-miR-6789-5p | hsa_circ_0050119 |
| hsa-miR-6789-5p | hsa_circ_0000950 |
| hsa-miR-6789-5p | hsa_circ_0092297 |
| hsa-miR-6789-5p | hsa_circ_0003854 |
| hsa-miR-6789-5p | hsa_circ_0060904 |
| hsa-miR-6789-5p | hsa_circ_0061052 |
| hsa-miR-6789-5p | hsa_circ_0002805 |
| hsa-miR-6789-5p | hsa_circ_0061179 |
| hsa-miR-6789-5p | hsa_circ_0092299 |
| hsa-miR-6789-5p | hsa_circ_0092283 |
| hsa-miR-6789-5p | hsa_circ_0001394 |
| hsa-miR-6789-5p | hsa_circ_0001400 |
| hsa-miR-6789-5p | hsa_circ_0001439 |
| hsa-miR-6789-5p | hsa_circ_0072857 |
| hsa-miR-6789-5p | hsa_circ_0073379 |
| hsa-miR-6789-5p | hsa_circ_0005540 |
| hsa-miR-6789-5p | hsa_circ_0003528 |
| hsa-miR-6789-5p | hsa_circ_0007132 |
| hsa-miR-6789-5p | hsa_circ_0004381 |
| hsa-miR-6789-5p | hsa_circ_0003340 |
| hsa-miR-6789-5p | hsa_circ_0081343 |
| hsa-miR-6789-5p | hsa_circ_0002094 |
| hsa-miR-6789-5p | hsa_circ_0005630 |
| hsa-miR-6789-5p | hsa_circ_0084789 |
| hsa-miR-6789-5p | hsa_circ_0001947 |
| hsa-miR-6789-3p | hsa_circ_0017639 |
| hsa-miR-6789-3p | hsa_circ_0000395 |
| hsa-miR-6789-3p | hsa_circ_0092297 |
| hsa-miR-6789-3p | hsa_circ_0004692 |
| hsa-miR-6789-3p | hsa_circ_0007132 |
| hsa-miR-6789-3p | hsa_circ_0084789 |
| hsa-miR-6790-5p | hsa_circ_0009349 |
| hsa-miR-6790-5p | hsa_circ_0007001 |
| hsa-miR-6790-3p | hsa_circ_0092297 |
| hsa-miR-6790-3p | hsa_circ_0007132 |
| hsa-miR-6790-3p | hsa_circ_0084789 |
| hsa-miR-6791-5p | hsa_circ_0002402 |
| hsa-miR-6791-5p | hsa_circ_0014132 |
| hsa-miR-6791-5p | hsa_circ_0007015 |
| hsa-miR-6791-5p | hsa_circ_0008102 |
| hsa-miR-6791-5p | hsa_circ_0003168 |
| hsa-miR-6791-5p | hsa_circ_0003379 |
| hsa-miR-6791-5p | hsa_circ_0006254 |
| hsa-miR-6791-5p | hsa_circ_0003768 |
| hsa-miR-6791-5p | hsa_circ_0022723 |
| hsa-miR-6791-5p | hsa_circ_0000417 |
| hsa-miR-6791-5p | hsa_circ_0031933 |
| hsa-miR-6791-5p | hsa_circ_0033144 |
| hsa-miR-6791-5p | hsa_circ_0007146 |
| hsa-miR-6791-5p | hsa_circ_0006735 |
| hsa-miR-6791-5p | hsa_circ_0000682 |
| hsa-miR-6791-5p | hsa_circ_0040921 |
| hsa-miR-6791-5p | hsa_circ_0041050 |
| hsa-miR-6791-5p | hsa_circ_0045890 |
| hsa-miR-6791-5p | hsa_circ_0046430 |
| hsa-miR-6791-5p | hsa_circ_0003865 |
| hsa-miR-6791-5p | hsa_circ_0000836 |
| hsa-miR-6791-5p | hsa_circ_0049998 |
| hsa-miR-6791-5p | hsa_circ_0092297 |
| hsa-miR-6791-5p | hsa_circ_0002805 |
| hsa-miR-6791-5p | hsa_circ_0061179 |
| hsa-miR-6791-5p | hsa_circ_0001187 |
| hsa-miR-6791-5p | hsa_circ_0008078 |
| hsa-miR-6791-5p | hsa_circ_0092299 |
| hsa-miR-6791-5p | hsa_circ_0092277 |
| hsa-miR-6791-5p | hsa_circ_0070467 |
| hsa-miR-6791-5p | hsa_circ_0071311 |
| hsa-miR-6791-5p | hsa_circ_0072437 |
| hsa-miR-6791-5p | hsa_circ_0007132 |
| hsa-miR-6791-5p | hsa_circ_0004381 |
| hsa-miR-6791-5p | hsa_circ_0003340 |
| hsa-miR-6791-5p | hsa_circ_0002755 |
| hsa-miR-6791-5p | hsa_circ_0001748 |
| hsa-miR-6791-5p | hsa_circ_0002094 |
| hsa-miR-6791-5p | hsa_circ_0005630 |
| hsa-miR-6791-5p | hsa_circ_0084429 |
| hsa-miR-6791-5p | hsa_circ_0001829 |
| hsa-miR-6791-5p | hsa_circ_0087305 |
| hsa-miR-6791-5p | hsa_circ_0001947 |
| hsa-miR-6791-3p | hsa_circ_0041050 |
| hsa-miR-6791-3p | hsa_circ_0092277 |
| hsa-miR-6791-3p | hsa_circ_0007145 |
| hsa-miR-6792-5p | hsa_circ_0004218 |
| hsa-miR-6792-5p | hsa_circ_0004712 |
| hsa-miR-6792-3p | hsa_circ_0000417 |
| hsa-miR-6792-3p | hsa_circ_0092299 |
| hsa-miR-6792-3p | hsa_circ_0007132 |
| hsa-miR-6793-5p | hsa_circ_0020594 |
| hsa-miR-6793-5p | hsa_circ_0006254 |
| hsa-miR-6793-5p | hsa_circ_0003768 |
| hsa-miR-6793-5p | hsa_circ_0022383 |
| hsa-miR-6793-5p | hsa_circ_0007846 |
| hsa-miR-6793-5p | hsa_circ_0000854 |
| hsa-miR-6793-5p | hsa_circ_0006006 |
| hsa-miR-6793-5p | hsa_circ_0007609 |
| hsa-miR-6793-5p | hsa_circ_0092283 |
| hsa-miR-6793-5p | hsa_circ_0001748 |
| hsa-miR-6793-5p | hsa_circ_0002094 |
| hsa-miR-6793-3p | hsa_circ_0092299 |
| hsa-miR-6793-3p | hsa_circ_0070396 |
| hsa-miR-6793-3p | hsa_circ_0007132 |
| hsa-miR-6794-5p | hsa_circ_0000417 |
| hsa-miR-6794-5p | hsa_circ_0000497 |
| hsa-miR-6794-5p | hsa_circ_0046430 |
| hsa-miR-6794-5p | hsa_circ_0052131 |
| hsa-miR-6794-5p | hsa_circ_0003854 |
| hsa-miR-6794-5p | hsa_circ_0004218 |
| hsa-miR-6794-5p | hsa_circ_0092277 |
| hsa-miR-6794-5p | hsa_circ_0009092 |
| hsa-miR-6794-5p | hsa_circ_0006566 |
| hsa-miR-6795-5p | hsa_circ_0003168 |
| hsa-miR-6795-5p | hsa_circ_0000375 |
| hsa-miR-6795-5p | hsa_circ_0000417 |
| hsa-miR-6795-5p | hsa_circ_0000497 |
| hsa-miR-6795-5p | hsa_circ_0004137 |
| hsa-miR-6795-5p | hsa_circ_0031933 |
| hsa-miR-6795-5p | hsa_circ_0007846 |
| hsa-miR-6795-5p | hsa_circ_0046430 |
| hsa-miR-6795-5p | hsa_circ_0092299 |
| hsa-miR-6795-5p | hsa_circ_0092277 |
| hsa-miR-6795-5p | hsa_circ_0070467 |
| hsa-miR-6795-5p | hsa_circ_0071869 |
| hsa-miR-6795-5p | hsa_circ_0073379 |
| hsa-miR-6795-5p | hsa_circ_0005540 |
| hsa-miR-6795-5p | hsa_circ_0002037 |
| hsa-miR-6795-5p | hsa_circ_0081423 |
| hsa-miR-6795-5p | hsa_circ_0002094 |
| hsa-miR-6795-5p | hsa_circ_0001806 |
| hsa-miR-6795-5p | hsa_circ_0087305 |
| hsa-miR-6795-5p | hsa_circ_0001866 |
| hsa-miR-6795-5p | hsa_circ_0087855 |
| hsa-miR-6795-5p | hsa_circ_0008812 |
| hsa-miR-6795-5p | hsa_circ_0006174 |
| hsa-miR-6795-5p | hsa_circ_0087861 |
| hsa-miR-6795-5p | hsa_circ_0087862 |
| hsa-miR-6795-3p | hsa_circ_0000395 |
| hsa-miR-6795-3p | hsa_circ_0000996 |
| hsa-miR-6795-3p | hsa_circ_0002094 |
| hsa-miR-6796-5p | hsa_circ_0017639 |
| hsa-miR-6796-5p | hsa_circ_0008102 |
| hsa-miR-6796-5p | hsa_circ_0036763 |
| hsa-miR-6796-5p | hsa_circ_0000650 |
| hsa-miR-6796-5p | hsa_circ_0036768 |
| hsa-miR-6796-5p | hsa_circ_0000682 |
| hsa-miR-6796-5p | hsa_circ_0046430 |
| hsa-miR-6796-5p | hsa_circ_0048025 |
| hsa-miR-6796-5p | hsa_circ_0004003 |
| hsa-miR-6796-5p | hsa_circ_0004891 |
| hsa-miR-6796-5p | hsa_circ_0050119 |
| hsa-miR-6796-5p | hsa_circ_0092283 |
| hsa-miR-6796-5p | hsa_circ_0063331 |
| hsa-miR-6796-5p | hsa_circ_0001258 |
| hsa-miR-6796-5p | hsa_circ_0071311 |
| hsa-miR-6796-5p | hsa_circ_0072857 |
| hsa-miR-6796-5p | hsa_circ_0073379 |
| hsa-miR-6796-5p | hsa_circ_0005540 |
| hsa-miR-6796-5p | hsa_circ_0001573 |
| hsa-miR-6796-5p | hsa_circ_0004381 |
| hsa-miR-6796-5p | hsa_circ_0087305 |
| hsa-miR-6796-5p | hsa_circ_0087855 |
| hsa-miR-6796-5p | hsa_circ_0008812 |
| hsa-miR-6796-5p | hsa_circ_0006174 |
| hsa-miR-6796-5p | hsa_circ_0087861 |
| hsa-miR-6796-5p | hsa_circ_0087862 |
| hsa-miR-6796-3p | hsa_circ_0002805 |
| hsa-miR-6797-5p | hsa_circ_0000417 |
| hsa-miR-6797-5p | hsa_circ_0004137 |
| hsa-miR-6797-5p | hsa_circ_0007846 |
| hsa-miR-6797-5p | hsa_circ_0040921 |
| hsa-miR-6797-5p | hsa_circ_0046430 |
| hsa-miR-6797-5p | hsa_circ_0008030 |
| hsa-miR-6797-5p | hsa_circ_0000973 |
| hsa-miR-6797-5p | hsa_circ_0002077 |
| hsa-miR-6797-5p | hsa_circ_0003602 |
| hsa-miR-6797-5p | hsa_circ_0001573 |
| hsa-miR-6797-5p | hsa_circ_0005630 |
| hsa-miR-6797-5p | hsa_circ_0084429 |
| hsa-miR-6797-5p | hsa_circ_0001829 |
| hsa-miR-6797-3p | hsa_circ_0000463 |
| hsa-miR-6797-3p | hsa_circ_0007132 |
| hsa-miR-6798-5p | hsa_circ_0009581 |
| hsa-miR-6798-5p | hsa_circ_0006837 |
| hsa-miR-6798-5p | hsa_circ_0002402 |
| hsa-miR-6798-5p | hsa_circ_0007437 |
| hsa-miR-6798-5p | hsa_circ_0007693 |
| hsa-miR-6798-5p | hsa_circ_0007015 |
| hsa-miR-6798-5p | hsa_circ_0003379 |
| hsa-miR-6798-5p | hsa_circ_0006254 |
| hsa-miR-6798-5p | hsa_circ_0003768 |
| hsa-miR-6798-5p | hsa_circ_0000375 |
| hsa-miR-6798-5p | hsa_circ_0025388 |
| hsa-miR-6798-5p | hsa_circ_0000417 |
| hsa-miR-6798-5p | hsa_circ_0029405 |
| hsa-miR-6798-5p | hsa_circ_0029633 |
| hsa-miR-6798-5p | hsa_circ_0007846 |
| hsa-miR-6798-5p | hsa_circ_0004519 |
| hsa-miR-6798-5p | hsa_circ_0040823 |
| hsa-miR-6798-5p | hsa_circ_0043691 |
| hsa-miR-6798-5p | hsa_circ_0046430 |
| hsa-miR-6798-5p | hsa_circ_0048025 |
| hsa-miR-6798-5p | hsa_circ_0002926 |
| hsa-miR-6798-5p | hsa_circ_0049657 |
| hsa-miR-6798-5p | hsa_circ_0000918 |
| hsa-miR-6798-5p | hsa_circ_0000936 |
| hsa-miR-6798-5p | hsa_circ_0000973 |
| hsa-miR-6798-5p | hsa_circ_0003854 |
| hsa-miR-6798-5p | hsa_circ_0004218 |
| hsa-miR-6798-5p | hsa_circ_0092299 |
| hsa-miR-6798-5p | hsa_circ_0004470 |
| hsa-miR-6798-5p | hsa_circ_0001247 |
| hsa-miR-6798-5p | hsa_circ_0001400 |
| hsa-miR-6798-5p | hsa_circ_0072437 |
| hsa-miR-6798-5p | hsa_circ_0001573 |
| hsa-miR-6798-5p | hsa_circ_0007132 |
| hsa-miR-6798-5p | hsa_circ_0004381 |
| hsa-miR-6798-5p | hsa_circ_0079480 |
| hsa-miR-6798-5p | hsa_circ_0003340 |
| hsa-miR-6798-5p | hsa_circ_0081423 |
| hsa-miR-6798-5p | hsa_circ_0001756 |
| hsa-miR-6799-5p | hsa_circ_0000375 |
| hsa-miR-6799-5p | hsa_circ_0046430 |
| hsa-miR-6799-5p | hsa_circ_0002926 |
| hsa-miR-6799-5p | hsa_circ_0049657 |
| hsa-miR-6799-5p | hsa_circ_0058522 |
| hsa-miR-6799-5p | hsa_circ_0070467 |
| hsa-miR-6799-5p | hsa_circ_0072857 |
| hsa-miR-6799-3p | hsa_circ_0003379 |
| hsa-miR-6799-3p | hsa_circ_0000690 |
| hsa-miR-6799-3p | hsa_circ_0003854 |
| hsa-miR-6799-3p | hsa_circ_0007132 |
| hsa-miR-6800-5p | hsa_circ_0048025 |
| hsa-miR-6800-3p | hsa_circ_0034920 |
| hsa-miR-6800-3p | hsa_circ_0040921 |
| hsa-miR-6800-3p | hsa_circ_0074816 |
| hsa-miR-6800-3p | hsa_circ_0074817 |
| hsa-miR-6800-3p | hsa_circ_0005630 |
| hsa-miR-6800-3p | hsa_circ_0008113 |
| hsa-miR-6801-3p | hsa_circ_0011571 |
| hsa-miR-6801-3p | hsa_circ_0011572 |
| hsa-miR-6802-5p | hsa_circ_0000417 |
| hsa-miR-6802-3p | hsa_circ_0092299 |
| hsa-miR-6803-5p | hsa_circ_0005782 |
| hsa-miR-6803-5p | hsa_circ_0002402 |
| hsa-miR-6803-5p | hsa_circ_0014132 |
| hsa-miR-6803-5p | hsa_circ_0015004 |
| hsa-miR-6803-5p | hsa_circ_0007015 |
| hsa-miR-6803-5p | hsa_circ_0017289 |
| hsa-miR-6803-5p | hsa_circ_0017438 |
| hsa-miR-6803-5p | hsa_circ_0004277 |
| hsa-miR-6803-5p | hsa_circ_0017446 |
| hsa-miR-6803-5p | hsa_circ_0000206 |
| hsa-miR-6803-5p | hsa_circ_0017461 |
| hsa-miR-6803-5p | hsa_circ_0006649 |
| hsa-miR-6803-5p | hsa_circ_0003168 |
| hsa-miR-6803-5p | hsa_circ_0020005 |
| hsa-miR-6803-5p | hsa_circ_0003379 |
| hsa-miR-6803-5p | hsa_circ_0006254 |
| hsa-miR-6803-5p | hsa_circ_0003768 |
| hsa-miR-6803-5p | hsa_circ_0022505 |
| hsa-miR-6803-5p | hsa_circ_0022723 |
| hsa-miR-6803-5p | hsa_circ_0002884 |
| hsa-miR-6803-5p | hsa_circ_0002264 |
| hsa-miR-6803-5p | hsa_circ_0007372 |
| hsa-miR-6803-5p | hsa_circ_0000375 |
| hsa-miR-6803-5p | hsa_circ_0025388 |
| hsa-miR-6803-5p | hsa_circ_0000417 |
| hsa-miR-6803-5p | hsa_circ_0029633 |
| hsa-miR-6803-5p | hsa_circ_0030051 |
| hsa-miR-6803-5p | hsa_circ_0000497 |
| hsa-miR-6803-5p | hsa_circ_0031933 |
| hsa-miR-6803-5p | hsa_circ_0008521 |
| hsa-miR-6803-5p | hsa_circ_0003848 |
| hsa-miR-6803-5p | hsa_circ_0002564 |
| hsa-miR-6803-5p | hsa_circ_0002289 |
| hsa-miR-6803-5p | hsa_circ_0033144 |
| hsa-miR-6803-5p | hsa_circ_0007146 |
| hsa-miR-6803-5p | hsa_circ_0007846 |
| hsa-miR-6803-5p | hsa_circ_0000682 |
| hsa-miR-6803-5p | hsa_circ_0040823 |
| hsa-miR-6803-5p | hsa_circ_0040921 |
| hsa-miR-6803-5p | hsa_circ_0042799 |
| hsa-miR-6803-5p | hsa_circ_0045890 |
| hsa-miR-6803-5p | hsa_circ_0046430 |
| hsa-miR-6803-5p | hsa_circ_0048025 |
| hsa-miR-6803-5p | hsa_circ_0004003 |
| hsa-miR-6803-5p | hsa_circ_0004891 |
| hsa-miR-6803-5p | hsa_circ_0049998 |
| hsa-miR-6803-5p | hsa_circ_0050119 |
| hsa-miR-6803-5p | hsa_circ_0008030 |
| hsa-miR-6803-5p | hsa_circ_0000936 |
| hsa-miR-6803-5p | hsa_circ_0000950 |
| hsa-miR-6803-5p | hsa_circ_0052131 |
| hsa-miR-6803-5p | hsa_circ_0054086 |
| hsa-miR-6803-5p | hsa_circ_0000996 |
| hsa-miR-6803-5p | hsa_circ_0003854 |
| hsa-miR-6803-5p | hsa_circ_0004218 |
| hsa-miR-6803-5p | hsa_circ_0061052 |
| hsa-miR-6803-5p | hsa_circ_0002805 |
| hsa-miR-6803-5p | hsa_circ_0061179 |
| hsa-miR-6803-5p | hsa_circ_0007609 |
| hsa-miR-6803-5p | hsa_circ_0061936 |
| hsa-miR-6803-5p | hsa_circ_0092299 |
| hsa-miR-6803-5p | hsa_circ_0064136 |
| hsa-miR-6803-5p | hsa_circ_0003602 |
| hsa-miR-6803-5p | hsa_circ_0092277 |
| hsa-miR-6803-5p | hsa_circ_0001394 |
| hsa-miR-6803-5p | hsa_circ_0070467 |
| hsa-miR-6803-5p | hsa_circ_0071311 |
| hsa-miR-6803-5p | hsa_circ_0001472 |
| hsa-miR-6803-5p | hsa_circ_0072437 |
| hsa-miR-6803-5p | hsa_circ_0002490 |
| hsa-miR-6803-5p | hsa_circ_0073379 |
| hsa-miR-6803-5p | hsa_circ_0005540 |
| hsa-miR-6803-5p | hsa_circ_0074264 |
| hsa-miR-6803-5p | hsa_circ_0001573 |
| hsa-miR-6803-5p | hsa_circ_0007132 |
| hsa-miR-6803-5p | hsa_circ_0075748 |
| hsa-miR-6803-5p | hsa_circ_0076793 |
| hsa-miR-6803-5p | hsa_circ_0008536 |
| hsa-miR-6803-5p | hsa_circ_0077765 |
| hsa-miR-6803-5p | hsa_circ_0003340 |
| hsa-miR-6803-5p | hsa_circ_0009092 |
| hsa-miR-6803-5p | hsa_circ_0002755 |
| hsa-miR-6803-5p | hsa_circ_0002094 |
| hsa-miR-6803-5p | hsa_circ_0005630 |
| hsa-miR-6803-5p | hsa_circ_0007521 |
| hsa-miR-6803-5p | hsa_circ_0005982 |
| hsa-miR-6803-5p | hsa_circ_0003221 |
| hsa-miR-6803-5p | hsa_circ_0001829 |
| hsa-miR-6803-5p | hsa_circ_0087288 |
| hsa-miR-6803-5p | hsa_circ_0087305 |
| hsa-miR-6803-5p | hsa_circ_0087855 |
| hsa-miR-6803-5p | hsa_circ_0008812 |
| hsa-miR-6803-5p | hsa_circ_0006174 |
| hsa-miR-6803-5p | hsa_circ_0087861 |
| hsa-miR-6803-5p | hsa_circ_0087862 |
| hsa-miR-6803-5p | hsa_circ_0001947 |
| hsa-miR-6803-3p | hsa_circ_0004699 |
| hsa-miR-6803-3p | hsa_circ_0007132 |
| hsa-miR-6803-3p | hsa_circ_0002094 |
| hsa-miR-6804-3p | hsa_circ_0033144 |
| hsa-miR-6804-3p | hsa_circ_0040921 |
| hsa-miR-6804-3p | hsa_circ_0002805 |
| hsa-miR-6804-3p | hsa_circ_0061179 |
| hsa-miR-6804-3p | hsa_circ_0092299 |
| hsa-miR-6805-5p | hsa_circ_0009142 |
| hsa-miR-6805-5p | hsa_circ_0000375 |
| hsa-miR-6805-5p | hsa_circ_0000417 |
| hsa-miR-6805-5p | hsa_circ_0007846 |
| hsa-miR-6805-5p | hsa_circ_0061052 |
| hsa-miR-6805-5p | hsa_circ_0092299 |
| hsa-miR-6805-5p | hsa_circ_0001472 |
| hsa-miR-6805-5p | hsa_circ_0087288 |
| hsa-miR-6805-5p | hsa_circ_0087305 |
| hsa-miR-6805-3p | hsa_circ_0007846 |
| hsa-miR-6805-3p | hsa_circ_0046430 |
| hsa-miR-6805-3p | hsa_circ_0002805 |
| hsa-miR-6805-3p | hsa_circ_0007132 |
| hsa-miR-6805-3p | hsa_circ_0002094 |
| hsa-miR-6805-3p | hsa_circ_0008113 |
| hsa-miR-6806-5p | hsa_circ_0005782 |
| hsa-miR-6806-5p | hsa_circ_0003557 |
| hsa-miR-6806-5p | hsa_circ_0000375 |
| hsa-miR-6806-5p | hsa_circ_0007146 |
| hsa-miR-6806-5p | hsa_circ_0006735 |
| hsa-miR-6806-5p | hsa_circ_0003854 |
| hsa-miR-6806-5p | hsa_circ_0003602 |
| hsa-miR-6806-5p | hsa_circ_0071869 |
| hsa-miR-6806-5p | hsa_circ_0007132 |
| hsa-miR-6807-5p | hsa_circ_0006649 |
| hsa-miR-6807-3p | hsa_circ_0029405 |
| hsa-miR-6807-3p | hsa_circ_0000973 |
| hsa-miR-6807-3p | hsa_circ_0002077 |
| hsa-miR-6807-3p | hsa_circ_0007132 |
| hsa-miR-6807-3p | hsa_circ_0087288 |
| hsa-miR-6808-5p | hsa_circ_0002884 |
| hsa-miR-6808-5p | hsa_circ_0002264 |
| hsa-miR-6808-5p | hsa_circ_0007846 |
| hsa-miR-6808-5p | hsa_circ_0004519 |
| hsa-miR-6808-5p | hsa_circ_0045890 |
| hsa-miR-6808-5p | hsa_circ_0003854 |
| hsa-miR-6808-5p | hsa_circ_0092299 |
| hsa-miR-6808-5p | hsa_circ_0092277 |
| hsa-miR-6808-5p | hsa_circ_0071311 |
| hsa-miR-6808-5p | hsa_circ_0072857 |
| hsa-miR-6808-5p | hsa_circ_0004381 |
| hsa-miR-6808-5p | hsa_circ_0005630 |
| hsa-miR-6808-5p | hsa_circ_0084789 |
| hsa-miR-6808-3p | hsa_circ_0002094 |
| hsa-miR-6809-3p | hsa_circ_0005630 |
| hsa-miR-6810-5p | hsa_circ_0049998 |
| hsa-miR-6810-5p | hsa_circ_0000918 |
| hsa-miR-6810-3p | hsa_circ_0009732 |
| hsa-miR-6810-3p | hsa_circ_0022723 |
| hsa-miR-6810-3p | hsa_circ_0000395 |
| hsa-miR-6810-3p | hsa_circ_0031933 |
| hsa-miR-6810-3p | hsa_circ_0045890 |
| hsa-miR-6810-3p | hsa_circ_0007132 |
| hsa-miR-6810-3p | hsa_circ_0008113 |
| hsa-miR-6811-3p | hsa_circ_0007372 |
| hsa-miR-6812-5p | hsa_circ_0011422 |
| hsa-miR-6812-5p | hsa_circ_0014132 |
| hsa-miR-6812-5p | hsa_circ_0017289 |
| hsa-miR-6812-5p | hsa_circ_0005090 |
| hsa-miR-6812-5p | hsa_circ_0000220 |
| hsa-miR-6812-5p | hsa_circ_0006665 |
| hsa-miR-6812-5p | hsa_circ_0007778 |
| hsa-miR-6812-5p | hsa_circ_0003379 |
| hsa-miR-6812-5p | hsa_circ_0022383 |
| hsa-miR-6812-5p | hsa_circ_0023918 |
| hsa-miR-6812-5p | hsa_circ_0023920 |
| hsa-miR-6812-5p | hsa_circ_0023923 |
| hsa-miR-6812-5p | hsa_circ_0000375 |
| hsa-miR-6812-5p | hsa_circ_0003855 |
| hsa-miR-6812-5p | hsa_circ_0003489 |
| hsa-miR-6812-5p | hsa_circ_0006597 |
| hsa-miR-6812-5p | hsa_circ_0030051 |
| hsa-miR-6812-5p | hsa_circ_0004137 |
| hsa-miR-6812-5p | hsa_circ_0031933 |
| hsa-miR-6812-5p | hsa_circ_0000565 |
| hsa-miR-6812-5p | hsa_circ_0007846 |
| hsa-miR-6812-5p | hsa_circ_0002696 |
| hsa-miR-6812-5p | hsa_circ_0045890 |
| hsa-miR-6812-5p | hsa_circ_0046430 |
| hsa-miR-6812-5p | hsa_circ_0004003 |
| hsa-miR-6812-5p | hsa_circ_0004891 |
| hsa-miR-6812-5p | hsa_circ_0049998 |
| hsa-miR-6812-5p | hsa_circ_0050119 |
| hsa-miR-6812-5p | hsa_circ_0055904 |
| hsa-miR-6812-5p | hsa_circ_0003854 |
| hsa-miR-6812-5p | hsa_circ_0062397 |
| hsa-miR-6812-5p | hsa_circ_0065284 |
| hsa-miR-6812-5p | hsa_circ_0092277 |
| hsa-miR-6812-5p | hsa_circ_0001400 |
| hsa-miR-6812-5p | hsa_circ_0069748 |
| hsa-miR-6812-5p | hsa_circ_0006693 |
| hsa-miR-6812-5p | hsa_circ_0001417 |
| hsa-miR-6812-5p | hsa_circ_0070467 |
| hsa-miR-6812-5p | hsa_circ_0007132 |
| hsa-miR-6812-5p | hsa_circ_0076179 |
| hsa-miR-6812-5p | hsa_circ_0001658 |
| hsa-miR-6812-5p | hsa_circ_0004381 |
| hsa-miR-6812-5p | hsa_circ_0082179 |
| hsa-miR-6812-5p | hsa_circ_0002094 |
| hsa-miR-6812-5p | hsa_circ_0084429 |
| hsa-miR-6812-5p | hsa_circ_0001806 |
| hsa-miR-6812-5p | hsa_circ_0084789 |
| hsa-miR-6812-5p | hsa_circ_0087305 |
| hsa-miR-6812-3p | hsa_circ_0022723 |
| hsa-miR-6812-3p | hsa_circ_0000996 |
| hsa-miR-6812-3p | hsa_circ_0002805 |
| hsa-miR-6813-5p | hsa_circ_0007778 |
| hsa-miR-6813-5p | hsa_circ_0003557 |
| hsa-miR-6813-5p | hsa_circ_0008521 |
| hsa-miR-6813-5p | hsa_circ_0003848 |
| hsa-miR-6813-5p | hsa_circ_0002564 |
| hsa-miR-6813-5p | hsa_circ_0002289 |
| hsa-miR-6813-5p | hsa_circ_0007846 |
| hsa-miR-6813-5p | hsa_circ_0006735 |
| hsa-miR-6813-5p | hsa_circ_0045890 |
| hsa-miR-6813-5p | hsa_circ_0046430 |
| hsa-miR-6813-5p | hsa_circ_0000973 |
| hsa-miR-6813-5p | hsa_circ_0000996 |
| hsa-miR-6813-5p | hsa_circ_0074371 |
| hsa-miR-6813-5p | hsa_circ_0001549 |
| hsa-miR-6813-5p | hsa_circ_0003340 |
| hsa-miR-6813-5p | hsa_circ_0002094 |
| hsa-miR-6813-5p | hsa_circ_0005630 |
| hsa-miR-6813-5p | hsa_circ_0087305 |
| hsa-miR-6813-3p | hsa_circ_0046430 |
| hsa-miR-6813-3p | hsa_circ_0007967 |
| hsa-miR-6813-3p | hsa_circ_0007132 |
| hsa-miR-6813-3p | hsa_circ_0002755 |
| hsa-miR-6814-5p | hsa_circ_0007001 |
| hsa-miR-6814-5p | hsa_circ_0007132 |
| hsa-miR-6814-3p | hsa_circ_0007846 |
| hsa-miR-6814-3p | hsa_circ_0002094 |
| hsa-miR-6815-5p | hsa_circ_0000417 |
| hsa-miR-6815-5p | hsa_circ_0049998 |
| hsa-miR-6815-5p | hsa_circ_0001658 |
| hsa-miR-6815-5p | hsa_circ_0003340 |
| hsa-miR-6815-5p | hsa_circ_0087305 |
| hsa-miR-6815-3p | hsa_circ_0002570 |
| hsa-miR-6815-3p | hsa_circ_0000497 |
| hsa-miR-6816-5p | hsa_circ_0002402 |
| hsa-miR-6816-5p | hsa_circ_0003168 |
| hsa-miR-6816-5p | hsa_circ_0003557 |
| hsa-miR-6816-5p | hsa_circ_0007372 |
| hsa-miR-6816-5p | hsa_circ_0000375 |
| hsa-miR-6816-5p | hsa_circ_0000417 |
| hsa-miR-6816-5p | hsa_circ_0045890 |
| hsa-miR-6816-5p | hsa_circ_0045905 |
| hsa-miR-6816-5p | hsa_circ_0046430 |
| hsa-miR-6816-5p | hsa_circ_0050119 |
| hsa-miR-6816-5p | hsa_circ_0002805 |
| hsa-miR-6816-5p | hsa_circ_0061179 |
| hsa-miR-6816-5p | hsa_circ_0092299 |
| hsa-miR-6816-5p | hsa_circ_0092277 |
| hsa-miR-6816-5p | hsa_circ_0001472 |
| hsa-miR-6816-5p | hsa_circ_0002094 |
| hsa-miR-6817-5p | hsa_circ_0049998 |
| hsa-miR-6817-5p | hsa_circ_0050119 |
| hsa-miR-6819-5p | hsa_circ_0006649 |
| hsa-miR-6819-5p | hsa_circ_0008102 |
| hsa-miR-6819-5p | hsa_circ_0006254 |
| hsa-miR-6819-5p | hsa_circ_0003768 |
| hsa-miR-6819-5p | hsa_circ_0022383 |
| hsa-miR-6819-5p | hsa_circ_0000339 |
| hsa-miR-6819-5p | hsa_circ_0003908 |
| hsa-miR-6819-5p | hsa_circ_0000417 |
| hsa-miR-6819-5p | hsa_circ_0031933 |
| hsa-miR-6819-5p | hsa_circ_0033144 |
| hsa-miR-6819-5p | hsa_circ_0000651 |
| hsa-miR-6819-5p | hsa_circ_0007846 |
| hsa-miR-6819-5p | hsa_circ_0046430 |
| hsa-miR-6819-5p | hsa_circ_0092297 |
| hsa-miR-6819-5p | hsa_circ_0072437 |
| hsa-miR-6819-5p | hsa_circ_0001577 |
| hsa-miR-6819-5p | hsa_circ_0001578 |
| hsa-miR-6819-5p | hsa_circ_0081873 |
| hsa-miR-6819-5p | hsa_circ_0002094 |
| hsa-miR-6819-5p | hsa_circ_0005630 |
| hsa-miR-6819-5p | hsa_circ_0006566 |
| hsa-miR-6819-5p | hsa_circ_0087855 |
| hsa-miR-6819-5p | hsa_circ_0008812 |
| hsa-miR-6819-5p | hsa_circ_0006174 |
| hsa-miR-6819-5p | hsa_circ_0087861 |
| hsa-miR-6819-5p | hsa_circ_0087862 |
| hsa-miR-6819-3p | hsa_circ_0002805 |
| hsa-miR-6820-5p | hsa_circ_0003278 |
| hsa-miR-6820-3p | hsa_circ_0092299 |
| hsa-miR-6820-3p | hsa_circ_0007132 |
| hsa-miR-6820-3p | hsa_circ_0084429 |
| hsa-miR-6821-5p | hsa_circ_0009581 |
| hsa-miR-6821-5p | hsa_circ_0006837 |
| hsa-miR-6821-5p | hsa_circ_0009732 |
| hsa-miR-6821-5p | hsa_circ_0007015 |
| hsa-miR-6821-5p | hsa_circ_0018909 |
| hsa-miR-6821-5p | hsa_circ_0006649 |
| hsa-miR-6821-5p | hsa_circ_0003379 |
| hsa-miR-6821-5p | hsa_circ_0022723 |
| hsa-miR-6821-5p | hsa_circ_0003557 |
| hsa-miR-6821-5p | hsa_circ_0000417 |
| hsa-miR-6821-5p | hsa_circ_0004137 |
| hsa-miR-6821-5p | hsa_circ_0000660 |
| hsa-miR-6821-5p | hsa_circ_0005733 |
| hsa-miR-6821-5p | hsa_circ_0040039 |
| hsa-miR-6821-5p | hsa_circ_0040823 |
| hsa-miR-6821-5p | hsa_circ_0046430 |
| hsa-miR-6821-5p | hsa_circ_0048025 |
| hsa-miR-6821-5p | hsa_circ_0002926 |
| hsa-miR-6821-5p | hsa_circ_0000909 |
| hsa-miR-6821-5p | hsa_circ_0049998 |
| hsa-miR-6821-5p | hsa_circ_0050119 |
| hsa-miR-6821-5p | hsa_circ_0000950 |
| hsa-miR-6821-5p | hsa_circ_0006006 |
| hsa-miR-6821-5p | hsa_circ_0004218 |
| hsa-miR-6821-5p | hsa_circ_0002805 |
| hsa-miR-6821-5p | hsa_circ_0061179 |
| hsa-miR-6821-5p | hsa_circ_0001187 |
| hsa-miR-6821-5p | hsa_circ_0008078 |
| hsa-miR-6821-5p | hsa_circ_0002077 |
| hsa-miR-6821-5p | hsa_circ_0004705 |
| hsa-miR-6821-5p | hsa_circ_0066444 |
| hsa-miR-6821-5p | hsa_circ_0001400 |
| hsa-miR-6821-5p | hsa_circ_0001472 |
| hsa-miR-6821-5p | hsa_circ_0001573 |
| hsa-miR-6821-5p | hsa_circ_0007132 |
| hsa-miR-6821-5p | hsa_circ_0004381 |
| hsa-miR-6821-5p | hsa_circ_0078522 |
| hsa-miR-6821-5p | hsa_circ_0002094 |
| hsa-miR-6821-5p | hsa_circ_0087855 |
| hsa-miR-6821-5p | hsa_circ_0008812 |
| hsa-miR-6821-5p | hsa_circ_0006174 |
| hsa-miR-6821-5p | hsa_circ_0087861 |
| hsa-miR-6821-5p | hsa_circ_0087862 |
| hsa-miR-6821-3p | hsa_circ_0005630 |
| hsa-miR-6822-5p | hsa_circ_0075748 |
| hsa-miR-6822-3p | hsa_circ_0007132 |
| hsa-miR-6823-5p | hsa_circ_0092297 |
| hsa-miR-6823-3p | hsa_circ_0007145 |
| hsa-miR-6823-3p | hsa_circ_0005630 |
| hsa-miR-6824-5p | hsa_circ_0007015 |
| hsa-miR-6824-5p | hsa_circ_0006649 |
| hsa-miR-6824-5p | hsa_circ_0022383 |
| hsa-miR-6824-5p | hsa_circ_0025388 |
| hsa-miR-6824-5p | hsa_circ_0000417 |
| hsa-miR-6824-5p | hsa_circ_0002696 |
| hsa-miR-6824-5p | hsa_circ_0045890 |
| hsa-miR-6824-5p | hsa_circ_0049657 |
| hsa-miR-6824-5p | hsa_circ_0002805 |
| hsa-miR-6824-5p | hsa_circ_0061179 |
| hsa-miR-6824-5p | hsa_circ_0092299 |
| hsa-miR-6824-5p | hsa_circ_0064136 |
| hsa-miR-6824-5p | hsa_circ_0070467 |
| hsa-miR-6824-5p | hsa_circ_0071311 |
| hsa-miR-6824-5p | hsa_circ_0001549 |
| hsa-miR-6824-5p | hsa_circ_0001577 |
| hsa-miR-6824-5p | hsa_circ_0001578 |
| hsa-miR-6824-5p | hsa_circ_0007132 |
| hsa-miR-6824-5p | hsa_circ_0004381 |
| hsa-miR-6824-5p | hsa_circ_0003340 |
| hsa-miR-6824-5p | hsa_circ_0005630 |
| hsa-miR-6824-5p | hsa_circ_0087305 |
| hsa-miR-6824-5p | hsa_circ_0001866 |
| hsa-miR-6824-5p | hsa_circ_0087855 |
| hsa-miR-6824-5p | hsa_circ_0008812 |
| hsa-miR-6824-5p | hsa_circ_0006174 |
| hsa-miR-6824-5p | hsa_circ_0087861 |
| hsa-miR-6824-5p | hsa_circ_0087862 |
| hsa-miR-6824-3p | hsa_circ_0003379 |
| hsa-miR-6824-3p | hsa_circ_0036763 |
| hsa-miR-6824-3p | hsa_circ_0000650 |
| hsa-miR-6824-3p | hsa_circ_0092283 |
| hsa-miR-6824-3p | hsa_circ_0001658 |
| hsa-miR-6824-3p | hsa_circ_0002094 |
| hsa-miR-6825-5p | hsa_circ_0045890 |
| hsa-miR-6825-5p | hsa_circ_0049657 |
| hsa-miR-6825-5p | hsa_circ_0000918 |
| hsa-miR-6825-5p | hsa_circ_0000973 |
| hsa-miR-6825-5p | hsa_circ_0003854 |
| hsa-miR-6825-5p | hsa_circ_0001400 |
| hsa-miR-6825-5p | hsa_circ_0001549 |
| hsa-miR-6825-5p | hsa_circ_0004381 |
| hsa-miR-6825-5p | hsa_circ_0087288 |
| hsa-miR-6825-5p | hsa_circ_0087305 |
| hsa-miR-6825-3p | hsa_circ_0007146 |
| hsa-miR-6825-3p | hsa_circ_0000936 |
| hsa-miR-6825-3p | hsa_circ_0007132 |
| hsa-miR-6826-5p | hsa_circ_0004381 |
| hsa-miR-6826-3p | hsa_circ_0004692 |
| hsa-miR-6827-5p | hsa_circ_0004008 |
| hsa-miR-6827-5p | hsa_circ_0046292 |
| hsa-miR-6827-5p | hsa_circ_0092297 |
| hsa-miR-6827-5p | hsa_circ_0002113 |
| hsa-miR-6827-5p | hsa_circ_0074371 |
| hsa-miR-6827-3p | hsa_circ_0031933 |
| hsa-miR-6829-5p | hsa_circ_0005782 |
| hsa-miR-6829-5p | hsa_circ_0004470 |
| hsa-miR-6829-5p | hsa_circ_0084429 |
| hsa-miR-6829-5p | hsa_circ_0001811 |
| hsa-miR-6829-3p | hsa_circ_0000463 |
| hsa-miR-6829-3p | hsa_circ_0045890 |
| hsa-miR-6829-3p | hsa_circ_0049998 |
| hsa-miR-6829-3p | hsa_circ_0092283 |
| hsa-miR-6831-5p | hsa_circ_0006649 |
| hsa-miR-6831-5p | hsa_circ_0000417 |
| hsa-miR-6831-5p | hsa_circ_0000936 |
| hsa-miR-6831-5p | hsa_circ_0069748 |
| hsa-miR-6831-5p | hsa_circ_0001472 |
| hsa-miR-6833-5p | hsa_circ_0002805 |
| hsa-miR-6833-5p | hsa_circ_0069748 |
| hsa-miR-6834-5p | hsa_circ_0000918 |
| hsa-miR-6780b-5p | hsa_circ_0000220 |
| hsa-miR-6780b-5p | hsa_circ_0006665 |
| hsa-miR-6780b-5p | hsa_circ_0007846 |
| hsa-miR-6780b-5p | hsa_circ_0049657 |
| hsa-miR-6780b-5p | hsa_circ_0005571 |
| hsa-miR-6780b-5p | hsa_circ_0000918 |
| hsa-miR-6780b-5p | hsa_circ_0003854 |
| hsa-miR-6780b-5p | hsa_circ_0065284 |
| hsa-miR-6780b-5p | hsa_circ_0007132 |
| hsa-miR-6780b-5p | hsa_circ_0003738 |
| hsa-miR-6780b-5p | hsa_circ_0001811 |
| hsa-miR-6780b-3p | hsa_circ_0007967 |
| hsa-miR-6836-5p | hsa_circ_0005782 |
| hsa-miR-6836-5p | hsa_circ_0000131 |
| hsa-miR-6836-5p | hsa_circ_0016979 |
| hsa-miR-6836-5p | hsa_circ_0003557 |
| hsa-miR-6836-5p | hsa_circ_0004519 |
| hsa-miR-6836-5p | hsa_circ_0040823 |
| hsa-miR-6836-5p | hsa_circ_0000909 |
| hsa-miR-6836-5p | hsa_circ_0092297 |
| hsa-miR-6836-5p | hsa_circ_0092299 |
| hsa-miR-6836-5p | hsa_circ_0003602 |
| hsa-miR-6836-5p | hsa_circ_0007132 |
| hsa-miR-6836-5p | hsa_circ_0005630 |
| hsa-miR-6836-3p | hsa_circ_0003379 |
| hsa-miR-6836-3p | hsa_circ_0045890 |
| hsa-miR-6836-3p | hsa_circ_0092297 |
| hsa-miR-6836-3p | hsa_circ_0070396 |
| hsa-miR-6836-3p | hsa_circ_0007132 |
| hsa-miR-6836-3p | hsa_circ_0001829 |
| hsa-miR-6837-5p | hsa_circ_0002289 |
| hsa-miR-6837-5p | hsa_circ_0092297 |
| hsa-miR-6837-3p | hsa_circ_0046430 |
| hsa-miR-6839-3p | hsa_circ_0002805 |
| hsa-miR-6840-5p | hsa_circ_0006693 |
| hsa-miR-6840-5p | hsa_circ_0001417 |
| hsa-miR-6840-5p | hsa_circ_0007132 |
| hsa-miR-6840-5p | hsa_circ_0001658 |
| hsa-miR-6840-5p | hsa_circ_0092125 |
| hsa-miR-6840-3p | hsa_circ_0022383 |
| hsa-miR-6840-3p | hsa_circ_0007846 |
| hsa-miR-6840-3p | hsa_circ_0092299 |
| hsa-miR-6842-5p | hsa_circ_0007437 |
| hsa-miR-6842-5p | hsa_circ_0007693 |
| hsa-miR-6842-5p | hsa_circ_0042799 |
| hsa-miR-6842-5p | hsa_circ_0000950 |
| hsa-miR-6842-5p | hsa_circ_0092297 |
| hsa-miR-6842-5p | hsa_circ_0008078 |
| hsa-miR-6842-5p | hsa_circ_0006936 |
| hsa-miR-6842-5p | hsa_circ_0081423 |
| hsa-miR-6842-3p | hsa_circ_0000690 |
| hsa-miR-6842-3p | hsa_circ_0064136 |
| hsa-miR-6843-3p | hsa_circ_0049888 |
| hsa-miR-6843-3p | hsa_circ_0072437 |
| hsa-miR-6845-5p | hsa_circ_0092299 |
| hsa-miR-6845-3p | hsa_circ_0006597 |
| hsa-miR-6845-3p | hsa_circ_0040921 |
| hsa-miR-6845-3p | hsa_circ_0000996 |
| hsa-miR-6845-3p | hsa_circ_0002805 |
| hsa-miR-6845-3p | hsa_circ_0092283 |
| hsa-miR-6845-3p | hsa_circ_0006528 |
| hsa-miR-6845-3p | hsa_circ_0007132 |
| hsa-miR-6846-5p | hsa_circ_0002402 |
| hsa-miR-6846-5p | hsa_circ_0017248 |
| hsa-miR-6846-5p | hsa_circ_0006254 |
| hsa-miR-6846-5p | hsa_circ_0003768 |
| hsa-miR-6846-5p | hsa_circ_0002884 |
| hsa-miR-6846-5p | hsa_circ_0002264 |
| hsa-miR-6846-5p | hsa_circ_0000417 |
| hsa-miR-6846-5p | hsa_circ_0000497 |
| hsa-miR-6846-5p | hsa_circ_0007846 |
| hsa-miR-6846-5p | hsa_circ_0040921 |
| hsa-miR-6846-5p | hsa_circ_0046430 |
| hsa-miR-6846-5p | hsa_circ_0048025 |
| hsa-miR-6846-5p | hsa_circ_0006877 |
| hsa-miR-6846-5p | hsa_circ_0002805 |
| hsa-miR-6846-5p | hsa_circ_0061179 |
| hsa-miR-6846-5p | hsa_circ_0001394 |
| hsa-miR-6846-5p | hsa_circ_0070040 |
| hsa-miR-6846-5p | hsa_circ_0001806 |
| hsa-miR-6846-5p | hsa_circ_0087855 |
| hsa-miR-6846-5p | hsa_circ_0008812 |
| hsa-miR-6846-5p | hsa_circ_0006174 |
| hsa-miR-6846-5p | hsa_circ_0087861 |
| hsa-miR-6846-5p | hsa_circ_0087862 |
| hsa-miR-6846-3p | hsa_circ_0020594 |
| hsa-miR-6846-3p | hsa_circ_0000395 |
| hsa-miR-6846-3p | hsa_circ_0007132 |
| hsa-miR-6847-5p | hsa_circ_0020594 |
| hsa-miR-6847-3p | hsa_circ_0076995 |
| hsa-miR-6848-5p | hsa_circ_0002402 |
| hsa-miR-6848-5p | hsa_circ_0009142 |
| hsa-miR-6848-5p | hsa_circ_0007437 |
| hsa-miR-6848-5p | hsa_circ_0007693 |
| hsa-miR-6848-5p | hsa_circ_0012151 |
| hsa-miR-6848-5p | hsa_circ_0000130 |
| hsa-miR-6848-5p | hsa_circ_0000131 |
| hsa-miR-6848-5p | hsa_circ_0015004 |
| hsa-miR-6848-5p | hsa_circ_0017289 |
| hsa-miR-6848-5p | hsa_circ_0006254 |
| hsa-miR-6848-5p | hsa_circ_0003768 |
| hsa-miR-6848-5p | hsa_circ_0002884 |
| hsa-miR-6848-5p | hsa_circ_0002264 |
| hsa-miR-6848-5p | hsa_circ_0000417 |
| hsa-miR-6848-5p | hsa_circ_0028190 |
| hsa-miR-6848-5p | hsa_circ_0029405 |
| hsa-miR-6848-5p | hsa_circ_0029633 |
| hsa-miR-6848-5p | hsa_circ_0000497 |
| hsa-miR-6848-5p | hsa_circ_0031933 |
| hsa-miR-6848-5p | hsa_circ_0007146 |
| hsa-miR-6848-5p | hsa_circ_0007846 |
| hsa-miR-6848-5p | hsa_circ_0006735 |
| hsa-miR-6848-5p | hsa_circ_0005733 |
| hsa-miR-6848-5p | hsa_circ_0040039 |
| hsa-miR-6848-5p | hsa_circ_0042799 |
| hsa-miR-6848-5p | hsa_circ_0045890 |
| hsa-miR-6848-5p | hsa_circ_0046430 |
| hsa-miR-6848-5p | hsa_circ_0000836 |
| hsa-miR-6848-5p | hsa_circ_0000909 |
| hsa-miR-6848-5p | hsa_circ_0050119 |
| hsa-miR-6848-5p | hsa_circ_0000918 |
| hsa-miR-6848-5p | hsa_circ_0000950 |
| hsa-miR-6848-5p | hsa_circ_0003854 |
| hsa-miR-6848-5p | hsa_circ_0004218 |
| hsa-miR-6848-5p | hsa_circ_0092299 |
| hsa-miR-6848-5p | hsa_circ_0064136 |
| hsa-miR-6848-5p | hsa_circ_0004692 |
| hsa-miR-6848-5p | hsa_circ_0004276 |
| hsa-miR-6848-5p | hsa_circ_0070040 |
| hsa-miR-6848-5p | hsa_circ_0071311 |
| hsa-miR-6848-5p | hsa_circ_0001573 |
| hsa-miR-6848-5p | hsa_circ_0007132 |
| hsa-miR-6848-5p | hsa_circ_0003340 |
| hsa-miR-6848-5p | hsa_circ_0001708 |
| hsa-miR-6848-5p | hsa_circ_0002094 |
| hsa-miR-6848-5p | hsa_circ_0087305 |
| hsa-miR-6848-5p | hsa_circ_0087855 |
| hsa-miR-6848-5p | hsa_circ_0008812 |
| hsa-miR-6848-5p | hsa_circ_0006174 |
| hsa-miR-6848-5p | hsa_circ_0087861 |
| hsa-miR-6848-5p | hsa_circ_0087862 |
| hsa-miR-6849-5p | hsa_circ_0008102 |
| hsa-miR-6849-5p | hsa_circ_0022723 |
| hsa-miR-6849-5p | hsa_circ_0000375 |
| hsa-miR-6849-5p | hsa_circ_0044195 |
| hsa-miR-6849-5p | hsa_circ_0046430 |
| hsa-miR-6849-5p | hsa_circ_0049657 |
| hsa-miR-6849-5p | hsa_circ_0004705 |
| hsa-miR-6849-5p | hsa_circ_0070467 |
| hsa-miR-6849-5p | hsa_circ_0001577 |
| hsa-miR-6849-5p | hsa_circ_0004381 |
| hsa-miR-6849-3p | hsa_circ_0011571 |
| hsa-miR-6849-3p | hsa_circ_0011572 |
| hsa-miR-6849-3p | hsa_circ_0007437 |
| hsa-miR-6849-3p | hsa_circ_0007693 |
| hsa-miR-6849-3p | hsa_circ_0023918 |
| hsa-miR-6849-3p | hsa_circ_0023920 |
| hsa-miR-6849-3p | hsa_circ_0023923 |
| hsa-miR-6849-3p | hsa_circ_0035944 |
| hsa-miR-6849-3p | hsa_circ_0044195 |
| hsa-miR-6849-3p | hsa_circ_0092283 |
| hsa-miR-6849-3p | hsa_circ_0003602 |
| hsa-miR-6849-3p | hsa_circ_0007132 |
| hsa-miR-6849-3p | hsa_circ_0001658 |
| hsa-miR-6850-5p | hsa_circ_0000417 |
| hsa-miR-6850-5p | hsa_circ_0040921 |
| hsa-miR-6850-5p | hsa_circ_0048025 |
| hsa-miR-6850-5p | hsa_circ_0050119 |
| hsa-miR-6850-5p | hsa_circ_0008261 |
| hsa-miR-6850-5p | hsa_circ_0092299 |
| hsa-miR-6850-5p | hsa_circ_0001394 |
| hsa-miR-6850-5p | hsa_circ_0072857 |
| hsa-miR-6850-5p | hsa_circ_0007132 |
| hsa-miR-6851-5p | hsa_circ_0007372 |
| hsa-miR-6851-5p | hsa_circ_0000417 |
| hsa-miR-6851-5p | hsa_circ_0007846 |
| hsa-miR-6851-5p | hsa_circ_0045890 |
| hsa-miR-6851-5p | hsa_circ_0045905 |
| hsa-miR-6851-5p | hsa_circ_0046430 |
| hsa-miR-6851-5p | hsa_circ_0000973 |
| hsa-miR-6851-5p | hsa_circ_0058522 |
| hsa-miR-6851-5p | hsa_circ_0002805 |
| hsa-miR-6851-5p | hsa_circ_0061179 |
| hsa-miR-6851-5p | hsa_circ_0092299 |
| hsa-miR-6851-5p | hsa_circ_0004470 |
| hsa-miR-6851-5p | hsa_circ_0001247 |
| hsa-miR-6851-5p | hsa_circ_0064136 |
| hsa-miR-6851-5p | hsa_circ_0001472 |
| hsa-miR-6851-5p | hsa_circ_0008536 |
| hsa-miR-6851-5p | hsa_circ_0077765 |
| hsa-miR-6851-5p | hsa_circ_0001866 |
| hsa-miR-6851-3p | hsa_circ_0020594 |
| hsa-miR-6851-3p | hsa_circ_0048025 |
| hsa-miR-6851-3p | hsa_circ_0092283 |
| hsa-miR-6852-5p | hsa_circ_0000131 |
| hsa-miR-6854-3p | hsa_circ_0092299 |
| hsa-miR-6855-5p | hsa_circ_0000660 |
| hsa-miR-6855-5p | hsa_circ_0006735 |
| hsa-miR-6855-5p | hsa_circ_0043691 |
| hsa-miR-6855-5p | hsa_circ_0008030 |
| hsa-miR-6855-5p | hsa_circ_0000936 |
| hsa-miR-6855-5p | hsa_circ_0000973 |
| hsa-miR-6855-5p | hsa_circ_0003602 |
| hsa-miR-6855-5p | hsa_circ_0079480 |
| hsa-miR-6855-5p | hsa_circ_0005630 |
| hsa-miR-6855-5p | hsa_circ_0087305 |
| hsa-miR-6855-5p | hsa_circ_0001947 |
| hsa-miR-6856-5p | hsa_circ_0058522 |
| hsa-miR-6856-5p | hsa_circ_0001400 |
| hsa-miR-6856-5p | hsa_circ_0007132 |
| hsa-miR-6856-3p | hsa_circ_0001400 |
| hsa-miR-6857-5p | hsa_circ_0002402 |
| hsa-miR-6857-5p | hsa_circ_0000836 |
| hsa-miR-6857-5p | hsa_circ_0002926 |
| hsa-miR-6857-5p | hsa_circ_0008261 |
| hsa-miR-6857-5p | hsa_circ_0060043 |
| hsa-miR-6857-5p | hsa_circ_0002805 |
| hsa-miR-6857-5p | hsa_circ_0061179 |
| hsa-miR-6857-5p | hsa_circ_0006693 |
| hsa-miR-6857-5p | hsa_circ_0001417 |
| hsa-miR-6857-5p | hsa_circ_0005630 |
| hsa-miR-6858-5p | hsa_circ_0009349 |
| hsa-miR-6858-5p | hsa_circ_0002402 |
| hsa-miR-6858-5p | hsa_circ_0006649 |
| hsa-miR-6858-5p | hsa_circ_0003379 |
| hsa-miR-6858-5p | hsa_circ_0023918 |
| hsa-miR-6858-5p | hsa_circ_0023923 |
| hsa-miR-6858-5p | hsa_circ_0031933 |
| hsa-miR-6858-5p | hsa_circ_0040823 |
| hsa-miR-6858-5p | hsa_circ_0046430 |
| hsa-miR-6858-5p | hsa_circ_0000909 |
| hsa-miR-6858-5p | hsa_circ_0000918 |
| hsa-miR-6858-5p | hsa_circ_0052131 |
| hsa-miR-6858-5p | hsa_circ_0008261 |
| hsa-miR-6858-5p | hsa_circ_0003854 |
| hsa-miR-6858-5p | hsa_circ_0004218 |
| hsa-miR-6858-5p | hsa_circ_0002805 |
| hsa-miR-6858-5p | hsa_circ_0092277 |
| hsa-miR-6858-5p | hsa_circ_0001472 |
| hsa-miR-6858-5p | hsa_circ_0001577 |
| hsa-miR-6858-5p | hsa_circ_0007132 |
| hsa-miR-6858-5p | hsa_circ_0004712 |
| hsa-miR-6858-5p | hsa_circ_0001658 |
| hsa-miR-6858-5p | hsa_circ_0084429 |
| hsa-miR-6858-5p | hsa_circ_0092125 |
| hsa-miR-6858-3p | hsa_circ_0016979 |
| hsa-miR-6858-3p | hsa_circ_0003838 |
| hsa-miR-6858-3p | hsa_circ_0007146 |
| hsa-miR-6858-3p | hsa_circ_0092299 |
| hsa-miR-6858-3p | hsa_circ_0092283 |
| hsa-miR-6858-3p | hsa_circ_0007132 |
| hsa-miR-6859-5p | hsa_circ_0005325 |
| hsa-miR-6769b-5p | hsa_circ_0007015 |
| hsa-miR-6769b-5p | hsa_circ_0008102 |
| hsa-miR-6769b-5p | hsa_circ_0020005 |
| hsa-miR-6769b-5p | hsa_circ_0022383 |
| hsa-miR-6769b-5p | hsa_circ_0000375 |
| hsa-miR-6769b-5p | hsa_circ_0000417 |
| hsa-miR-6769b-5p | hsa_circ_0002696 |
| hsa-miR-6769b-5p | hsa_circ_0092337 |
| hsa-miR-6769b-5p | hsa_circ_0049657 |
| hsa-miR-6769b-5p | hsa_circ_0002805 |
| hsa-miR-6769b-5p | hsa_circ_0001187 |
| hsa-miR-6769b-5p | hsa_circ_0008078 |
| hsa-miR-6769b-5p | hsa_circ_0092299 |
| hsa-miR-6769b-5p | hsa_circ_0004470 |
| hsa-miR-6769b-5p | hsa_circ_0064136 |
| hsa-miR-6769b-5p | hsa_circ_0004705 |
| hsa-miR-6769b-5p | hsa_circ_0069748 |
| hsa-miR-6769b-5p | hsa_circ_0007132 |
| hsa-miR-6769b-5p | hsa_circ_0005630 |
| hsa-miR-6769b-5p | hsa_circ_0006566 |
| hsa-miR-6769b-5p | hsa_circ_0087305 |
| hsa-miR-6860 | hsa_circ_0006649 |
| hsa-miR-6860 | hsa_circ_0022383 |
| hsa-miR-6860 | hsa_circ_0044195 |
| hsa-miR-6860 | hsa_circ_0046430 |
| hsa-miR-6860 | hsa_circ_0003854 |
| hsa-miR-6860 | hsa_circ_0002954 |
| hsa-miR-6860 | hsa_circ_0007132 |
| hsa-miR-6860 | hsa_circ_0002755 |
| hsa-miR-6860 | hsa_circ_0005630 |
| hsa-miR-6860 | hsa_circ_0084789 |
| hsa-miR-6860 | hsa_circ_0092125 |
| hsa-miR-6861-5p | hsa_circ_0006608 |
| hsa-miR-6861-5p | hsa_circ_0000497 |
| hsa-miR-6861-5p | hsa_circ_0031933 |
| hsa-miR-6861-5p | hsa_circ_0000592 |
| hsa-miR-6861-5p | hsa_circ_0034972 |
| hsa-miR-6861-5p | hsa_circ_0007146 |
| hsa-miR-6861-5p | hsa_circ_0007846 |
| hsa-miR-6861-5p | hsa_circ_0061052 |
| hsa-miR-6861-5p | hsa_circ_0003340 |
| hsa-miR-6861-3p | hsa_circ_0003768 |
| hsa-miR-6861-3p | hsa_circ_0007132 |
| hsa-miR-6862-5p | hsa_circ_0020005 |
| hsa-miR-6862-5p | hsa_circ_0092277 |
| hsa-miR-6862-3p | hsa_circ_0009349 |
| hsa-miR-6862-3p | hsa_circ_0002402 |
| hsa-miR-6862-3p | hsa_circ_0020594 |
| hsa-miR-6862-3p | hsa_circ_0030051 |
| hsa-miR-6862-3p | hsa_circ_0000996 |
| hsa-miR-6862-3p | hsa_circ_0092299 |
| hsa-miR-6862-3p | hsa_circ_0092283 |
| hsa-miR-6862-3p | hsa_circ_0070396 |
| hsa-miR-6862-3p | hsa_circ_0070467 |
| hsa-miR-6862-3p | hsa_circ_0007132 |
| hsa-miR-6865-5p | hsa_circ_0006649 |
| hsa-miR-6865-5p | hsa_circ_0002805 |
| hsa-miR-6865-5p | hsa_circ_0002490 |
| hsa-miR-6865-5p | hsa_circ_0005630 |
| hsa-miR-6865-5p | hsa_circ_0001824 |
| hsa-miR-6865-5p | hsa_circ_0008934 |
| hsa-miR-6865-5p | hsa_circ_0001866 |
| hsa-miR-6867-3p | hsa_circ_0044195 |
| hsa-miR-6868-5p | hsa_circ_0038799 |
| hsa-miR-6869-5p | hsa_circ_0003557 |
| hsa-miR-6869-5p | hsa_circ_0007846 |
| hsa-miR-6869-5p | hsa_circ_0000682 |
| hsa-miR-6869-5p | hsa_circ_0002805 |
| hsa-miR-6869-5p | hsa_circ_0061179 |
| hsa-miR-6869-5p | hsa_circ_0007132 |
| hsa-miR-6869-5p | hsa_circ_0002755 |
| hsa-miR-6869-5p | hsa_circ_0002094 |
| hsa-miR-6869-5p | hsa_circ_0001910 |
| hsa-miR-6869-3p | hsa_circ_0045890 |
| hsa-miR-6869-3p | hsa_circ_0081343 |
| hsa-miR-6869-3p | hsa_circ_0087305 |
| hsa-miR-6870-5p | hsa_circ_0009732 |
| hsa-miR-6870-5p | hsa_circ_0007846 |
| hsa-miR-6870-5p | hsa_circ_0046430 |
| hsa-miR-6870-5p | hsa_circ_0002926 |
| hsa-miR-6870-5p | hsa_circ_0006877 |
| hsa-miR-6870-5p | hsa_circ_0000973 |
| hsa-miR-6871-5p | hsa_circ_0046430 |
| hsa-miR-6871-5p | hsa_circ_0052131 |
| hsa-miR-6871-5p | hsa_circ_0002077 |
| hsa-miR-6871-5p | hsa_circ_0064136 |
| hsa-miR-6871-5p | hsa_circ_0001811 |
| hsa-miR-6871-5p | hsa_circ_0084789 |
| hsa-miR-6871-3p | hsa_circ_0000395 |
| hsa-miR-6871-3p | hsa_circ_0004519 |
| hsa-miR-6871-3p | hsa_circ_0002926 |
| hsa-miR-6871-3p | hsa_circ_0092299 |
| hsa-miR-6871-3p | hsa_circ_0006528 |
| hsa-miR-6871-3p | hsa_circ_0007132 |
| hsa-miR-6872-3p | hsa_circ_0007146 |
| hsa-miR-6872-3p | hsa_circ_0002926 |
| hsa-miR-6872-3p | hsa_circ_0050119 |
| hsa-miR-6872-3p | hsa_circ_0092297 |
| hsa-miR-6872-3p | hsa_circ_0092283 |
| hsa-miR-6872-3p | hsa_circ_0007132 |
| hsa-miR-6872-3p | hsa_circ_0001829 |
| hsa-miR-6873-3p | hsa_circ_0000996 |
| hsa-miR-6873-3p | hsa_circ_0092299 |
| hsa-miR-6874-5p | hsa_circ_0008102 |
| hsa-miR-6874-3p | hsa_circ_0064136 |
| hsa-miR-6875-5p | hsa_circ_0001866 |
| hsa-miR-6875-3p | hsa_circ_0020594 |
| hsa-miR-6875-3p | hsa_circ_0007132 |
| hsa-miR-6876-3p | hsa_circ_0048025 |
| hsa-miR-6876-3p | hsa_circ_0002805 |
| hsa-miR-6877-5p | hsa_circ_0004008 |
| hsa-miR-6877-5p | hsa_circ_0040921 |
| hsa-miR-6877-5p | hsa_circ_0048025 |
| hsa-miR-6877-5p | hsa_circ_0092297 |
| hsa-miR-6877-5p | hsa_circ_0002077 |
| hsa-miR-6877-5p | hsa_circ_0087288 |
| hsa-miR-6877-5p | hsa_circ_0087305 |
| hsa-miR-6877-3p | hsa_circ_0011571 |
| hsa-miR-6877-3p | hsa_circ_0011572 |
| hsa-miR-6877-3p | hsa_circ_0020594 |
| hsa-miR-6877-3p | hsa_circ_0000417 |
| hsa-miR-6877-3p | hsa_circ_0000973 |
| hsa-miR-6877-3p | hsa_circ_0092299 |
| hsa-miR-6877-3p | hsa_circ_0004692 |
| hsa-miR-6877-3p | hsa_circ_0065147 |
| hsa-miR-6877-3p | hsa_circ_0001658 |
| hsa-miR-6877-3p | hsa_circ_0085923 |
| hsa-miR-6879-5p | hsa_circ_0017289 |
| hsa-miR-6879-5p | hsa_circ_0005090 |
| hsa-miR-6879-5p | hsa_circ_0003379 |
| hsa-miR-6879-5p | hsa_circ_0022383 |
| hsa-miR-6879-5p | hsa_circ_0003557 |
| hsa-miR-6879-5p | hsa_circ_0045890 |
| hsa-miR-6879-5p | hsa_circ_0004705 |
| hsa-miR-6879-5p | hsa_circ_0007137 |
| hsa-miR-6879-5p | hsa_circ_0071311 |
| hsa-miR-6879-5p | hsa_circ_0001472 |
| hsa-miR-6879-5p | hsa_circ_0072437 |
| hsa-miR-6879-5p | hsa_circ_0007132 |
| hsa-miR-6879-5p | hsa_circ_0076742 |
| hsa-miR-6879-5p | hsa_circ_0004381 |
| hsa-miR-6879-5p | hsa_circ_0001806 |
| hsa-miR-6879-5p | hsa_circ_0084789 |
| hsa-miR-6879-5p | hsa_circ_0087305 |
| hsa-miR-6879-5p | hsa_circ_0087855 |
| hsa-miR-6879-5p | hsa_circ_0008812 |
| hsa-miR-6879-5p | hsa_circ_0006174 |
| hsa-miR-6879-5p | hsa_circ_0087861 |
| hsa-miR-6879-5p | hsa_circ_0087862 |
| hsa-miR-6879-3p | hsa_circ_0022723 |
| hsa-miR-6879-3p | hsa_circ_0000996 |
| hsa-miR-6879-3p | hsa_circ_0001258 |
| hsa-miR-6879-3p | hsa_circ_0007132 |
| hsa-miR-6880-5p | hsa_circ_0009581 |
| hsa-miR-6880-5p | hsa_circ_0006837 |
| hsa-miR-6880-5p | hsa_circ_0000417 |
| hsa-miR-6880-5p | hsa_circ_0004712 |
| hsa-miR-6880-5p | hsa_circ_0005630 |
| hsa-miR-6880-5p | hsa_circ_0087305 |
| hsa-miR-6880-3p | hsa_circ_0020594 |
| hsa-miR-6880-3p | hsa_circ_0002805 |
| hsa-miR-6880-3p | hsa_circ_0004692 |
| hsa-miR-6880-3p | hsa_circ_0065147 |
| hsa-miR-6880-3p | hsa_circ_0065149 |
| hsa-miR-6880-3p | hsa_circ_0002569 |
| hsa-miR-6880-3p | hsa_circ_0007132 |
| hsa-miR-6880-3p | hsa_circ_0002451 |
| hsa-miR-6880-3p | hsa_circ_0005630 |
| hsa-miR-6881-5p | hsa_circ_0014132 |
| hsa-miR-6881-5p | hsa_circ_0007846 |
| hsa-miR-6881-5p | hsa_circ_0043278 |
| hsa-miR-6881-5p | hsa_circ_0001748 |
| hsa-miR-6881-5p | hsa_circ_0002094 |
| hsa-miR-6881-3p | hsa_circ_0002094 |
| hsa-miR-6882-3p | hsa_circ_0011571 |
| hsa-miR-6882-3p | hsa_circ_0011572 |
| hsa-miR-6882-3p | hsa_circ_0007001 |
| hsa-miR-6882-3p | hsa_circ_0033144 |
| hsa-miR-6882-3p | hsa_circ_0092299 |
| hsa-miR-6882-3p | hsa_circ_0092283 |
| hsa-miR-6882-3p | hsa_circ_0004692 |
| hsa-miR-6882-3p | hsa_circ_0007132 |
| hsa-miR-6882-3p | hsa_circ_0001658 |
| hsa-miR-6882-3p | hsa_circ_0002755 |
| hsa-miR-6882-3p | hsa_circ_0002094 |
| hsa-miR-6882-3p | hsa_circ_0001910 |
| hsa-miR-6883-3p | hsa_circ_0022505 |
| hsa-miR-6884-3p | hsa_circ_0007132 |
| hsa-miR-6885-5p | hsa_circ_0015004 |
| hsa-miR-6885-5p | hsa_circ_0002884 |
| hsa-miR-6885-5p | hsa_circ_0007146 |
| hsa-miR-6886-5p | hsa_circ_0004218 |
| hsa-miR-6886-3p | hsa_circ_0004692 |
| hsa-miR-6887-5p | hsa_circ_0015004 |
| hsa-miR-6887-5p | hsa_circ_0000417 |
| hsa-miR-6887-5p | hsa_circ_0004137 |
| hsa-miR-6887-5p | hsa_circ_0004003 |
| hsa-miR-6887-5p | hsa_circ_0092299 |
| hsa-miR-6887-5p | hsa_circ_0008583 |
| hsa-miR-6887-5p | hsa_circ_0069748 |
| hsa-miR-6887-5p | hsa_circ_0072437 |
| hsa-miR-6887-5p | hsa_circ_0005630 |
| hsa-miR-6887-5p | hsa_circ_0001806 |
| hsa-miR-6887-3p | hsa_circ_0004692 |
| hsa-miR-6887-3p | hsa_circ_0007132 |
| hsa-miR-6889-5p | hsa_circ_0014132 |
| hsa-miR-6889-5p | hsa_circ_0006254 |
| hsa-miR-6889-5p | hsa_circ_0003768 |
| hsa-miR-6889-5p | hsa_circ_0000375 |
| hsa-miR-6889-5p | hsa_circ_0029410 |
| hsa-miR-6889-5p | hsa_circ_0031933 |
| hsa-miR-6889-5p | hsa_circ_0002564 |
| hsa-miR-6889-5p | hsa_circ_0002289 |
| hsa-miR-6889-5p | hsa_circ_0033144 |
| hsa-miR-6889-5p | hsa_circ_0007146 |
| hsa-miR-6889-5p | hsa_circ_0002696 |
| hsa-miR-6889-5p | hsa_circ_0045890 |
| hsa-miR-6889-5p | hsa_circ_0004003 |
| hsa-miR-6889-5p | hsa_circ_0004891 |
| hsa-miR-6889-5p | hsa_circ_0049998 |
| hsa-miR-6889-5p | hsa_circ_0003854 |
| hsa-miR-6889-5p | hsa_circ_0092299 |
| hsa-miR-6889-5p | hsa_circ_0003602 |
| hsa-miR-6889-5p | hsa_circ_0066444 |
| hsa-miR-6889-5p | hsa_circ_0001400 |
| hsa-miR-6889-5p | hsa_circ_0072797 |
| hsa-miR-6889-5p | hsa_circ_0007132 |
| hsa-miR-6889-5p | hsa_circ_0078522 |
| hsa-miR-6889-5p | hsa_circ_0003340 |
| hsa-miR-6889-5p | hsa_circ_0081343 |
| hsa-miR-6889-5p | hsa_circ_0002094 |
| hsa-miR-6889-5p | hsa_circ_0005630 |
| hsa-miR-6889-5p | hsa_circ_0084789 |
| hsa-miR-6889-5p | hsa_circ_0087305 |
| hsa-miR-6889-5p | hsa_circ_0001947 |
| hsa-miR-6889-3p | hsa_circ_0011571 |
| hsa-miR-6889-3p | hsa_circ_0011572 |
| hsa-miR-6890-5p | hsa_circ_0046430 |
| hsa-miR-6890-5p | hsa_circ_0050119 |
| hsa-miR-6890-3p | hsa_circ_0003379 |
| hsa-miR-6891-5p | hsa_circ_0002037 |
| hsa-miR-6891-3p | hsa_circ_0005630 |
| hsa-miR-6892-3p | hsa_circ_0002805 |
| hsa-miR-6892-3p | hsa_circ_0004692 |
| hsa-miR-6892-3p | hsa_circ_0065147 |
| hsa-miR-6892-3p | hsa_circ_0065149 |
| hsa-miR-6892-3p | hsa_circ_0002569 |
| hsa-miR-6892-3p | hsa_circ_0007132 |
| hsa-miR-6893-5p | hsa_circ_0003379 |
| hsa-miR-6893-5p | hsa_circ_0007846 |
| hsa-miR-6893-5p | hsa_circ_0003315 |
| hsa-miR-6893-5p | hsa_circ_0003738 |
| hsa-miR-6893-5p | hsa_circ_0004712 |
| hsa-miR-6893-5p | hsa_circ_0087855 |
| hsa-miR-6893-5p | hsa_circ_0008812 |
| hsa-miR-6893-5p | hsa_circ_0006174 |
| hsa-miR-6893-5p | hsa_circ_0087861 |
| hsa-miR-6893-5p | hsa_circ_0087862 |
| hsa-miR-6893-3p | hsa_circ_0000131 |
| hsa-miR-6893-3p | hsa_circ_0007146 |
| hsa-miR-6893-3p | hsa_circ_0092297 |
| hsa-miR-6893-3p | hsa_circ_0002805 |
| hsa-miR-6893-3p | hsa_circ_0092299 |
| hsa-miR-6893-3p | hsa_circ_0002094 |
| hsa-miR-6894-5p | hsa_circ_0000247 |
| hsa-miR-6894-5p | hsa_circ_0000417 |
| hsa-miR-6894-5p | hsa_circ_0040039 |
| hsa-miR-6894-5p | hsa_circ_0004354 |
| hsa-miR-6894-5p | hsa_circ_0002926 |
| hsa-miR-6894-5p | hsa_circ_0049657 |
| hsa-miR-6894-5p | hsa_circ_0005079 |
| hsa-miR-6894-5p | hsa_circ_0002805 |
| hsa-miR-6894-5p | hsa_circ_0061179 |
| hsa-miR-6894-5p | hsa_circ_0065284 |
| hsa-miR-6894-5p | hsa_circ_0092277 |
| hsa-miR-6894-5p | hsa_circ_0006552 |
| hsa-miR-6894-5p | hsa_circ_0074264 |
| hsa-miR-6894-5p | hsa_circ_0084429 |
| hsa-miR-6894-5p | hsa_circ_0087855 |
| hsa-miR-6894-5p | hsa_circ_0008812 |
| hsa-miR-6894-5p | hsa_circ_0006174 |
| hsa-miR-6894-5p | hsa_circ_0087861 |
| hsa-miR-6894-5p | hsa_circ_0087862 |
| hsa-miR-6894-5p | hsa_circ_0001947 |
| hsa-miR-6894-3p | hsa_circ_0020594 |
| hsa-miR-6894-3p | hsa_circ_0004692 |
| hsa-miR-6895-5p | hsa_circ_0092299 |
| hsa-miR-7106-5p | hsa_circ_0020005 |
| hsa-miR-7106-5p | hsa_circ_0000375 |
| hsa-miR-7106-5p | hsa_circ_0042799 |
| hsa-miR-7106-5p | hsa_circ_0048025 |
| hsa-miR-7106-5p | hsa_circ_0002805 |
| hsa-miR-7106-5p | hsa_circ_0003602 |
| hsa-miR-7106-5p | hsa_circ_0001708 |
| hsa-miR-7106-5p | hsa_circ_0002094 |
| hsa-miR-7106-3p | hsa_circ_0007132 |
| hsa-miR-7107-5p | hsa_circ_0017289 |
| hsa-miR-7107-5p | hsa_circ_0008102 |
| hsa-miR-7107-5p | hsa_circ_0003168 |
| hsa-miR-7107-5p | hsa_circ_0003379 |
| hsa-miR-7107-5p | hsa_circ_0022383 |
| hsa-miR-7107-5p | hsa_circ_0000375 |
| hsa-miR-7107-5p | hsa_circ_0000417 |
| hsa-miR-7107-5p | hsa_circ_0029405 |
| hsa-miR-7107-5p | hsa_circ_0033144 |
| hsa-miR-7107-5p | hsa_circ_0007846 |
| hsa-miR-7107-5p | hsa_circ_0040823 |
| hsa-miR-7107-5p | hsa_circ_0008604 |
| hsa-miR-7107-5p | hsa_circ_0045890 |
| hsa-miR-7107-5p | hsa_circ_0000936 |
| hsa-miR-7107-5p | hsa_circ_0004218 |
| hsa-miR-7107-5p | hsa_circ_0002805 |
| hsa-miR-7107-5p | hsa_circ_0061179 |
| hsa-miR-7107-5p | hsa_circ_0092299 |
| hsa-miR-7107-5p | hsa_circ_0004470 |
| hsa-miR-7107-5p | hsa_circ_0002077 |
| hsa-miR-7107-5p | hsa_circ_0064136 |
| hsa-miR-7107-5p | hsa_circ_0001394 |
| hsa-miR-7107-5p | hsa_circ_0070467 |
| hsa-miR-7107-5p | hsa_circ_0003278 |
| hsa-miR-7107-5p | hsa_circ_0074816 |
| hsa-miR-7107-5p | hsa_circ_0074817 |
| hsa-miR-7107-5p | hsa_circ_0007132 |
| hsa-miR-7107-5p | hsa_circ_0084429 |
| hsa-miR-7107-5p | hsa_circ_0007521 |
| hsa-miR-7107-5p | hsa_circ_0005982 |
| hsa-miR-7107-3p | hsa_circ_0007778 |
| hsa-miR-7107-3p | hsa_circ_0004519 |
| hsa-miR-7107-3p | hsa_circ_0006877 |
| hsa-miR-7107-3p | hsa_circ_0002094 |
| hsa-miR-7108-5p | hsa_circ_0007015 |
| hsa-miR-7108-5p | hsa_circ_0003168 |
| hsa-miR-7108-5p | hsa_circ_0033144 |
| hsa-miR-7108-5p | hsa_circ_0045890 |
| hsa-miR-7108-5p | hsa_circ_0046430 |
| hsa-miR-7108-5p | hsa_circ_0048025 |
| hsa-miR-7108-5p | hsa_circ_0000973 |
| hsa-miR-7108-5p | hsa_circ_0004218 |
| hsa-miR-7108-5p | hsa_circ_0002805 |
| hsa-miR-7108-5p | hsa_circ_0061179 |
| hsa-miR-7108-5p | hsa_circ_0071311 |
| hsa-miR-7108-5p | hsa_circ_0007132 |
| hsa-miR-7108-5p | hsa_circ_0087305 |
| hsa-miR-7108-3p | hsa_circ_0000996 |
| hsa-miR-7109-5p | hsa_circ_0011571 |
| hsa-miR-7109-5p | hsa_circ_0011572 |
| hsa-miR-7109-5p | hsa_circ_0007372 |
| hsa-miR-7109-5p | hsa_circ_0000375 |
| hsa-miR-7109-5p | hsa_circ_0000417 |
| hsa-miR-7109-5p | hsa_circ_0002696 |
| hsa-miR-7109-5p | hsa_circ_0046430 |
| hsa-miR-7109-5p | hsa_circ_0004003 |
| hsa-miR-7109-5p | hsa_circ_0004891 |
| hsa-miR-7109-5p | hsa_circ_0000936 |
| hsa-miR-7109-5p | hsa_circ_0055904 |
| hsa-miR-7109-5p | hsa_circ_0002805 |
| hsa-miR-7109-5p | hsa_circ_0061179 |
| hsa-miR-7109-5p | hsa_circ_0002113 |
| hsa-miR-7109-5p | hsa_circ_0092299 |
| hsa-miR-7109-5p | hsa_circ_0003602 |
| hsa-miR-7109-5p | hsa_circ_0007132 |
| hsa-miR-7109-5p | hsa_circ_0087288 |
| hsa-miR-7109-3p | hsa_circ_0047700 |
| hsa-miR-7109-3p | hsa_circ_0004692 |
| hsa-miR-7109-3p | hsa_circ_0065147 |
| hsa-miR-7109-3p | hsa_circ_0065149 |
| hsa-miR-7109-3p | hsa_circ_0002569 |
| hsa-miR-7109-3p | hsa_circ_0076995 |
| hsa-miR-7109-3p | hsa_circ_0005630 |
| hsa-miR-7110-5p | hsa_circ_0015004 |
| hsa-miR-7110-5p | hsa_circ_0018909 |
| hsa-miR-7110-5p | hsa_circ_0000375 |
| hsa-miR-7110-5p | hsa_circ_0033144 |
| hsa-miR-7110-5p | hsa_circ_0007846 |
| hsa-miR-7110-5p | hsa_circ_0049998 |
| hsa-miR-7110-5p | hsa_circ_0092277 |
| hsa-miR-7110-5p | hsa_circ_0001549 |
| hsa-miR-7110-5p | hsa_circ_0004381 |
| hsa-miR-7110-3p | hsa_circ_0000996 |
| hsa-miR-7110-3p | hsa_circ_0007132 |
| hsa-miR-7111-5p | hsa_circ_0022383 |
| hsa-miR-7111-5p | hsa_circ_0000339 |
| hsa-miR-7111-5p | hsa_circ_0003908 |
| hsa-miR-7111-5p | hsa_circ_0000417 |
| hsa-miR-7111-5p | hsa_circ_0030051 |
| hsa-miR-7111-5p | hsa_circ_0031933 |
| hsa-miR-7111-5p | hsa_circ_0000936 |
| hsa-miR-7111-5p | hsa_circ_0092299 |
| hsa-miR-7111-5p | hsa_circ_0004470 |
| hsa-miR-7111-5p | hsa_circ_0007137 |
| hsa-miR-7111-5p | hsa_circ_0001577 |
| hsa-miR-7111-5p | hsa_circ_0087305 |
| hsa-miR-7111-3p | hsa_circ_0020594 |
| hsa-miR-7111-3p | hsa_circ_0004692 |
| hsa-miR-7111-3p | hsa_circ_0065147 |
| hsa-miR-7111-3p | hsa_circ_0065149 |
| hsa-miR-7111-3p | hsa_circ_0002569 |
| hsa-miR-7111-3p | hsa_circ_0007132 |
| hsa-miR-7111-3p | hsa_circ_0007145 |
| hsa-miR-7111-3p | hsa_circ_0005630 |
| hsa-miR-7112-5p | hsa_circ_0029405 |
| hsa-miR-7112-5p | hsa_circ_0007146 |
| hsa-miR-7112-5p | hsa_circ_0050119 |
| hsa-miR-7112-3p | hsa_circ_0004692 |
| hsa-miR-7112-3p | hsa_circ_0065147 |
| hsa-miR-7112-3p | hsa_circ_0084789 |
| hsa-miR-7113-5p | hsa_circ_0007609 |
| hsa-miR-7113-3p | hsa_circ_0020594 |
| hsa-miR-7113-3p | hsa_circ_0023918 |
| hsa-miR-7113-3p | hsa_circ_0023920 |
| hsa-miR-7113-3p | hsa_circ_0023923 |
| hsa-miR-7113-3p | hsa_circ_0007372 |
| hsa-miR-7113-3p | hsa_circ_0034189 |
| hsa-miR-7113-3p | hsa_circ_0007146 |
| hsa-miR-7113-3p | hsa_circ_0050119 |
| hsa-miR-7113-3p | hsa_circ_0000996 |
| hsa-miR-7113-3p | hsa_circ_0002805 |
| hsa-miR-7113-3p | hsa_circ_0061179 |
| hsa-miR-7113-3p | hsa_circ_0092299 |
| hsa-miR-7113-3p | hsa_circ_0004692 |
| hsa-miR-7113-3p | hsa_circ_0065147 |
| hsa-miR-7113-3p | hsa_circ_0065149 |
| hsa-miR-7113-3p | hsa_circ_0002569 |
| hsa-miR-7113-3p | hsa_circ_0070396 |
| hsa-miR-7113-3p | hsa_circ_0001573 |
| hsa-miR-7113-3p | hsa_circ_0007132 |
| hsa-miR-7113-3p | hsa_circ_0001748 |
| hsa-miR-7113-3p | hsa_circ_0005630 |
| hsa-miR-7113-3p | hsa_circ_0008113 |
| hsa-miR-7114-5p | hsa_circ_0002094 |
| hsa-miR-7114-3p | hsa_circ_0002805 |
| hsa-miR-7114-3p | hsa_circ_0092299 |
| hsa-miR-7114-3p | hsa_circ_0007132 |
| hsa-miR-7114-3p | hsa_circ_0002451 |
| hsa-miR-7114-3p | hsa_circ_0005630 |
| hsa-miR-7150 | hsa_circ_0036763 |
| hsa-miR-7150 | hsa_circ_0000650 |
| hsa-miR-7152-5p | hsa_circ_0007967 |
| hsa-miR-7152-5p | hsa_circ_0007145 |
| hsa-miR-7152-3p | hsa_circ_0007846 |
| hsa-miR-7152-3p | hsa_circ_0046292 |
| hsa-miR-7152-3p | hsa_circ_0061936 |
| hsa-miR-7152-3p | hsa_circ_0001394 |
| hsa-miR-7152-3p | hsa_circ_0084789 |
| hsa-miR-7154-5p | hsa_circ_0007001 |
| hsa-miR-7156-3p | hsa_circ_0000836 |
| hsa-miR-7156-3p | hsa_circ_0049657 |
| hsa-miR-7156-3p | hsa_circ_0092299 |
| hsa-miR-7156-3p | hsa_circ_0092283 |
| hsa-miR-7156-3p | hsa_circ_0007132 |
| hsa-miR-7158-5p | hsa_circ_0038799 |
| hsa-miR-7158-5p | hsa_circ_0045890 |
| hsa-miR-7158-5p | hsa_circ_0048025 |
| hsa-miR-7161-3p | hsa_circ_0009349 |
| hsa-miR-7161-3p | hsa_circ_0009357 |
| hsa-miR-7161-3p | hsa_circ_0011422 |
| hsa-miR-7161-3p | hsa_circ_0015004 |
| hsa-miR-7161-3p | hsa_circ_0020594 |
| hsa-miR-7161-3p | hsa_circ_0023918 |
| hsa-miR-7161-3p | hsa_circ_0023920 |
| hsa-miR-7161-3p | hsa_circ_0023923 |
| hsa-miR-7161-3p | hsa_circ_0030051 |
| hsa-miR-7161-3p | hsa_circ_0035649 |
| hsa-miR-7161-3p | hsa_circ_0000613 |
| hsa-miR-7161-3p | hsa_circ_0008153 |
| hsa-miR-7161-3p | hsa_circ_0007846 |
| hsa-miR-7161-3p | hsa_circ_0000690 |
| hsa-miR-7161-3p | hsa_circ_0045890 |
| hsa-miR-7161-3p | hsa_circ_0008261 |
| hsa-miR-7161-3p | hsa_circ_0006693 |
| hsa-miR-7161-3p | hsa_circ_0001417 |
| hsa-miR-7161-3p | hsa_circ_0074371 |
| hsa-miR-7160-5p | hsa_circ_0006254 |
| hsa-miR-7160-5p | hsa_circ_0003768 |
| hsa-miR-7160-5p | hsa_circ_0049888 |
| hsa-miR-7160-3p | hsa_circ_0092299 |
| hsa-miR-7160-3p | hsa_circ_0092283 |
| hsa-miR-7702 | hsa_circ_0022505 |
| hsa-miR-7703 | hsa_circ_0020594 |
| hsa-miR-7703 | hsa_circ_0045890 |
| hsa-miR-7703 | hsa_circ_0045905 |
| hsa-miR-7703 | hsa_circ_0092299 |
| hsa-miR-7703 | hsa_circ_0007132 |
| hsa-miR-7704 | hsa_circ_0003168 |
| hsa-miR-7704 | hsa_circ_0007846 |
| hsa-miR-7704 | hsa_circ_0046430 |
| hsa-miR-7706 | hsa_circ_0007146 |
| hsa-miR-7706 | hsa_circ_0007132 |
| hsa-miR-7706 | hsa_circ_0082179 |
| hsa-miR-7843-5p | hsa_circ_0017639 |
| hsa-miR-7843-5p | hsa_circ_0033144 |
| hsa-miR-7843-5p | hsa_circ_0045890 |
| hsa-miR-7843-5p | hsa_circ_0045905 |
| hsa-miR-7843-5p | hsa_circ_0002805 |
| hsa-miR-7843-5p | hsa_circ_0061179 |
| hsa-miR-7843-5p | hsa_circ_0072437 |
| hsa-miR-7843-5p | hsa_circ_0005630 |
| hsa-miR-7843-5p | hsa_circ_0084789 |
| hsa-miR-4433b-5p | hsa_circ_0034920 |
| hsa-miR-4433b-3p | hsa_circ_0003168 |
| hsa-miR-4433b-3p | hsa_circ_0003379 |
| hsa-miR-4433b-3p | hsa_circ_0006254 |
| hsa-miR-4433b-3p | hsa_circ_0003768 |
| hsa-miR-4433b-3p | hsa_circ_0022383 |
| hsa-miR-4433b-3p | hsa_circ_0003557 |
| hsa-miR-4433b-3p | hsa_circ_0000417 |
| hsa-miR-4433b-3p | hsa_circ_0000650 |
| hsa-miR-4433b-3p | hsa_circ_0036768 |
| hsa-miR-4433b-3p | hsa_circ_0007846 |
| hsa-miR-4433b-3p | hsa_circ_0048025 |
| hsa-miR-4433b-3p | hsa_circ_0049657 |
| hsa-miR-4433b-3p | hsa_circ_0049998 |
| hsa-miR-4433b-3p | hsa_circ_0061052 |
| hsa-miR-4433b-3p | hsa_circ_0074816 |
| hsa-miR-4433b-3p | hsa_circ_0074817 |
| hsa-miR-4433b-3p | hsa_circ_0001549 |
| hsa-miR-4433b-3p | hsa_circ_0004381 |
| hsa-miR-4433b-3p | hsa_circ_0009092 |
| hsa-miR-4433b-3p | hsa_circ_0084615 |
| hsa-miR-4433b-3p | hsa_circ_0084789 |
| hsa-miR-1273h-5p | hsa_circ_0029405 |
| hsa-miR-1273h-5p | hsa_circ_0082179 |
| hsa-miR-1273h-3p | hsa_circ_0011571 |
| hsa-miR-1273h-3p | hsa_circ_0011572 |
| hsa-miR-1273h-3p | hsa_circ_0020594 |
| hsa-miR-1273h-3p | hsa_circ_0003557 |
| hsa-miR-1273h-3p | hsa_circ_0007846 |
| hsa-miR-1273h-3p | hsa_circ_0000973 |
| hsa-miR-1273h-3p | hsa_circ_0069399 |
| hsa-miR-7845-5p | hsa_circ_0071311 |
| hsa-miR-7846-3p | hsa_circ_0005782 |
| hsa-miR-7846-3p | hsa_circ_0060904 |
| hsa-miR-7847-3p | hsa_circ_0006649 |
| hsa-miR-7847-3p | hsa_circ_0003379 |
| hsa-miR-7847-3p | hsa_circ_0022723 |
| hsa-miR-7847-3p | hsa_circ_0000417 |
| hsa-miR-7847-3p | hsa_circ_0000497 |
| hsa-miR-7847-3p | hsa_circ_0052131 |
| hsa-miR-7847-3p | hsa_circ_0002077 |
| hsa-miR-7847-3p | hsa_circ_0069748 |
| hsa-miR-7847-3p | hsa_circ_0073379 |
| hsa-miR-7847-3p | hsa_circ_0005540 |
| hsa-miR-7847-3p | hsa_circ_0001549 |
| hsa-miR-7847-3p | hsa_circ_0001573 |
| hsa-miR-7847-3p | hsa_circ_0001577 |
| hsa-miR-7847-3p | hsa_circ_0001578 |
| hsa-miR-7847-3p | hsa_circ_0082179 |
| hsa-miR-7847-3p | hsa_circ_0084615 |
| hsa-miR-7847-3p | hsa_circ_0084789 |
| hsa-miR-7848-3p | hsa_circ_0033144 |
| hsa-miR-7850-5p | hsa_circ_0084615 |
| hsa-miR-7851-3p | hsa_circ_0014132 |
| hsa-miR-7851-3p | hsa_circ_0029405 |
| hsa-miR-7851-3p | hsa_circ_0034189 |
| hsa-miR-7851-3p | hsa_circ_0000836 |
| hsa-miR-7855-5p | hsa_circ_0003379 |
| hsa-miR-7855-5p | hsa_circ_0000375 |
| hsa-miR-7855-5p | hsa_circ_0087288 |
| hsa-miR-7974 | hsa_circ_0007132 |
| hsa-miR-7974 | hsa_circ_0001756 |
| hsa-miR-8052 | hsa_circ_0046430 |
| hsa-miR-8052 | hsa_circ_0002094 |
| hsa-miR-8052 | hsa_circ_0001829 |
| hsa-miR-8060 | hsa_circ_0047347 |
| hsa-miR-8060 | hsa_circ_0000909 |
| hsa-miR-8060 | hsa_circ_0005571 |
| hsa-miR-8060 | hsa_circ_0004712 |
| hsa-miR-8069 | hsa_circ_0005371 |
| hsa-miR-8069 | hsa_circ_0007015 |
| hsa-miR-8069 | hsa_circ_0000417 |
| hsa-miR-8069 | hsa_circ_0046430 |
| hsa-miR-8069 | hsa_circ_0004003 |
| hsa-miR-8069 | hsa_circ_0004891 |
| hsa-miR-8069 | hsa_circ_0002926 |
| hsa-miR-8069 | hsa_circ_0004699 |
| hsa-miR-8069 | hsa_circ_0061052 |
| hsa-miR-8069 | hsa_circ_0002077 |
| hsa-miR-8069 | hsa_circ_0072437 |
| hsa-miR-8069 | hsa_circ_0074816 |
| hsa-miR-8069 | hsa_circ_0074817 |
| hsa-miR-8069 | hsa_circ_0007132 |
| hsa-miR-8069 | hsa_circ_0004712 |
| hsa-miR-8069 | hsa_circ_0082179 |
| hsa-miR-8069 | hsa_circ_0002094 |
| hsa-miR-8071 | hsa_circ_0046430 |
| hsa-miR-8071 | hsa_circ_0004705 |
| hsa-miR-8072 | hsa_circ_0002402 |
| hsa-miR-8072 | hsa_circ_0007846 |
| hsa-miR-8072 | hsa_circ_0045890 |
| hsa-miR-8072 | hsa_circ_0048025 |
| hsa-miR-8072 | hsa_circ_0006877 |
| hsa-miR-8072 | hsa_circ_0001134 |
| hsa-miR-8072 | hsa_circ_0061052 |
| hsa-miR-8072 | hsa_circ_0092299 |
| hsa-miR-8072 | hsa_circ_0092277 |
| hsa-miR-8072 | hsa_circ_0002094 |
| hsa-miR-8072 | hsa_circ_0001824 |
| hsa-miR-8072 | hsa_circ_0008934 |
| hsa-miR-8073 | hsa_circ_0003557 |
| hsa-miR-8073 | hsa_circ_0004218 |
| hsa-miR-8073 | hsa_circ_0002805 |
| hsa-miR-8073 | hsa_circ_0061179 |
| hsa-miR-8073 | hsa_circ_0092277 |
| hsa-miR-8073 | hsa_circ_0001400 |
| hsa-miR-8073 | hsa_circ_0007132 |
| hsa-miR-8073 | hsa_circ_0075748 |
| hsa-miR-8073 | hsa_circ_0003340 |
| hsa-miR-8073 | hsa_circ_0009092 |
| hsa-miR-8074 | hsa_circ_0092283 |
| hsa-miR-8075 | hsa_circ_0017446 |
| hsa-miR-8075 | hsa_circ_0000206 |
| hsa-miR-8075 | hsa_circ_0022392 |
| hsa-miR-8075 | hsa_circ_0008916 |
| hsa-miR-8075 | hsa_circ_0007846 |
| hsa-miR-8075 | hsa_circ_0085923 |
| hsa-miR-8077 | hsa_circ_0000497 |
| hsa-miR-8077 | hsa_circ_0033144 |
| hsa-miR-8077 | hsa_circ_0002926 |
| hsa-miR-8078 | hsa_circ_0029405 |
| hsa-miR-8078 | hsa_circ_0045890 |
| hsa-miR-8078 | hsa_circ_0045905 |
| hsa-miR-8078 | hsa_circ_0092299 |
| hsa-miR-8085 | hsa_circ_0046430 |
| hsa-miR-8085 | hsa_circ_0001829 |
| hsa-miR-8089 | hsa_circ_0009581 |
| hsa-miR-8089 | hsa_circ_0006837 |
| hsa-miR-8089 | hsa_circ_0017248 |
| hsa-miR-8089 | hsa_circ_0006649 |
| hsa-miR-8089 | hsa_circ_0003379 |
| hsa-miR-8089 | hsa_circ_0000417 |
| hsa-miR-8089 | hsa_circ_0000497 |
| hsa-miR-8089 | hsa_circ_0031933 |
| hsa-miR-8089 | hsa_circ_0034189 |
| hsa-miR-8089 | hsa_circ_0000592 |
| hsa-miR-8089 | hsa_circ_0003838 |
| hsa-miR-8089 | hsa_circ_0002696 |
| hsa-miR-8089 | hsa_circ_0040921 |
| hsa-miR-8089 | hsa_circ_0092337 |
| hsa-miR-8089 | hsa_circ_0044195 |
| hsa-miR-8089 | hsa_circ_0045890 |
| hsa-miR-8089 | hsa_circ_0046430 |
| hsa-miR-8089 | hsa_circ_0000836 |
| hsa-miR-8089 | hsa_circ_0048025 |
| hsa-miR-8089 | hsa_circ_0002926 |
| hsa-miR-8089 | hsa_circ_0050119 |
| hsa-miR-8089 | hsa_circ_0000918 |
| hsa-miR-8089 | hsa_circ_0054086 |
| hsa-miR-8089 | hsa_circ_0000996 |
| hsa-miR-8089 | hsa_circ_0055904 |
| hsa-miR-8089 | hsa_circ_0092297 |
| hsa-miR-8089 | hsa_circ_0003854 |
| hsa-miR-8089 | hsa_circ_0004218 |
| hsa-miR-8089 | hsa_circ_0061052 |
| hsa-miR-8089 | hsa_circ_0002805 |
| hsa-miR-8089 | hsa_circ_0061179 |
| hsa-miR-8089 | hsa_circ_0061936 |
| hsa-miR-8089 | hsa_circ_0092299 |
| hsa-miR-8089 | hsa_circ_0064136 |
| hsa-miR-8089 | hsa_circ_0003602 |
| hsa-miR-8089 | hsa_circ_0092277 |
| hsa-miR-8089 | hsa_circ_0001394 |
| hsa-miR-8089 | hsa_circ_0006693 |
| hsa-miR-8089 | hsa_circ_0001417 |
| hsa-miR-8089 | hsa_circ_0001439 |
| hsa-miR-8089 | hsa_circ_0072857 |
| hsa-miR-8089 | hsa_circ_0073379 |
| hsa-miR-8089 | hsa_circ_0005540 |
| hsa-miR-8089 | hsa_circ_0001573 |
| hsa-miR-8089 | hsa_circ_0007132 |
| hsa-miR-8089 | hsa_circ_0075748 |
| hsa-miR-8089 | hsa_circ_0003340 |
| hsa-miR-8089 | hsa_circ_0002094 |
| hsa-miR-8089 | hsa_circ_0005630 |
| hsa-miR-8089 | hsa_circ_0084429 |
| hsa-miR-8089 | hsa_circ_0001806 |
| hsa-miR-8089 | hsa_circ_0084789 |
| hsa-miR-8089 | hsa_circ_0087288 |
| hsa-miR-8089 | hsa_circ_0087305 |
| hsa-miR-8089 | hsa_circ_0087855 |
| hsa-miR-8089 | hsa_circ_0008812 |
| hsa-miR-8089 | hsa_circ_0006174 |
| hsa-miR-8089 | hsa_circ_0087861 |
| hsa-miR-8089 | hsa_circ_0087862 |
| hsa-miR-8089 | hsa_circ_0092125 |
